# Synthesis of Alkyl Substituted Carbatripyrrins and Their Application to the Preparation of Carbaporphyrins and Oxacarbaporphyrins

**DOI:** 10.1021/acsomega.5c07422

**Published:** 2025-10-27

**Authors:** Ian A. McLauchlan, Bethany K. X. Overbey, Tyler J. Smolczyk, John J. Woods, Timothy D. Lash

**Affiliations:** Department of Chemistry, 6049 Illinois State University, Normal, Illinois 61790-4160, United States

## Abstract

Unsubstituted carbatripyrrins have previously been shown to be useful intermediates in the synthesis of benzocarbaporphyrins and related core-modified analogues. However, the absence of substituents often resulted in the formation of poorly soluble products. In this study, four examples alkyl substituted carbatripyrrins were constructed by base-catalyzed condensation of dihydrofulvenes with pyrrole aldehydes. Further reaction with a pyrrole dialdehyde in the presence of trifluoroacetic acid afforded a series of carbaporphyrins, while reactions with 2,5-furandicarboxaldehyde generated oxacarbaporphyrins. These results demonstrate that alkyl substituted carbatripyrrins are promising precursors to new carbaporphyrinoid structures.

## Graphical abstract

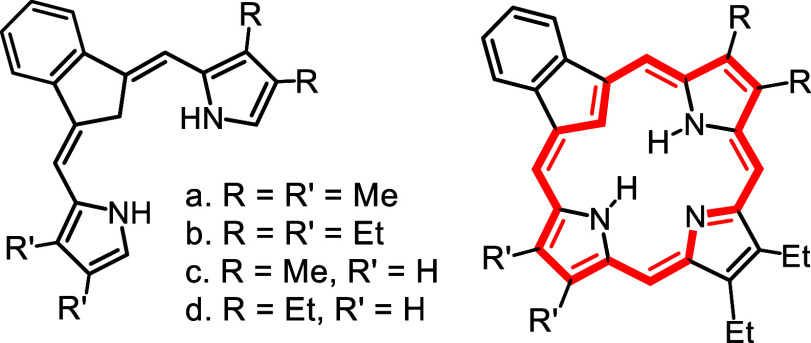

## 1 Introduction

Porphyrins are widely studied due in part to their biological significance,^1,2^ medicinal applications,^3,4^ catalytic activity^5^ and utility in sensor applications.^6^ This has led to investigations into related macrocyclic systems, including core modified structures,^7^ porphyrinoids with fused aromatic rings,^8^ N-confused porphyrins,^9^ contracted porphyrins^10^ and expanded porphyrins.^11^ Our group has focused on the synthesis and properties of carbaporphyrins and related systems,^12^ including benzocarbaporphyrins **1** ^13^ and azuliporphyrins **2** (Scheme 1).^14^ Carbaporphyrinoids systems display many unique properties undergoing unusual oxidation reactions^15^ and generating organometallic derivatives under mild conditions.^16^ A common route to these systems involves the condensation of tripyrrane dicarboxylic acids **3** with dialdehydes such as **4** and **5** in the presence of trifluoroacetic acid (TFA), followed by oxidation with 2,3-dichloro-5,6-dicyano-1,4-benzoquinone (DDQ) or ferric chloride (Scheme 1).^17^ Although this versatile approach has provided access to numerous porphyrinoid structures, the oxidation step may lead to complications and alternative routes have been considered. As carbaporphyrins contain fulvene subunits, structures of this type can act as alternative precursors to carbaporphyrin-type structures.

**Scheme 1:**
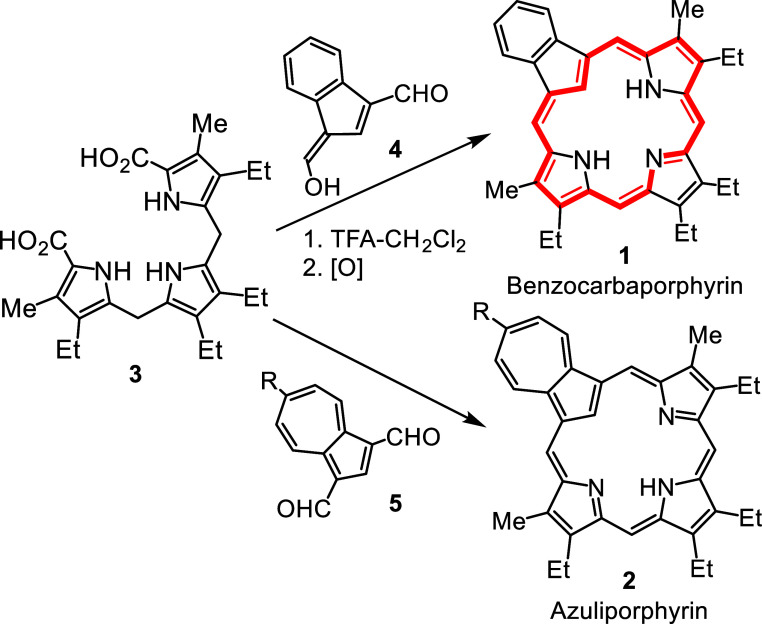
Synthesis of Carbaporphyrins from Tripyrranes

Fulvene (**6**), sometimes referred to as pentafulvene to denote the presence of a cyclopentadiene component, is a 6π electron isomer of benzene exhibiting a small dipole moment that can be attributed to dipolar canonical forms such as **6′** (Scheme 2).^18^ Although the latter structure has a cyclopentadienyl anion component, simple fulvenes are considered to be nonaromatic. However, substituted fulvenes can take on significant aromatic character.^19^ Calicene provides a particularly pertinent example as this can introduce two aromatic components, a cyclopentadienyl anion and a cyclopropenyl cation (Scheme 2); nevertheless, the parent structure is not presently known, although hexaphenylcalicene has been described,^20^ and the calculated resonance stabilization energy for **7** is low.^21,22^ Fulvenes aldehydes are important intermediates in the synthesis of oxacarba-^23^ and dicarbaporphyrins.^24^ In an earlier study, indene was reacted with pyrrole-2-carboxaldehyde (**7**) in the presence of potassium hydroxide in refluxing ethanol to give fulvene **8** and this was reduced with lithium aluminum hydride in refluxing THF to generate the related dihydrofulvene **9** (Scheme 3).^25^ We envisaged that further base catalyzed reaction of **9** with **7** would give dipyrrolic derivatives **10** that could be utilized in the construction of carbaporphyrinoid systems. Initially, complex mixtures of products were obtained that appeared to contain mixtures of *E* and *Z* isomers corresponding to **10** but only the *E*-isomer would have the appropriate geometry to afford macrocyclic products. A small amount of a relatively insoluble material was also noted, and further analysis showed that this corresponded to carbatripyrrin **11**. This structure, although unexpected, was intriguing as it has the correct geometry for generating carbaporphyrinoid products. Unfortunately, only very small amounts of **11** were generated in the initial studies, but we reasoned that if **10** and **11** were in equilibrium under the reaction conditions, it might be possible to direct the reaction toward the desired product. In essence, this is an application of Le Chatelier’s principle as precipitation of poorly soluble **11** would drive the equilibrium toward this product. This was accomplished by running the reaction under far more concentrated conditions and resulted in **11** being isolated in 74% yield.^25^ The carbatripyrrin proved to be a suitable precursor to carbaporphyrins and an oxidation step was no longer required as the products were generated at the correct oxidation state. Reactions of **11** with heterocyclic dicarbinols also enabled the syntheses of diphenylcarbaporphyrinoids and the chemistry was adapted to prepared the first examples **12** of porphyrin analogues with 4 different elements enclosed within a porphyrin-type core (Figure 1).^26,27^ The strategy also allowed the synthesis of carbaporphyrins with fused aromatic rings such as phenanthrocarbaporphyrin **13**.^25,28^ In addition, these studies provided access to the parent aromatic systems, including unsubstituted benzocarbaporphyrin **1a**.^28^ The methodology proved to be versatile but the absence of substituents often afforded products with poor solubilities and this limited their applications.

**Scheme 2:**
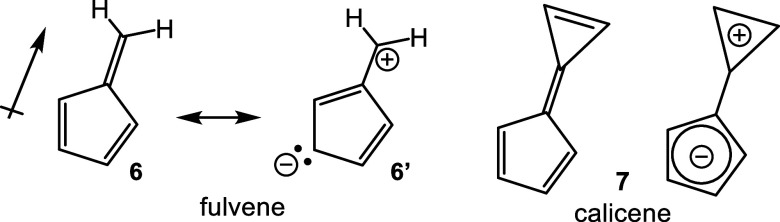
Fulvenes and Potential Resonance Contributors

**Scheme 3:**
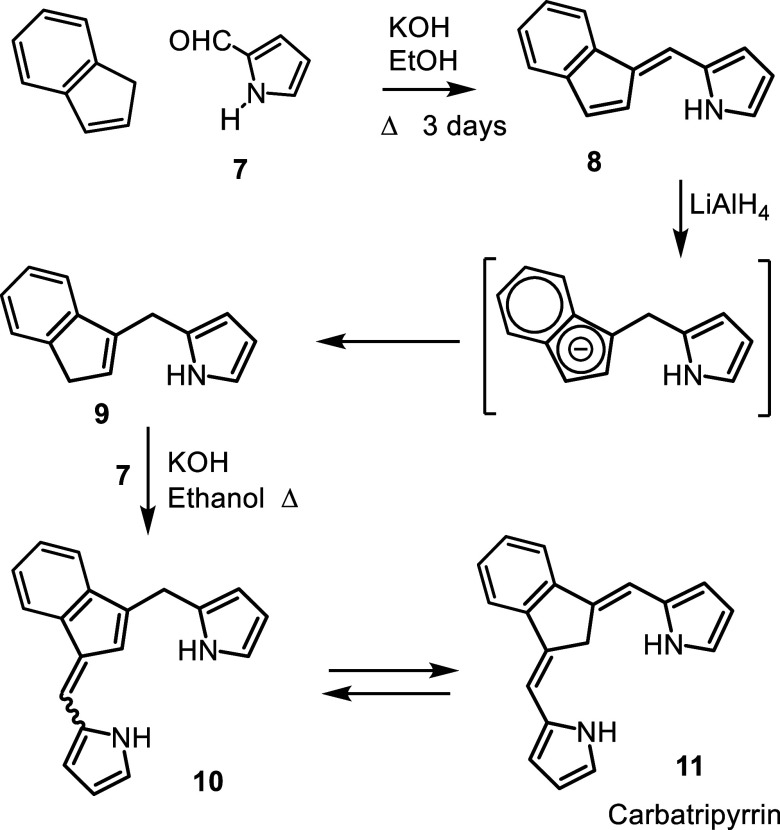
Synthesis of a Carbatripyrrin

**Figure 1:**
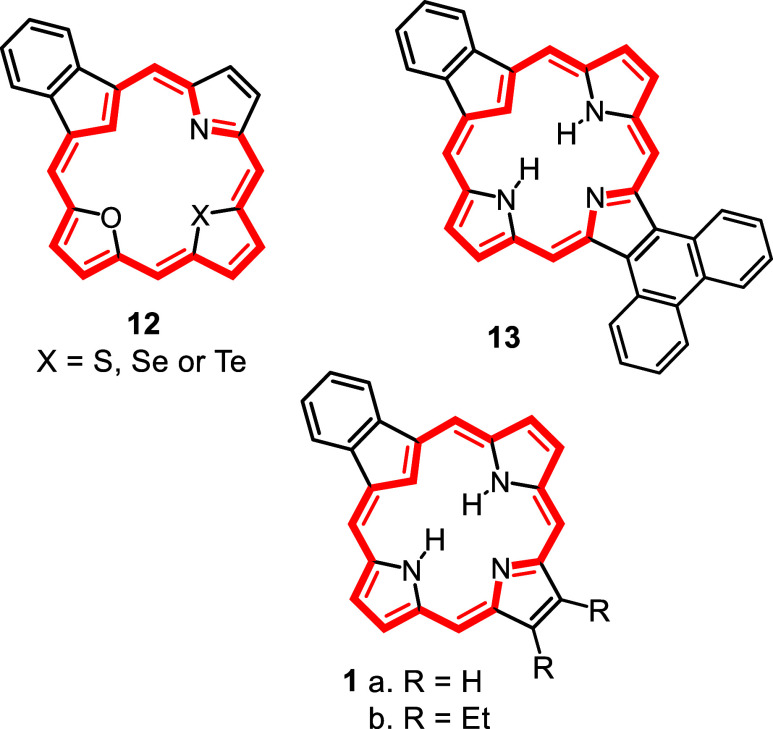
Selected carbaporphyrins and heterocarbaporphyrin structures.

In order to overcome this limitation, the synthesis of carbatripyrrins with alkyl substituents was investigated. It was anticipated that the presence of these substituents would improve the solubility of the porphyrinoid products. However, a major flaw in this strategy is that the formation of **11** relies upon its limited solubility as it is necessary for it to precipitate out in order to shift the equilibrium in the desired direction. As the alkyl substituents may also enhance the solubility of the carbatripyrrins, there was a distinct possibility that this could block access to these crucial intermediates. This concern proved to be valid, but these difficulties could be overcome under suitably modified conditions.

## 2 Results and Discussion

Initially, the synthesis of tetramethylcarbatripyrrin **14a** (Scheme 4) was investigated. Technical grade indene was reacted with 3,4-dimethylpyrrole-2-carboxaldehyde (**15a**) and potassium hydroxide in refluxing ethanol to give fulvene **16a** in 66% yield. The fulvene was isolated as an orange solid and was fully characterized by ^1^H and ^13^C NMR, as well as HRMS. Fulvenes such as **16a** represent almost 50% of a carbaporphyrin structure and provide a useful comparison. The proton NMR spectrum for **16a** (Figure 2) gave a broad resonance for the NH at 8.3 ppm, while the benzo-unit produced multiplets at 7.72–7.69 (1H), 7.38–7.34 (1H) and 7.23–7.20 ppm (2H). The indene protons at positions 1 and 2 gave rise to peaks at 6.91 ppm (d) and 7.01 (dd), respectively. The latter resonance shows long-range coupling to the bridging methine proton (6-H) which appears as a broadened resonance at 7.33 ppm. The pyrrole C–H (5′-H) afforded a doublet (*J* = 2.4 Hz) at 6.78 ppm due to coupling with the N–H. The methyl substituents at positions 3′ and 4′ gave peaks at 2.21 and 2.06 ppm, the latter appearing as a weakly coupled doublet (*J* = 0.7 Hz) due to long-range interactions with the N–H. In carbaporphyrins, the chemical shifts for the analogous protons are vastly altered due to the strongly diatropic ring currents for these macrocycles.^29^

**Scheme 4:**
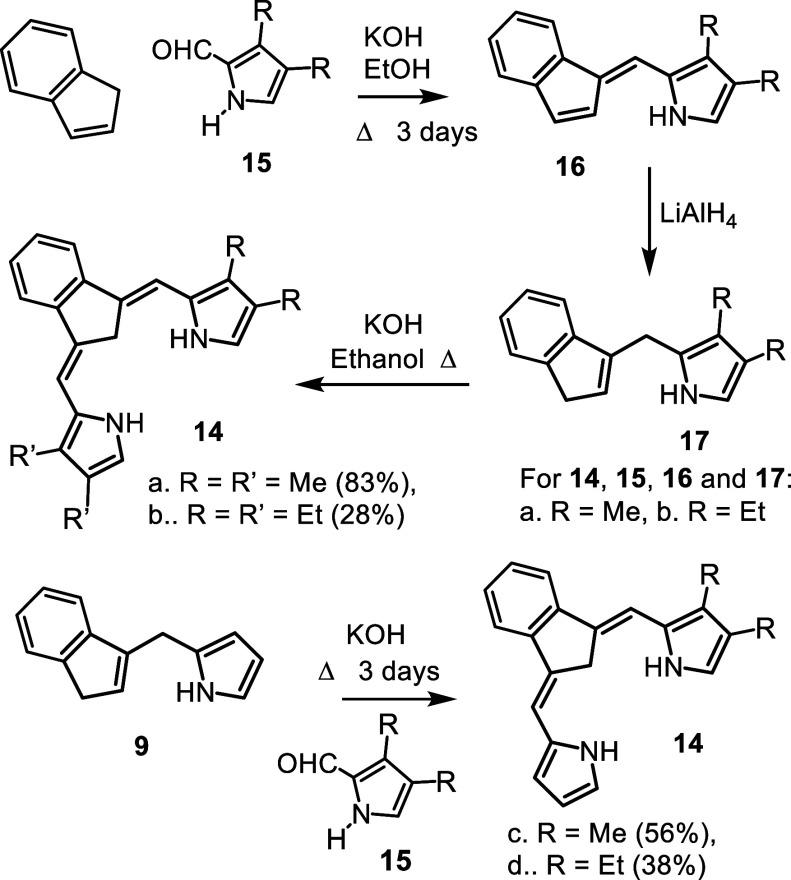
Synthesis of Alkyl Substituted Carbatripyrrins

**Figure 2:**
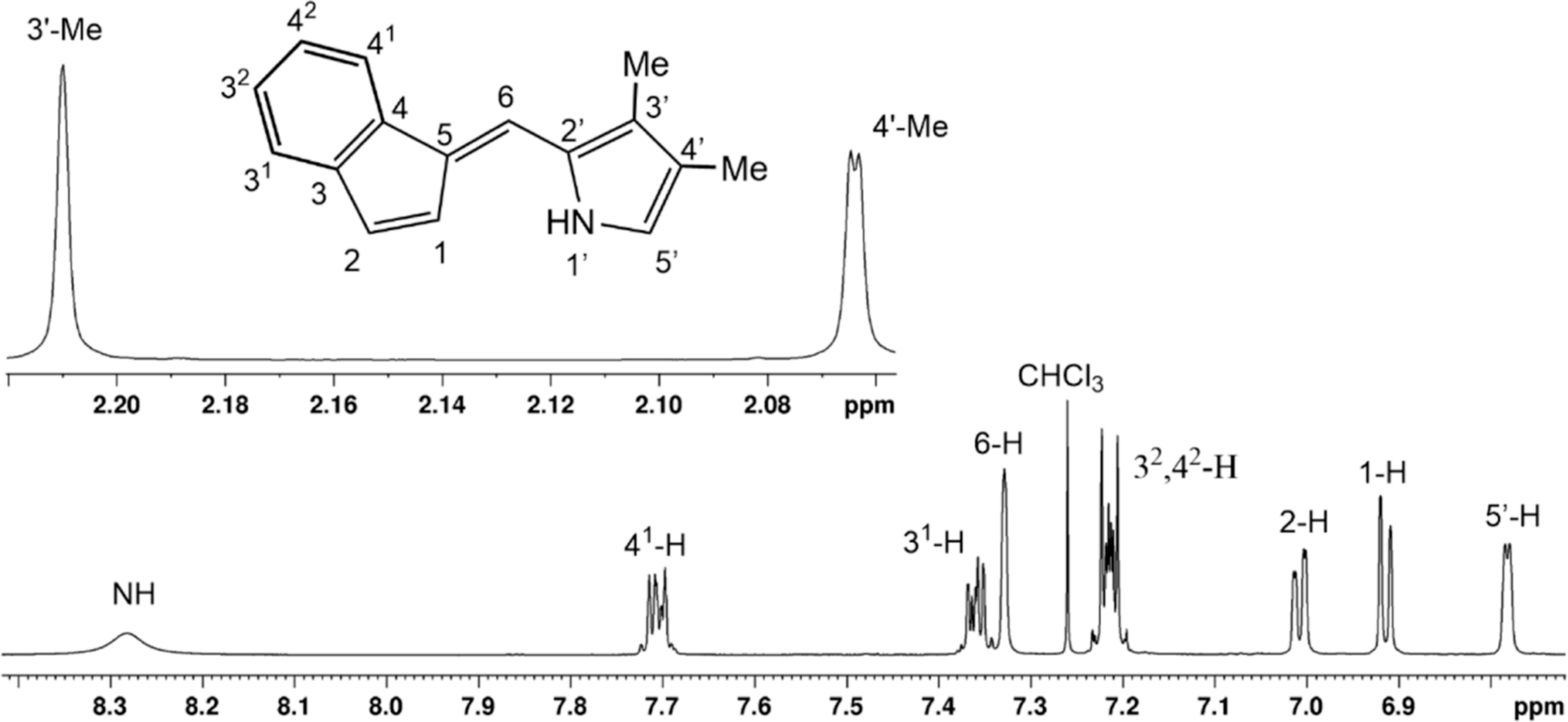
Proton NMR spectrum of fulvene **16a** in CDCl_3_.

Treatment of **16a** with lithium aluminum hydride in refluxing THF, followed by purification of the crude product by column chromatography, afforded the related dihydrofulvene **17a** as an oil. This was further reacted with **15a** and KOH in refluxing ethanol under the conditions used to prepare carbatripyrrin **11**. However, the desired product **14a** could not be isolated and orange colored oils corresponding to complex mixtures were obtained instead. Addition of water to reduce the solubility of these compounds did not give useful results and alternative solvents such as methanol or 2-propanol were also ineffective. However, when the solvent volume was further reduced so that there was barely enough to dissolve the reactants (see Experimental Section), carbatripyrrin **14a** precipitated out in up to 83% yield. The same strategy was applied to the synthesis of tetraethylcarbatripyrrin **14b**, although this proved to be more of a challenge. Reaction of 3,4-diethylpyrrole-2-carboxaldehyde (**15b**) with indene gave fulvene **16b** in 74% yield and this could be reduced with lithium aluminum hydride to give the corresponding dihydrofulvene **17b**. However, the targeted carbatripyrrin **14b** could not be isolated under the conditions used to prepare **14a**. Eventually, an acceptable yield of **14b** was obtained by carrying out the reaction of **15b** with **17b** in 20% water-80% ethanol. The amount of water present was important as larger quantities led to decomposition. Although **14a** and **14b** were obtained in reasonably pure form, they proved to be somewhat unstable and needed to be stored in the freezer to avoid decomposition. Unsubstituted dihydrofulvene **9** was also reacted with pyrrole aldehydes **15a** and **15b** to give dialkylcarbatripyrrins **14c** and **14d** and this produced satisfactory results, affording 56% and 38% yields, respectively (Scheme 4). Carbatripyrrins **14c** and **14d** were stable and could be stored indefinitely at room temperature without noticeable decomposition.

In order to assess the utility of carbatripyrrins **14a**–**d**, they were used to prepare a series of carbaporphyrins **18** and oxacarbaporphyrins **19** (Scheme 5). TFA was added to a stirred solution of **14a**–**d** and pyrrole dialdehyde **20** in dichloromethane and the resulting mixture was allowed to react at room temperature for 30 min. Following workup, purification by column chromatography and recrystallization from chloroform–methanol, the carbaporphyrin products were isolated in 35–65% yield. Carbatripyrrins **14a**–**d** were similarly reacted with 2,5-furandicarboxaldehyde (**21**) to give oxacarbaporphyrins **19a**–**d** in 29–53% yield.

**Scheme 5:**
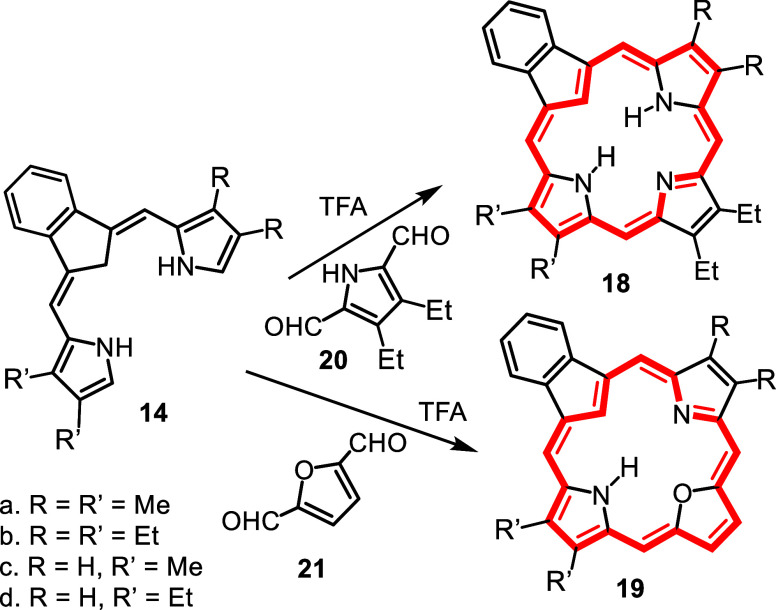
Synthesis of Carba- and Oxacarbaporphyrins

Carbaporphyrins **1** usually give orange-brown solutions in CH_2_Cl_2_ or CHCl_3_.^29^ However, disubstituted carbaporphyrin **1b** (Figure 1)^15^ was reported to give green solutions even though the chromophore is the same. For the new carbaporphyrins, **18c** gave greenish-yellow solutions while **18a**, **18b** and **148** afforded the usual orange-brown colored solutions. Clearly, the alkyl groups are acting as weak auxochromes and analysis of the observed UV–visible absorptions indicate that the effects are subtle (Table 1). Comparing the results with unsubstituted benzocarbaporphyrin **1a**,^28^ diethylcarbaporphyrin **1b** shows only minor shifts but **18b**–**d** all exhibit bathochromic shifts to Q bands 3 and 4, as well as to the Soret band. Tetraethylcarbaporphyrin **18d** gives similar absorptions to hexaalkylcarbaporphyrins **18a** and **18b**, but the related dimethyl carbaporphyrin **18c** gives intermediary results. The data indicates that alkyl substituents shift the absorptions to slightly longer wavelengths and that ethyl groups are more effective than methyl in inducing these changes, presumably due to their greater electron-donating abilities. The UV–vis spectrum for **18b** gave the Soret band at 423 nm and four Q bands (Figure 3). Addition of trace amounts of TFA led to protonation of an interior nitrogen to give the related monocation **18**H^+^ (Scheme 6) and this showed a weakened Soret band at 436 nm and Q bands at 472, 547, and 608 nm (Figure 3). Further changes were noted at higher concentration of acid. In 50% TFA-CH_2_Cl_2_, a new species was generated giving a Soret band at 426 nm and a moderately strong Q-band at 671 nm (Figure 4) and this can be attributed to the formation of a C-protonated dication **18**H_2_^2+^ (Scheme 6).^29^ Similar results were obtained for **18a**, **18c** and **18d**.

**Table 1:** UV–Vis Absorptions for Selected Carbaporphyrins with CH_2_Cl_2_

	soret	Q4	Q3	Q2	Q1
**18a**	423	509	544	602	662
**18b**	424	510	544	602	662
**18c**	422	504	537	602	661
**18d**	423	510	544	603	661
**1b** ^ref^	419	500	shoulder	603	663
**1a** ^ref^	418	503	534	602	660

**Figure 3:**
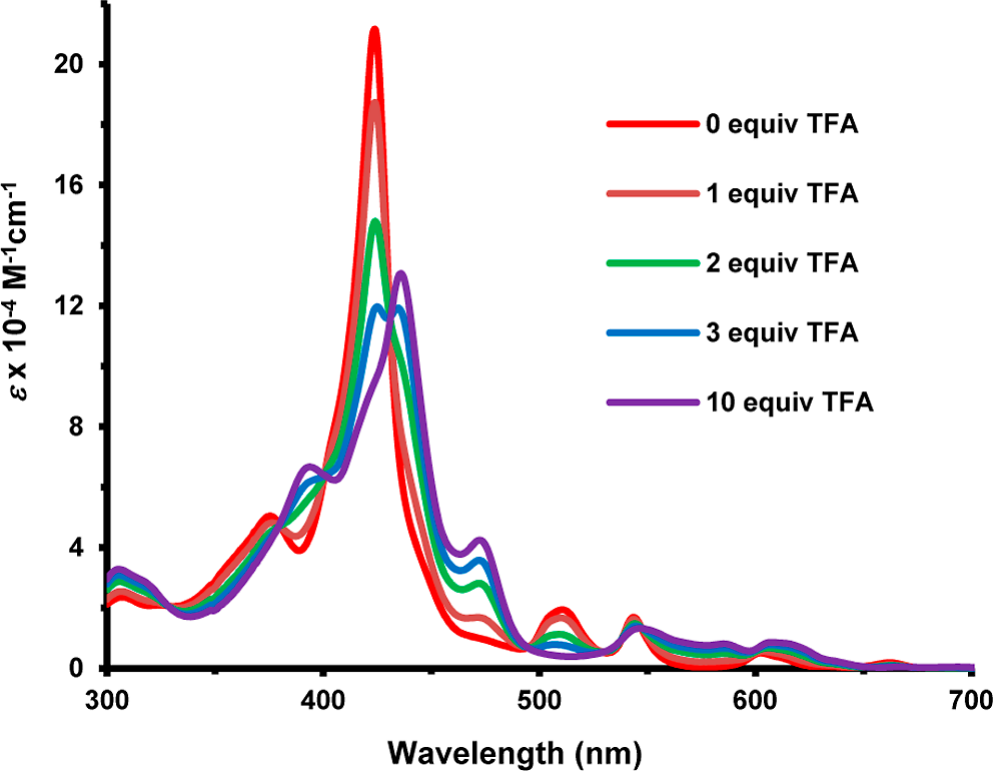
UV–vis spectra of carbaporphyrins **18b** in CH_2_Cl_2_ with 0–10 equiv of TFA.

**Scheme 6:**
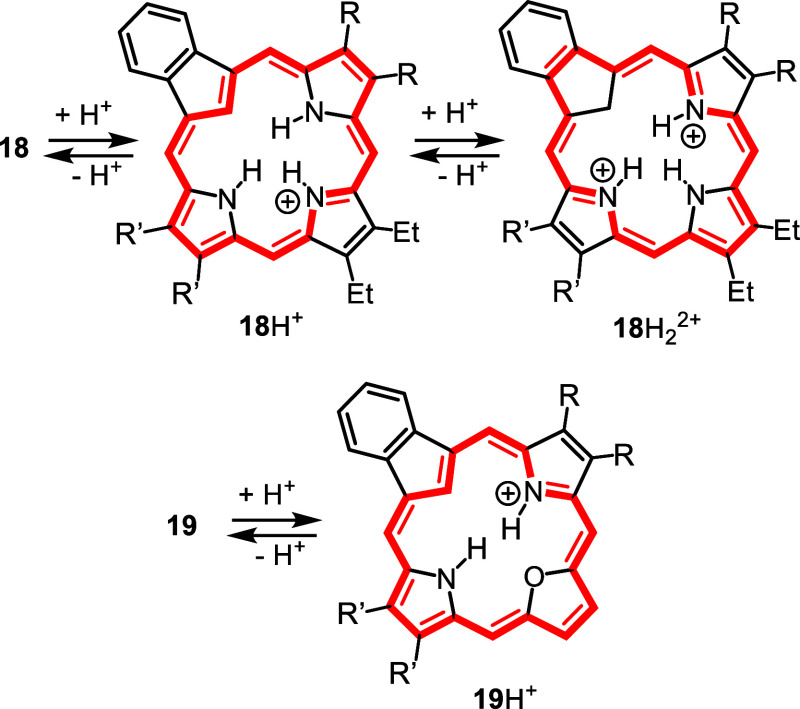
Protonation of Carbaporphyrins and Oxacarbaporphyrins

**Figure 4:**
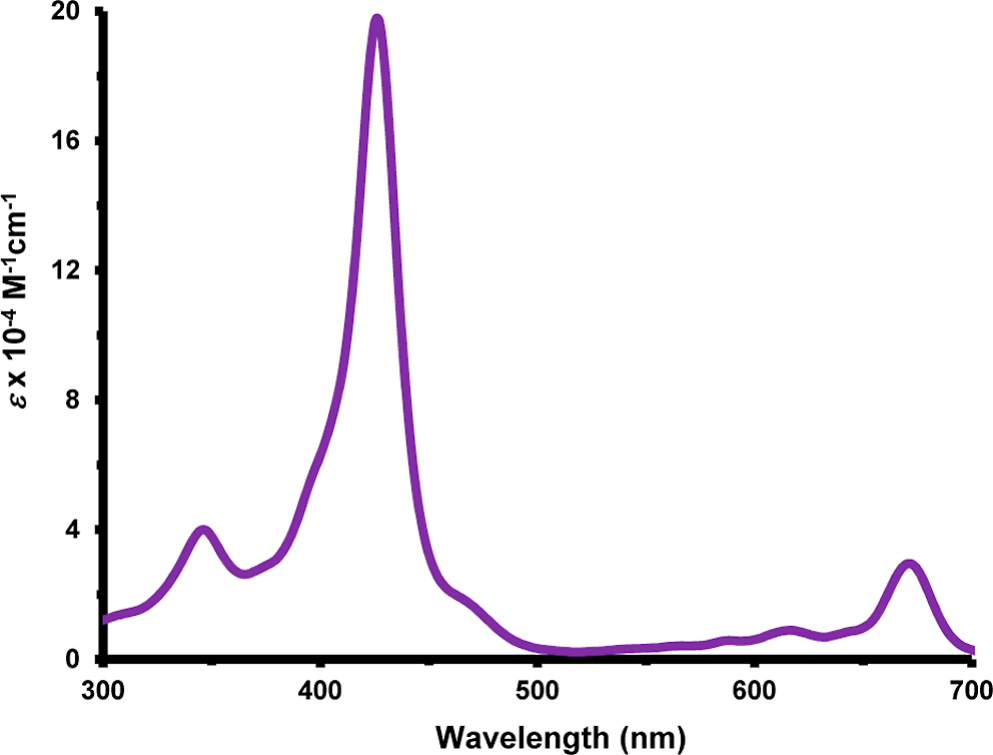
UV–vis spectrum of carbaporphyrin **18b** in 50%TFA-CH_2_Cl_2_.

The proton NMR spectrum of **18b** demonstrated the manifestation of a strong diamagnetic ring current, showing the presence of four strongly deshielded *meso*-protons as two 2H singlets at 10.14 and 9.84 ppm, while the internal NH and CH protons were strongly shielded to giving rise to peaks at −3.87 and −6.65 ppm, respectively (Figure 5). A useful gauge of aromatic character in macrocyclic systems is the difference in chemical shifts between the internal and external protons (Δδ) and in this case the value is 16.79 ppm. The strong ring current also results in comparatively downfield shifts to the alkyl substituents and the CH_2_ resonances appeared between 4.15 and 3.98 ppm. Addition of trace amounts of TFA gave the corresponding monocation **18b**H^+^ and the proton NMR spectrum for this species (Figure 6) indicated that it has a slightly enhanced aromatic ring current with the external *meso*-protons showing up at 10.32 (2H) and 10.05 ppm (2H), while the interior CH appeared at −6.71 ppm (Δδ 17.03 ppm). The carbon-13 NMR spectrum for **18b** showed the *meso*-carbons at 95.6 and 98.8 ppm, while the internal carbon (21-CH) was located at 109.5 ppm. The simplicity of the NMR spectra for both the free base and protonated forms of **18b** also demonstrated the presence of *a* plane of symmetry. However, carbaporphyrins **18c** and **18d** are asymmetrical and this results in a duplication of the NMR resonances. The proton NMR spectrum for **18d** in CDCl_3_ (Figure 7) gave four 1H singlets for the *meso*-protons at 10.10, 10.00, 9.81, and 9.71 ppm, while the internal CH appeared at −6.84 ppm (Δδ 16.94). The external pyrrolic protons due to the unsubstituted ring gave rise to two doublets at 9.25 and 9.16 ppm (*J* = 4.4 Hz).

**Figure 5:**
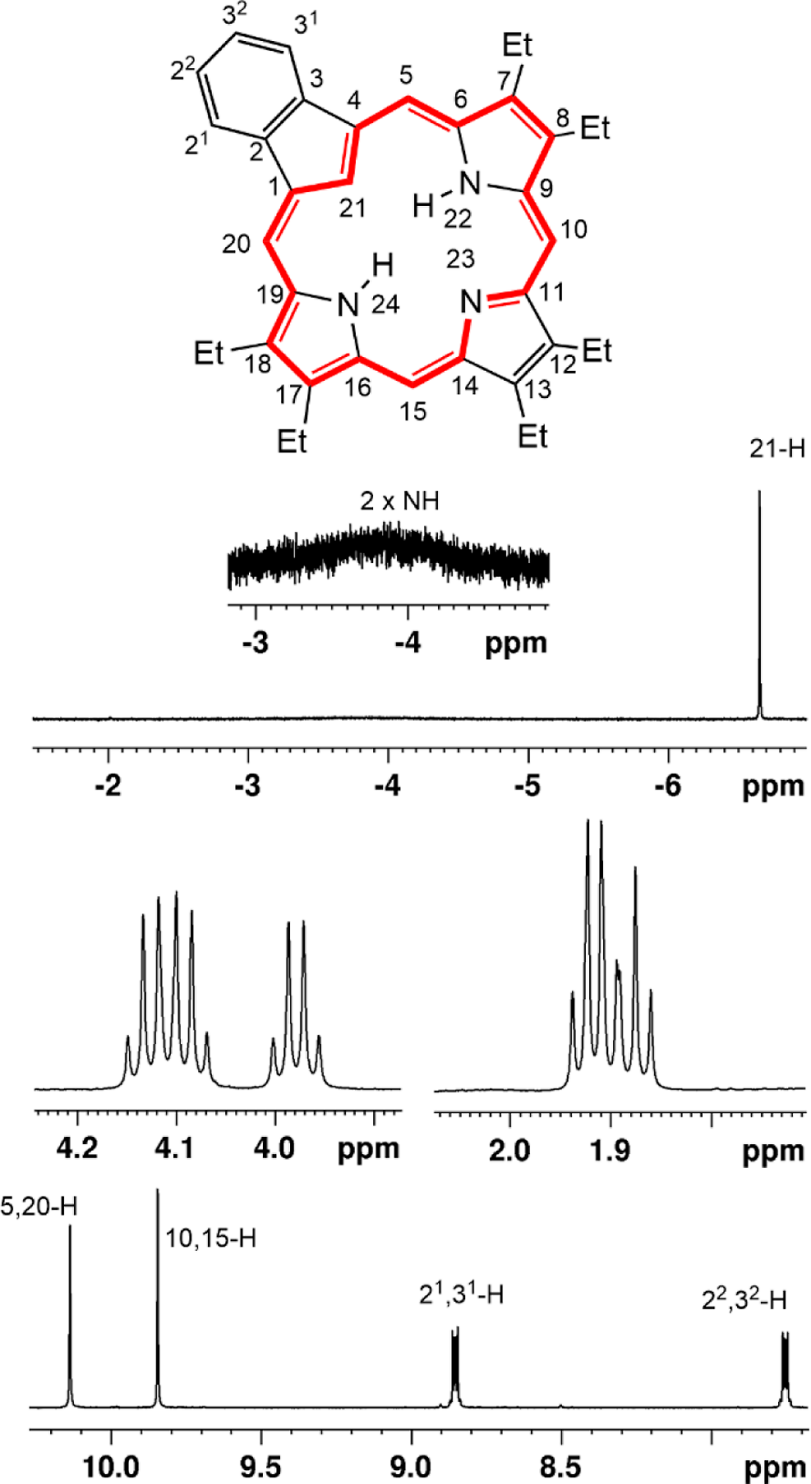
Proton NMR spectrum of carbaporphyrin **18b** in CDCl_3_.

**Figure 6:**
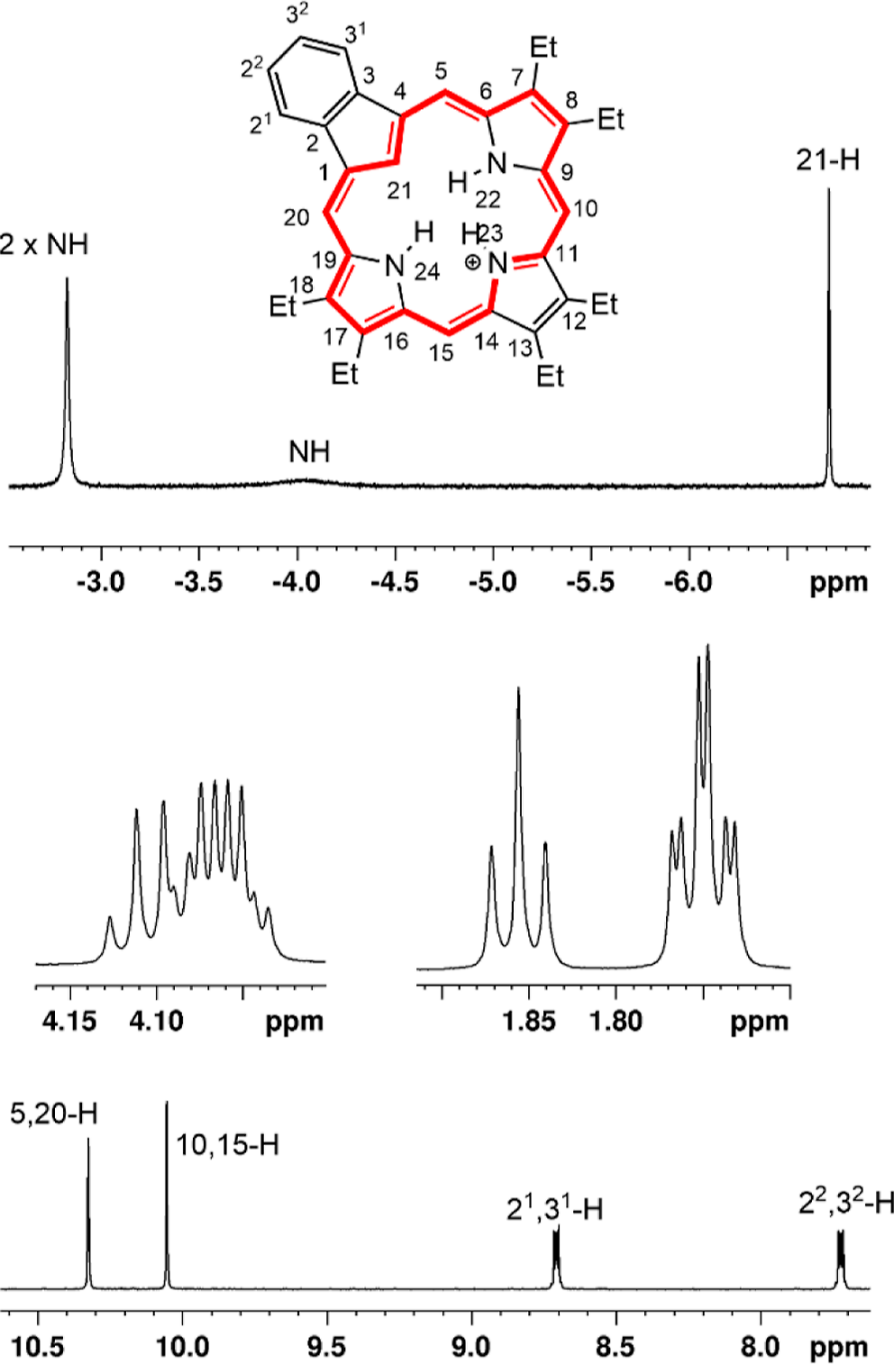
Proton NMR spectrum of carbaporphyrin monocation **18b**H^+^ in CDCl_3_ containing 2 μL TFA.

**Figure 7:**
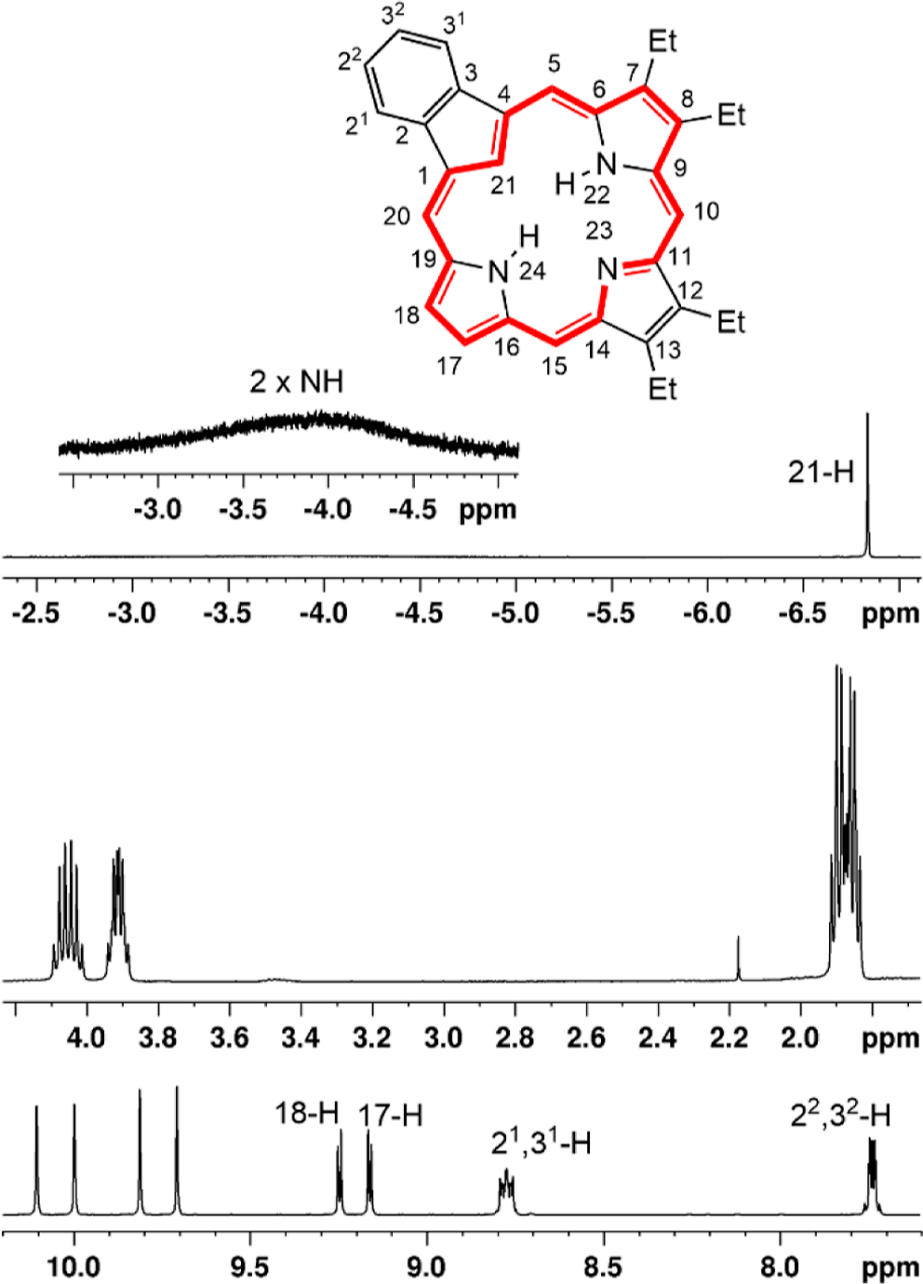
Proton NMR spectrum of carbaporphyrin **18d** in CDCl_3_.

Oxacarbaporphyrins **19** exhibited similar aromatic characteristics. The UV–vis spectrum for oxacarbaporphyrin **19b** gave a broad Soret band at 423 nm and Q bands at 508, 544, 603, and 662 nm (Figure 8). Addition of trace amounts of TFA resulted in monoprotonation to give **19b**H^+^ (Scheme 6) and this resulted the appearance of multiple absorptions between 400 and 500 nm and smaller broadened peaks near 600 nm (Figure 8). Further changes were noted at higher acid concentrations. Similar spectra were obtained for **19a**, **19c** and **19d**. The proton NMR spectrum for **19a** gave two 2H singlets for the *meso*-protons at 10.11 and 9.85 ppm, while the furan protons appeared at 9.58 ppm and the internal CH resonance was located at −4.69 ppm (Δδ 15.0 ppm). The corresponding monocation **19a**H^+^ (Scheme 6) gave the corresponding peaks at 10.26, 10.12, 9.82 and −7.13 ppm and this demonstrates the presence of a strengthened aromatic ring current (Δδ = 17.39 ppm). The proton NMR spectra for **19a** and **19b** again show the presence of *a* plane of symmetry, while there is a duplication of signals for dialkyl oxacarbaporphyrins **19c** and **19d**. As the furan protons are no longer equivalent, they give rise to two doublets. For **19d**, the doublets appeared at 9.57 and 9.51 ppm (*J* = 4.5 Hz); the unsubstituted pyrrole unit also gave rise to a pair of doublets at 9.16 and 9.09 ppm (*J* = 4.2 Hz).

**Figure 8:**
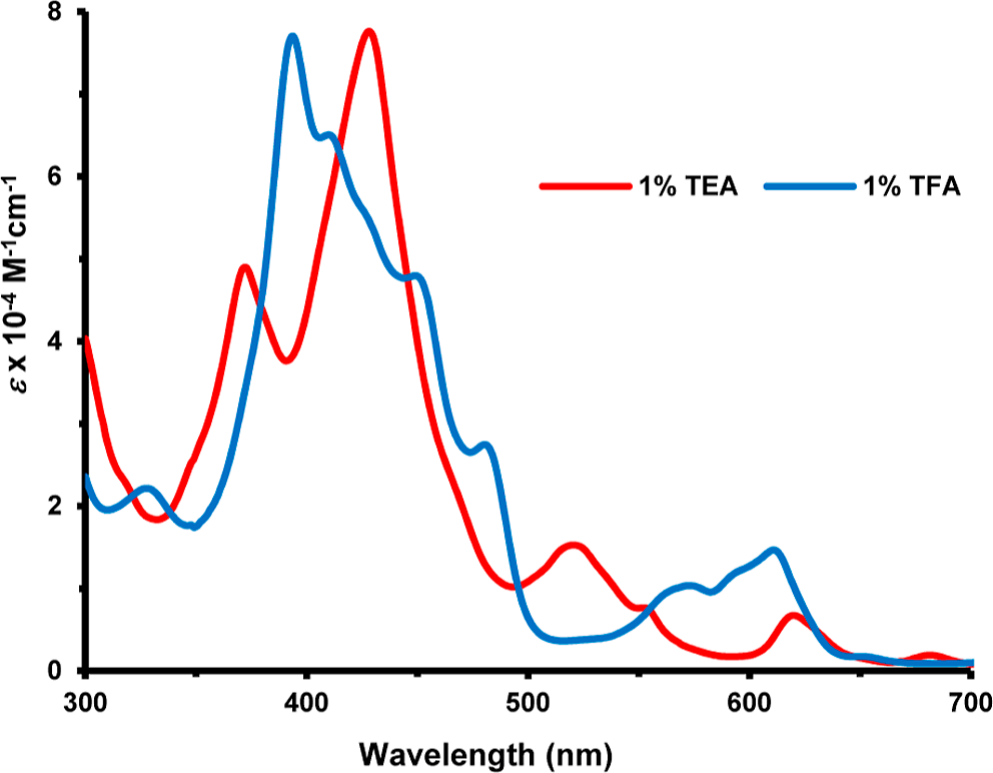
UV–vis spectra of oxacarbaporphyrin **19b** in 1% Et_3_N–CH_2_Cl_2_ and 1% TFA-CH_2_Cl_2_.

Access to alkyl substituted tripyrrins has enabled the synthesis of new examples of carbaporphyrins and oxacarbaporphyrins. In some cases the solubilities were improved but this was not always the case. For instance, tetramethyldiethylcarbaporphyrin **18a** had poor solubility characteristics compared to hexaethylcarbaporphyrin **18b**. Nevertheless, this is a versatile approach that has the potential to provide an entrance to new carbaporphyrin-type structures.

## 3 Conclusions

Four examples of alkyl substituted carbatripyrrins have been synthesized. The formation of these useful intermediates requires that the reactions be carried out under concentrated conditions to ensure precipitation of the required products. Carbatripyrrins have been shown to be versatile precursors to carbaporphyrins and oxacarbaporphyrins and eight examples of new porphyrinoid structures have been prepared and spectroscopically characterized. Proton NMR spectroscopy demonstrates that these structures exhibit strong diatropic ring currents that are enhanced upon protonation. The presence of alkyl substituents exerts minor, but measurable, effects on the electronic absorption spectra and in some cases improved the solubility of the macrocycles. It is difficult to characterize poorly soluble porphyrinoids by NMR spectroscopy and proton NMR spectra for methyl-substituted carbaporphyrins **18a** and **18c** could only be obtained at elevated temperatures (50–55 °C). However, as expected, ethyl-substituted carbaporphyrins **18b** and **18d** exhibited superior solubility characteristics and the results indicate that alkyl substituted carbatripyrrins have promise for further applications in the synthesis of related carbaporphyrinoid systems.

## 4 Experimental Section

Melting points are uncorrected. NMR spectra were recorded using a 400 or 500 MHz NMR spectrometer and were run at 302 K unless otherwise indicated. ^1^H NMR values are reported as chemical shifts δ, relative integral, multiplicity (s, singlet; d, doublet; dd, doublet of doublets, t, triplet; q, quartet; p, pentet; m, multiplet; br, broad peak), and coupling constant (*J*). Chemical shifts are reported in parts per million (ppm) relative to CDCl_3_ (^1^H residual CHCl_3_ singlet δ 7.26 ppm, ^13^C CDCl_3_ triplet δ 77.23 ppm), and coupling constants were taken directly from the spectra. NMR assignments were made with the aid of ^1^H–^1^H COSY, HSQC, DEPT-135, and NOE difference proton NMR spectroscopy. 2D-NMR experiments were performed using standard software. Mass spectral data were acquired using positive-mode electrospray ionization (ESI+) and a high-resolution time-of-flight mass spectrometer.
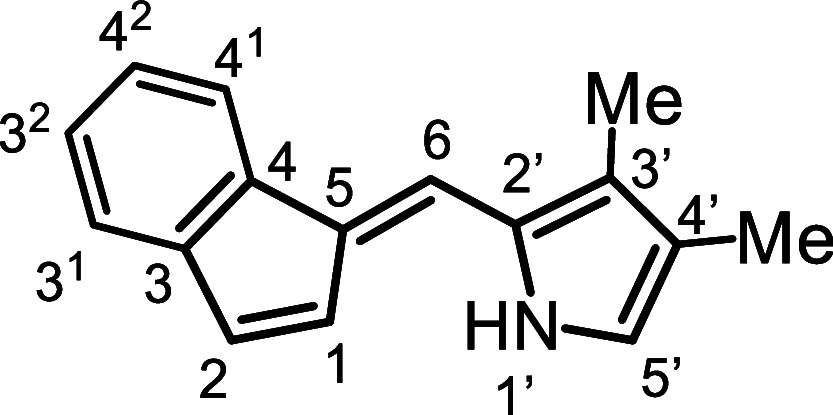


### 4.1 *E*-5­(3,4-Dimethyl-2-pyrrolyl)­benzo­[*c*]­fulvene (**16a**)

Technical grade indene (1.102 g, 90%, 8.55 mmol) and 3,4-dimethylpyrrole-2-carboxaldehyde^30^ (1.076 g, 8.75 mmol) were dissolved in 1% KOH-ethanol (40 mL) and the mixture heated under reflux for 4 days. The solution was diluted with water, extracted with ether (3 × 50 mL), and dried over sodium sulfate. Following suction filtration, the solvent was evaporated and the residue recrystallized from ethyl acetate to give the fulvene (1.241 g, 5.61 mmol, 66%) as orange crystals, mp 172–174 °C. ^1^H NMR (500 MHz, CDCl_3_): δ 8.28 (br s, 1H, NH), 7.72–7.69 (m, 1H, 4^1^-H), 7.38–7.34 (m, 1H, 3^1^-H), 7.33 (br s, 1H, 6-H), 7.23–7.20 (m, 2H, 2^2^,3^2^-H), 7.01 (dd, 1H, *J* = 1.0, 5.4 Hz, 2-H), 6.91 (d, 1H, *J* = 5.4 Hz, 1-H), 6.78 (d, 1H, *J* = 2.4 Hz, 5′-H), 2.21 (s, 3H, 3′-Me), 2.06 (d, 3H, *J* = 0.7 Hz, 4′-Me). {^1^H}^13^C NMR (125 MHz, CDCl_3_): δ 140.9, 138.2, 132.2 (2-CH), 131.5, 127.9, 126.2, 125.8 (3^2^ or 4^2^-CH), 124.8 (3^2^ or 4^2^-CH), 124.7 (1-CH), 121.5 (5′-CH), 121.2 (3^1^-H), 120.8, 118.7 (4^1^-CH), 117.0 (6-CH), 10.3 (4′-Me), 9.6 (3′-Me). HRMS (ESI) *m*/*z*: M^+^ calcd for C_16_H_15_N 221.1204; found, 221.1198.
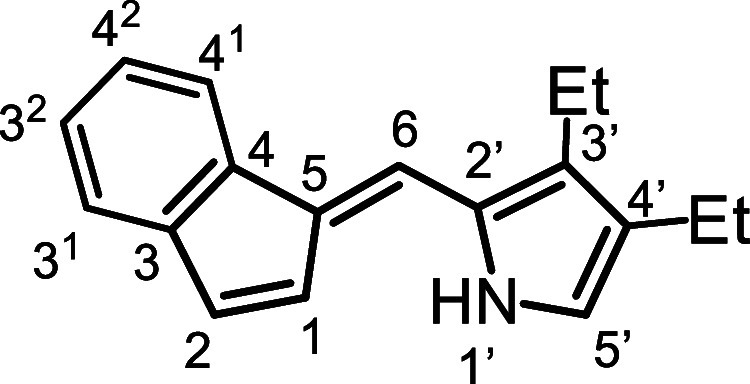


### 4.2 *E*-5­(3,4-Diethyl-2-pyrrolyl)­benzo­[*c*]­fulvene (**16b**)

Technical grade indene (2.204 g, 90%, 17.1 mmol) and 3,4-diethylpyrrole-2-carboxaldehyde^31^ (2.628 g, 17.4 mmol) were dissolved in 1% KOH-ethanol (80 mL) and the mixture heated under reflux for 3 days. The volume of the solution was reduced on a rotary evaporator and then diluted with water, extracted with ether (3 × 50 mL), and dried over sodium sulfate. Following suction filtration, the solvent was evaporated under reduced pressure and the residue recrystallized from hexanes to give the fulvene (3.32 g, 13.3 mmol, 78%) as orange crystals, mp 90–91.5 °C. ^1^H NMR (500 MHz, CDCl_3_): δ 8.32 (br s, 1H, NH), 7.72–7.69 (m, 1H, 4^1^-H), 7.38–7.35 (m, 1H, 3^1^-H), 7.33 (br s, 1H, 6-H), 7.24–7.20 (m, 2H, 2^2^,3^2^-H), 7.01 (dd, 1H, *J* = 1.0, 5.4 Hz, 2-H), 6.93 (d, 1H, *J* = 5.4 Hz, 1-H), 6.79 (d, 1H, *J* = 2.7 Hz, 5′-H), 2.67 (q, 2H, *J* = 7.6 Hz, 3′-CH_2_), 2.51 (dq, 2H, *J* = 0.8, 7.6 Hz, 4′-CH_2_), 1.24 (t, 3H, *J* = 7.6 Hz, 4′-CH_2_C*H*_3_), 1.21 (t, 3H, *J* = 7.6 Hz, 3′-CH_2_C*H*_3_). {^1^H}^13^C NMR (125 MHz, CDCl_3_): δ 140.9, 138.2, 132.2 (2-CH), 131.7, 131.5, 127.3, 127.2, 126.2 (3^2^ or 4^2^-CH), 124.8 (3^2^ or 4^2^-CH and 1-CH), 121.2 (3^1^-H), 120.6 (5′-CH), 118.7 (4^1^-CH), 116.8 (6-CH), 18.4 (4′-CH_2_), 18.0 (3′-CH_2_), 16.7 (3′-CH_2_ *C*H_3_), 14.8 (4′-CH_2_ *C*H_3_). HRMS (ESI) *m*/*z*: [M + H]^+^ calcd for C_18_H_20_N 250.1590; found, 250.1589.
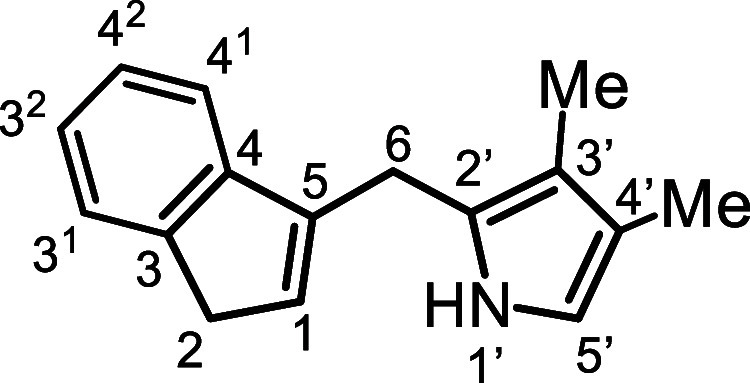


### 4.3 1­(3,4-Dimethyl-2-pyrrolylmethyl)­indene (**17a**)

Fulvene **16a** (1.004 g, 4.54 mmol) was dissolved in THF (50 mL) and LiAlH_4_ (0.23 g) was cautiously added in small portions to avoid foaming. The resulting mixture was stirred under reflux for 16 h. Water (40 mL) was added dropwise and the organic product was extracted with ether (3 × 50 mL) and dried over sodium sulfate. The solvent was removed on a rotary evaporator and the residue purified by column chromatography on silica, eluting with 25% dichloromethane-75% hexanes. The product was collected as a yellow band. Evaporation of the solvent under reduced pressure gave the dihydrofulvene (0.673 g, 3.02 mmol, 66%) as a yellow oil. ^1^H NMR (500 MHz, CDCl_3_): δ 7.50 (br s, 1H, NH), 7.48–7.46 (m, 1H, 4^1^-H), 7.32–7.26 (m, 2H, 3^1^,3^2^-H), 7.22 (dt, 1H, *J* = 1.9, 7.0 Hz, 4^2^-H), 6.40–6.39 (m, 1H, 5′-H), 6.22 (p, 1H, *J* = 1.7 Hz, 1-H), 3.82 (q, 2H, *J* = 1.7 Hz, 2-CH_2_), 3.37 (q, 2H, *J* = 2.0 Hz, bridge-CH_2_), 2.055 (s, 3H, 3′-Me), 2.050 (d, 3H, *J* = 1.0 Hz, 4′-Me). {^1^H}^13^C NMR (125 MHz, CDCl_3_): δ 145.1, 144.7, 142.3, 130.1 (1-CH), 126.4 (3^2^-CH), 125.0 (4^2^-CH), 124.0 (4^1^-CH), 119.4 (3^1^-CH), 118.6, 114.3, 113.4 (5′-H), 37.9 (bridge-CH_2_), 25.2 (2-CH_2_), 10.6, 9.1 (3′,4′-Me). HRMS (ESI) *m*/*z*: [M + H]^+^ calcd for C_16_H_18_N 224.1434; found, 224.1434.
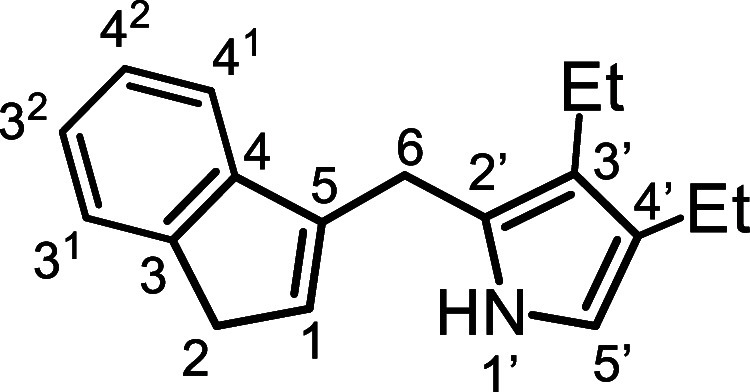


### 4.4 1­(3,4-Diethyl-2-pyrrolylmethyl)­indene (**17b**)

Fulvene **16b** (1.13 g, 4.54 mmol) was dissolved in THF (50 mL) and LiAlH_4_ (0.23 g) was cautiously added in small portions to avoid foaming. The resulting mixture was stirred under reflux for 16 h. Water (40 mL) was added dropwise, and the organic product was extracted with ether (3 × 50 mL) and dried over sodium sulfate. The solvent was removed on a rotary evaporator and the residue purified by column chromatography on silica, eluting with 25% dichloromethane-75% hexanes. The product was collected as a yellow band. Evaporation of the solvent under reduced pressure gave the dihydrofulvene (631.8 mg, 2.52 mmol, 55%) as a yellow oil. ^1^H NMR (500 MHz, CDCl_3_): δ 7.55 (br s, 1H, NH), 7.48–7.45 (m, 1H, 4^1^-H), 7.33–7.21 (m, 1H, 3^1^-H), 7.30–7.27 (m, 1H, 3^2^-H), 7.22 (dt, 1H, *J* = 1.5, 7.2 Hz, 4^2^-H), 6.39–6.38 (m, 1H, 5′-H), 6.22 (p, 1H, *J* = 1.8 Hz, 1-H), 3.85 (q, 2H, *J* = 1.7 Hz, 2-CH_2_), 3.37–3.36 (m, 2H, bridge-CH_2_), 2.52 (q, 2H, *J* = 7.5 Hz, 3′-CH_2_), 2.50 (dq, 3H, *J* = 1.0, 7.5 Hz, 4′-CH_2_), 1.22 (t, 3H, *J* = 7.5 Hz, 3′-CH_2_C*H*_3_), 1.15 (t, 3H, *J* = 7.5 Hz, 4′-CH_2_C*H*_3_). {^1^H}^13^C NMR (125 MHz, CDCl_3_): δ 145.2, 144.7, 142.4, 130.3 (1-CH), 126.4 (3^2^-CH), 125.2, 125.0 (4^2^-CH), 124.6, 124.0 (4^1^-CH), 120.3, 119.4 (3^1^-CH), 112.4 (5′-H), 37.9 (bridge-CH_2_), 25.0 (2-CH_2_), 18.8 (4′-CH_2_), 17.7 (3′-CH_2_), 16.2 (3′-CH_2_ *C*H_3_), 14.8 (4′-CH_2_ *C*H_3_). HRMS (ESI) *m*/*z*: [M + H]^+^ calcd for C_34_H_39_N_3_O_2_ 252.1747; found, 252.1745.
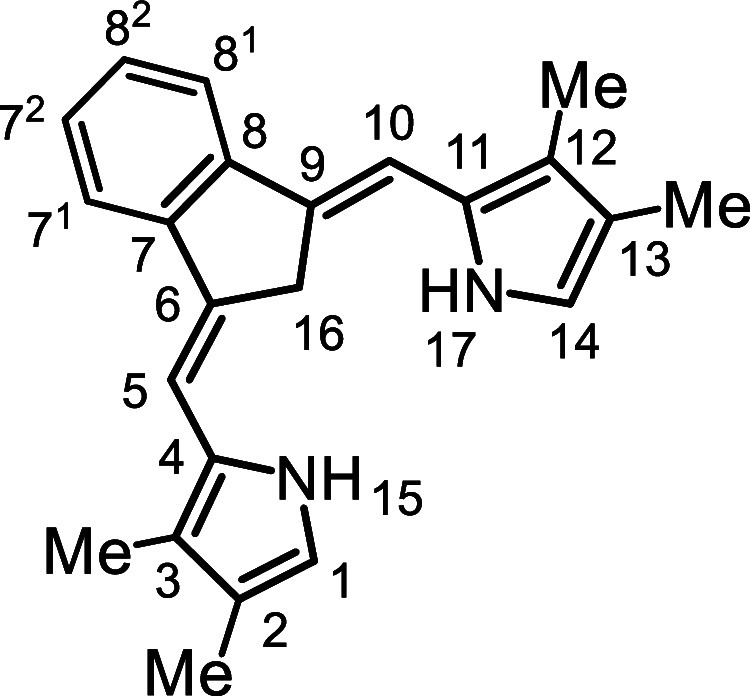


### 4.5 2,3,12,13-Tetramethyl-9-carbabenzo­[*g*]­tripyrrin (**14a**)

A mixture of the crude dihydrofulvene **17a** (622 mg, 2.79 mmol) and 3,4-dimethylpyrrole-2-carbaldehyde^30^ (0.343 g, 2.79 mmol) were taken up in ethanol (4 mL) containing potassium hydroxide (200 mg). The mixture was stirred under reflux for 2 days. The resulting precipitate was collected by suction filtration and washed with 8 mL of cold ethanol. Following vacuum drying, the carbatripyrrin was obtained as a light brown solid (765 mg, 2.33 mmol, 83%), mp 86–87 °C. Prolonged drying in vacuo failed to remove traces of ethanol but due to the instability of this compound it was used without further purification. ^1^H NMR (500 MHz, CDCl_3_): δ 8.09 (br s, 2H, 2 × NH), 7.60–7.57 (m, 2H, 7^1^,8^1^-H), 7.23–7.19 (m, 2H, 7^2^,8^2^-H), 6.90 (t, 2H, *J* = 2.3 Hz, 5,10-H), 6.70 (br d, 2H, *J* = 2.0 Hz, 1,14-H), 3.78 (t, 2H, *J* = 2.2 Hz, 16-CH_2_), 2.13 (s, 6H, 3,12-Me), 2.07 (d, 6H, *J* = 0.9 Hz, 2,13-Me). {^1^H}^13^C NMR (125 MHz, CDCl_3_): δ 143.1, 131.4, 127.79, 127.73 (7^2^,8^2^-CH), 120.0, 119.9 (7^1^,8^1^-CH), 117.3, 108.4 (5,10-CH), 35.7 (16-CH_2_), 10.4 (3,12-Me), 9.5 (2,13-Me). HRMS (ESI) *m*/*z*: M^+^ calcd for C_23_H_24_N_2_ 328.1939; found, 328.1930.
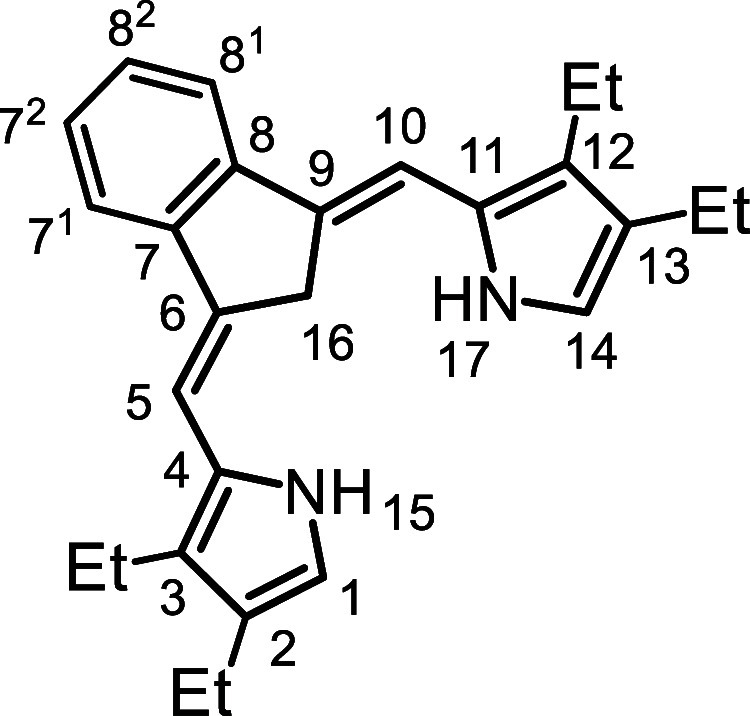


### 4.6 2,3,12,13-Tetraethyl-9-carbabenzo­[*g*]­tripyrrin (**14b**)

A mixture of the crude dihydrofulvene **17b** (620 mg, 2.47 mmol) and 3,4-diethylpyrrole-2-carbaldehyde^31^ (394 mg, 2.61 mmol) were taken up in 20% H_2_O-80% ethanol (6 mL) containing potassium hydroxide (160 mg). The mixture was stirred under reflux for 2 days. The resulting precipitate was collected by suction filtration and washed with 6 mL 20% H_2_O-80% ethanol. Following vacuum drying, the carbatripyrrin was obtained as a brown solid (280 mg, 0.729 mmol, 29%), mp 94–95 °C. Prolonged drying in vacuo failed to remove traces of ethanol but due to the instability of this compound it was used without further purification. ^1^H NMR (500 MHz, CDCl_3_): δ 8.16 (br s, 2H, 2 × NH), 7.61–7.58 (m, 2H, 7^1^,8^1^-H), 7.23–7.20 (m, 2H, 7^2^,8^2^-H), 6.92 (t, 2H, *J* = 2.2 Hz, 5,10-H), 6.71 (br d, 2H, *J* = 2.4 Hz, 1,14-H), 3.83 (t, 2H, *J* = 2.1 Hz, 16-CH_2_), 2.61 (q, 4H, *J* = 7.6 Hz, 3,12-CH_2_), 2.52 (q, 4H, *J* = 7.6 Hz, 2,13-CH_2_), 1.24 (t, 6H, *J* = 7.6 Hz, 2,13-CH_2_C*H*_3_), 1.17 (t, 6H, *J* = 7.6 Hz, 3,12-CH_2_C*H*_3_). {^1^H}^13^C NMR (125 MHz, CDCl_3_): δ 143.1, 131.4, 127.7 (7^2^,8^2^-CH), 127.3, 126.3, 120.0 (7^1^,8^1^-CH), 116.4, 108.3 (5,10-CH), 35.8 (16-CH_2_), 18.5 (2,13-CH_2_), 17.8 (3,12-CH_2_), 16.6 (2,13-CH_2_ *C*H_3_), 14.9 (3,12–4′-CH_2_ *C*H_3_). HRMS (ESI) *m*/*z*: M^+^ calcd for C_27_H_32_N_2_ 384.2565; found, 384.2554.
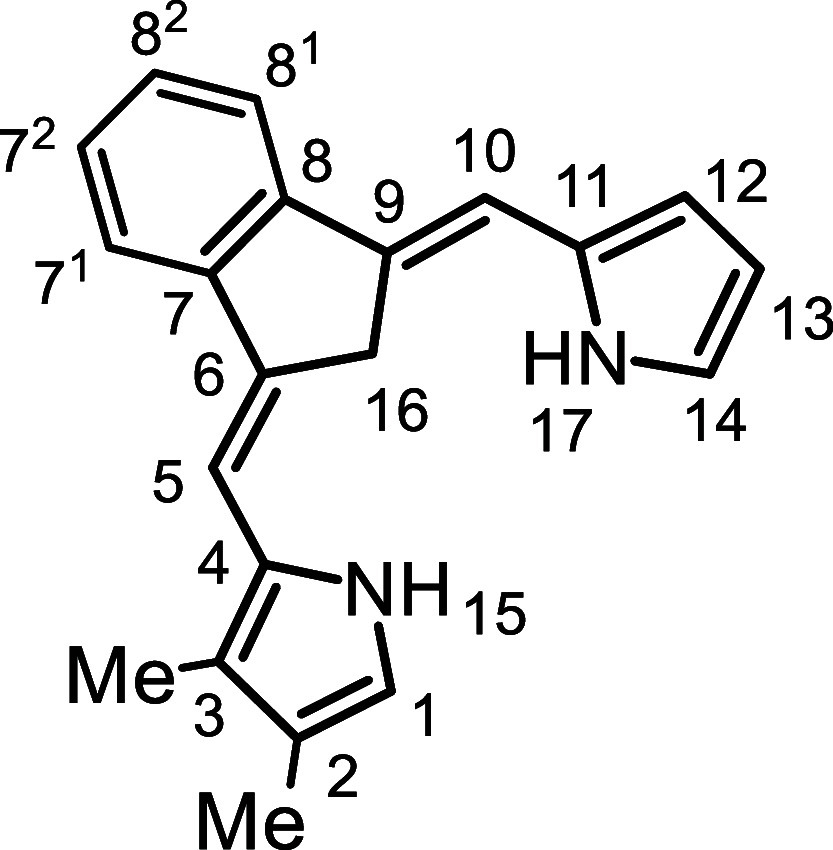


### 4.7 2,3-Dimethyl-9-carbabenzo­[*g*]­tripyrrin (**14c**)

A mixture of dihydrofulvene **9** ^25^ (500 mg, 2.56 mmol) and 3,4-dimethylpyrrole-2-carbaldehyde^30^ (315 mg, 2.56 mmol) were taken up in ethanol (4 mL) containing potassium hydroxide (200 mg). The mixture was stirred under reflux for 2 days. The resulting precipitate was collected by suction filtration and washed with 8 mL of cold ethanol. Following vacuum drying, the carbatripyrrin was obtained as a light brown solid (427 mg, 1.42 mmol, 56%), mp 189–190 °C. ^1^H NMR (500 MHz, CDCl_3_, 55 °C): δ 8.22 (br s, 1H), 8.10 (br s, 1H) (2 × NH), 7.60 (d, 1H, *J* = 7.5 Hz), 7.54 (d, 1H, *J* = 7.5 Hz) (7^1^,8^1^-H), 7.24–7.18 (m, 2H, 7^2^,8^2^-H), 6.93 (t, 1H, *J* = 2.0 Hz, 5 or 10-H), 6.87–6.85 (m, 1H, 14-H), 6.84 (t, 1H, *J* = 2.0 Hz, 5 or 10-H), 6.72 (d, 1H, *J* = 1.7 Hz, 1-H), 3.78 (t, 2H, *J* = 2.1 Hz, 16-CH_2_), 2.15 (s, 3H), 2.08 (s, 3H) (2 × Me). {^1^H}^13^C NMR (125 MHz, CDCl_3_): δ 144.1, 142.5, 135.5, 131.4, 131.3, 128.1, 128.0, 127.6, 120.14, 120.08, 120.0, 119.9, 118.8, 117.6, 110.8, 109.2, 109.0, 108.7, 36.6, 10.3, 9.4. HRMS (ESI) *m*/*z*: [M + H]^+^ calcd for C_21_H_21_N_2_ 301.1699; found, 301.1696.
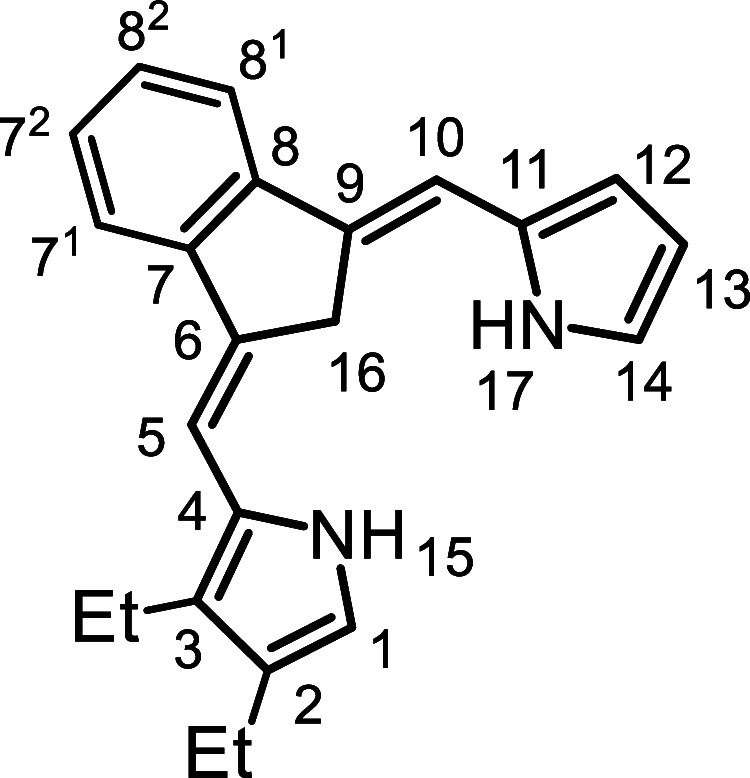


### 4.8 2,3-Diethyl-9-carbabenzo­[*g*]­tripyrrin (**14d**)

A mixture of dihydrofulvene **9** ^25^ (510 mg, 2.61 mmol) and 3,4-diethylpyrrole-2-carbaldehyde^31^ (394 mg, 2.61 mmol) were taken up in 10% water-90% ethanol (4 mL) containing potassium hydroxide (200 mg). The mixture was stirred under reflux for 2 days. The resulting precipitate was collected by suction filtration and washed with 6–7 mL of cold 10% water-90% ethanol. Following vacuum drying, the carbatripyrrin was obtained as a light brown solid (323 mg, 0.985 mmol, 38%), mp 145–147 °C. ^1^H NMR (500 MHz, CDCl_3_, 50 °C): δ 8.22 (br s, 1H), 8.15 (br s, 1H) (2 × NH), 7.61 (d, 1H, *J* = 7.4 Hz), 7.55 (d, 1H, *J* = 7.4 Hz), 7.25–7.18 (m, 2H), 6.95 (t, 1H, *J* = 2.2 Hz, 5- or 10-H), 6.88–6.86 (m, 1H, 14-H), 6.85 (t, 1H, *J* = 2.2 Hz, 5- or 10-H), 6.74 (d, 1H, *J* = 2.5 Hz, 1-H), 6.43–6.42 (m, 1H), 6.38 (q, 1H, 3.0 Hz), 3.80 (t, 2H, *J* = 2.2 Hz), 2.63 (q, 2H, *J* = 7.6 Hz), 2.53 (q, 2H, *J* = 2.5 Hz) (2,3-CH_2_), 1.25 (t, 3H, *J* = 7.5 Hz), 1.19 (t, 3H, *J* = 7.6 Hz) (2,3-CH_2_C*H*_3_). {^1^H}^13^C NMR (125 MHz, CDCl_3_): δ 144.1, 142.4, 135.5, 131.35, 131.29, 128.1, 127.6, 127.5, 126.4, 126.3, 120.1, 120.0, 118.6, 116.7, 110.9, 109.1, 109.0, 108.5, 36.7, 18.6, 17.8, 16.6, 15.0. HRMS (ESI) *m*/*z*: [M + H]^+^ calcd for C_23_H_25_N_2_ 329.2012; found, 329.2021.
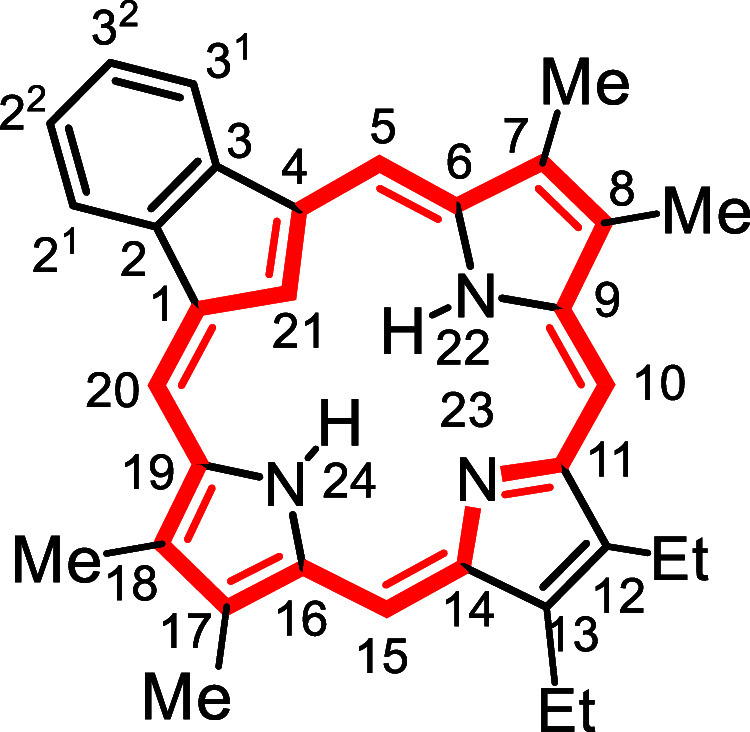


### 4.9 12,13-Diethyl-7,8,17,18-tetramethyl-21-carbabenzo­[*b*]­porphyrin (**18a**)

Carbatripyrrin **14a** (60.3 mg, 0.184 mmol) and 3,4-diethyl-2,5-pyrroledicarbaldehyde^32^ (32.9 mg, 0.184 mmol) were dissolved with stirring in dichloromethane (20 mL). Trifluoroacetic acid (1 mL) was added dropwise to the solution over 1 min, and the mixture was allowed to stir at room temperature open to the air for 30 min. The dark green solution was diluted with dichloromethane (40 mL), and washed with sequentially with water and aqueous sodium bicarbonate solution. The organic solvent was evaporated under reduced pressure and the residue purified by column chromatography on silica gel eluting with dichloromethane. Recrystallization from chloroform–methanol gave the carbaporphyrin (56.5 mg, 0.120 mmol, 65%) as a dark purple solid, mp > 260 °C. UV–vis (1% Et_3_N–CH_2_Cl_2_) λ_max_/nm (log ε): 375 (4.66), 423 (5.24), 508 (4.29), 544 (4.21), 602 (3.75), 662 (3.30). UV–vis (10 equiv TFA-CH_2_Cl_2_): λ_max_/nm (log ε): 393 (4.69), 435 (4.97), 472 (4.46), 546 (4.05), 585 (3.88), 608 (3.86). UV–vis (50% TFA-CH_2_Cl_2_): λ_max_/nm (log ε): 346 (4.48), 426 (5.07), 613 (3.87), 669 (4.25). ^1^H NMR (500 MHz, CDCl_3_, 55 °C): δ 10.10 (s, 2H, 5,20-H), 9.98 (s, 2H, 10,15-H), 8.84–8.81 (m, 2H, 2^1^,3^1^-H), 7.75–7.72 (m, 2H, 2^2^,3^2^-H), 3.98 (q, 4H, *J* = 7.6 Hz, 12,13-CH_2_), 3.63 (s, 6H), 3.59 (s, 6H) (4 × pyrrole-Me), 1.89 (t, 6H, 7.6 Hz, 12,13-CH_2_C*H*_3_), −3.70 (v br, 2H, 2 × NH), −6.60 (s, 1H, 21-H). ^1^H NMR (500 MHz, 2 μL TFA-TFA-CDCl_3_): δ 10.22 (s, 2H, 5,20-H), 9.96 (s, 2H, 10,15-H), 8.66–8.63 (m, 2H, 2^1^,3^1^-H), 7.71–7.68 (m, 2H, 2^2^,3^2^-H), 4.09 (q, 4H, *J* = 7.6 Hz, 12,13-CH_2_), 3.532 (s, 6H), 3.528 (s, 6H) (4 × pyrrole-Me), 1.86 (t, 6H, *J* = 7.6 Hz, 12,13-CH_2_C*H*_3_), −3.11 (s, 2H), −4.39 (br s, 1H) (3 × NH), −6.97 (s, 1H, 21-H). {^1^H}^13^C NMR (125 MHz, 2 μL TFA-CDCl_3_): δ 142.0, 141.3, 141.1, 138.4, 137.5, 136.7, 135.5, 134.7, 128.1 (2^2^,3^2^-CH), 121.5 (2^1^,3^1^-H), 119.6 (21-CH), 104.4 (5,20-CH), 94.3 (10,15-CH), 19.9, 17.8, 12.0, 11.9. HRMS (ESI) *m*/*z*: [M + H]^+^ calcd for C_33_H_34_N_3_ 472.2747; found, 472.2739.
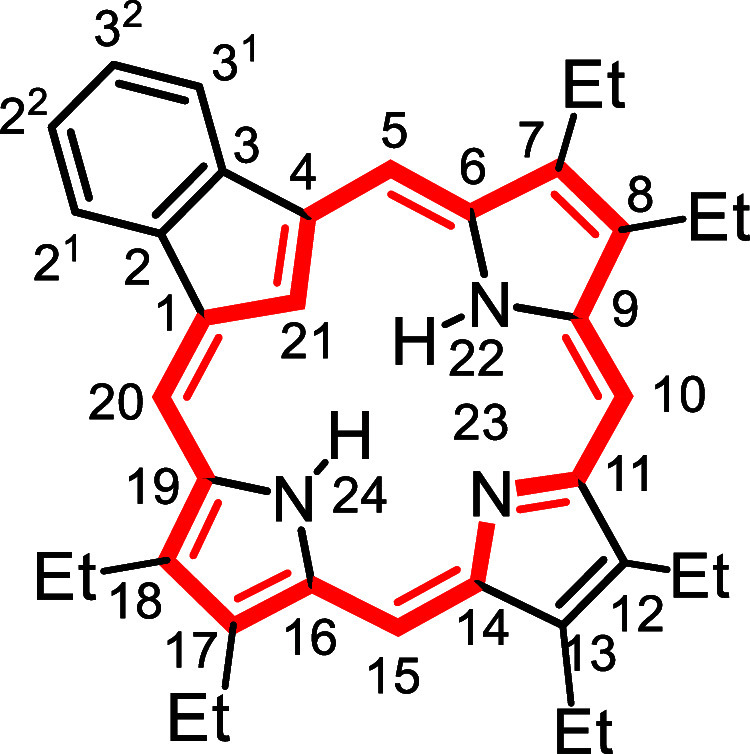


### 4.10 7,8,12,13,17,18-Hexaethyl-21-carbabenzo­[*b*]­porphyrin (**18b**)

Trifluoroacetic acid (0.7 mL) was added dropwise to a stirred solution of carbatripyrrin **14b** (50.0 mg, 0.130 mmol) and 3,4-diethyl-2,5-pyrroledicarbaldehyde^32^ (23.3 mg, 0.130 mmol) in dichloromethane (14 mL) over a period of 1 min, and the mixture was allowed to stir at room temperature open to the air for 30 min. The mixture was diluted with dichloromethane (30 mL) and washed sequentially with water and aqueous sodium bicarbonate solution. The organic solvent was evaporated under reduced pressure and the residue purified by column chromatography on silica gel eluting with dichloromethane. Recrystallization from chloroform–methanol gave the carbaporphyrin (38.5 mg, 0.073 mmol, 56%) as dark purple crystals, mp 228–230 °C. UV–vis (CH_2_Cl_2_) λ_max_/nm (log ε): 375 (4.70), 424 (5.32), 510 (4.29), 544 (4.22), 602 (3.70), 662 (3.28). UV–vis (10 equiv TFA-CH_2_Cl_2_): λ_max_/nm (log ε): 394 (4.82), 436 (5.11), 472 (4.63), 547 (4.12), 608 (3.94). UV–vis (50% TFA-CH_2_Cl_2_): λ_max_/nm (log ε): 346 (4.62), 426 (5.30), 616 (3.95), 671 (4.47). ^1^H NMR (500 MHz, CDCl_3_): δ 10.14 (s, 2H, 5,20-H), 9.84 (s, 2H, 10,15-H), 8.87–8.84 (m, 2H, 2^1^,3^1^-H), 7.77–7.74 (m, 2H, 2^2^,3^2^-H), 4.15–4.07 (m, 8H, 7,8,17,18-CH_2_), 3.98 (q, 4H, *J* = 7.6 Hz, 12,13-CH_2_), 1.94–1.86 (m, 18H, 6 × CH_2_C*H*_3_), −3.87 (v br, 2H, 2 × NH), −6.65 (s, 1H, 21-H). ^1^H NMR (500 MHz, 2 μL TFA-TFA-CDCl_3_): δ 10.32 (s, 2H, 5,20-H), 10.05 (s, 2H, 10,15-H), 8.72–8.69 (m, 2H, 2^1^,3^1^-H), 7.74–7.70 (m, 2H, 2^2^,3^2^-H), 4.13–4.03 (m, 12H, 6 × C*H*_2_CH_3_), 1.85 (t, 6H, *J* = 7.7 Hz), 1.77–1.73 (2 overlapping triplets, 12H) (6 × CH_2_C*H*_3_), −2.83 (s, 2H), −4.05 (br s, 1H) (3 × NH), −6.71 (s, 1H, 21-H). {^1^H}^13^C NMR (125 MHz, CDCl_3_): δ 152.8, 144.4, 141.5, 139.0, 137.2, 135.6, 134.7, 133.9, 126.6 (2^2^,3^2^-CH), 120.6 (2^1^,3^1^-CH), 109.5 (21-CH), 98.8 (5,20-CH), 95.6 (10,15-CH), 20.1, 19.9, 19.7, 18.7, 18.5, 18.4. HRMS (ESI) *m*/*z*: [M + H]^+^ calcd for C_37_H_42_N_3_ 528.3373, found 528.3365.
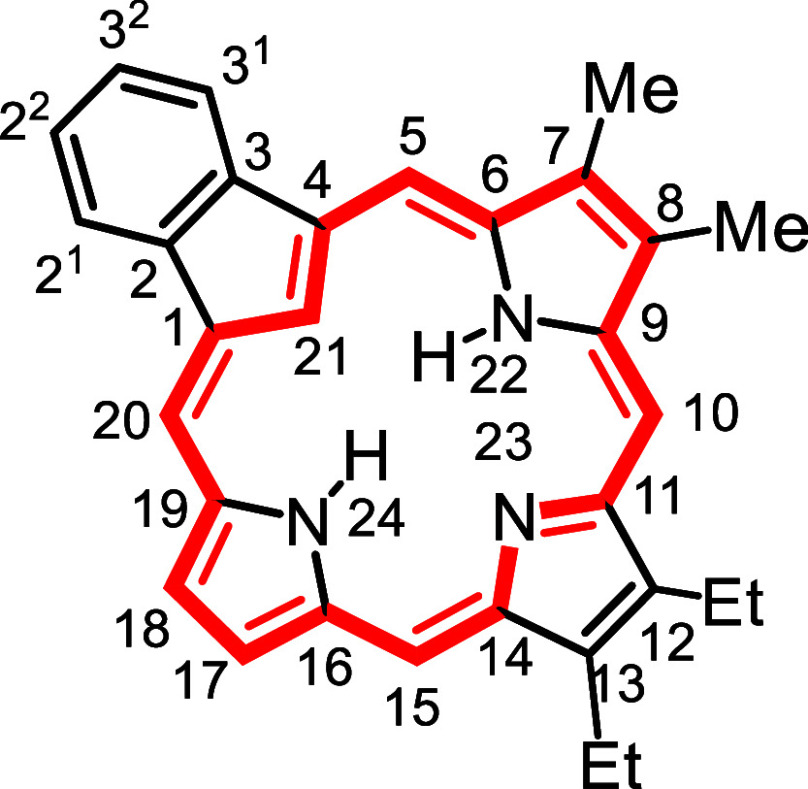


### 4.11 12,13-Diethyl-7,8-dimethyl-21-carbabenzo­[*b*]­porphyrin (**18c**)

Carbatripyrrin **14c** (54.1 mg, 0.180 mmol) and 3,4-diethyl-2,5-pyrroledicarbaldehyde^32^ (32.9 mg, 0.184 mmol) were dissolved with stirring in dichloromethane (20 mL). Trifluoroacetic acid (1 mL) was added dropwise to the solution over 1 min, and the mixture was allowed to stir at room temperature open to the air for 30 min. The dark green solution was diluted with dichloromethane (40 mL) and washed sequentially with water and aqueous sodium bicarbonate solution. The organic solvent was evaporated under reduced pressure and the residue purified by column chromatography on silica gel eluting with dichloromethane. Recrystallization from chloroform–methanol gave the carbaporphyrin (45.2 mg, 0.102 mmol, 56%) as dark purple crystals, mp > 260 °C. UV–vis (CH_2_Cl_2_) λ_max_/nm (log ε): 373 (4.68), 422 (5.21), 504 (4.27), 537 (3.97), 602 (3.62), 661 (3.25). UV–vis (20 equiv TFA-CH_2_Cl_2_): λ_max_/nm (log ε): 395 (4.81), 434 (5.02), 469 (4.48), 543 (4.07), 608 (3.85). UV–vis (50% TFA-CH_2_Cl_2_): λ_max_/nm (log ε): 346 (4.55), 423 (5.17), 615 (3.98), 670 (4.35). ^1^H NMR (500 MHz, CDCl_3_, 50 °C): δ 10.07 (s, 1H, 20-H), 9.82 (s, 1H, 5-H), 9.79 (s, 1H, 15-H), 9.55 (s, 1H, 10-H), 9.23 (d, 1H, *J* = 4.4 Hz, 18-H), 9.15 (d, 1H, *J* = 4.4 Hz, 17-H), 8.76–8.70 (m, 2H, 2^1^,3^1^-H), 7.73–7.70 (m, 2H, 2^2^,3^2^-H), 3.93–3.86 (2 overlapping quartets, 4H, 12,13-CH_2_), 3.48 (s, 3H, 7-Me), 3.45 (s, 3H, 8-Me), 1.87–1.83 (2 overlapping triplets, 6H, 2 × CH_2_C*H*_3_), −3.93 (br, 2H, 2 × NH), −6.93 (s, 1H, 21-H). {^1^H}^13^C NMR (125 MHz, CDCl_3_, 50 °C): δ 154.1, 152.7, 145.0, 144.3, 141.7, 141.4, 137.5, 136.7, 136.3, 136.0, 134.5, 134.4, 133.8, 131.8, 126.9, 126.8 (2^2^,3^2^-CH), 126.0 (18-CH), 123.8 (17-CH), 120.72, 120.65 (2^1^,3^1^-CH), 110.3 (21-CH), 103.1 (20-CH), 99.8 (5-CH), 98.1 (15-CH), 94.6 (10-CH), 20.05, 20.03, 18.50, 18.46, 11.6, 11.3. HRMS (ESI) *m*/*z*: [M + H]^+^ calcd for C_31_H_30_N_3_ 444.2434; found, 444.2425.
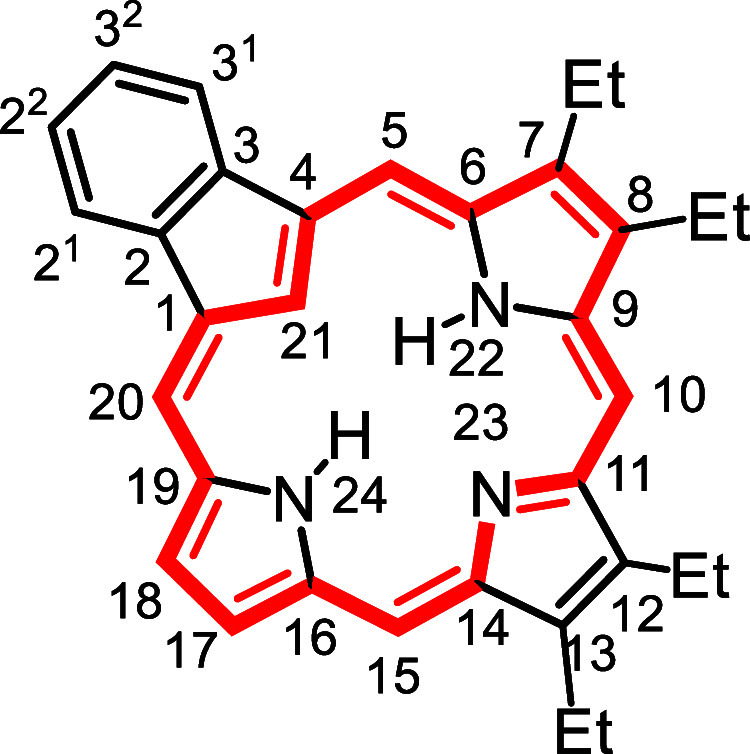


### 4.12 7,8,12,13-Tetraethyl-21-carbabenzo­[*b*]­porphyrin (**18d**)

Carbatripyrrin **14d** (60.3 mg, 0.184 mmol) and 3,4-diethyl-2,5-pyrroledicarbaldehyde^32^ (32.9 mg, 0.184 mmol) were dissolved with stirring in dichloromethane (20 mL). Trifluoroacetic acid (1 mL) was added dropwise to the solution over 1 min, and the mixture was allowed to stir at room temperature open to the air for 30 min. The dark green solution was diluted with dichloromethane (40 mL) and washed sequentially with water and aqueous sodium bicarbonate solution. The organic solvent was evaporated under reduced pressure and the residue purified by column chromatography on silica gel eluting with dichloromethane. Recrystallization from chloroform–methanol gave the carbaporphyrin (30.8 mg, 0.0654 mmol, 35%) as dark purple crystals, mp > 260 °C. UV–vis (1% Et_3_N–CH_2_Cl_2_) λ_max_/nm (log ε): 375 (4.72), 423 (5.11), 508 (4.11), 544 (4.09), 603 (3.53), 662 (2.88). UV–vis (5 equiv TFA-CH_2_Cl_2_): λ_max_/nm (log ε): 392 (4.63), 435 (4.91), 472 (4.42), 546 (3.94), 586 (3.79), 608 (3.75). UV–vis (50% TFA-CH_2_Cl_2_): λ_max_/nm (log ε): 346 (4.57), 426 (5.17), 614 (3.92), 669 (4.36). ^1^H NMR (500 MHz, CDCl_3_): δ 10.10 (s, 1H, 20-H), 10.00 (s, 1H, 5-H), 9.81 (s, 1H, 15-H), 9.71 (s, 1H, 10-H), 9.25 (d, 1H, *J* = 4.4 Hz, 18-H), 9.16 (d, 1H, *J* = 4.4 Hz, 17-H), 8.80–8.75 (m, 2H, 2^1^,3^1^-H), 7.77–7.71 (m, 2H, 2^2^,3^2^-H), 4.09–4.01 (m, 4H, 7,8-CH_2_), 3.94–3.88 (m, 4H, 12,13-CH_2_), 1.91–1.83 (m, 12H, 4 × CH_2_C*H*_3_), −3.90 (v br, 2H, 2 × NH), −6.84 (s, 1H, 21-H). {^1^H}^13^C NMR (125 MHz, CDCl_3_, 50 °C): δ 153.0, 152.7, 145.0, 144.3, 141.6, 141.3, 139.6, 137.7, 136.60, 136.53, 135.8, 135.4, 134.4, 134.3, 126.80, 126.76 (2^2^,3^2^-CH), 126.0 (18-CH), 123.8 (17-CH), 120.71, 120.64 (2^1^,3^1^-CH), 110.2 (21-CH), 103.0 (20-CH), 99.6 (15-CH), 98.3 (5-CH), 94.9 (10-CH), 20.1, 20.0, 19.8, 19.6, 18.63, 18.57, 18.4, 18.3. HRMS (ESI) *m*/*z*: [M + H]^+^ calcd for C_35_H_34_N_3_ 472.2747; found, 472.2735.
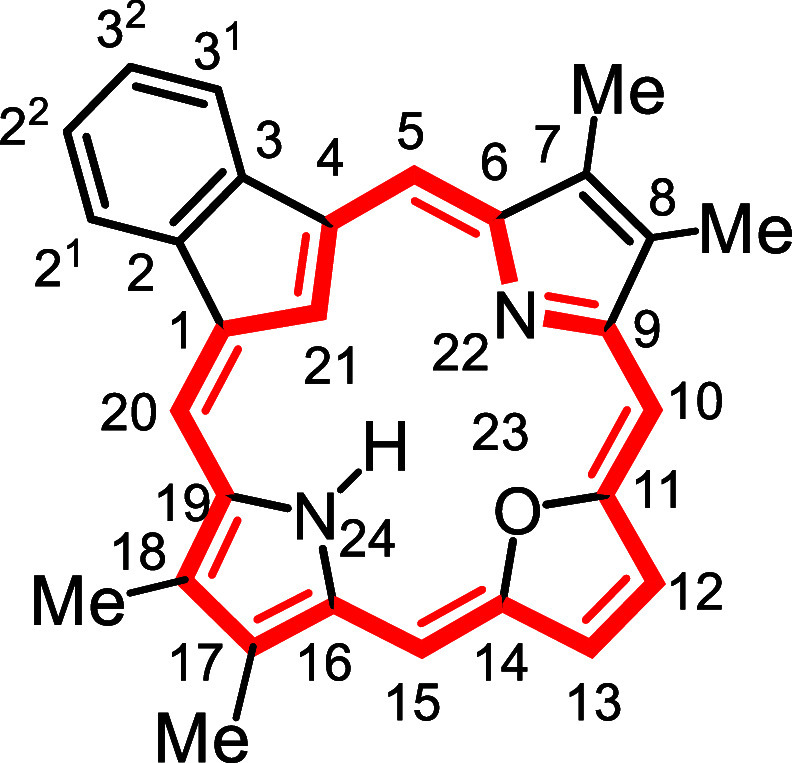


### 4.13 7,8,17,18-Tetramethyl-21-carba-23-oxabenzo­[*b*]­porphyrin (**19a**)

TFA (1 mL) was added dropwise over 1 min to a stirred solution of 2,5-furandicarboxaldehyde (22.3 mg, 0.180 mmol) and carbatripyrrin **14a** (60.3 mg, 0.184 mmol) in dichloromethane (20 mL), and stirring was continued at room temperature for 20 min. The resulting mixture was diluted with dichloromethane (40 mL) and washed sequentially with water and 5% aqueous sodium bicarbonate solution. The organic solution was dried over sodium sulfate and evaporated to dryness. The residue was loaded onto a grade 3 basic alumina column, eluting with 2–10% ethyl acetate-toluene. The solvent was evaporated under reduced pressure and the residue recrystallized from chloroform-hexanes to give the oxacarbaporphyrin (32.7 mg, 0.0786 mmol, 43%) as a dark solid, mp > 260 °C. UV–vis (1% Et_3_N–CH_2_Cl_2_) λ_max_/nm (log ε): 372 (4.71), 424 (4.92), 521 (4.20), 552 (sh, 3.90), 620 (3.84), 682 (3.33). UV–vis (3 equiv TFA-CH_2_Cl_2_): λ_max_/nm (log ε): 391 (4.85), 417 (4.81), 436 (4.79), 481 (4.52), 573 (4.01), 607 (4.09), 664 (3.32). ^1^H NMR (500 MHz, CDCl_3_): δ 10.11 (s, 2H, 5,20-H), 9.85 (s, 2H, 10,15-H), 9.58 (12,13-H), 8.77–8.74 (m, 2H, 2^1^,3^1^-H), 7.70–7.69 (m, 2H, 2^2^,3^2^-H), 3.51 (s, 6H), 3.47 (s, 6H) (4 × pyrrole-Me), −4.89 (s, 1H, 21-H). {^1^H}^13^C NMR (125 MHz, 2 μL TFA-CDCl_3_): δ 152.9, 141.4, 136.9, 128.74, 128.64 (2^2^,3^2^-CH), 128.4, 121.7 (2^1^,3^1^-H), 121.5 (21-CH), 105.5 (5,20-CH), 94.8 (10,15-CH), 12.1, 11.8. HRMS (ESI) *m*/*z*: [M + H]^+^ calcd for C_29_H_25_N_2_O 417.1961; found, 417.1961.
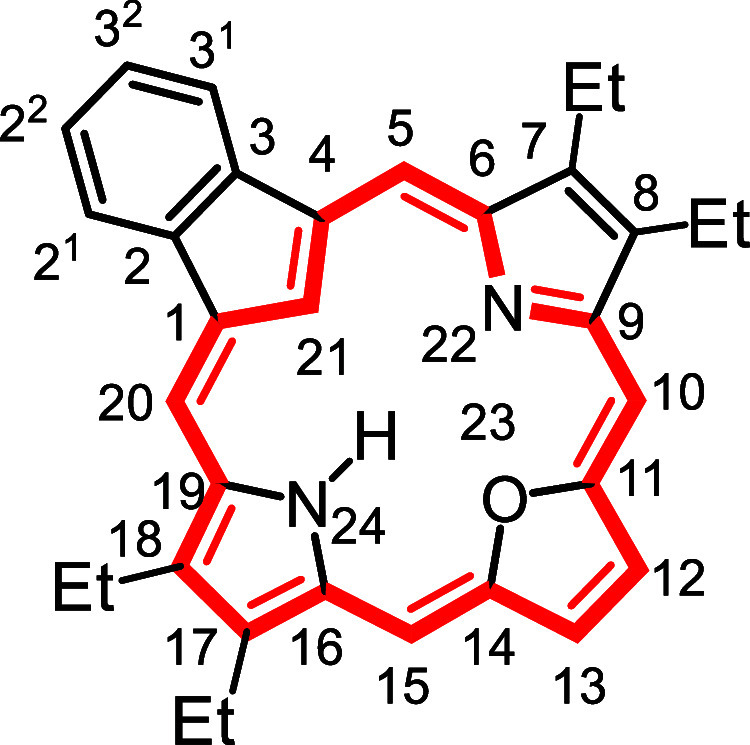


### 4.14 7,8,17,18-Tetraethyl-21-carba-23-oxabenzo­[*b*]­porphyrin (**19b**)

TFA (0.7 mL) was added dropwise over 1 min to a stirred solution of 2,5-furandicarboxaldehyde (16.5 mg, 0.133 mmol) and carbatripyrrin **14b** (50.0 mg, 0.130 mmol) in dichloromethane (14 mL), and stirring was continued at room temperature for 20 min. The resulting mixture was diluted with dichloromethane (40 mL) and washed sequentially with water and 5% aqueous sodium bicarbonate solution. The organic solution was dried over sodium sulfate and evaporated to dryness. The residue was loaded onto a grade 3 basic alumina column, eluting with 2–5% ethyl acetate-toluene. The solvent was evaporated under reduced pressure and the residue recrystallized from chloroform-hexanes to give the oxacarbaporphyrin (32.8 mg, 0.0695 mmol, 53%) as dark purple crystals, mp 238–240 °C. UV–vis (1% Et_3_N–CH_2_Cl_2_) λ_max_/nm (log ε): 372 (4.69), 428 (4.89), 521 (4.18), 553 (sh, 3.88), 620 (3.83), 681 (3.28). UV–vis (3 equiv TFA-CH_2_Cl_2_): λ_max_/nm (log ε): 392 (4.83), 416 (4.76), 436 (sh, 4.74), 482 (4.53), 576 (sh, 3.99), 610 (4.07), 662 (3.29). ^1^H NMR (500 MHz, 2 μL TFA-CDCl_3_): δ 10.42 (s, 2H, 5,20-H), 10.30 (s, 2H, 10,15-H), 10.00 (12,13-H), 8.71–8.68 (m, 2H, 2^1^,3^1^-H), 7.78–7.75 (m, 2H, 2^2^,3^2^-H), 4.16–4.09 (m, 8H, 2 × pyrrole-CH_2_), 1.86 (t, 6H, *J* = 7.8 Hz, 2 × CH_2_C*H*_3_), −4.71 (s, 2H, 2 × NH), −6.97 (s, 1H, 21-H). {^1^H}^13^C NMR (125 MHz, 2 μL TFA-CDCl_3_): δ 153.2, 142.8, 141.5, 141.1, 140.16, 140.09, 135.8, 129.3, 129.0 (2^2^,3^2^-H), 122.0 (2^1^,3^1^-H), 119.3 (21-CH), 105.3 (5,20-CH), 95.0 (10,15-CH), 20.0, 19.8, 18.2, 17.8. HRMS (ESI) *m*/*z*: [M + H]^+^ calcd for C_33_H_33_N_2_O 473.2587; found, 473.2578.
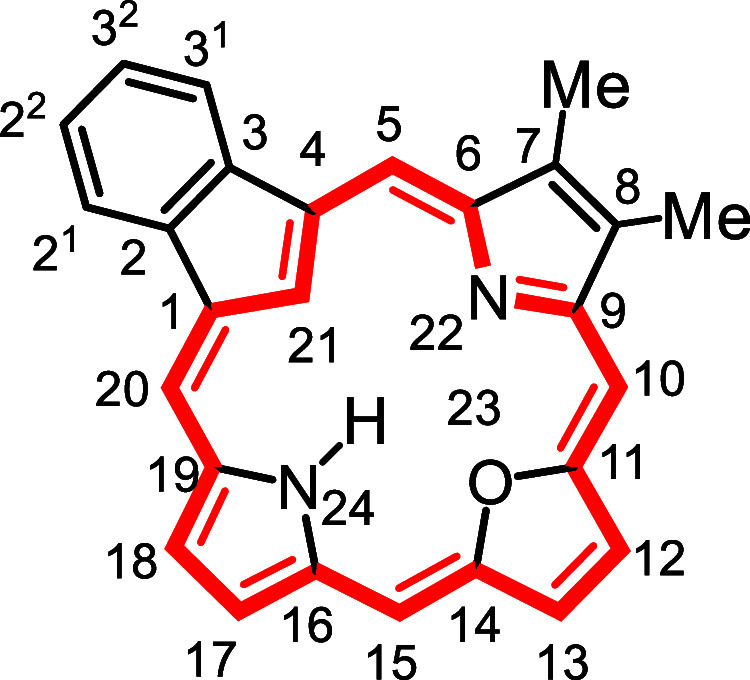


### 4.15 7,8-Dimethyl-21-carba-23-oxabenzo­[*b*]­porphyrin (**19c**)

TFA (1 mL) was added dropwise over 1 min to a stirred solution of 2,5-furandicarboxaldehyde (22.3 mg, 0.183 mmol) and carbatripyrrin **14c** (55.1 mg, 0.184 mmol) in dichloromethane (20 mL), and stirring was continued at room temperature for 20 min. The resulting mixture was diluted with dichloromethane (40 mL) and washed sequentially with water and 5% aqueous sodium bicarbonate solution. The organic solution was dried over sodium sulfate and evaporated to dryness. The residue was loaded onto a grade 3 basic alumina column, eluting with 2–15% ethyl acetate-toluene. The solvent was evaporated under reduced pressure and the residue recrystallized from chloroform-hexanes to give the oxacarbaporphyrin (31.5 mg, 0.0812 mmol, 44%) as a dark solid, mp > 260 °C. λ_max_/nm (log ε): 370 (4.59), 425 (4.91), 519 (4.12), 554 (sh, 3.81), 617 (3.82), 681 (3.21). UV–vis (5 equiv TFA-CH_2_Cl_2_): λ_max_/nm (log ε): 393 (4.86), 420 (sh, 4.73), 438 (sh, 4.69), 480 (4.45), 573 (sh, 3.93), 604 (3.98), 659 (3.19). ^1^H NMR (500 MHz, CDCl_3_): δ 10.14 (s, 1H), 9.98 (s, 1H), 9.91 (s, 1H), 9.66 (s, 1H), 9.59 (d, 1H, *J* = 4.2 Hz), 9.49 (d, 1H, *J* = 4.2 Hz), 9.15 (d, 1H, *J* = 4.1 Hz), 9.10 (d, 1H, *J* = 4.1 Hz), 8.71–8.68 (m, 2H, 2^1^,3^1^-H), 7.69–7.67 (m, 2H, 2^2^,3^2^-H), 3.47 (s, 3H), 3.42 (s, 3H) (2 × pyrrole-Me), −5.40 (s, 1H, 21-H). {^1^H}^13^C NMR (125 MHz, 2 μL TFA-CDCl_3_): δ 153.9, 152.5, 141.5, 141.4, 141.2, 140.0, 139.7, 138.2, 137.8, 136.6, 135.7, 129.8, 129.3 (12,13-H), 129.0, 128.9 (2^2^,3^2^-H), 128.5, 127.4 (17,18-H), 122.4 (21-CH), 121.9 (2^1^,3^1^-H), 109.6, 106.5, 98.2, 94.6, 12.1, 11.8. HRMS (ESI) *m*/*z*: [M + H]^+^ calcd for C_27_H_21_N_2_O 389.1648; found, 389.1641.
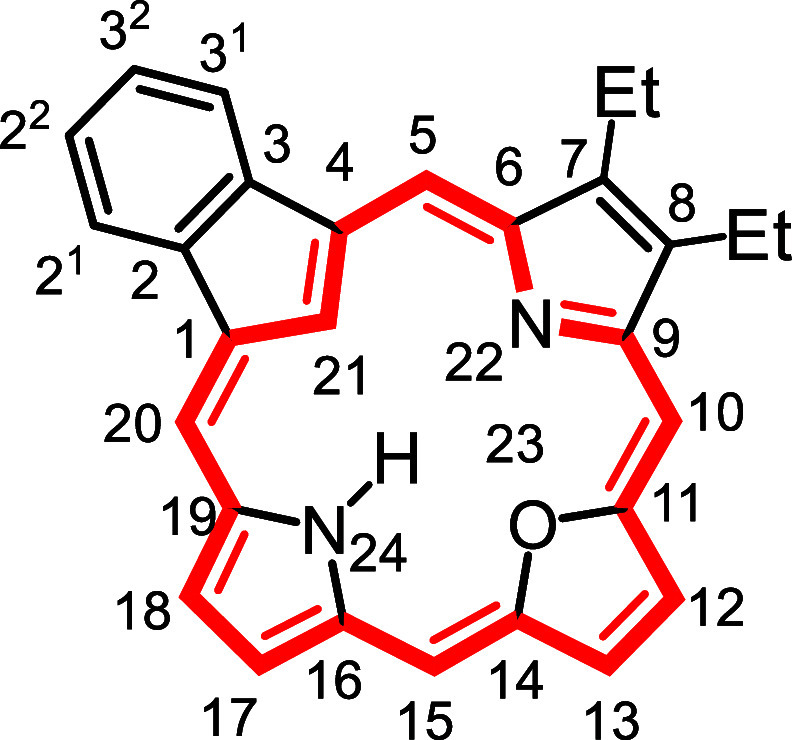


### 4.16 7,8-Diethyl-21-carba-23-oxabenzo­[*b*]­porphyrin (**19d**)

TFA (1 mL) was added dropwise over 1 min to a stirred solution of 2,5-furandicarboxaldehyde (22.3 mg, 0.180 mmol) and carbatripyrrin **14d** (60.3 mg, 0.184 mmol) in dichloromethane (20 mL), and stirring was continued at room temperature for 20 min. The resulting mixture was diluted with dichloromethane (40 mL) and washed sequentially with water and 5% aqueous sodium bicarbonate solution. The organic solution was dried over sodium sulfate and evaporated to dryness. The residue was loaded onto a grade 3 basic alumina column, eluting with 2–15% ethyl acetate-toluene. The solvent evaporated under reduced pressure and the residue recrystallized from chloroform-hexanes to give the oxacarbaporphyrin (22.1 mg, 0.0531 mmol, 29%) as dark purple crystals, mp > 300 °C. UV–vis (1% Et_3_N–CH_2_Cl_2_) λ_max_/nm (log ε): 370 (4.60), 425 (4.92), 520 (4.13), 554 (sh, 3.80), 618 (3.84), 680 (3.00). UV–vis (3 equiv TFA-CH_2_Cl_2_): λ_max_/nm (log ε): 394 (4.88), 420 (sh, 4.73), 438 (sh, 4.47), 480 (4.47), 572 (3.93), 606 (4.00), 662 (sh, 3.13). ^1^H NMR (500 MHz, CDCl_3_, 50 °C): δ 10.17 (s, 1H), 10.12 (s, 1H), 9.89 (s, 1H), 9.77 (s, 1H), 9.57 (d, 1H, *J* = 4.5 Hz), 9.51 (d, 1H, *J* = 4.5 Hz), 9.16 (d, 1H, *J* = 4.1 Hz), 9.09 (d, 1H, *J* = 4.1 Hz), 8.77–8.72 (m, 2H, 2^1^,3^1^-H), 7.71–7.68 (m, 2H, 2^2^,3^2^-H), 4.07 (q, 2H, *J* = 7.7 Hz), 3.97 (q, 2H, *J* = 7.7 Hz) (7,8-CH_2_), 1.90 (t, 3H, *J* = 7.7 Hz), 1.87 (t, 3H, *J* = 7.7 Hz) (2 × CH_2_C*H*_3_), −4.78 (s, 1H, 21-H). ^1^H NMR (500 MHz, 2 μL TFA-CDCl_3_): δ 9.99 (s, 1H), 9.86 (s, 1H), 9.82 (s, 1H), 9.72 (s, 1H), 9.65 (d, 1H, *J* = 4.5 Hz), 9.62 (d, 1H, *J* = 4.5 Hz), 9.01 (d, 1H, *J* = 4.0 Hz), 8.95 (d, 1H, *J* = 4.0 Hz), 8.47–8.45 (m, 1H), 8.41–8.39 (m, 1H) (2^1^,3^1^-H), 7.70–7.66 (m, 2H, 2^2^,3^2^-H), 3.98–3.92 (m, 4H, 7,8-CH_2_), 1.80–1.76 (m, 6H, 2 × CH_2_C*H*_3_), −5.08 (s, 1H), −5.18 (s, 1H) (2 × NH), −6.23 (s, 1H, 21-H). {^1^H}^13^C NMR (125 MHz, 2 μL TFA-CDCl_3_): δ 153.1, 151.9, 142.9, 141.1, 141.0, 140.8, 140.5, 140.0, 139.2, 139.0, 136.7, 135.9, 129.3, 128.8 (12,13-H), 128.1 (2^2^,3^2^-CH), 127.0 (17,18-CH), 121.75, 121.68 (2^1^,3^1^-CH), 121.2 (21-CH), 108.9, 105.7, 97.4, 93.9, 19.9, 19.6, 18.2, 17.8. HRMS (ESI) *m*/*z*: [M + H]^+^ calcd for C_29_H_25_N_2_O 417.1961; found, 417.1952.

## Supplementary Material



## Data Availability

The data underlying this study are available in the published article and its online Supporting Information.

## Notes

Selected UV–vis (Figures S1–S44), ^1^H NMR, ^1^H–^1^H COSY, HSQC, DEPT-135, ^13^C­{^1^H} NMR (Figures S45–S116) and mass spectra (Figures S117–S132), are provided (PDF)
