# Supplementary material for: Synthesis of Alkyl Substituted Carbatripyrrins and Their Application to the Preparation of Carbaporphyrins and Oxacarbaporphyrins
Source: ACS Omega. 2025 Oct 27;10(43):51644–56. doi: 10.1021/acsomega.5c07422 (PMC12593086; doi:10.1021/acsomega.5c07422)
Supplement: Supplementary file 1 [file ao5c07422_si_001.pdf]

Supporting information for

Synthesis of Alkyl Substituted Carbatripyrrins and Their Application to the Preparation of Carbaporphyrins and Oxacarbaporphyrins

Ian A. McLauchlan,[1] Bethany K. X. Overbey,[1] Tyler J. Smolczyk,[1] John J. Woods[1] and Timothy D. Lash[1]*

[1]Department of Chemistry, Illinois State University, Normal, Illinois 61790-4160

Table of Contents

Page

S2-S23 Selected UV-Vis spectra (Figures S1-S44)

S24-S77 Selected proton, DEPT-135, ^{1}H-^{1}H COSY, HSQC and carbon-13 NMR spectra (Figures S45-S116)

S78-S85 Selected mass spectra (Figures S117-S132)

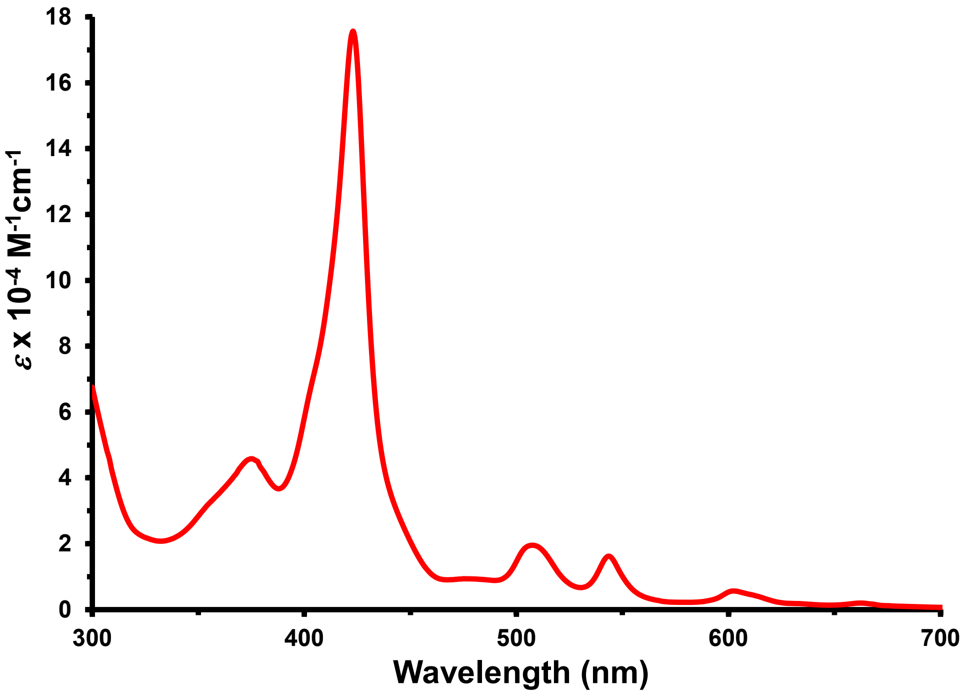

Figure S1. UV-vis spectrum of tetramethylcarbaporphyrin **18a** in 1% Et_3N-CH_2Cl_2.

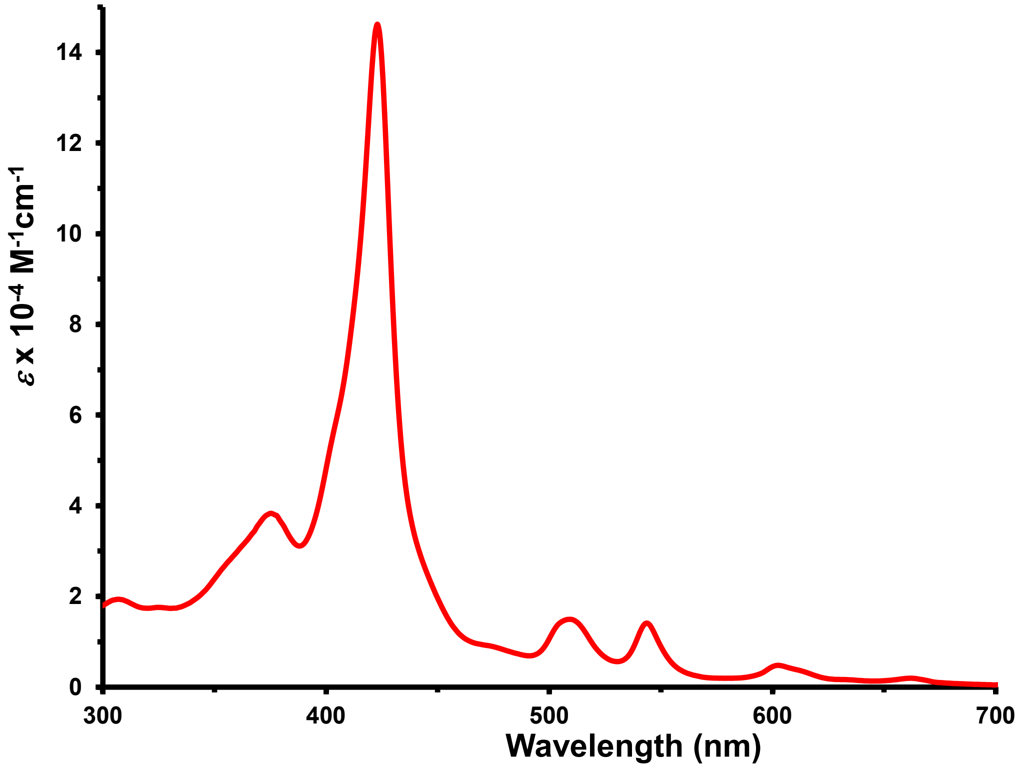

Figure S2. UV-vis spectrum of tetramethylcarbaporphyrin **18a** in CH_2Cl_2.

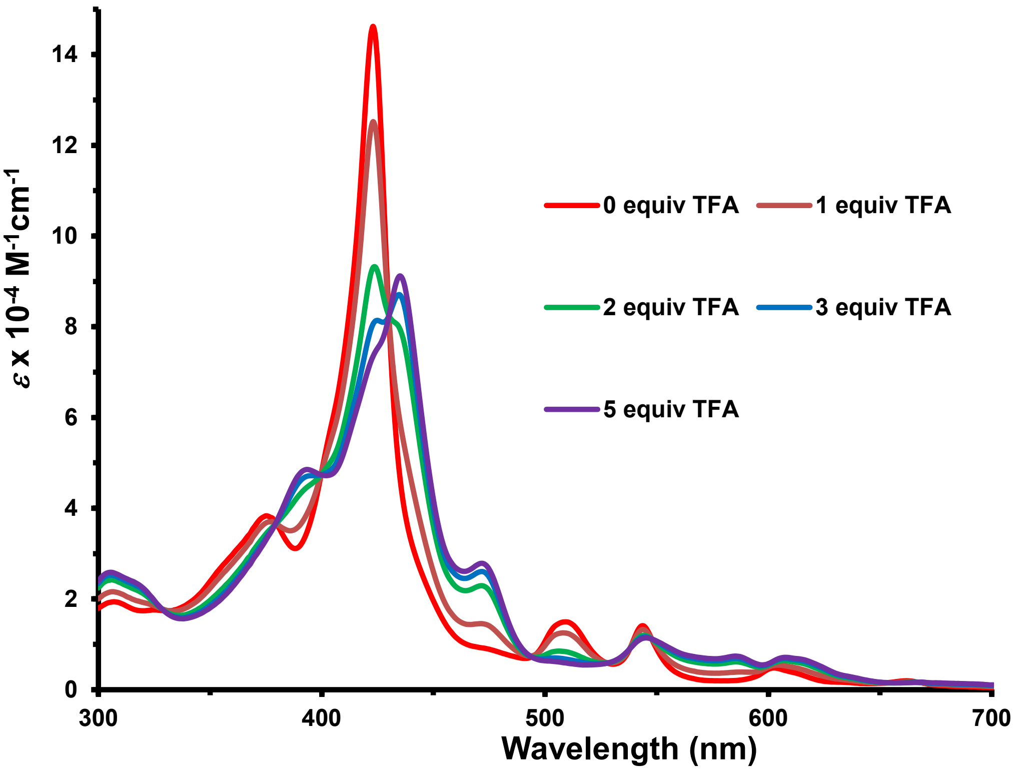

Figure S3. UV-vis spectra of tetramethylcarbaporphyrin **18a** in CH_2Cl_2 with 0-5 equivalents of TFA.

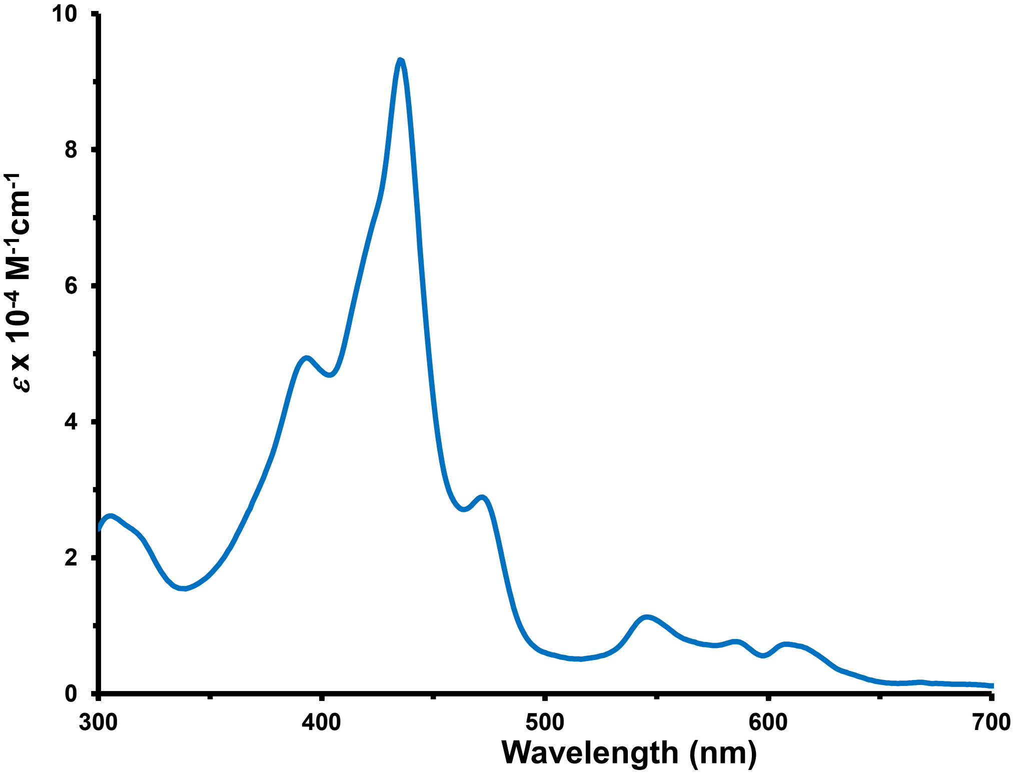

Figure S4. UV-vis spectrum of **18a** in CH_2Cl_2 with 10 equivalents of TFA.

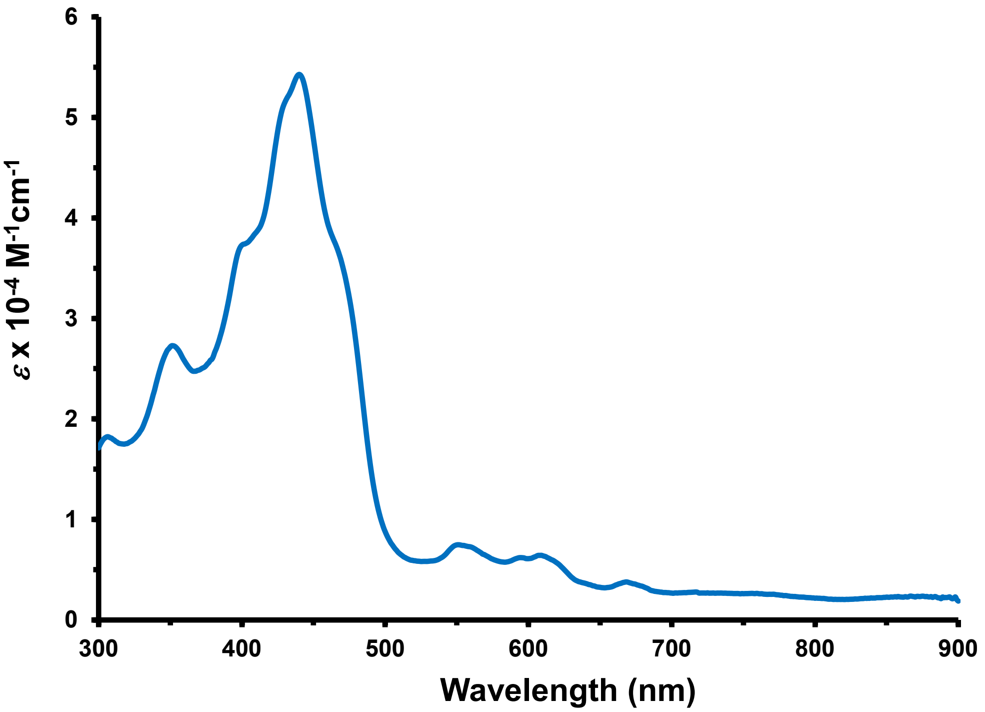

Figure S5. UV-vis spectrum of tetramethylcarbaporphyrin **18a** in 1% TFA-CH_2Cl_2.

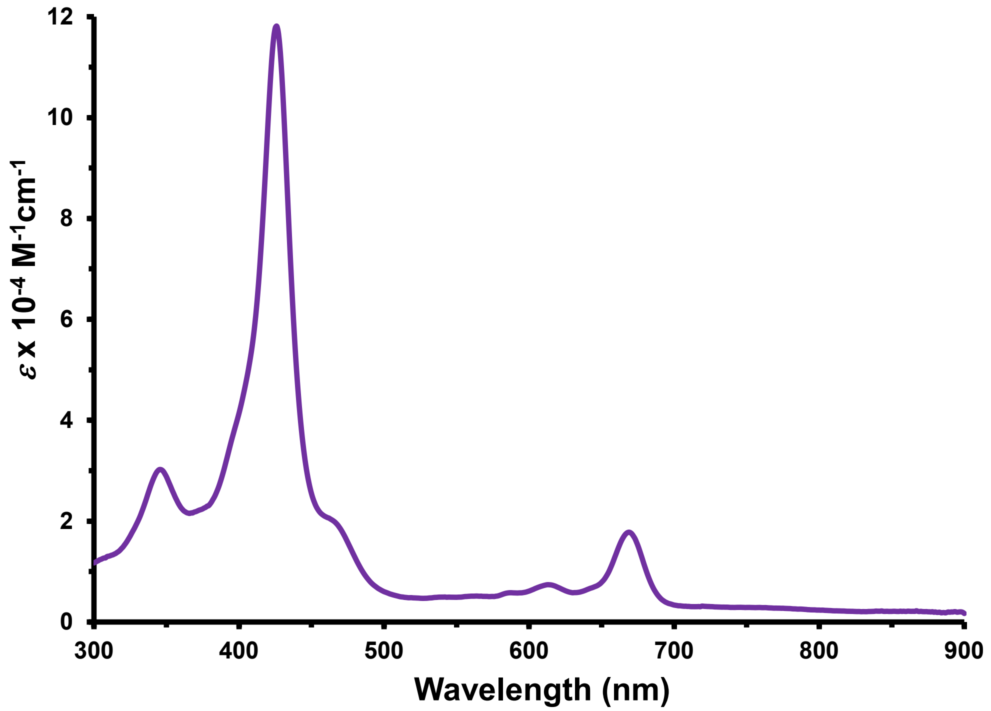

Figure S6. UV-vis spectrum of tetramethylcarbaporphyrin **18a** in 50% TFA-CH_2Cl_2.

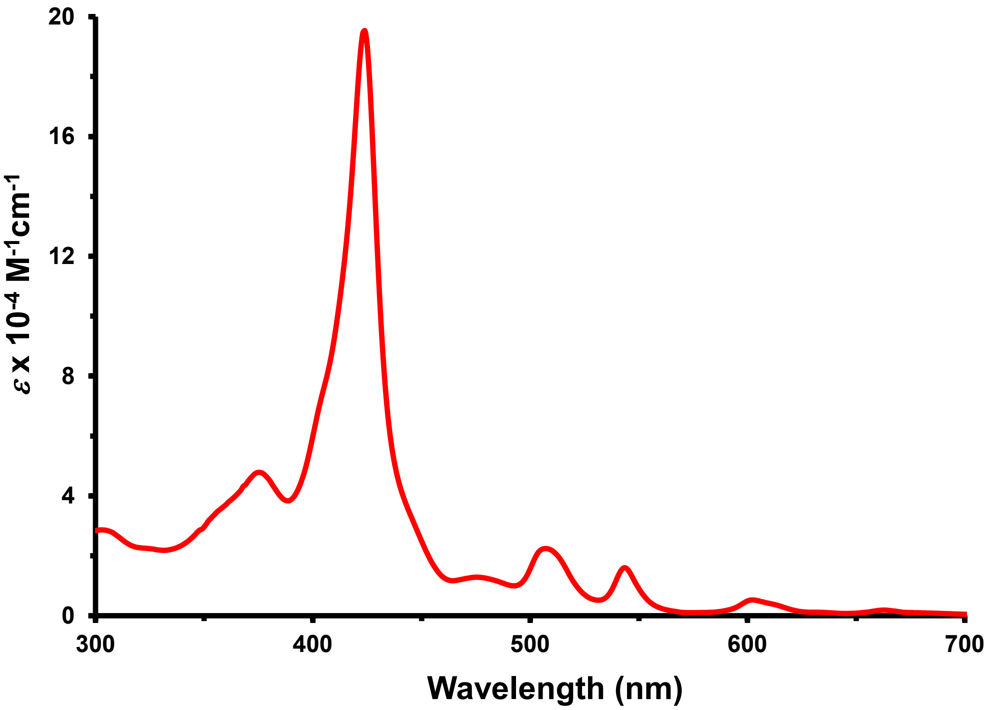

Figure S7. UV-vis spectrum of hexaethylcarbaporphyrin **18b** in 1% Et_3N-CH_2Cl_2.

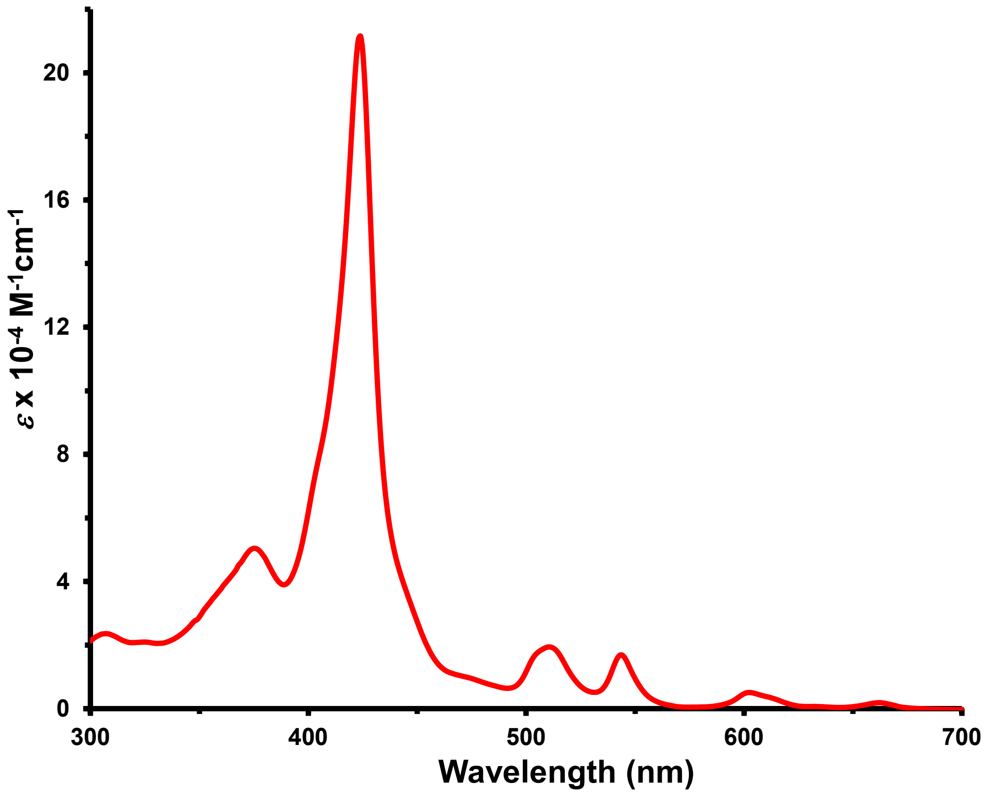

Figure S8. UV-vis spectrum of **18b** in CH_2Cl_2.

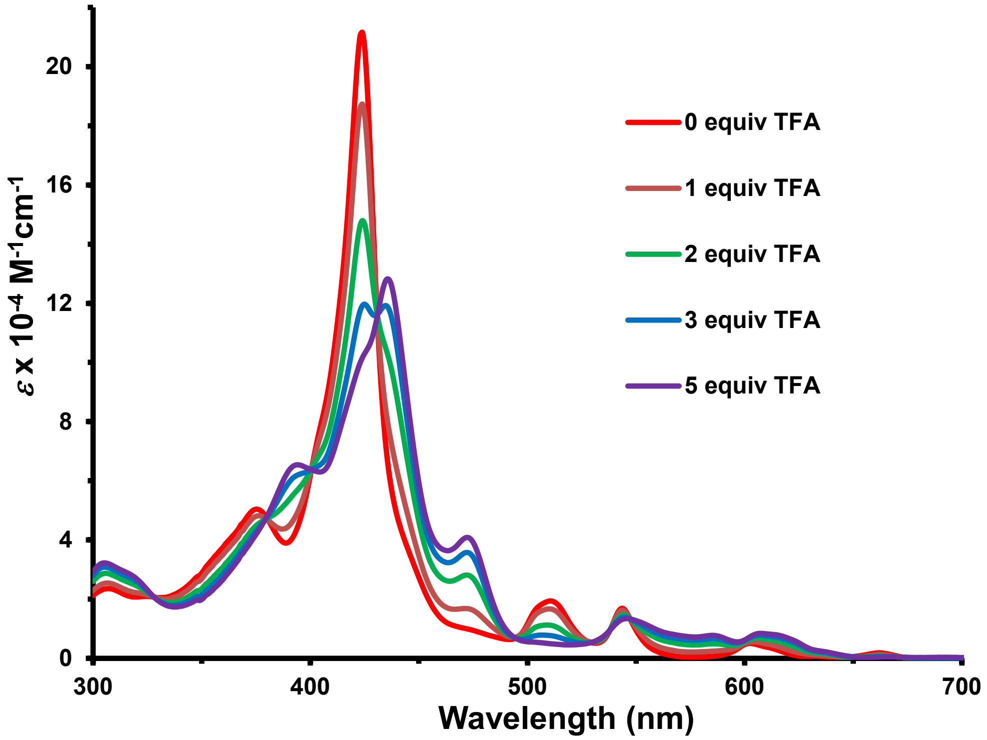

Figure S9. UV-vis spectra of **18b** in CH_2Cl_2 with 0-5 equivalents of TFA.

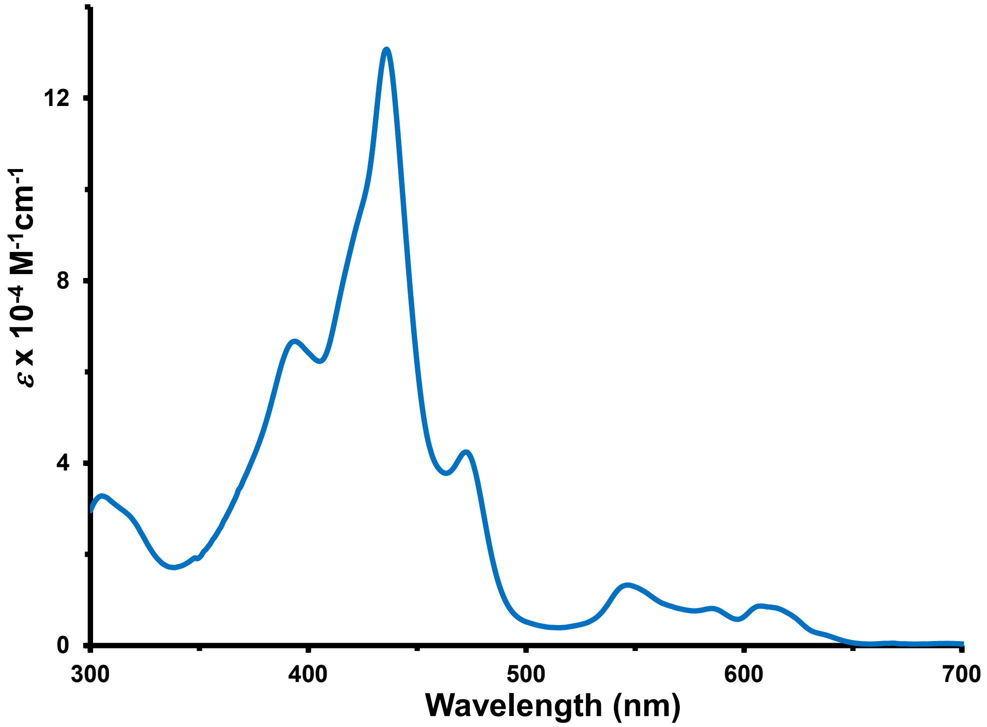

Figure S10. UV-vis spectrum of **18b** in CH_2Cl_2 with 10 equivalents of TFA.

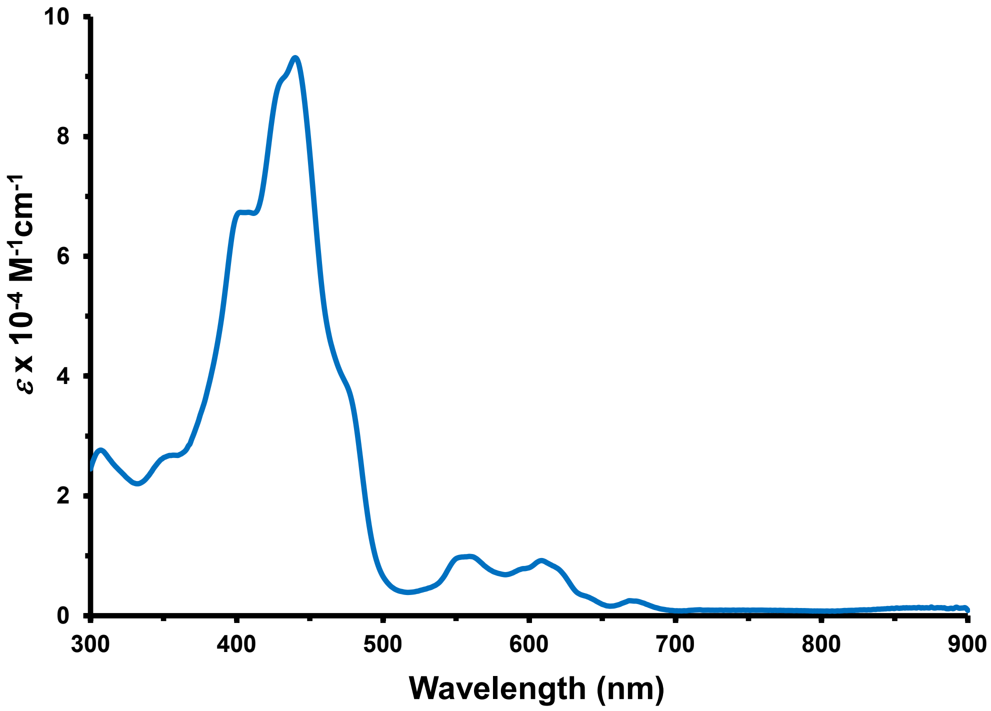

Figure S11. UV-vis spectrum of hexaethylcarbaporphyrin **18b** in 1% TFA-CH_2Cl_2.

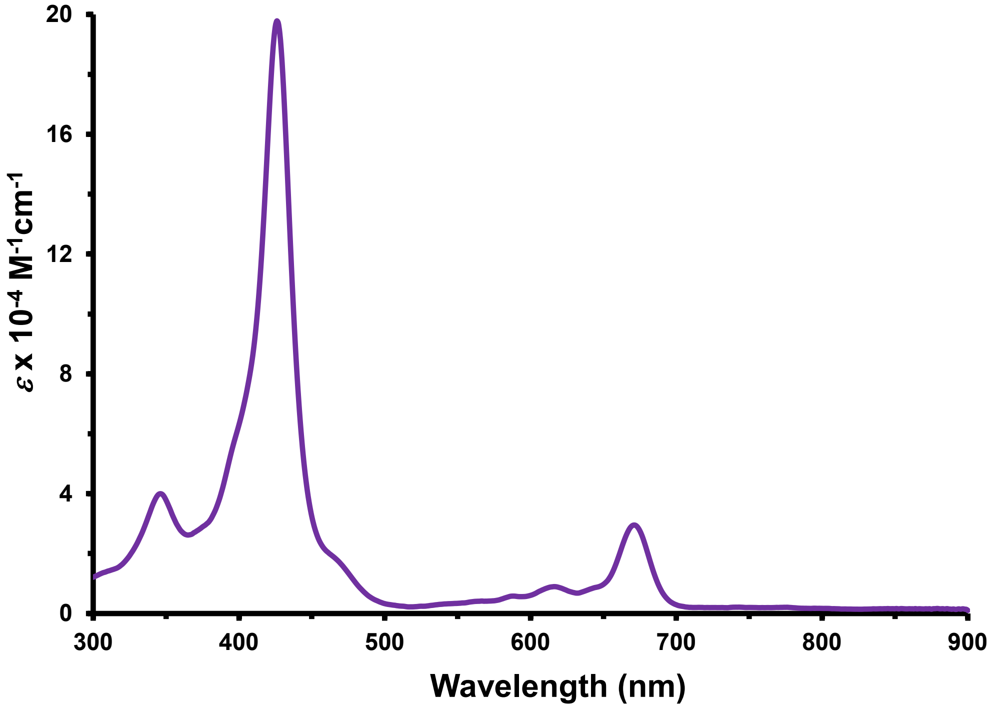

Figure S12. UV-vis spectrum of hexaethylcarbaporphyrin **18b** in 50% TFA-CH_2Cl_2.

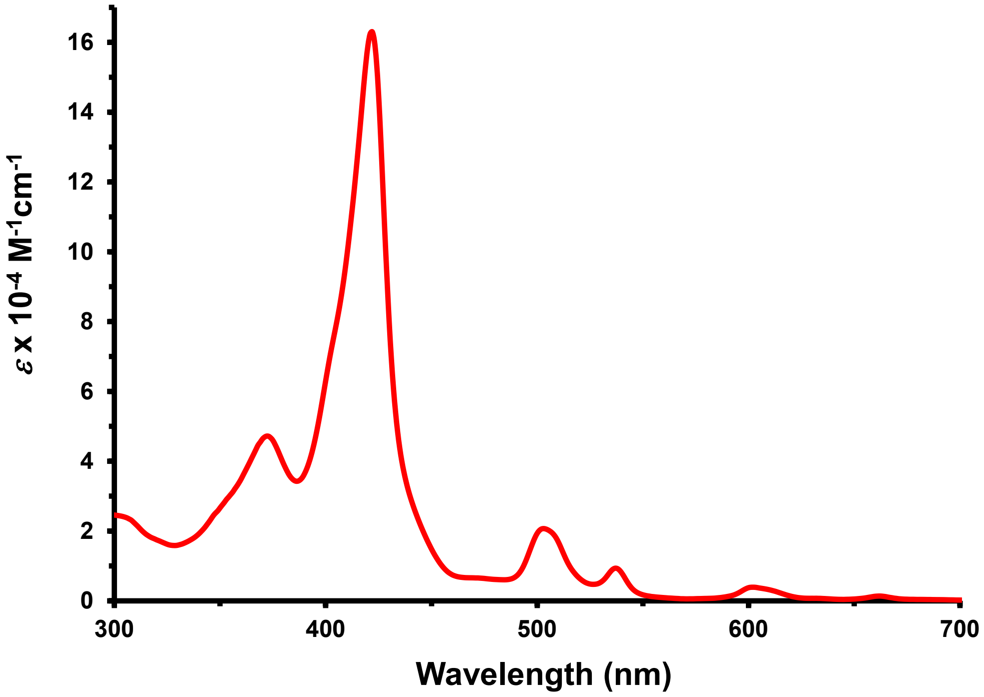

Figure S13. UV-vis spectrum of dimethylcarbaporphyrin **18c** in 1% Et_3N-CH_2Cl_2.

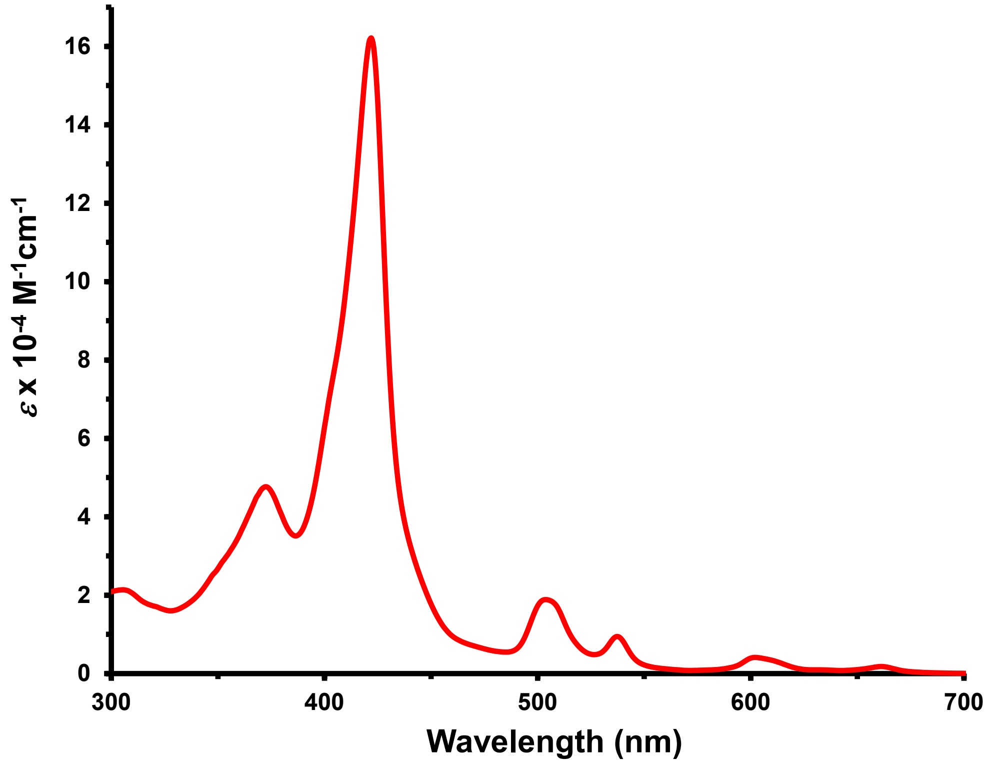

Figure S14. UV-vis spectrum of dimethylcarbaporphyrin **18c** in CH_2Cl_2.

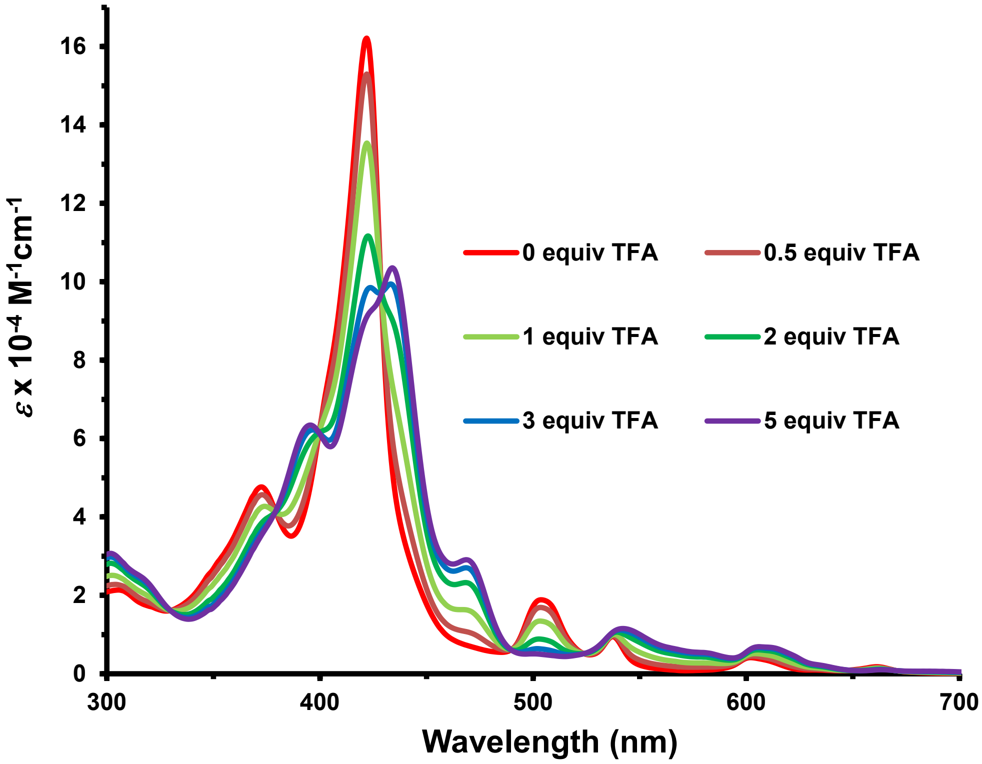

Figure S15. UV-vis spectra of **18c** in CH_2Cl_2 with o-5 equivalents of TFA.

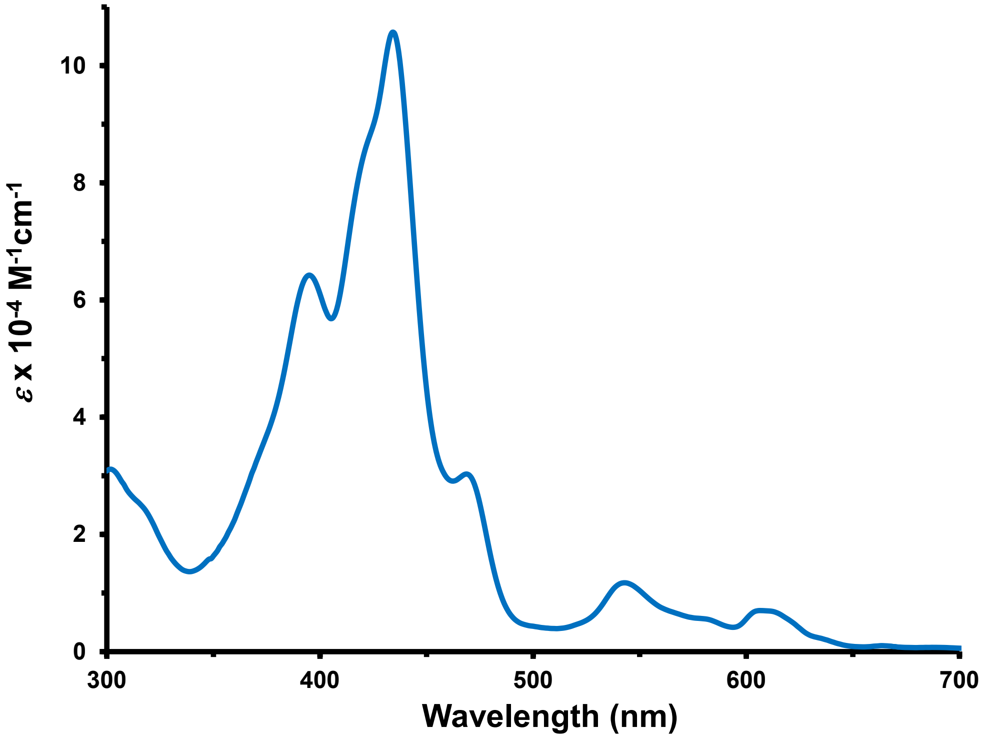

Figure S16. UV-vis spectrum of **18c** in CH_2Cl_2 with 10 equivalents of TFA.

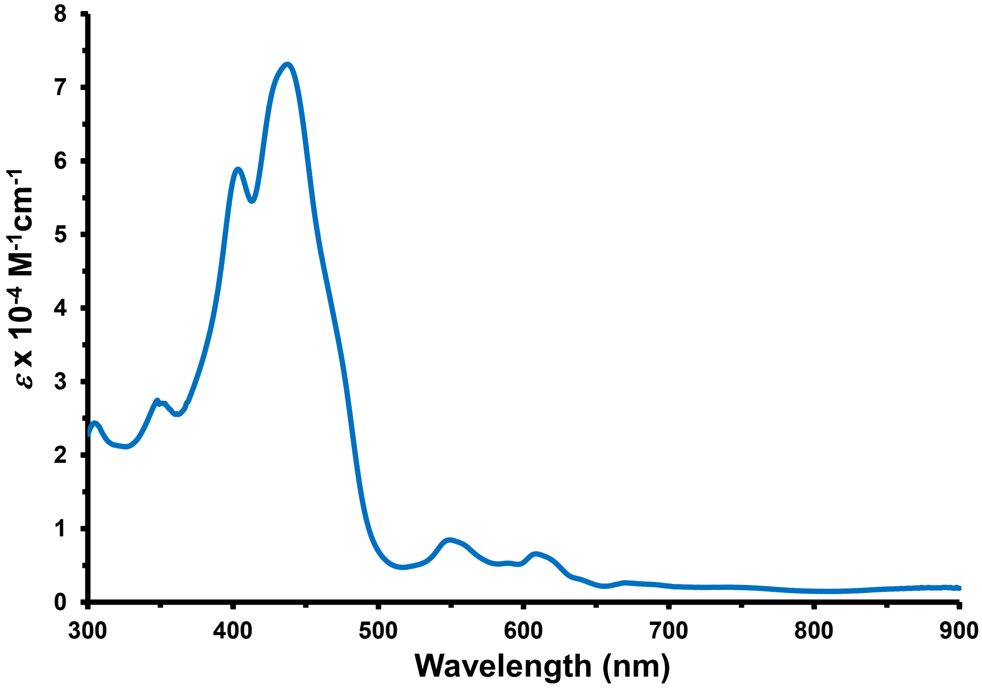

Figure S17. UV-vis spectrum of dimethylcarbaporphyrin **18c** in 1% TFA-CH_2Cl_2.

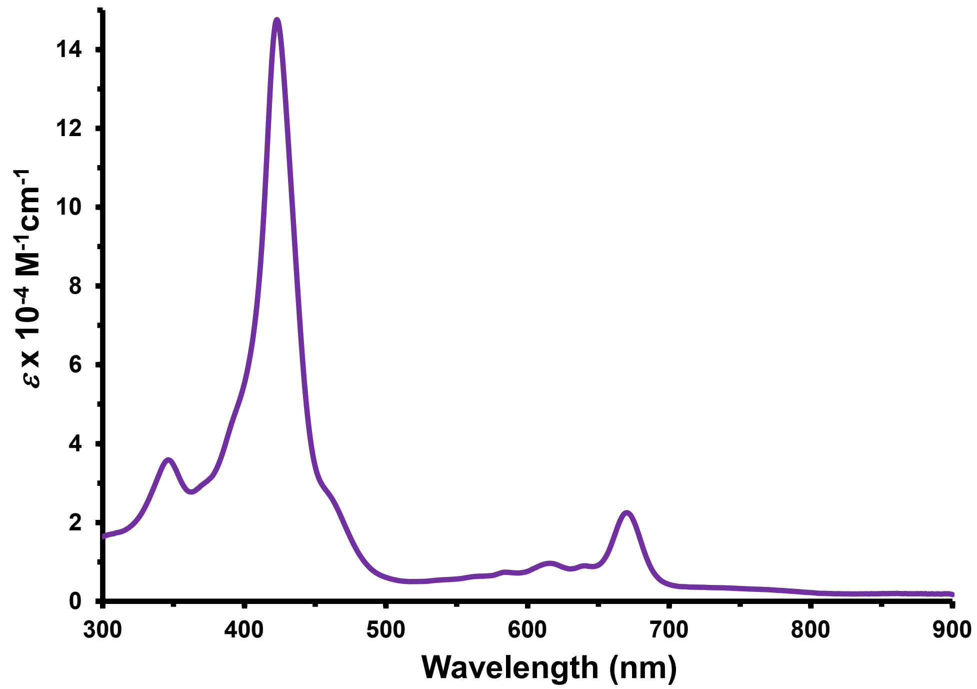

Figure S18. UV-vis spectrum of dimethylcarbaporphyrin **18c** in 50% TFA-CH_2Cl_2.

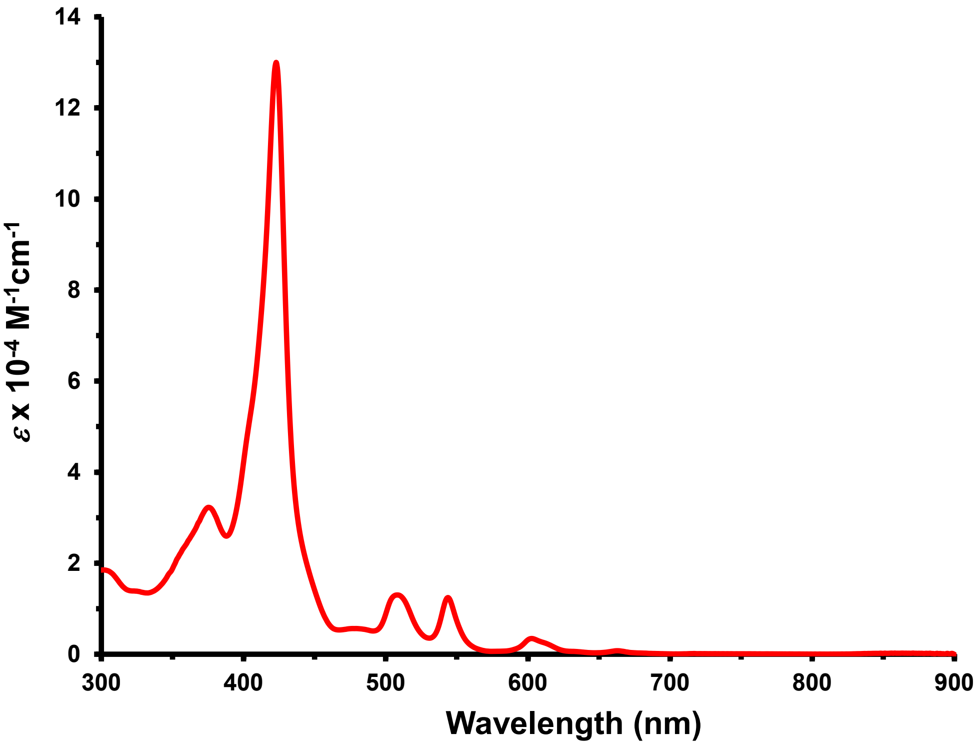

Figure S19. UV-vis spectrum of carbaporphyrin **18d** in 1% Et_3N-CH_2Cl_2.

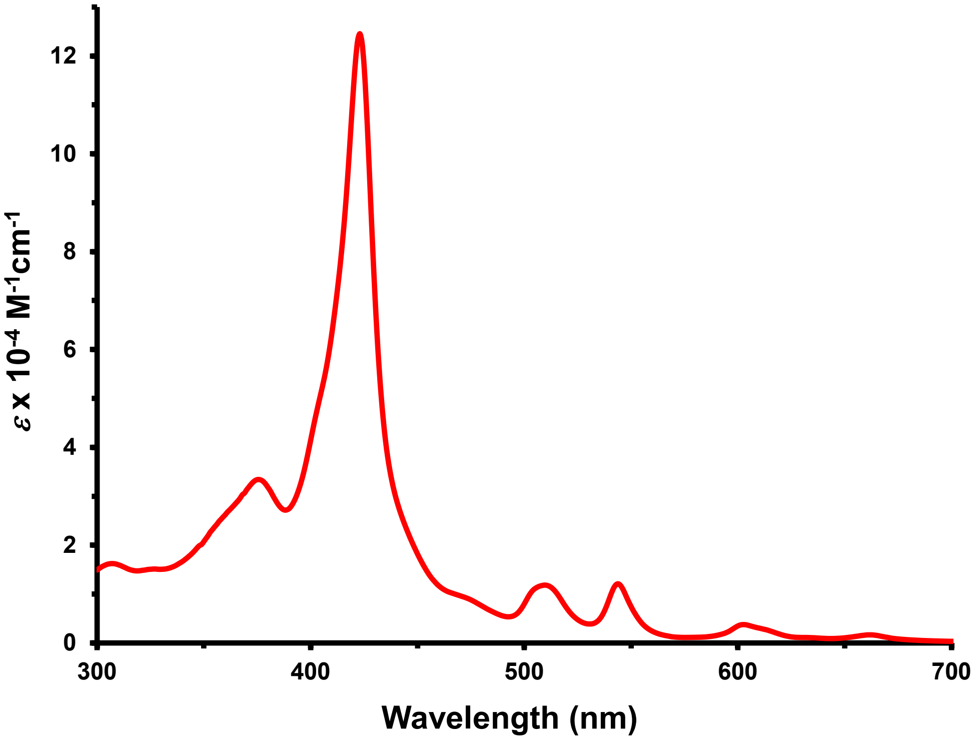

Figure S20. UV-vis spectrum of carbaporphyrin **18d** in CH_2Cl_2.

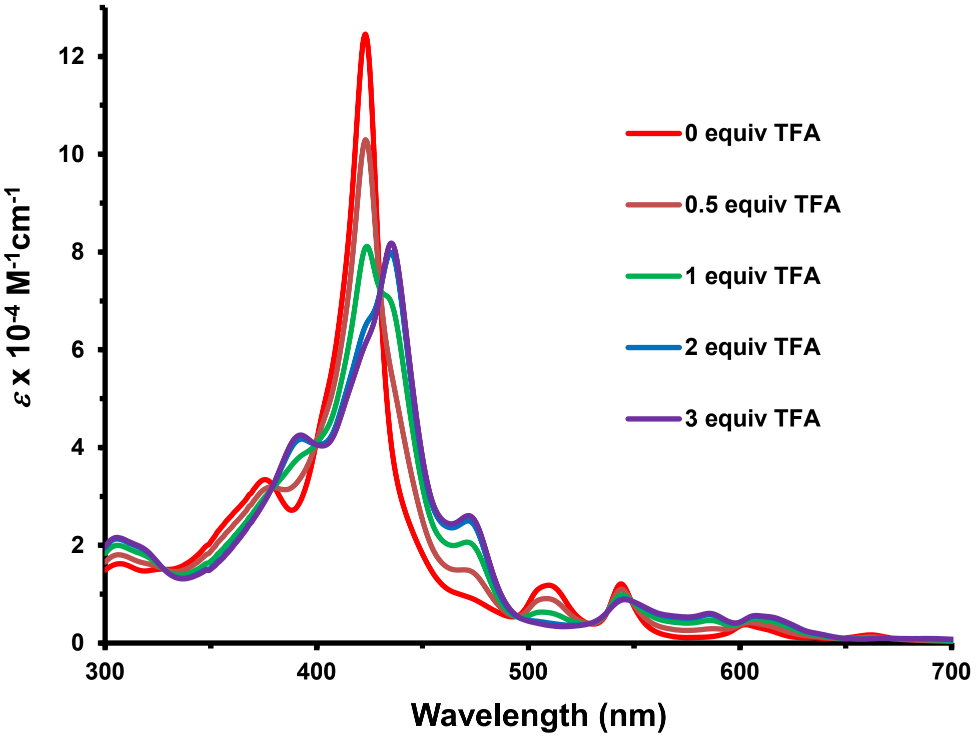

Figure S21. UV-vis spectra of carbaporphyrin **18d** in CH_2Cl_2 with 0-3 equivalents of TFA.

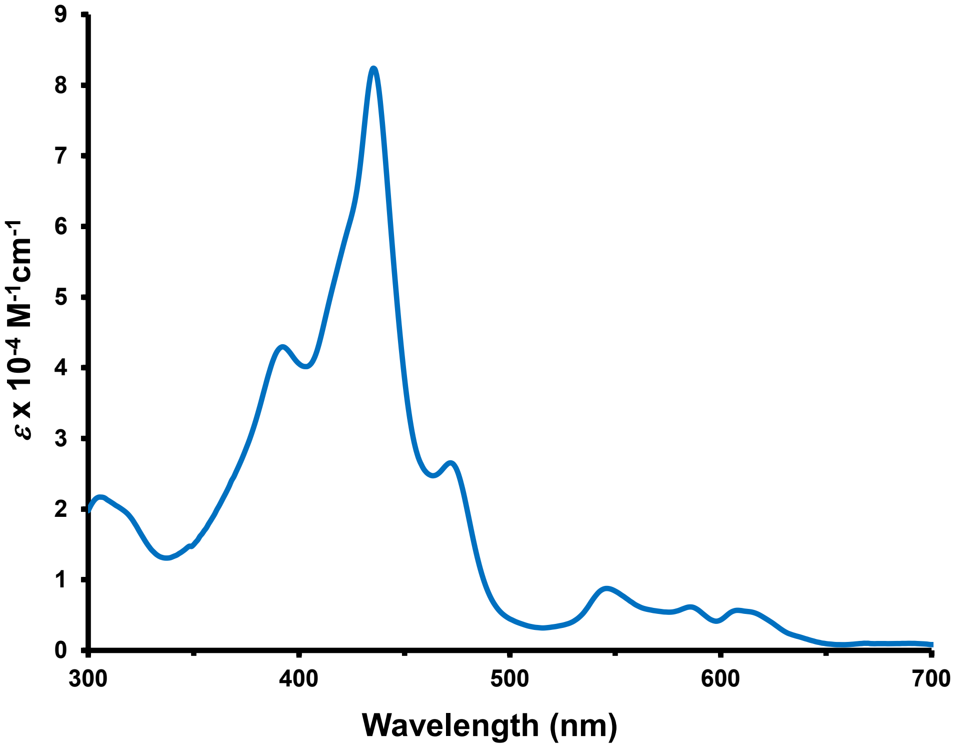

Figure S22. UV-vis spectrum of carbaporphyrin **18d** in CH_2Cl_2 with 5 equivalents of TFA.

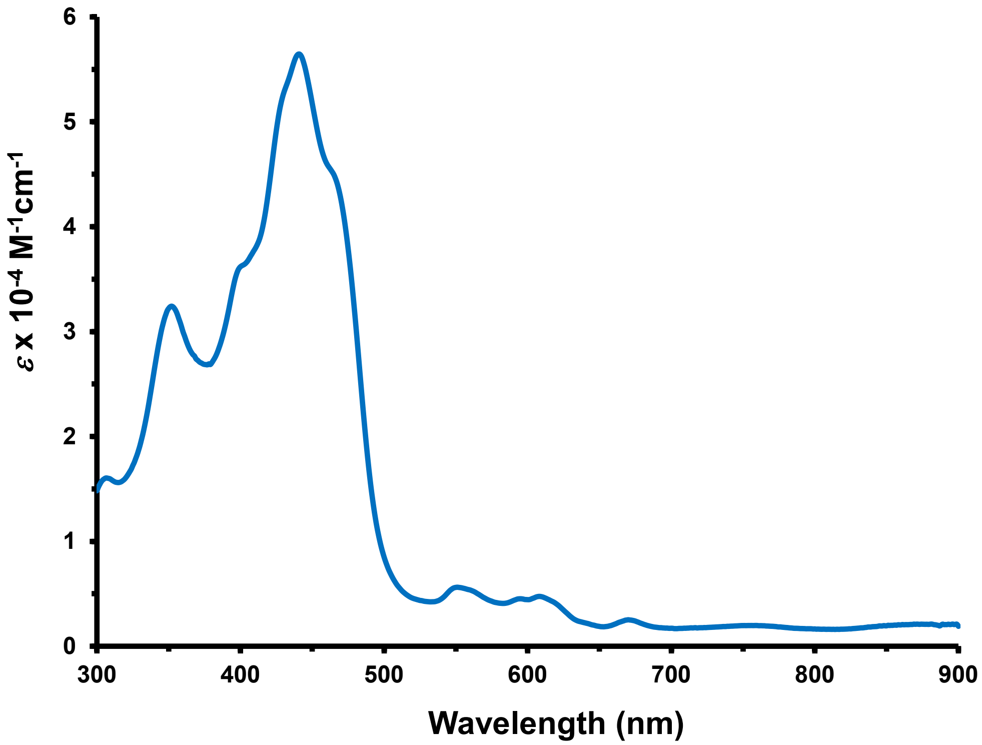

Figure S23. UV-vis spectrum of carbaporphyrin **18d** in 1% TFA-CH_2Cl_2.

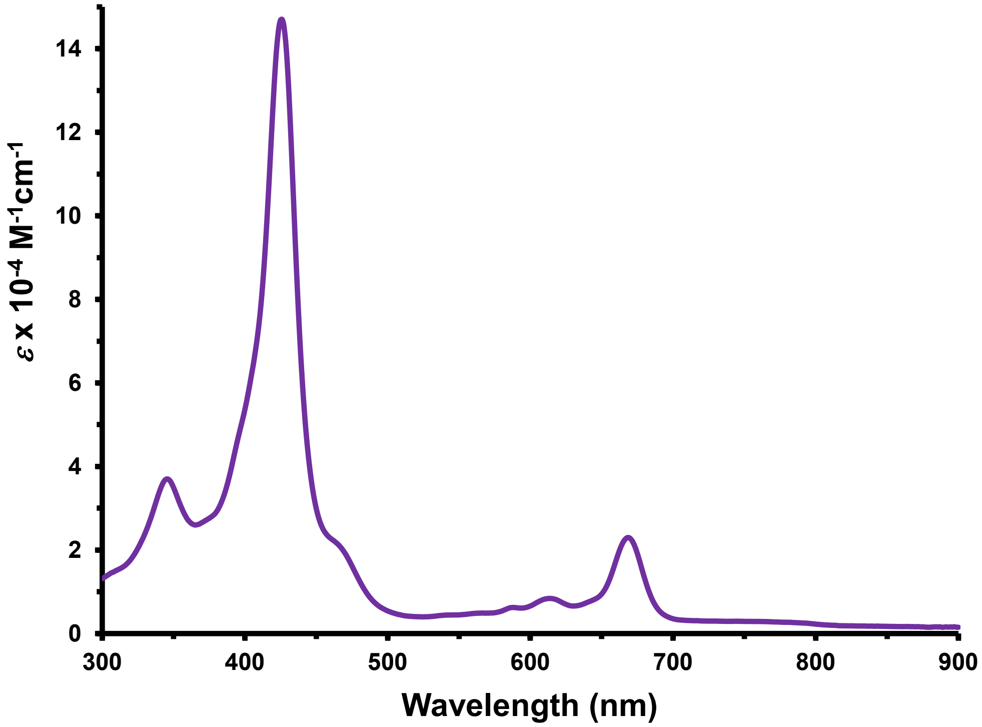

Figure S24. UV-vis spectrum of carbaporphyrin **18d** in 50% Et_3N-CH_2Cl_2.

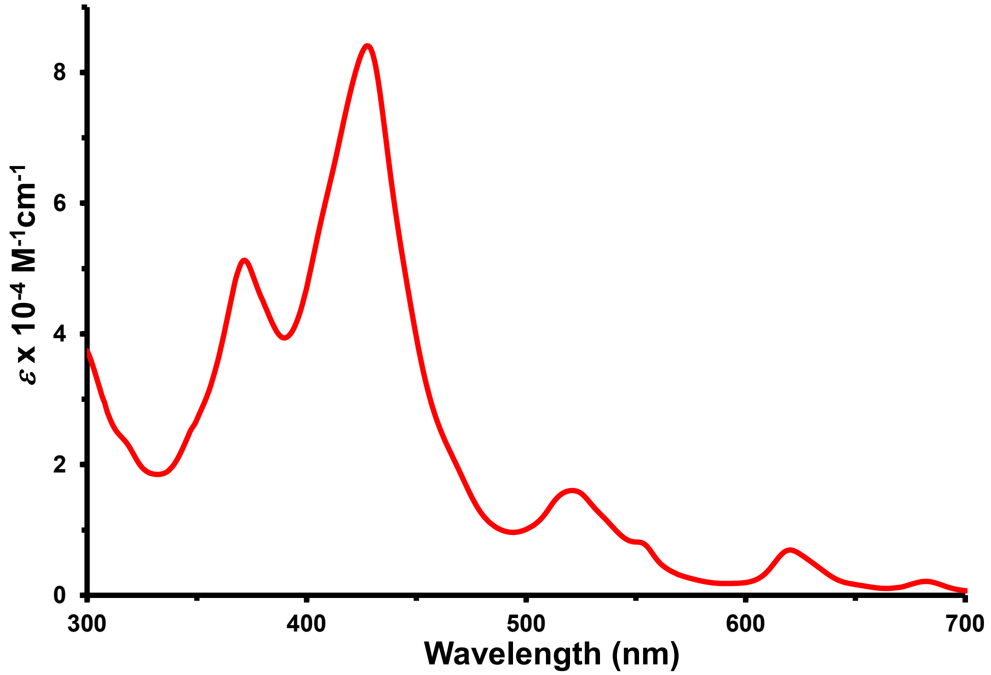

Figure S25. UV-vis spectrum of oxacarbaporphyrin **19a** in 1% Et_3N-CH_2Cl_2.

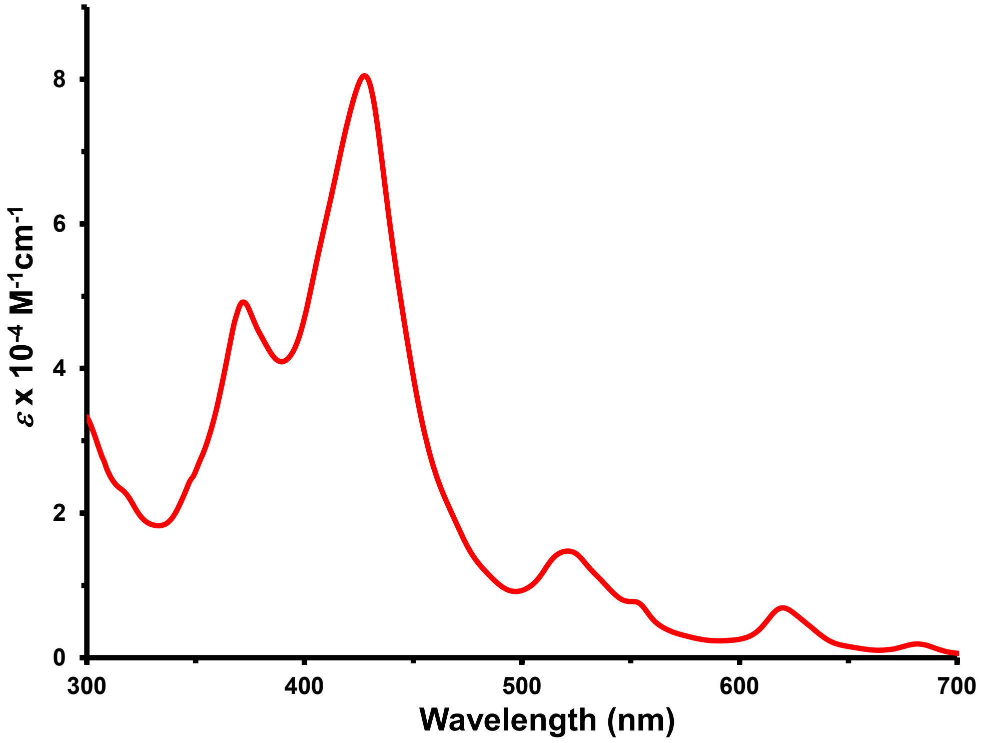

Figure S26. UV-vis spectrum of oxacarbaporphyrin **19a** in CH_2Cl_2.

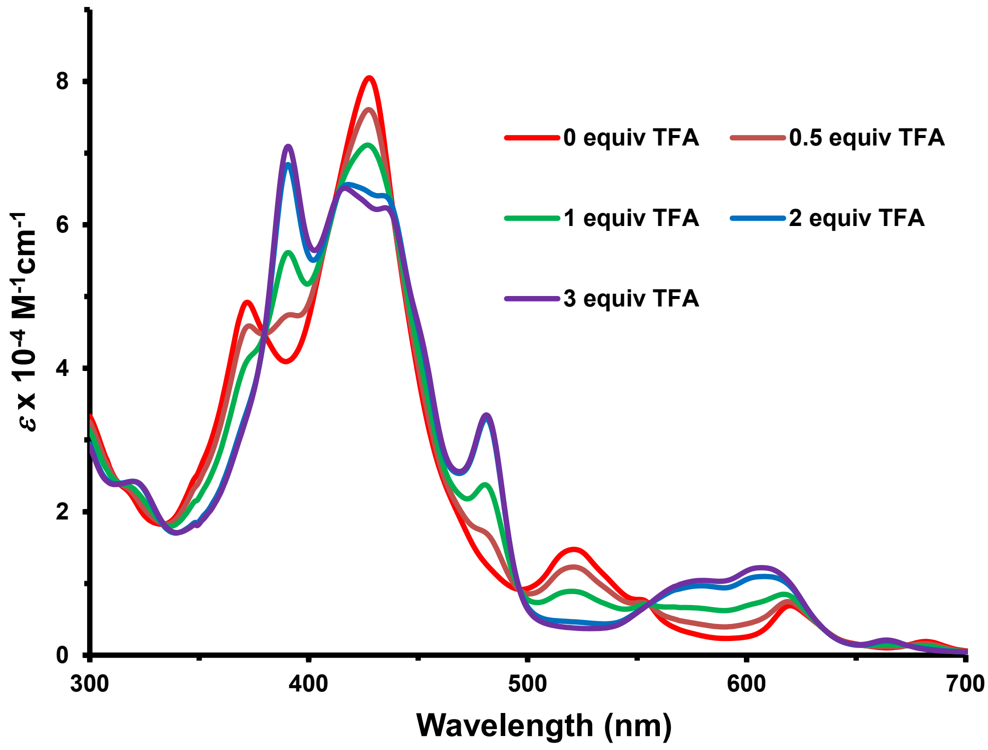

Figure S27. UV-vis spectra of **19a** in CH_2Cl_2 with 0-3 equivalents of TFA.

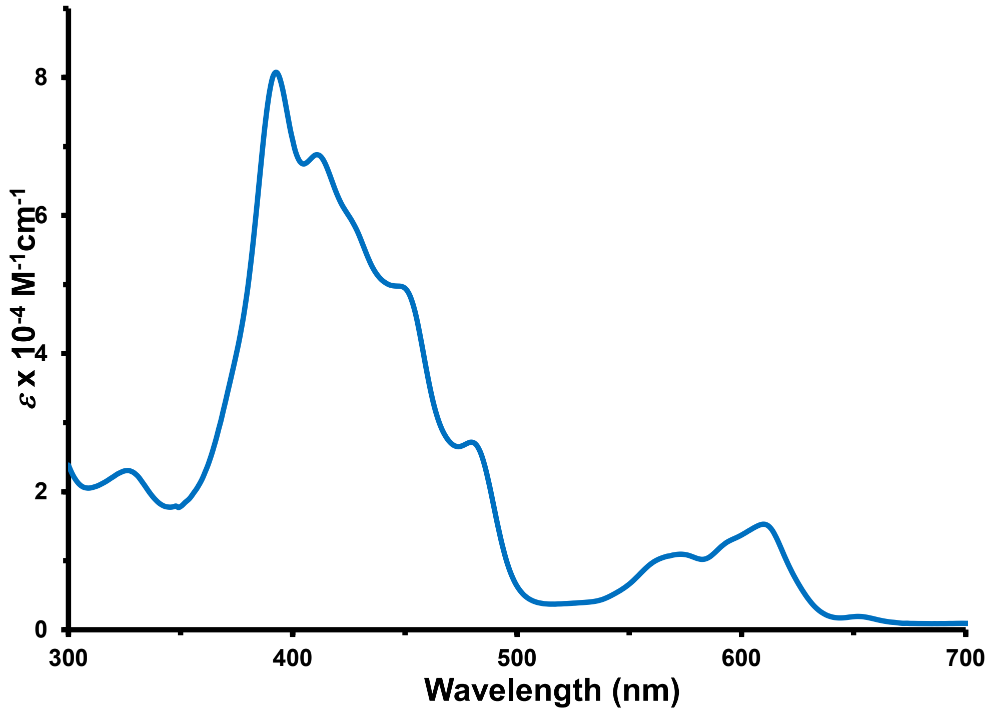

Figure S28. UV-vis spectrum of oxacarbaporphyrin **19a** in 1% TFA-CH_2Cl_2.

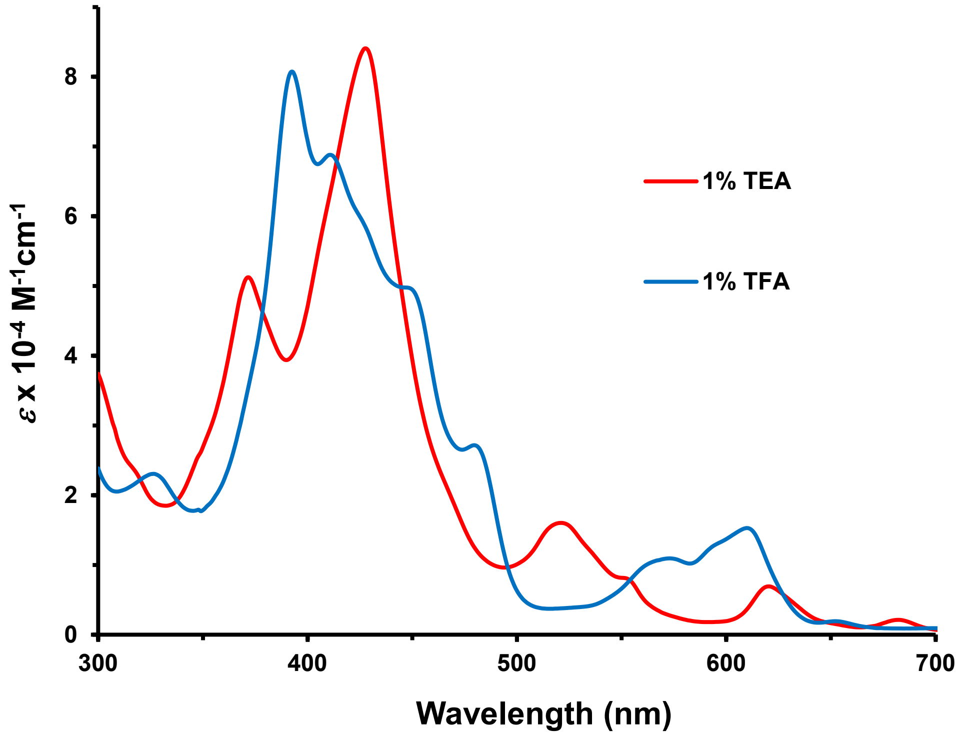

Figure S29. UV-vis spectra of oxacarbaporphyrin **19a** in 1% Et_3N-CH_2Cl_2 (red line) and 1% TFA-CH_2Cl_2 (blue line).

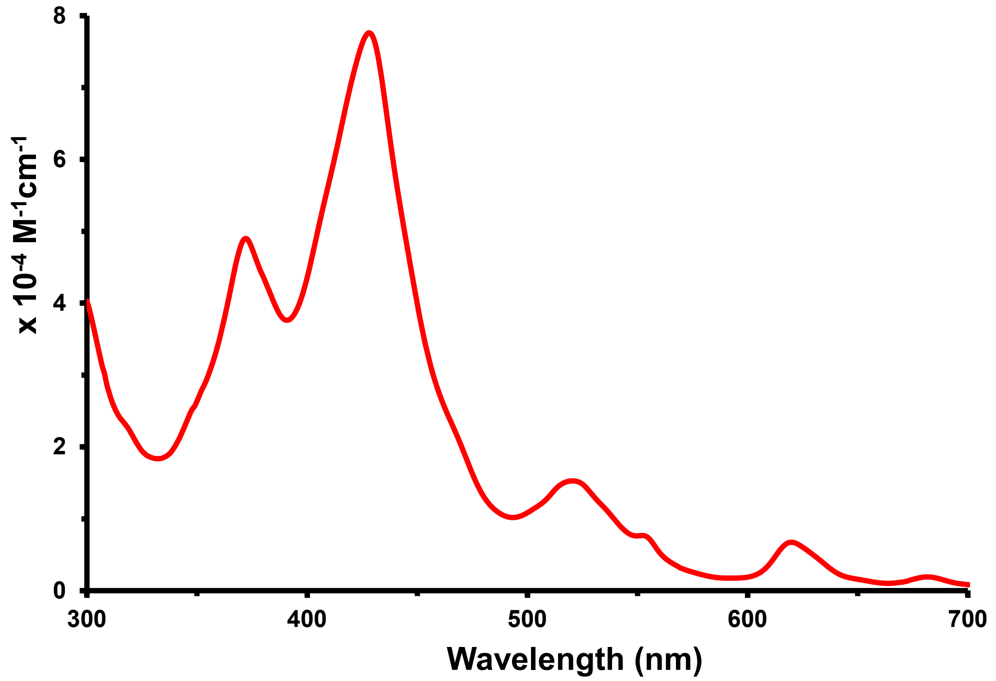

Figure S30. UV-vis spectrum of oxacarbaporphyrin **19b** in 1% Et_3N-CH_2Cl_2.

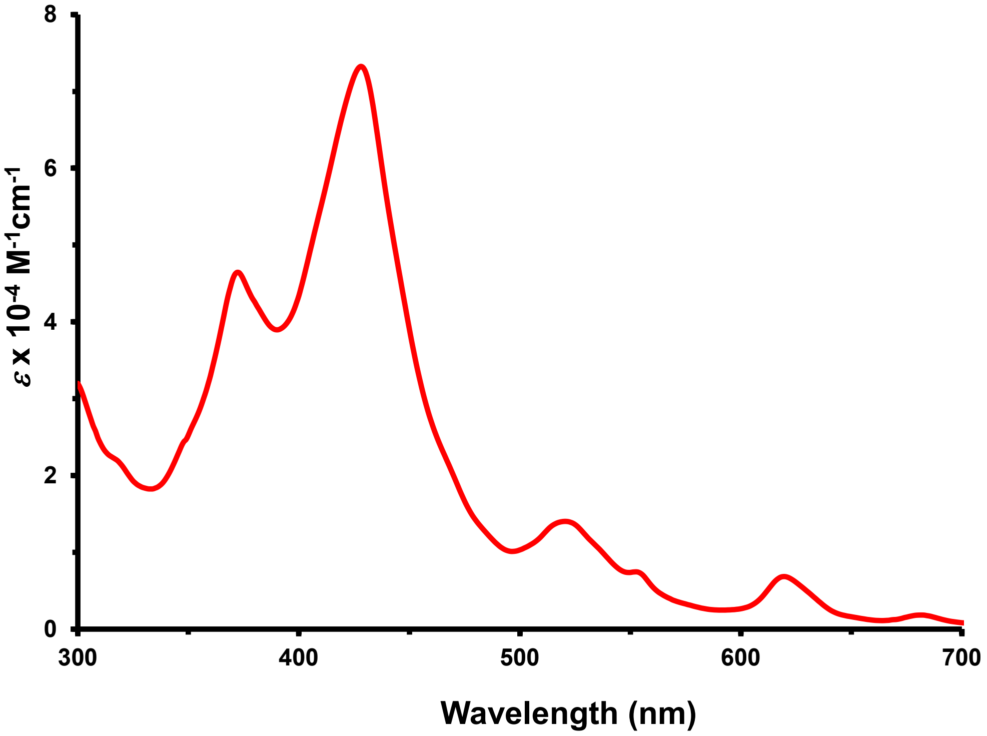

Figure S31. UV-vis spectrum of oxacarbaporphyrin **19b** in CH_2Cl_2.

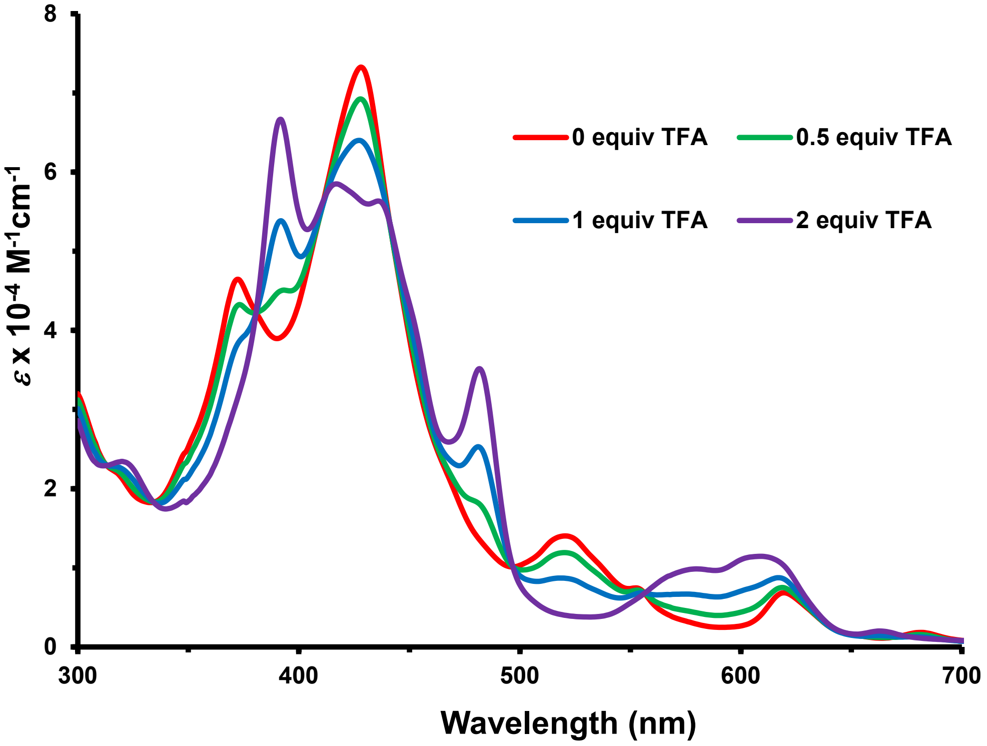

Figure S32. UV-vis spectra of **19b** in CH_2Cl_2 with 0-2 equivalents of TFA.

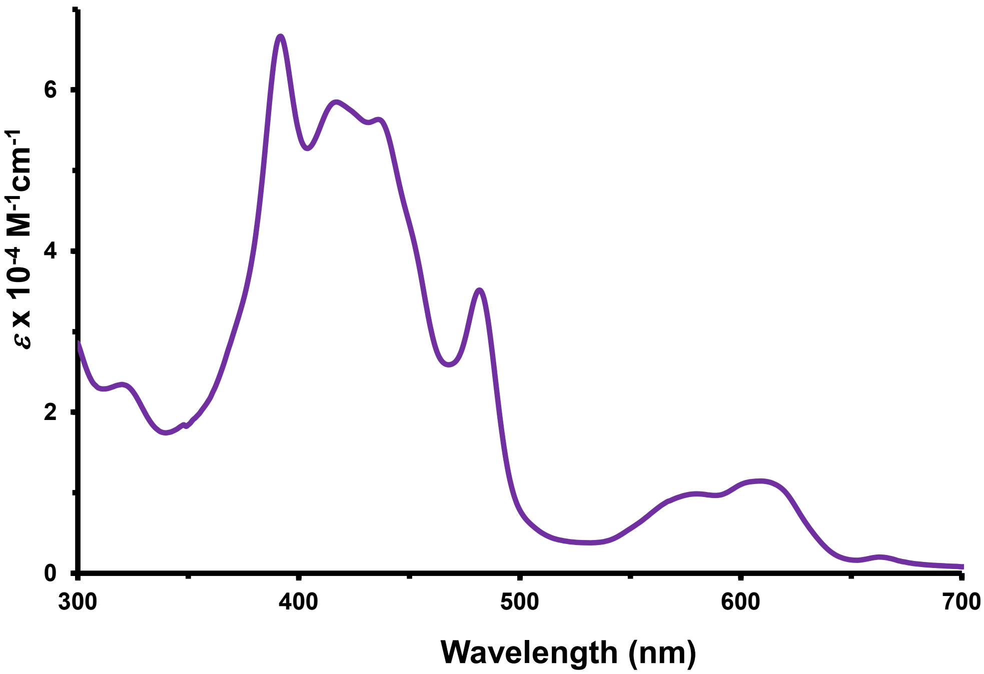

Figure S33. UV-vis spectrum of oxacarbaporphyrin **19b** in CH_2Cl_2 with 3 equivalents of TFA.

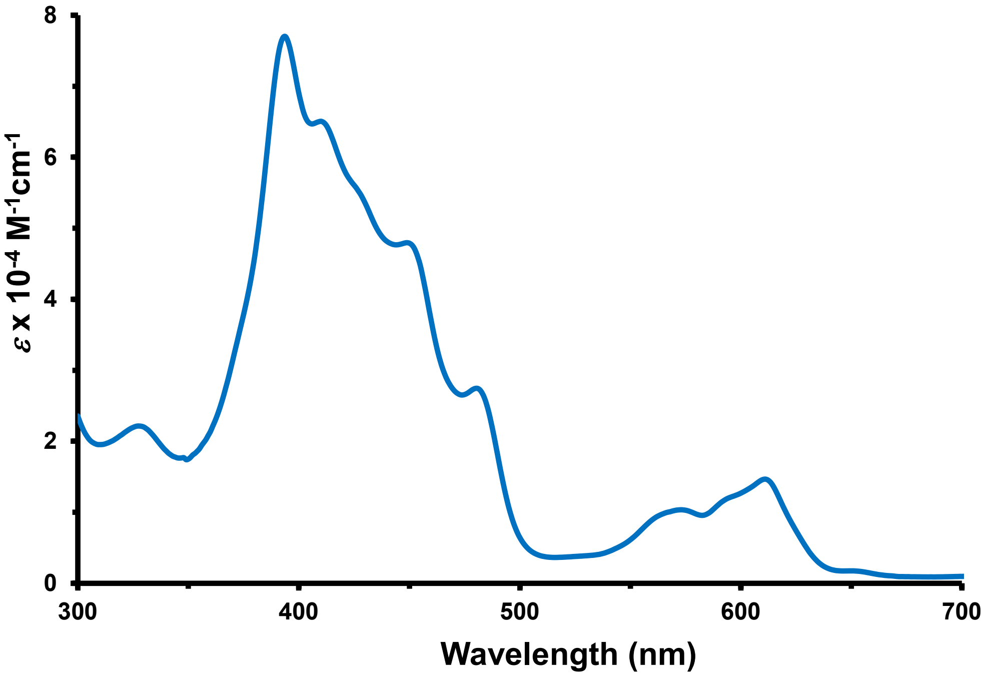

Figure S34. UV-vis spectrum of oxacarbaporphyrin **19b** in 1% TFA-CH_2Cl_2.

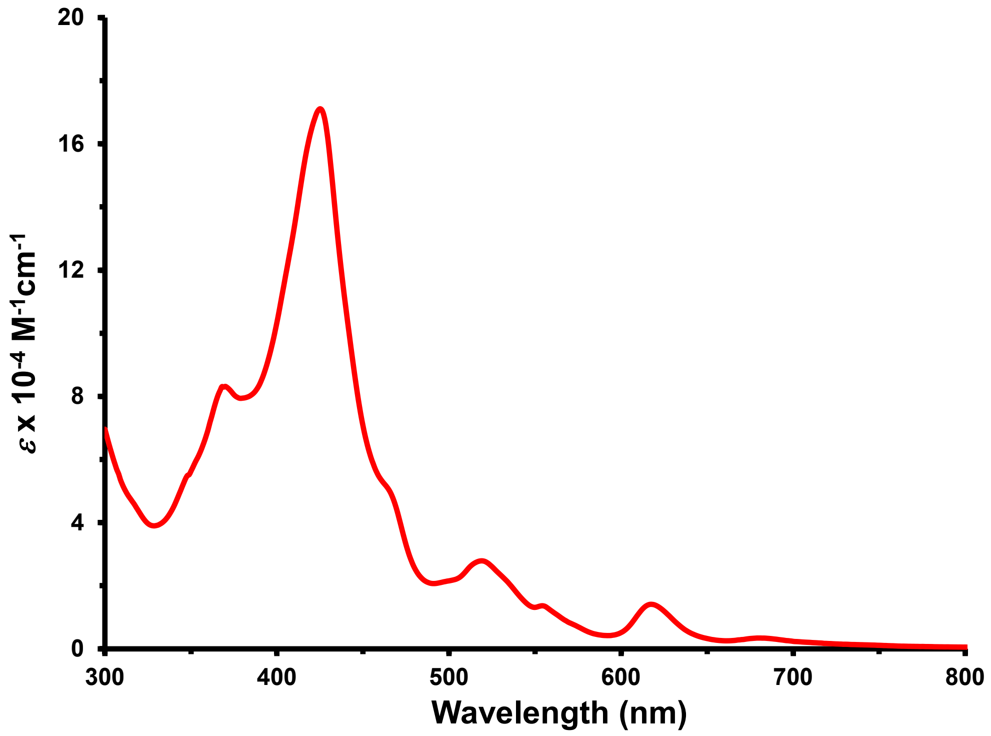

Figure S35. UV-vis spectrum of oxacarbaporphyrin **19c** in 1% Et_3N-CH_2Cl_2.

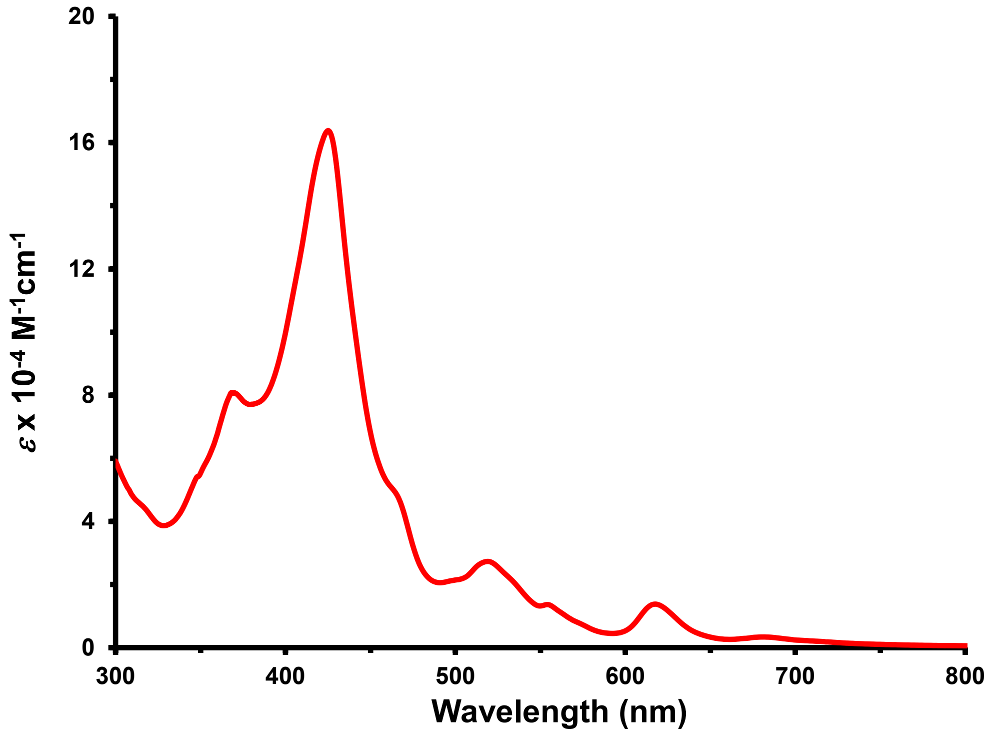

Figure S36. UV-vis spectrum of oxacarbaporphyrin **19c** in CH_2Cl_2.

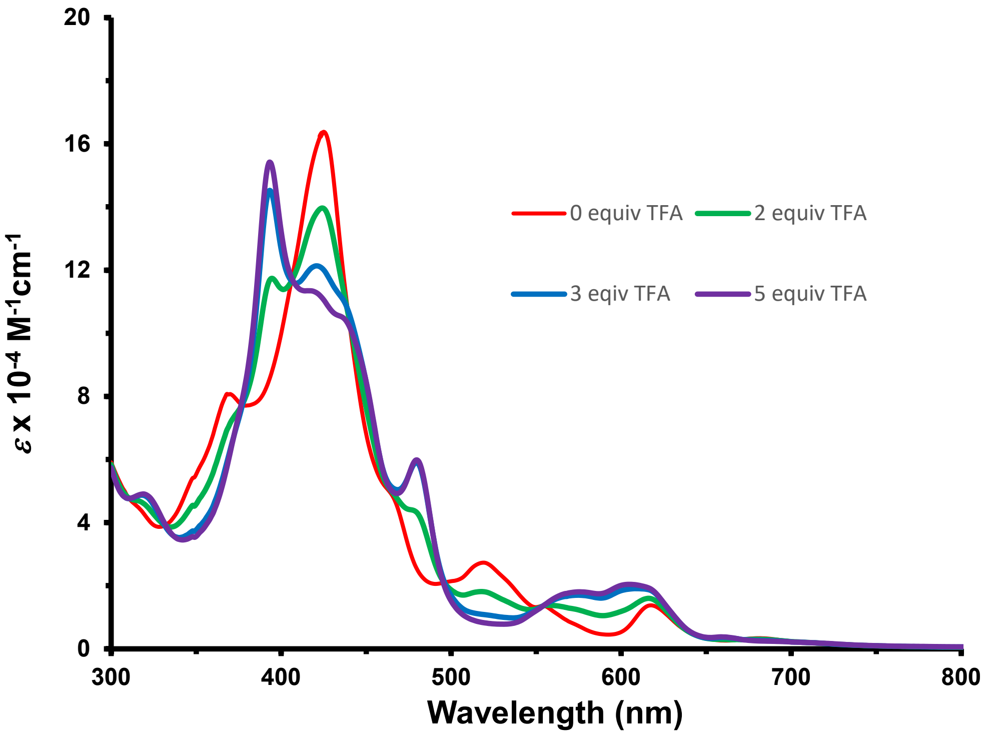

Figure S37. UV-vis spectra of oxacarbaporphyrin **19c** in CH_2Cl_2 with 0-5 equivalents of TFA.

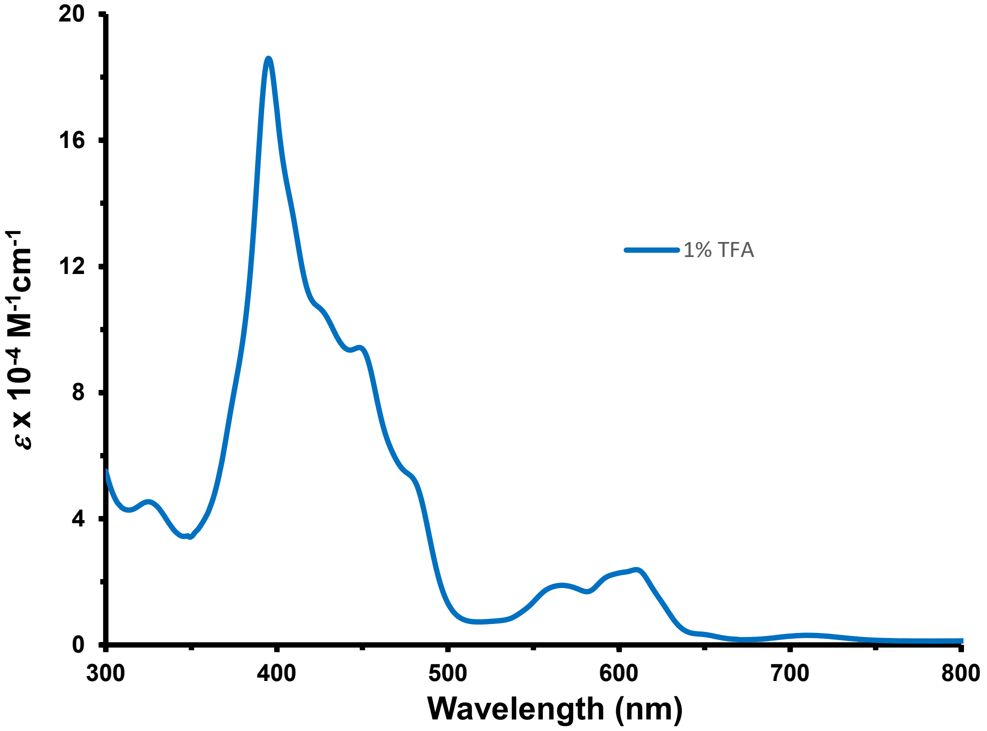

Figure S38. UV-vis spectrum of oxacarbaporphyrin **19c** in 1% TFA-CH_2Cl_2.

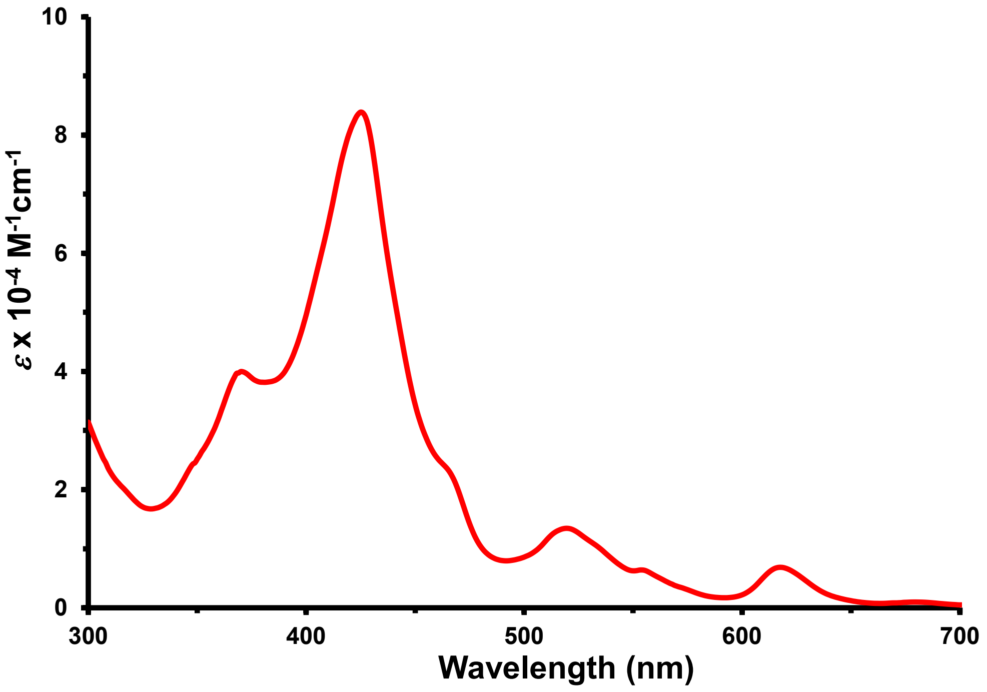

Figure S39. UV-vis spectrum of oxacarbaporphyrin **19d** in 1% Et_3N-CH_2Cl_2.

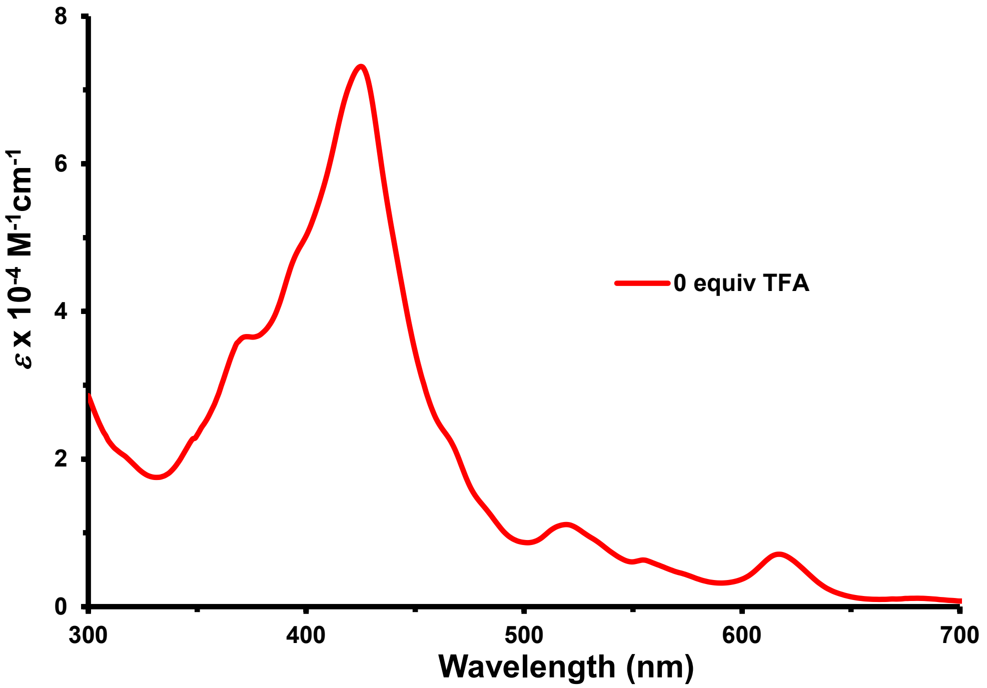

Figure S40. UV-vis spectrum of oxacarbaporphyrin **19d** in CH_2Cl_2.

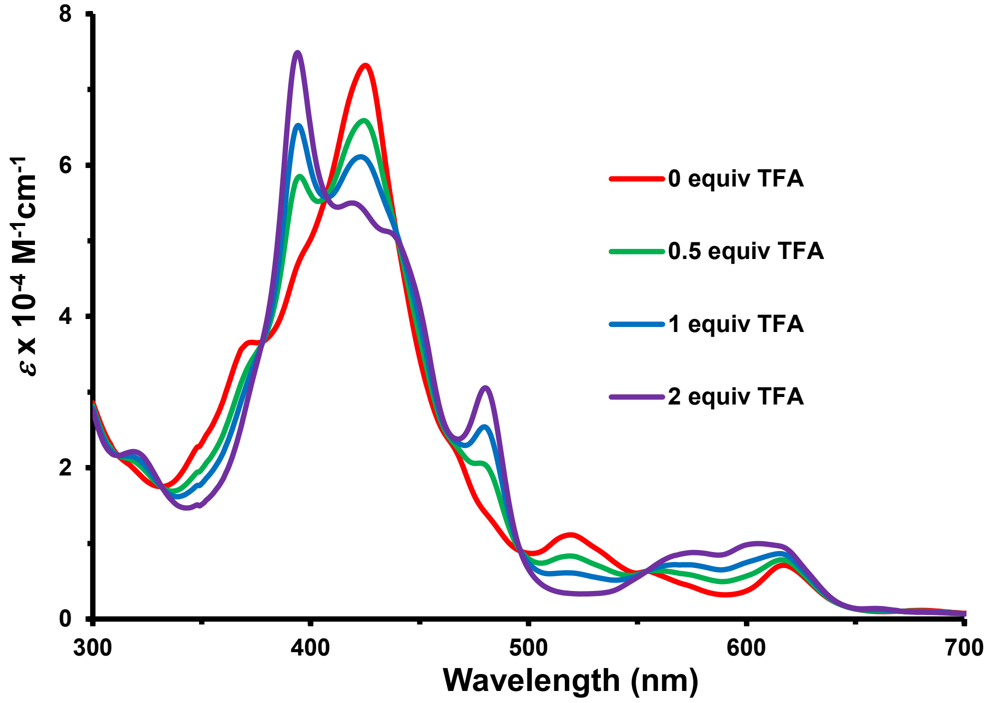

Figure S41. UV-vis spectra of **19d** in CH_2Cl_2 with 0-2 equivalents of TFA.

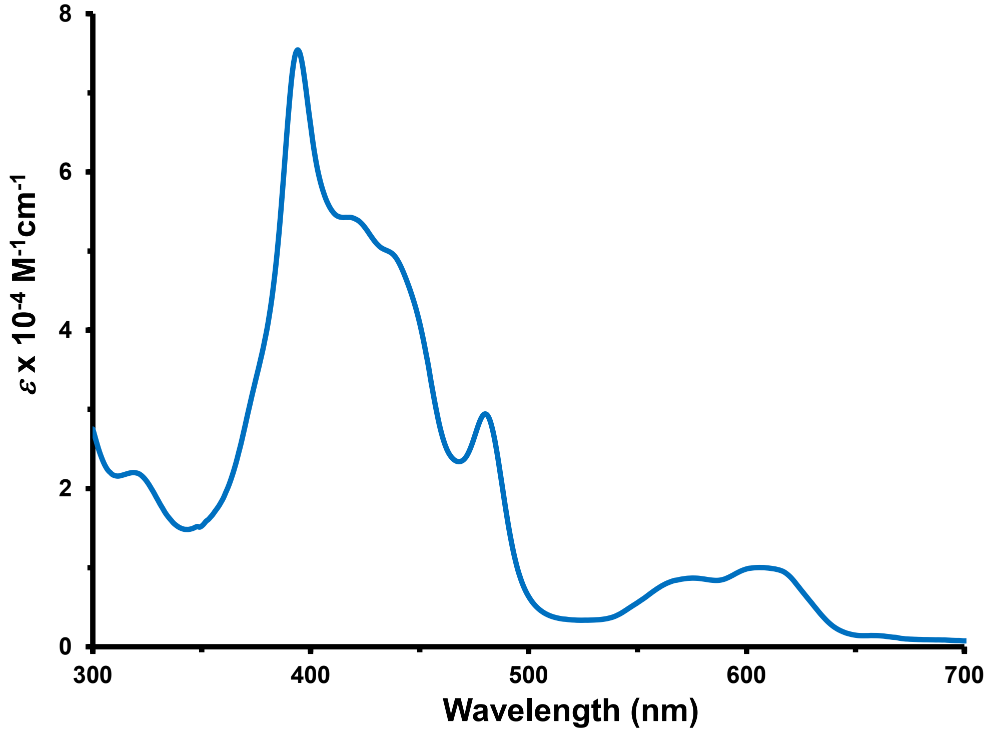

Figure S42. UV-vis spectrum of oxacarbaporphyrin **19d** in CH_2Cl_2 with 3 equivalents of TFA.

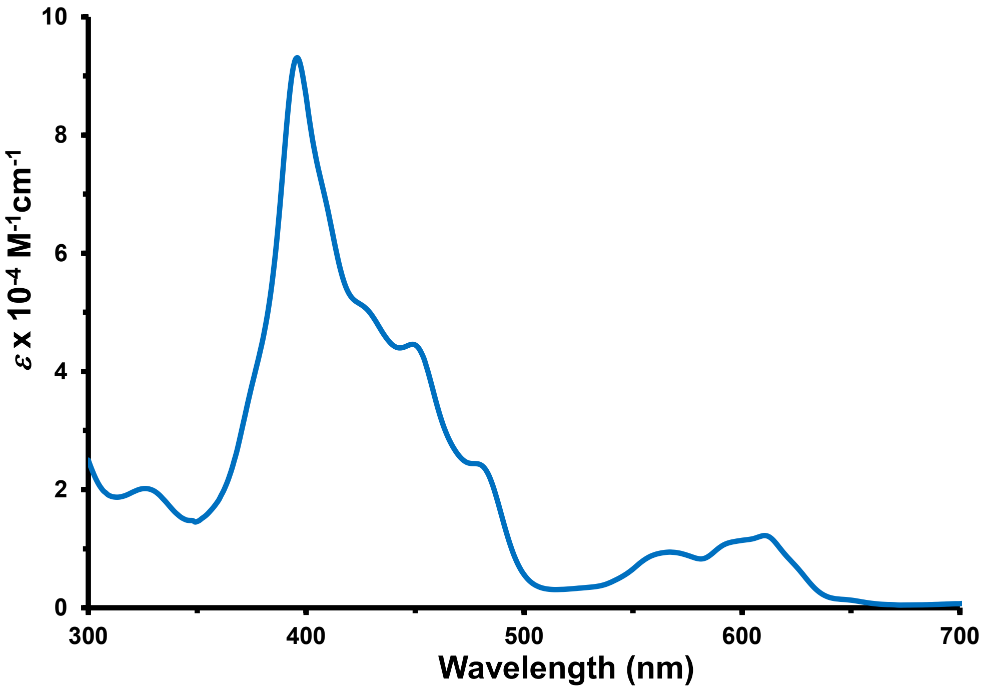

Figure S43. UV-vis spectrum of oxacarbaporphyrin **19d** in 1% TFA-CH_2Cl_2.

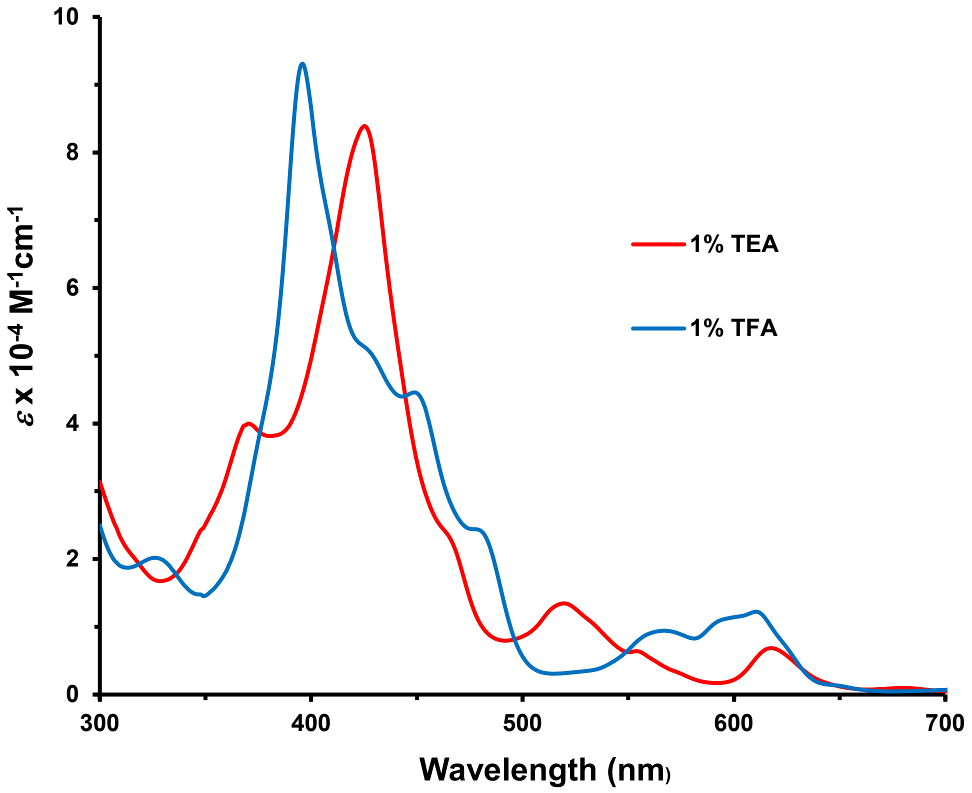

Figure S44. UV-vis spectra of oxacarbaporphyrin **19d** in 1% Et_3N-CH_2Cl_2 (blue line) and 1% TFA-CH_2Cl_2 (red line).

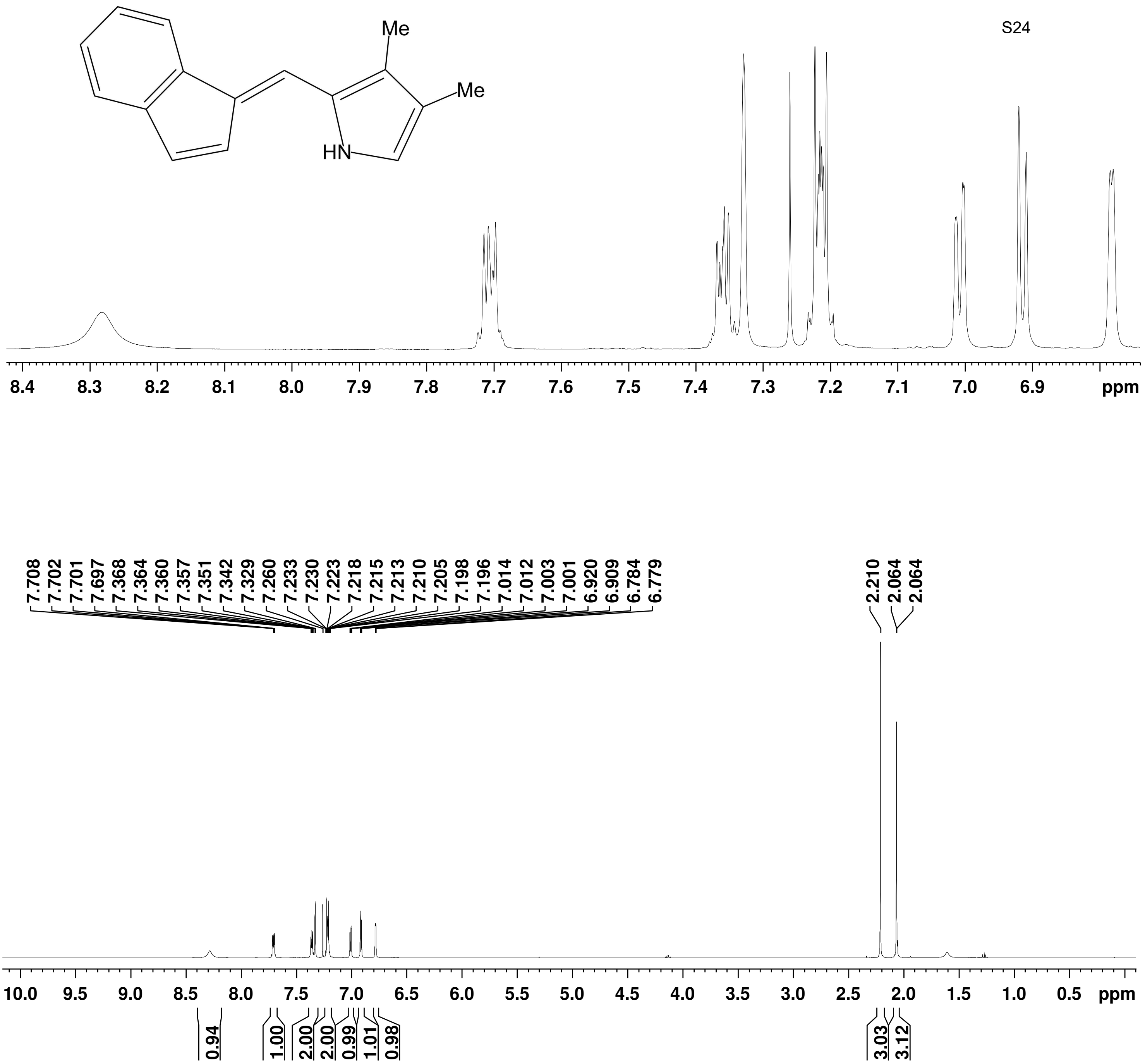

Figure S45. 500 MHz proton NMR (above) and ^{1}H-^{1}H COSY NMR (right) spectra of fulvene **16a** in $CDCl_3$.

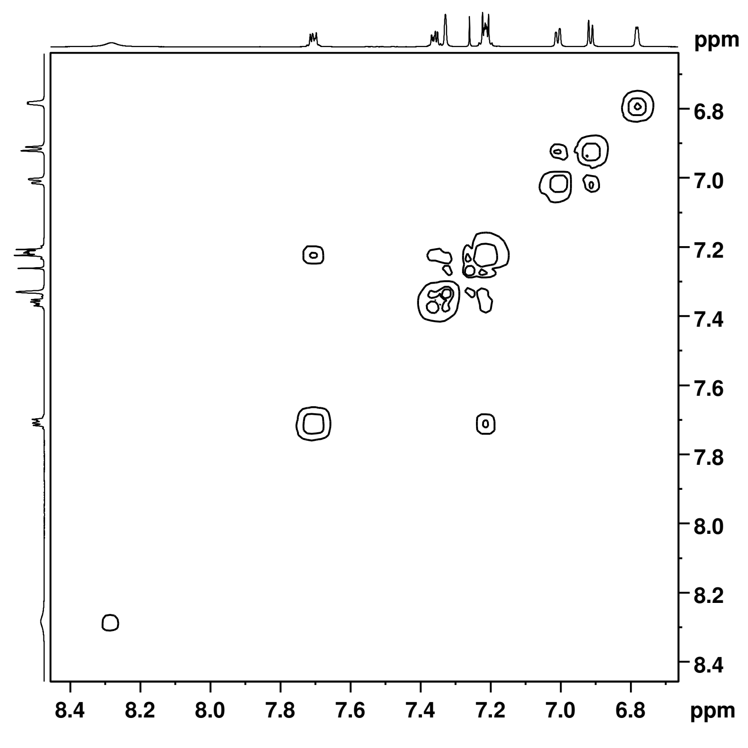

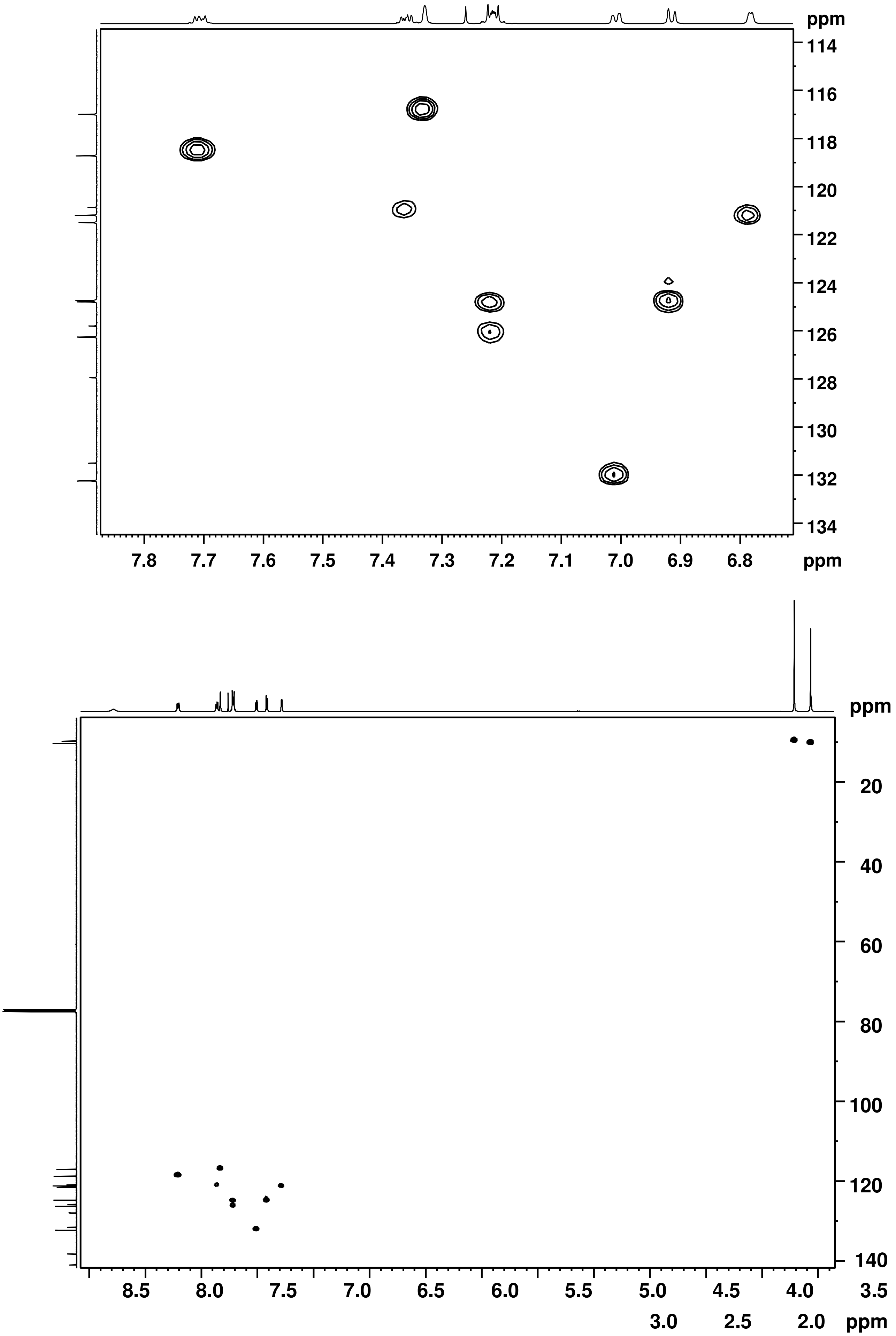

Figure S46. 500 MHz HSQC NMR spectrum of fulvene **16a** in $CDCl_3$.

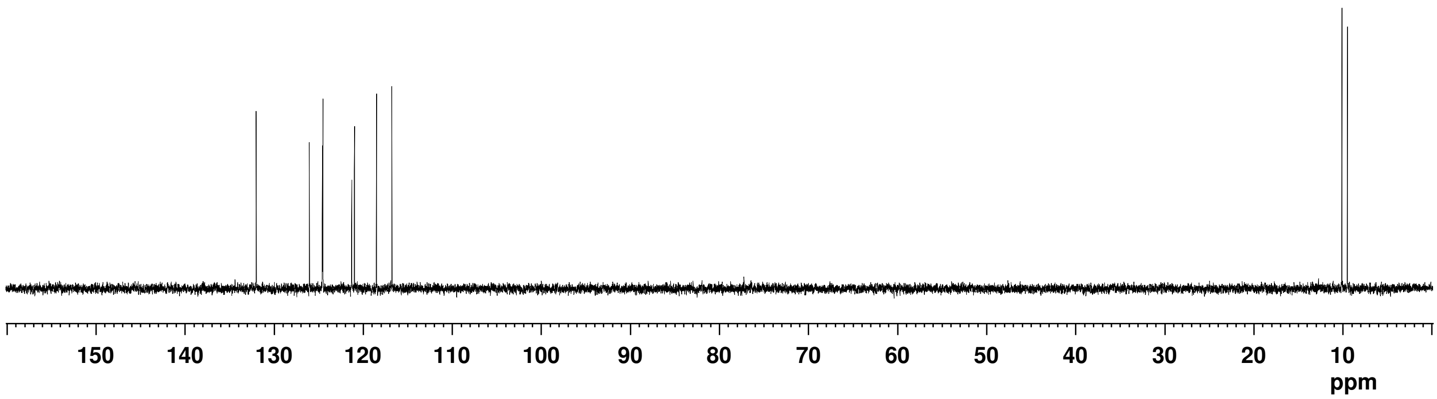

Figure S47. DEPT-135 NMR spectrum of fulvene **16a** in $CDCl_3$.

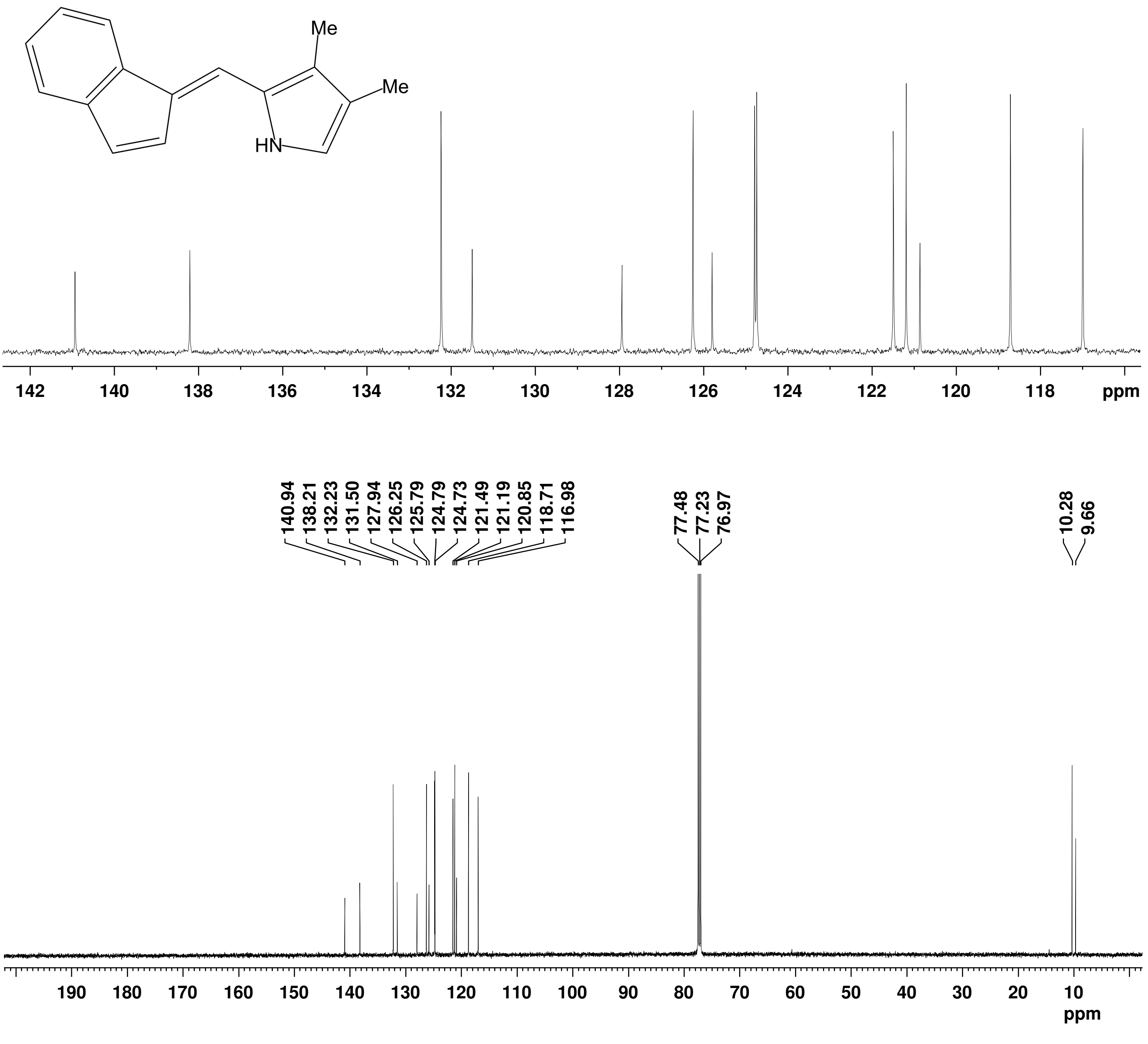

Figure S48. 125 MHz carbon-13 NMR spectrum of fulvene **16a** in $CDCl_3$.

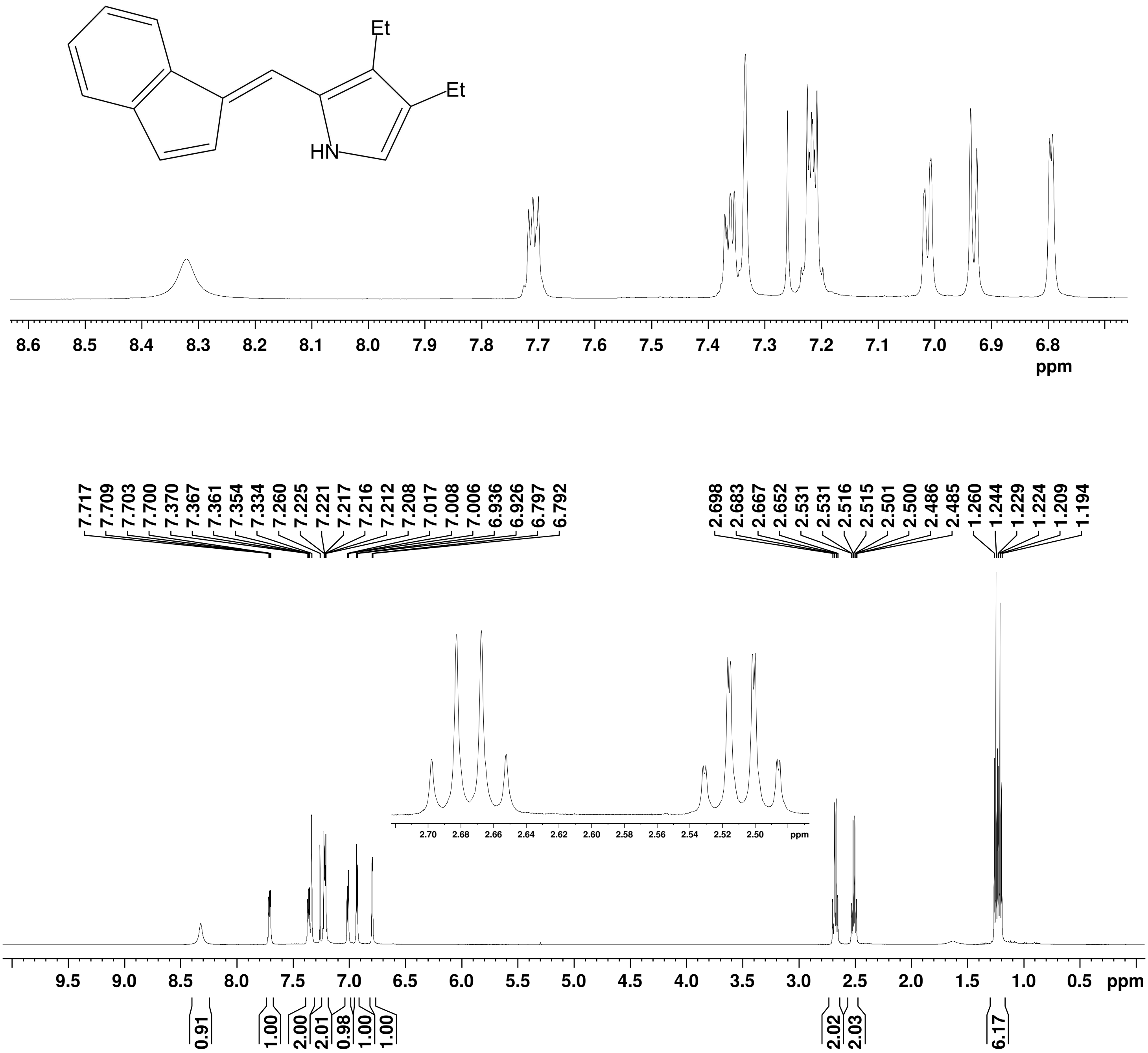

Figure S49. 500 MHz proton NMR spectrum of fulvene **16b** in $CDCl_3$.

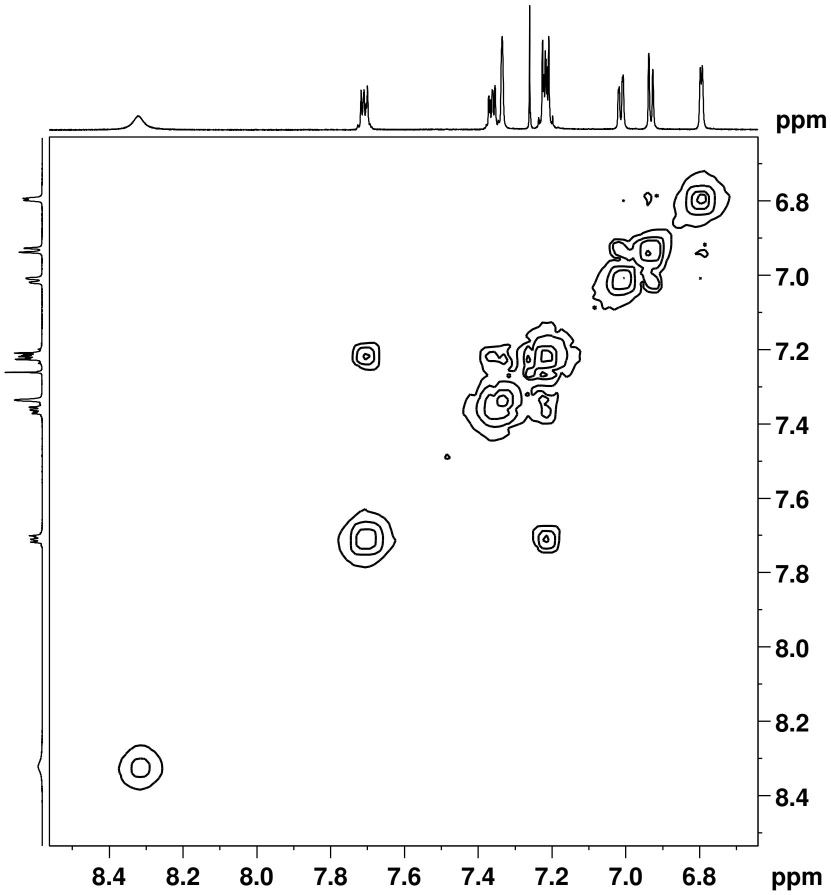

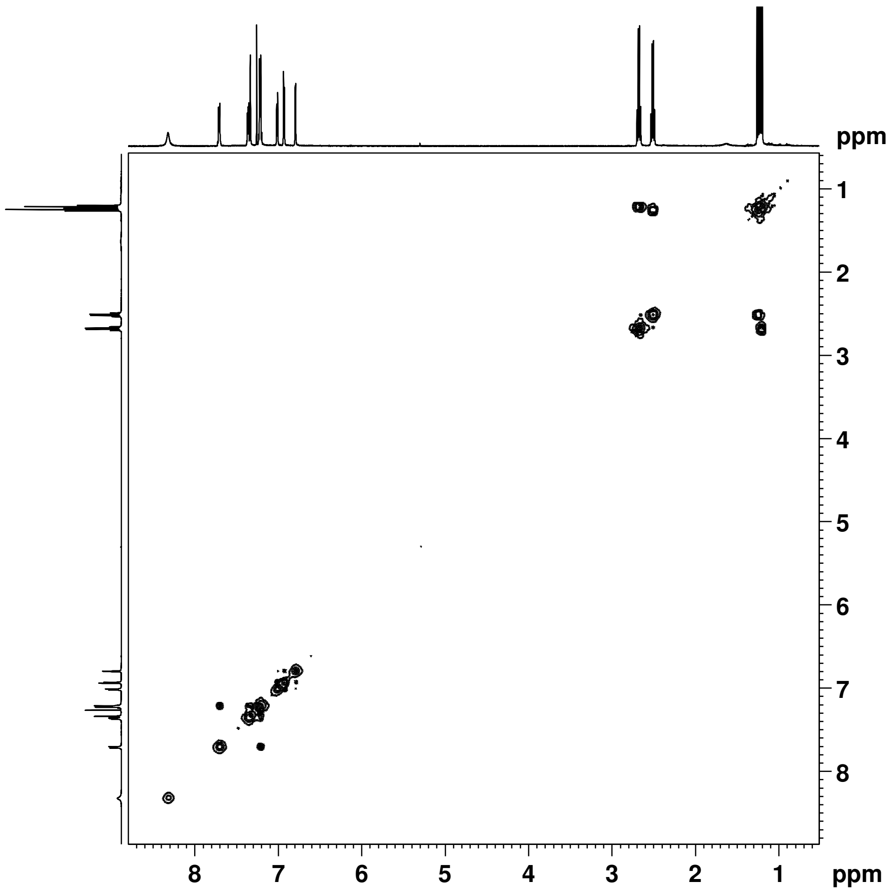

Figure S50. 1H-1H COSY NMR spectrum of fulvene **16b** in $CDCl_3$.

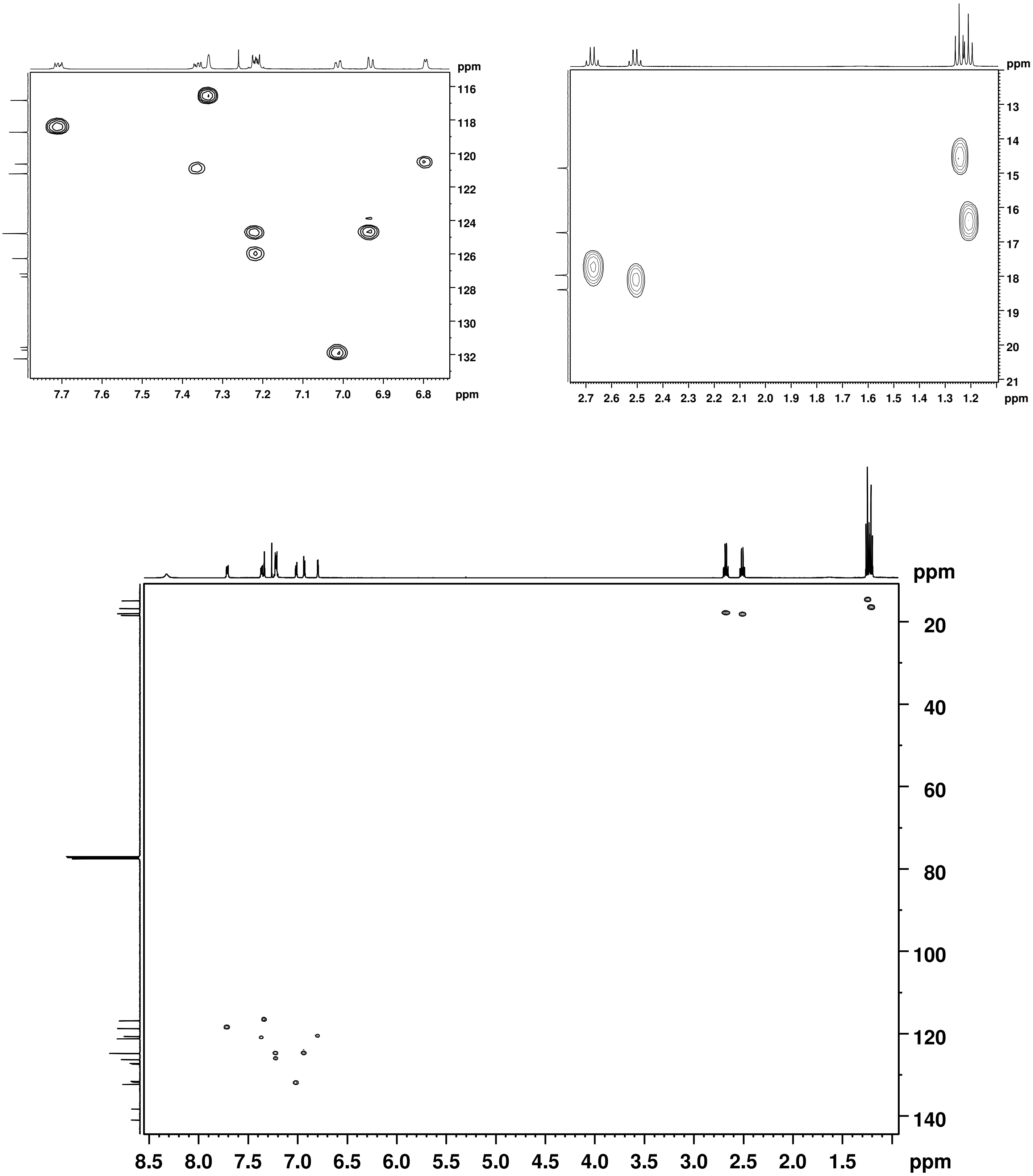

Figure S51. HSQC NMR spectrum of fulvene **16b** in $CDCl_3$.

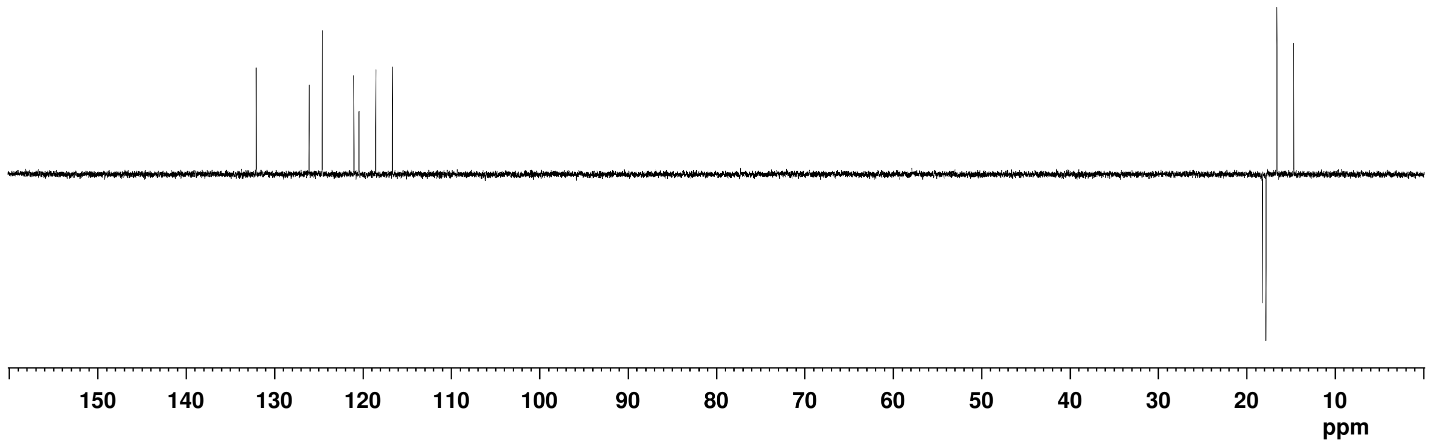

Figure S52. DEPT-135 NMR spectrum of fulvene **16b** in $CDCl_3$.

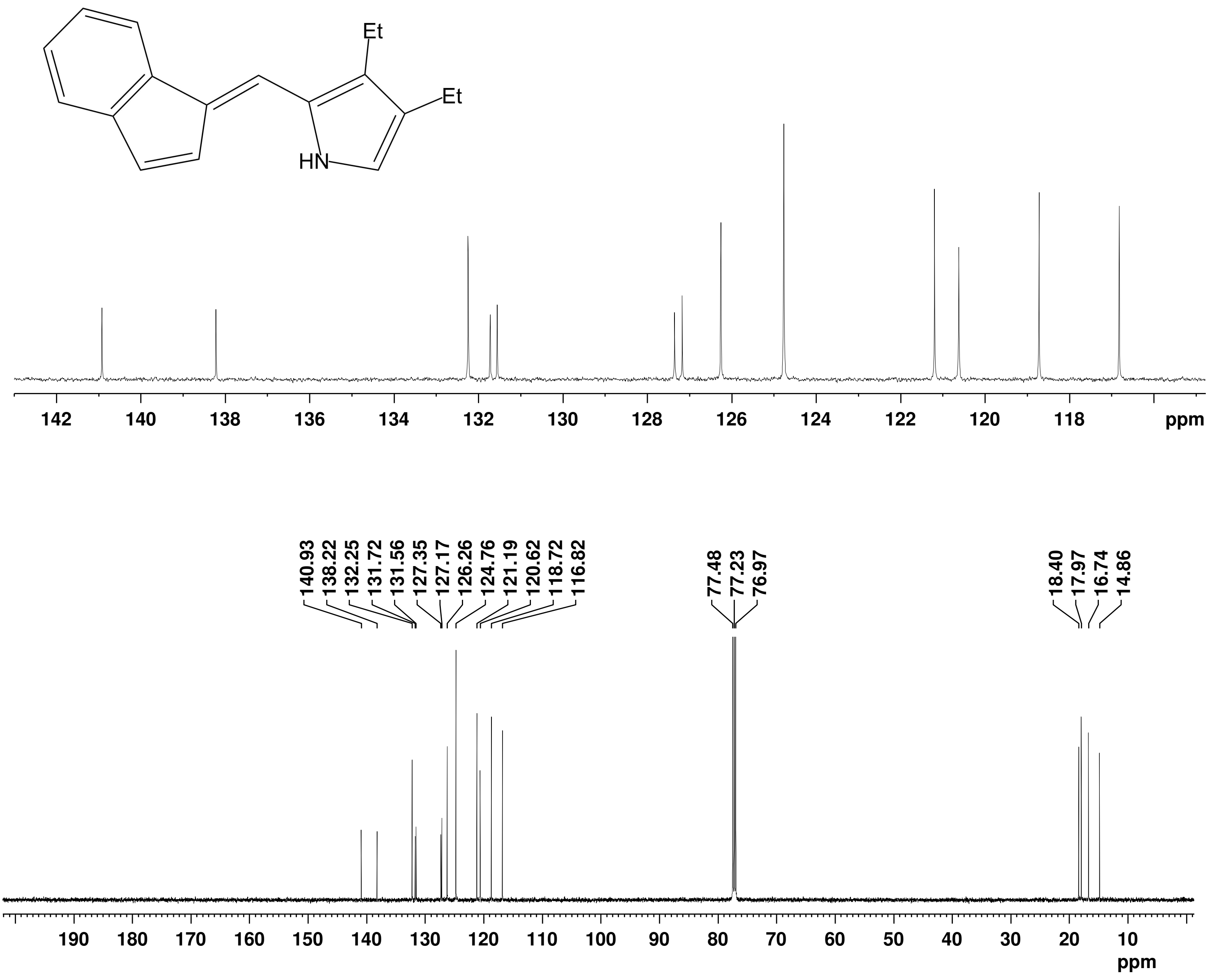

Figure S53. 125 MHz carbon-13 NMR spectrum of fulvene **16b** in $CDCl_3$.

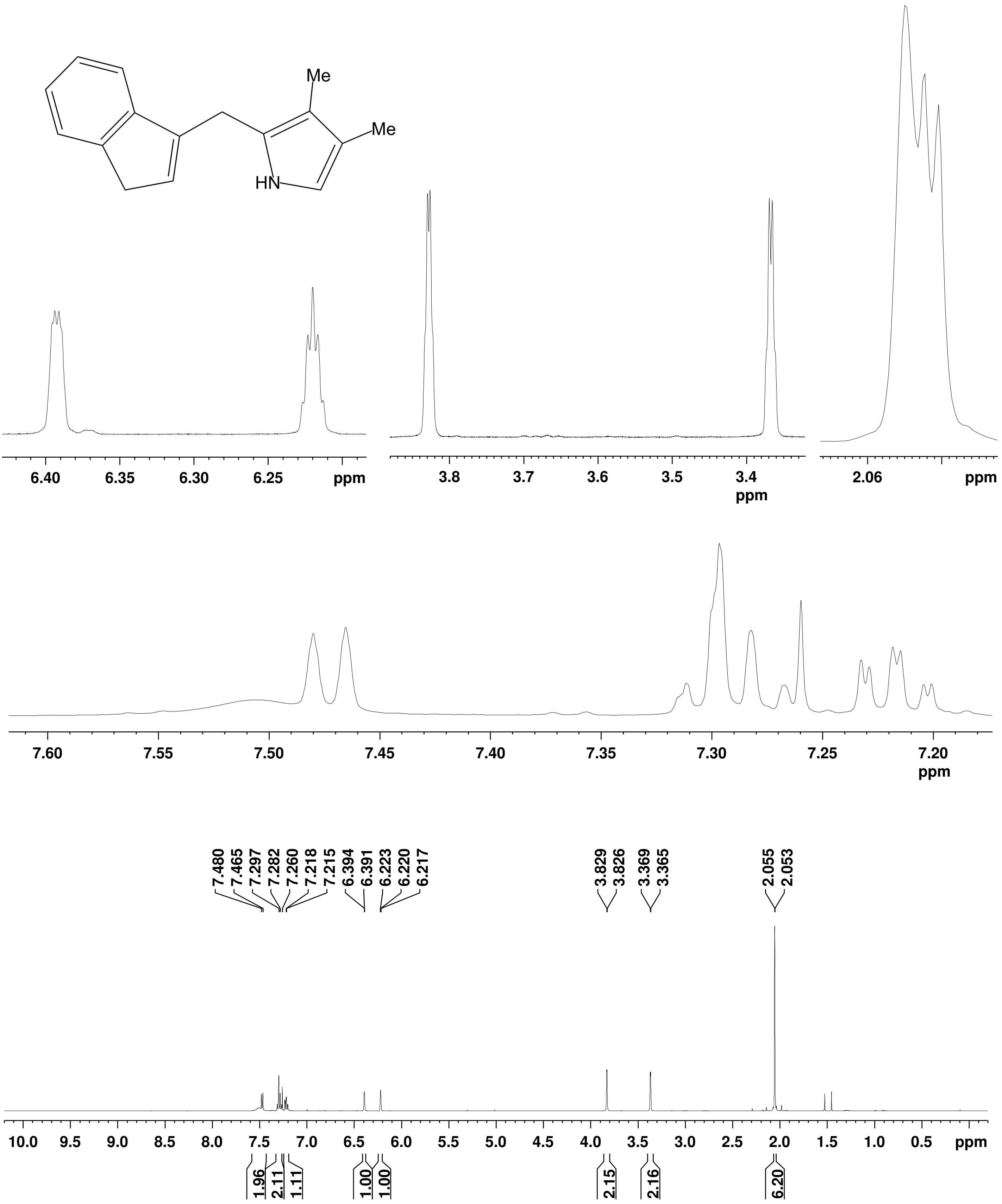

Figure S54. 500 MHz proton NMR spectrum of dihydrofulvene **17a** in $CDCl_3$.

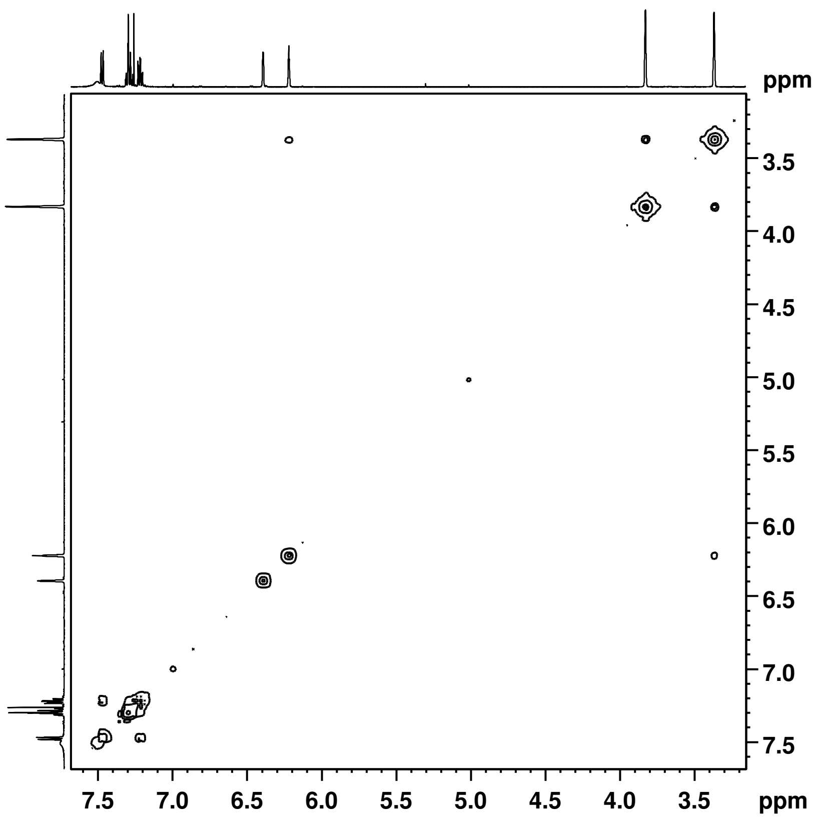

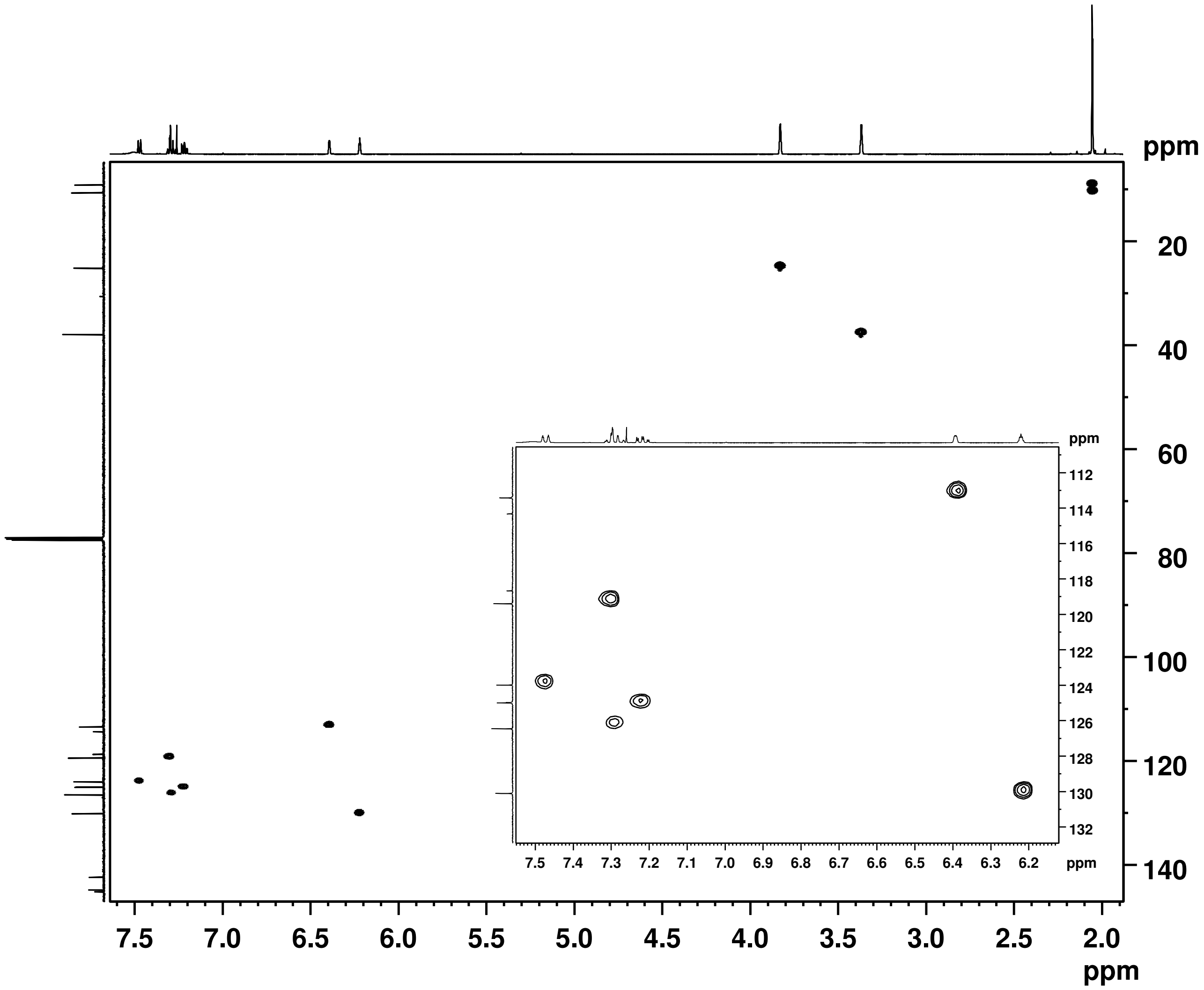

Figure S55. ^{1}H-^{1}H COSY (top) and HSQC (bottom) NMR spectra of **17a** in $CDCl_3$.

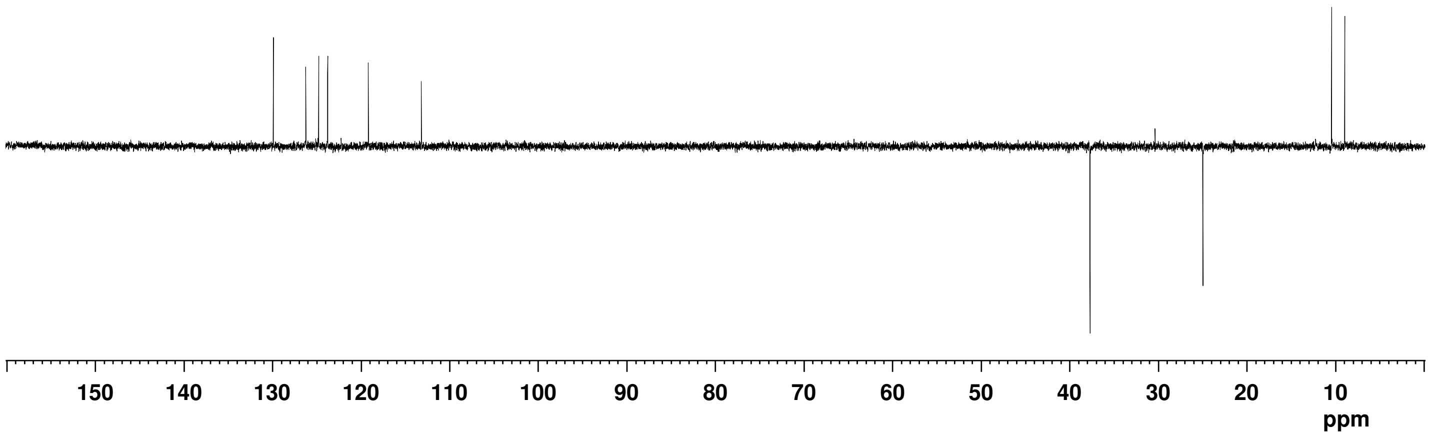

Figure S56. DEPT-135 NMR spectrum of dihydrofulvene **17a** in $CDCl_3$.

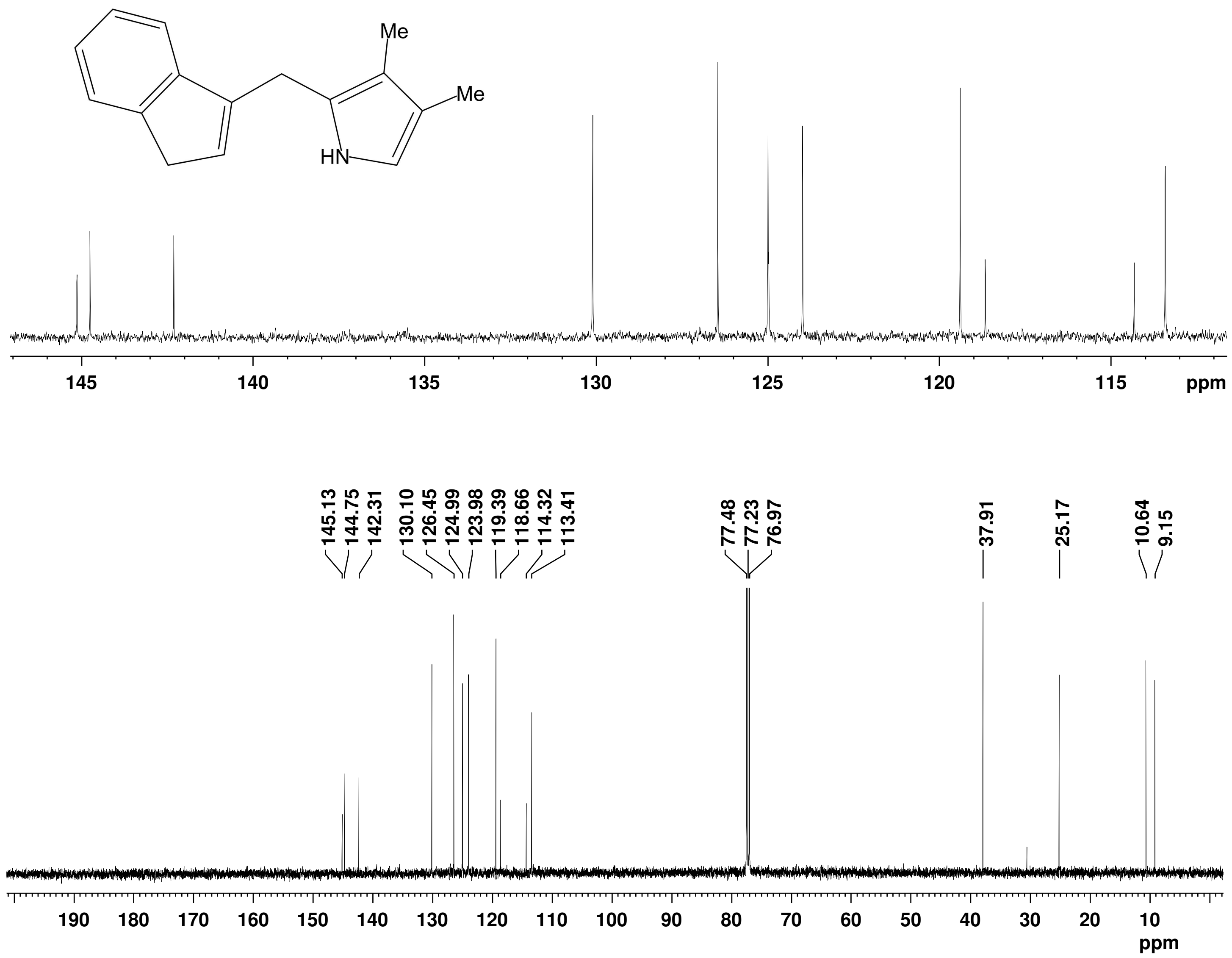

Figure S57. 125 MHz carbon-13 NMR spectrum of dihydrofulvene **17a** in $CDCl_3$.

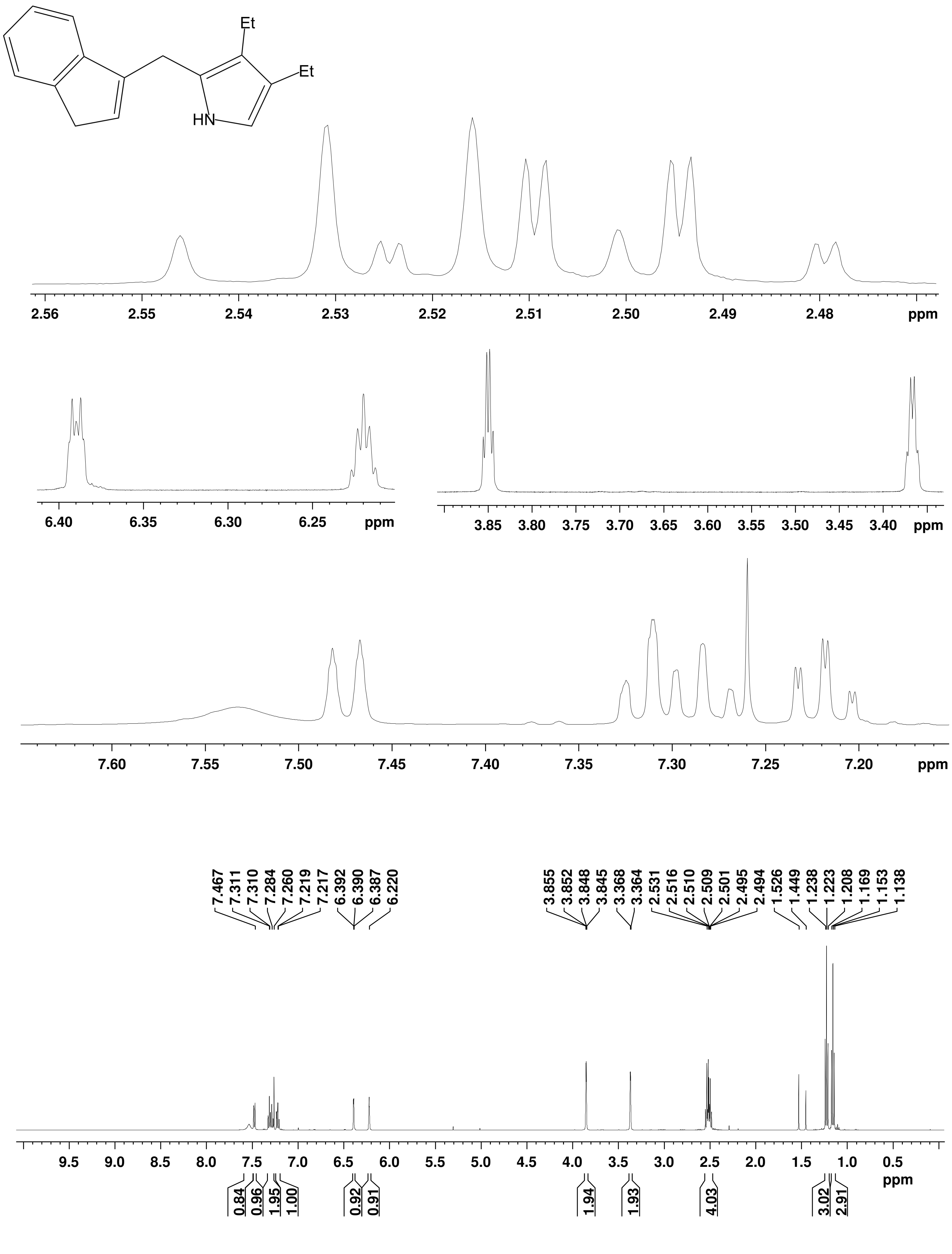

Figure S58. 500 MHz proton NMR spectrum of dihydrofulvene **17b** in $CDCl_3$.

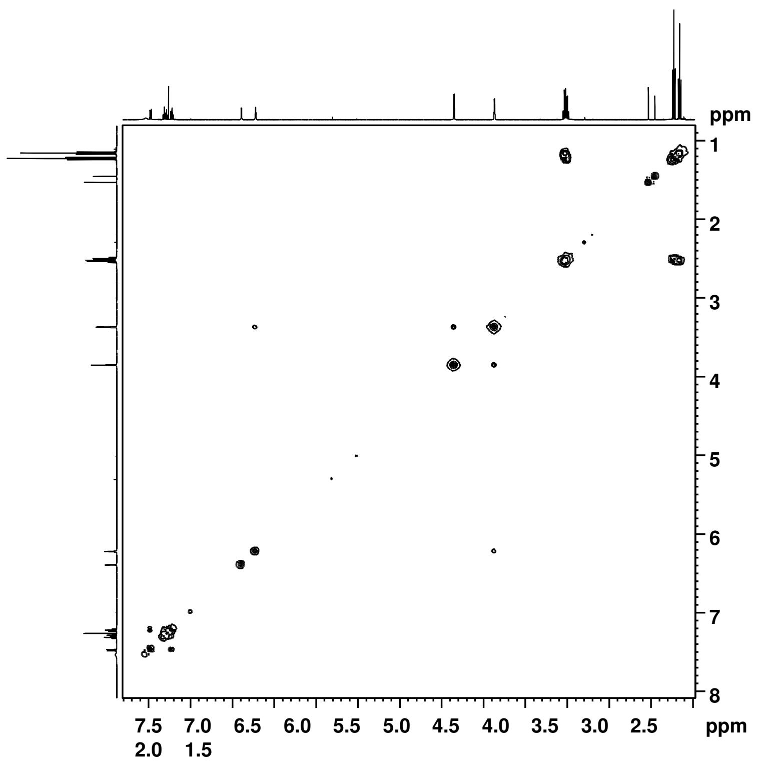

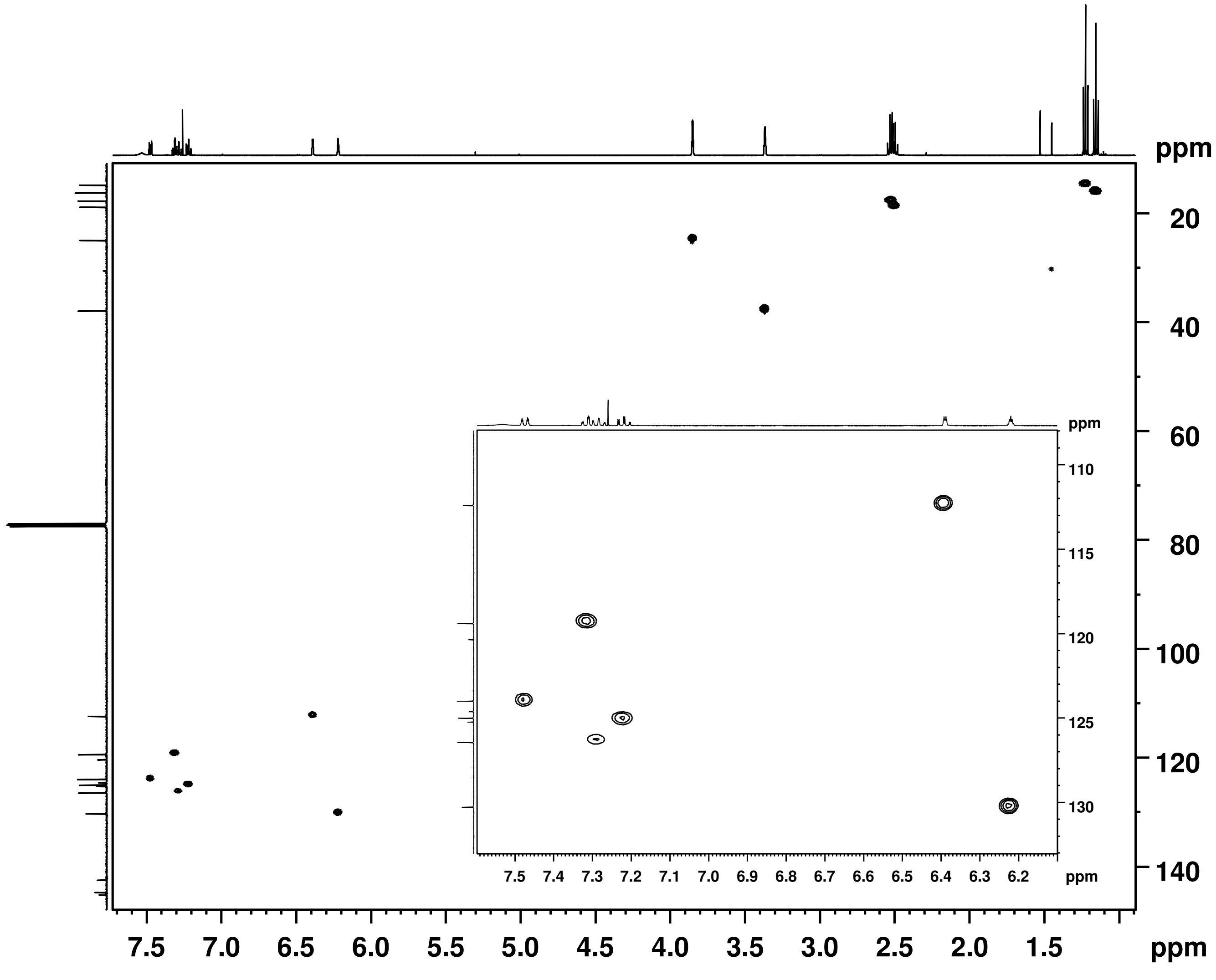

Figure S59. ^{1}H-^{1}H COSY (top) and HSQC (bottom) NMR spectra of dihydrofulvene **17b** in $CDCl_3$.

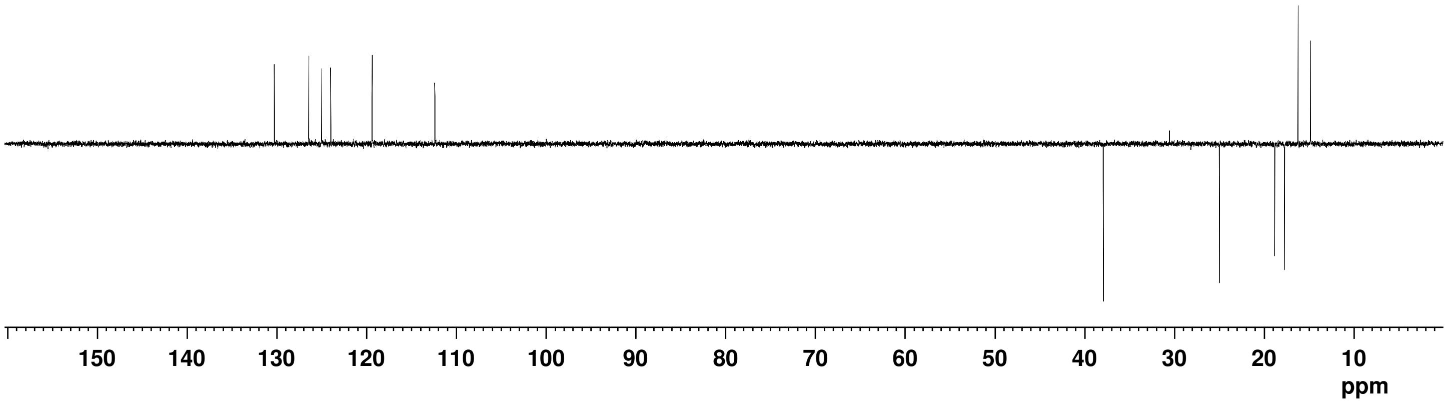

Figure S60. DEPT-135 NMR spectrum of dihydrofulvene **17b** in $CDCl_3$.

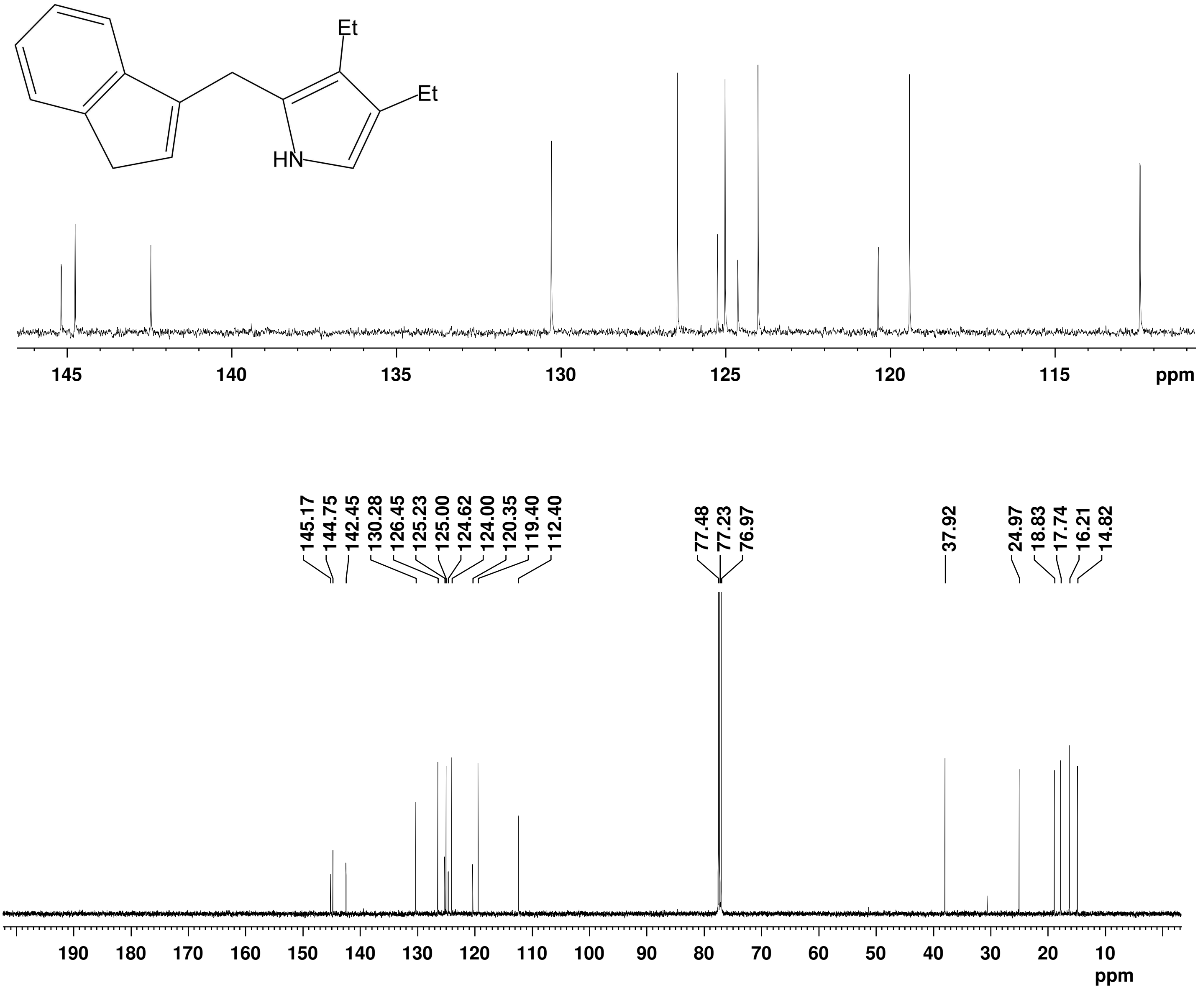

Figure S61. 125 MHz carbon-13 NMR spectrum of dihydrofulvene **17b** in $CDCl_3$.

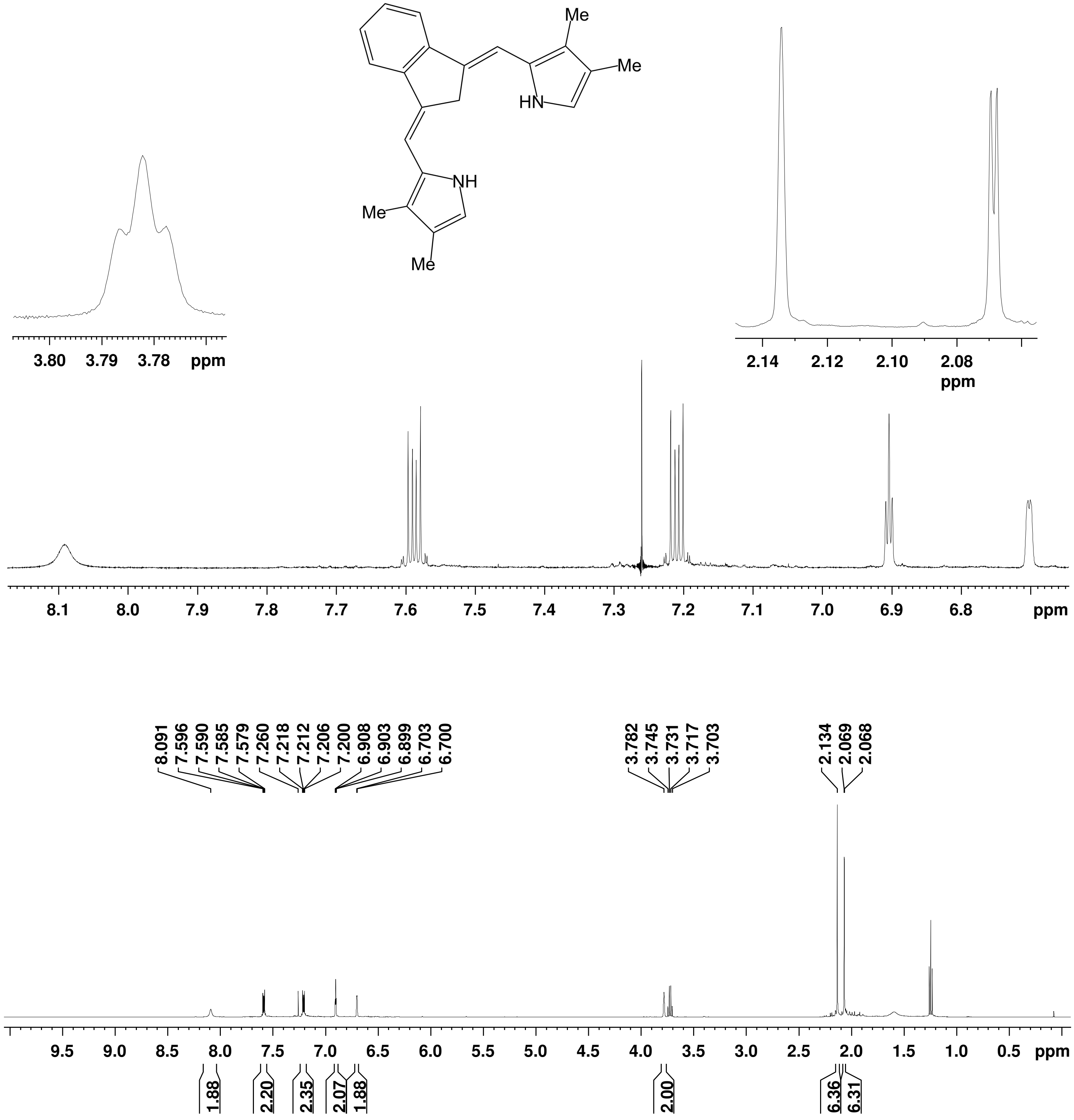

Figure S62. 500 MHz proton NMR spectrum of carbatripyrrin **14a** in $CDCl_3$.

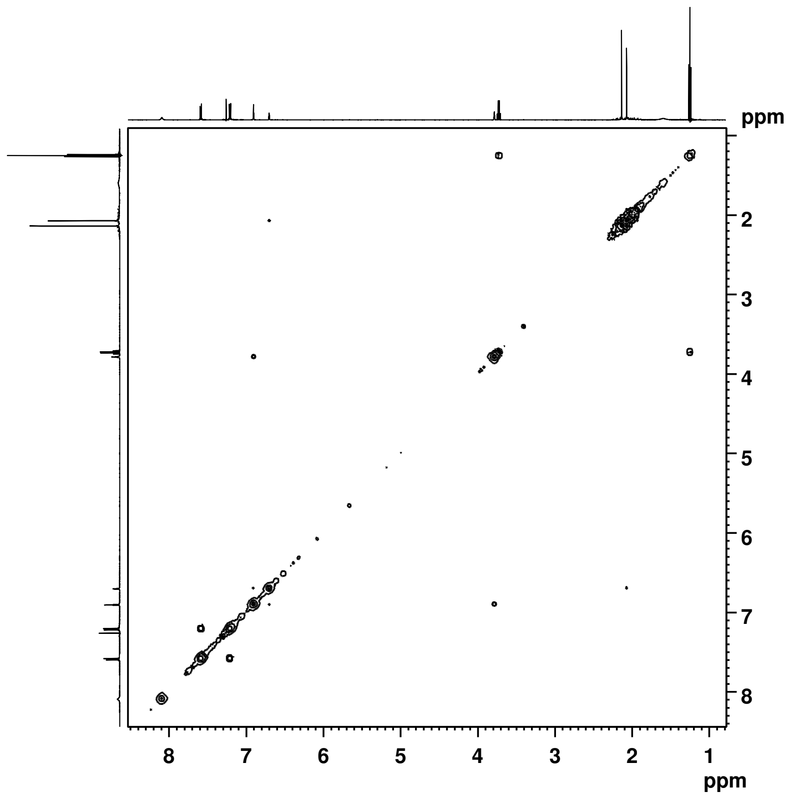

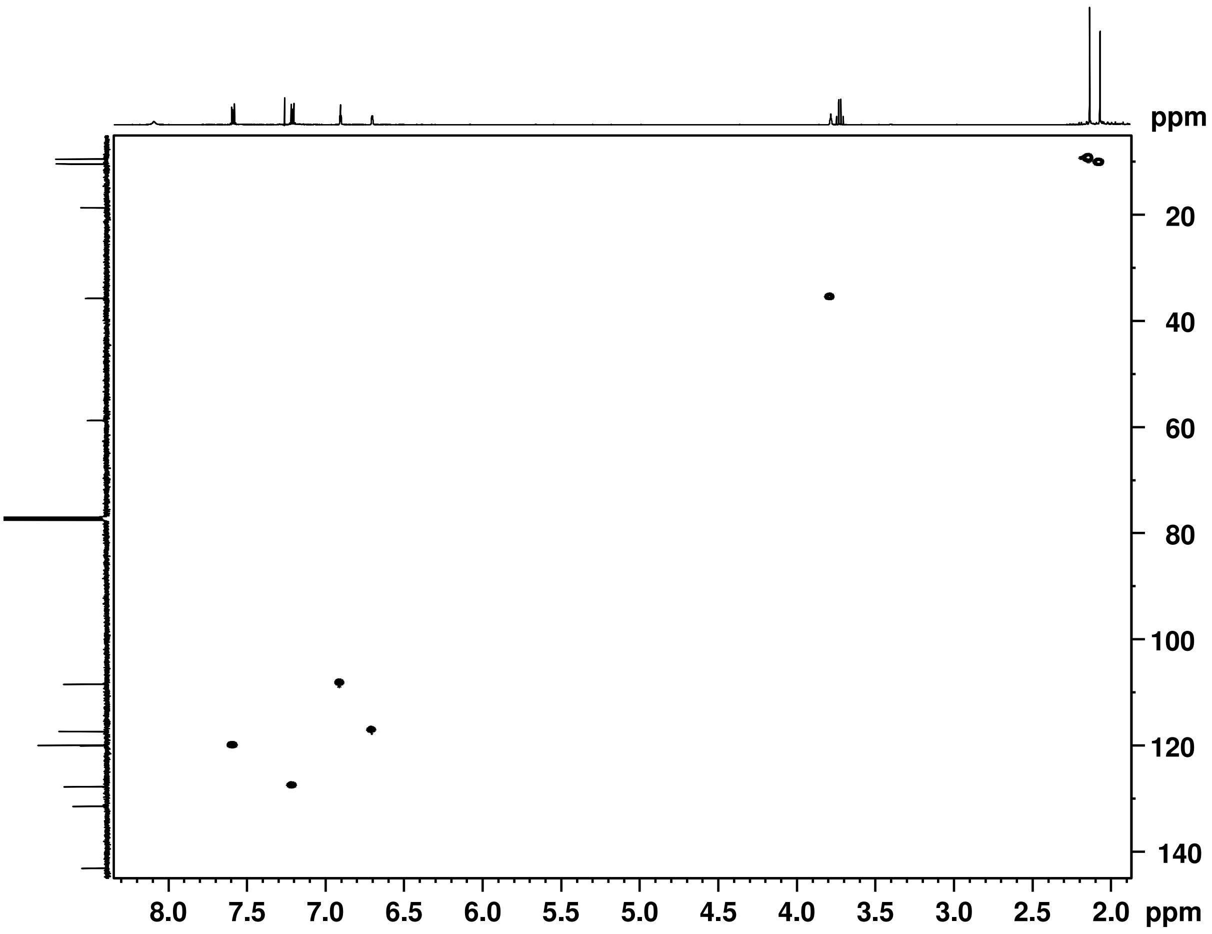

Figure S63. ^{1}H-^{1}H COSY (top) and HSQC (bottom) NMR spectra of carbatripyrrin **14a** in $CDCl_3$.

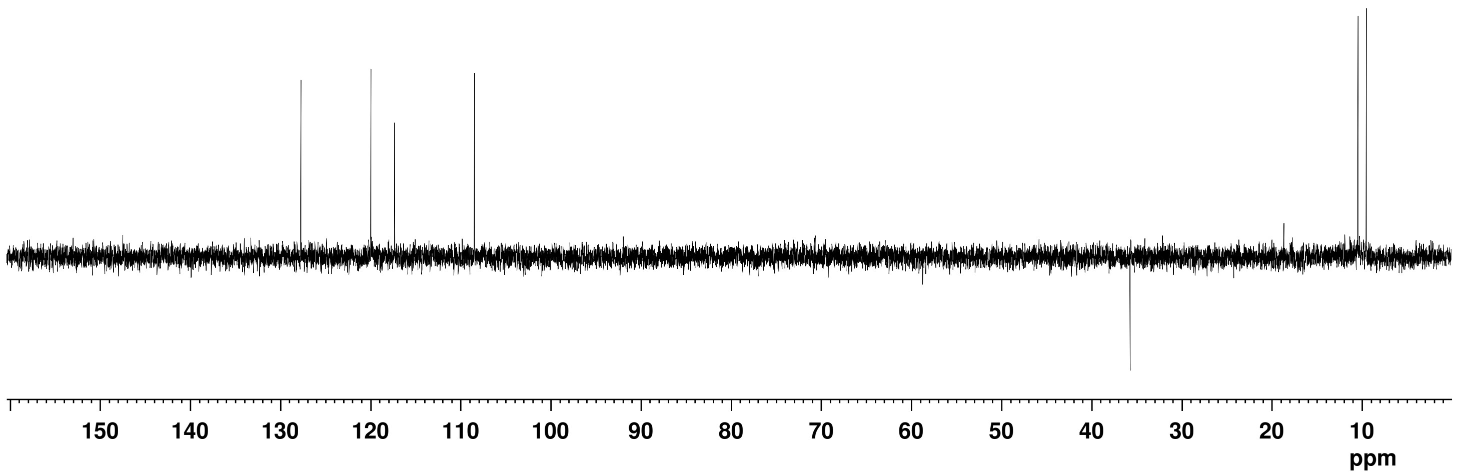

Figure S64. DEPT-135 NMR spectrum of carbatripyrrin **14a** in $CDCl_3$.

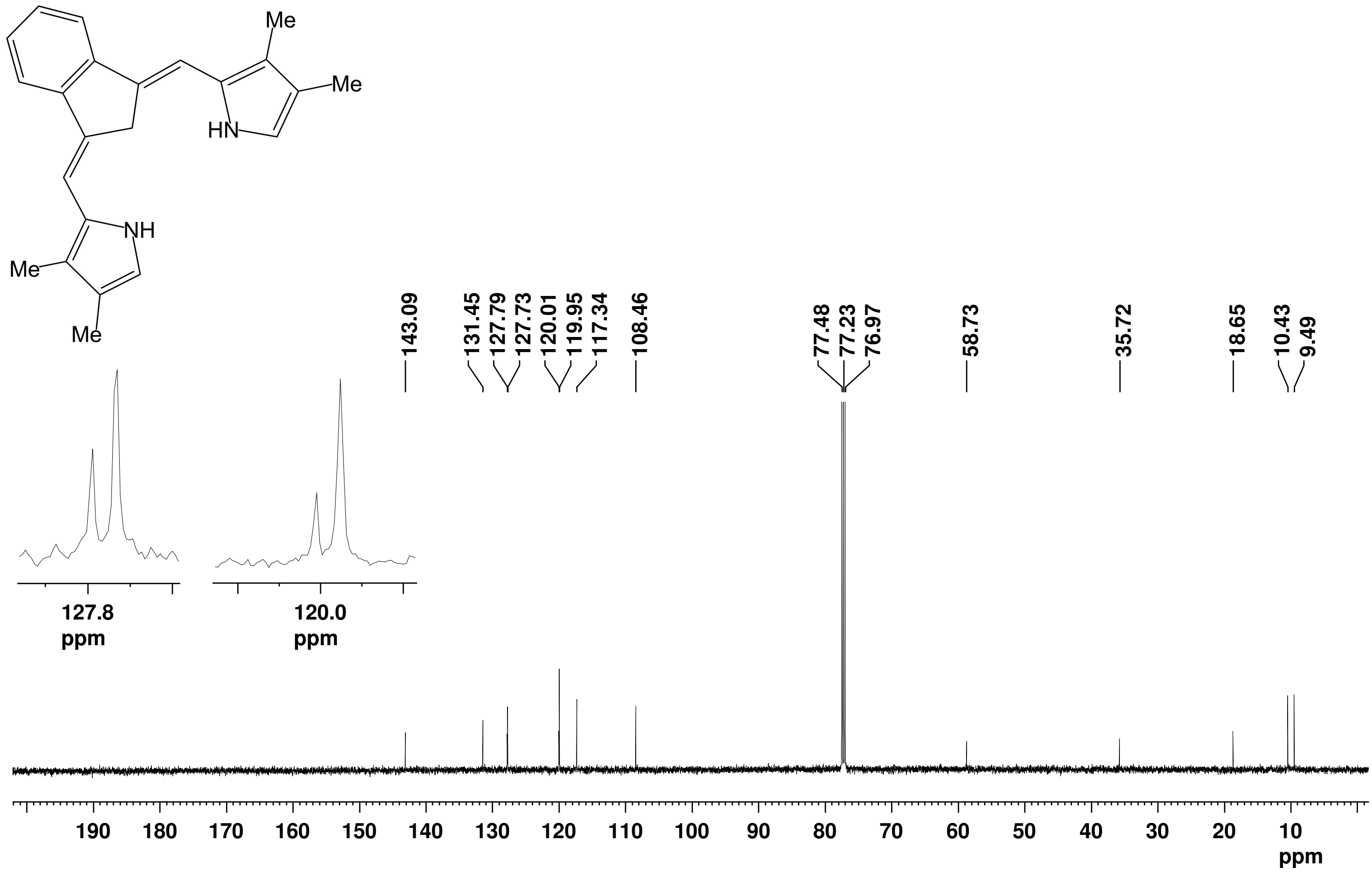

Figure S65. 125 MHz carbon-13 NMR spectrum of carbatripyrrin **14a** in $CDCl_3$.

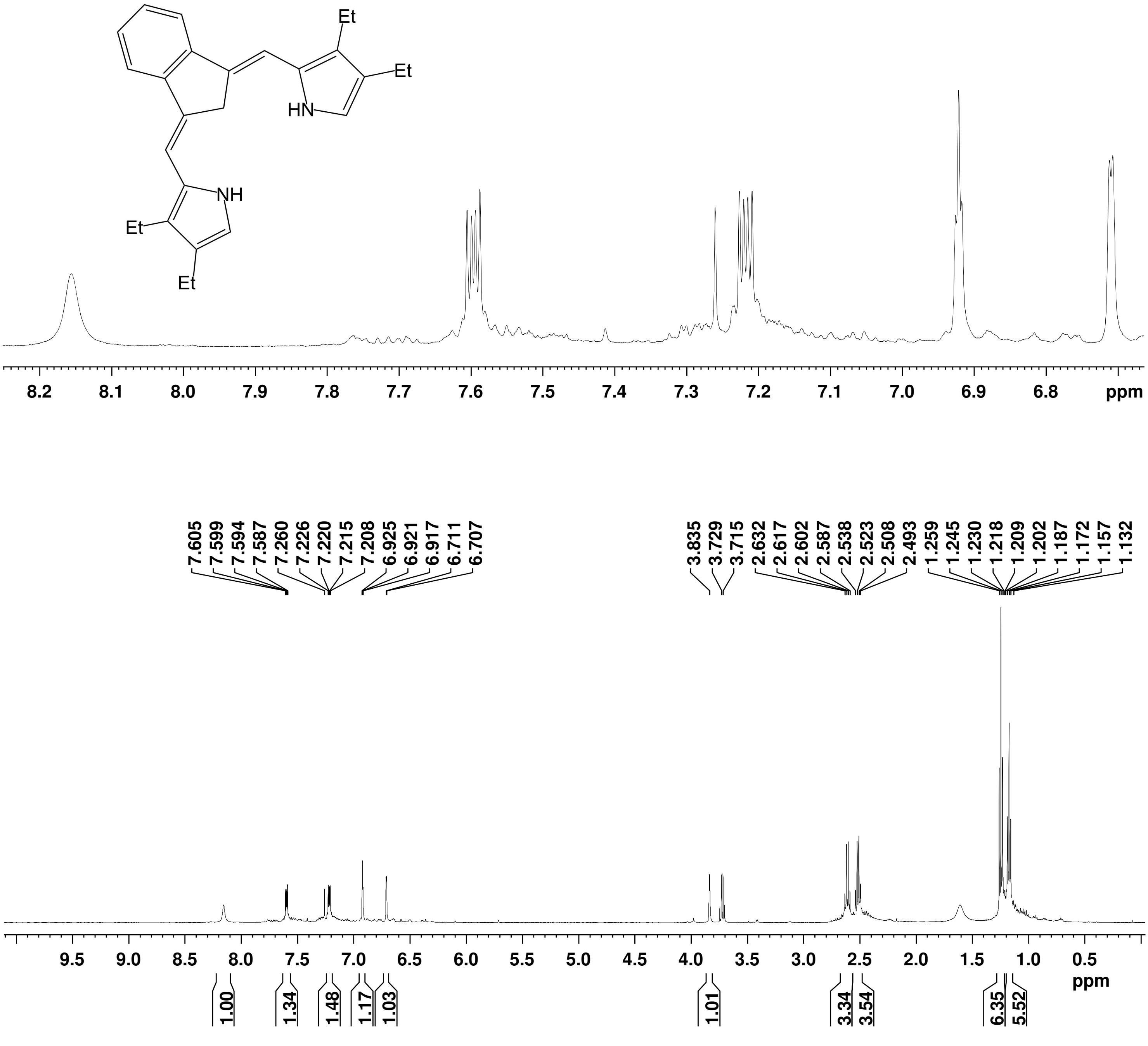

Figure S66. 500 MHz proton NMR spectrum of carbatripyrrin **14b** in $CDCl_3$.

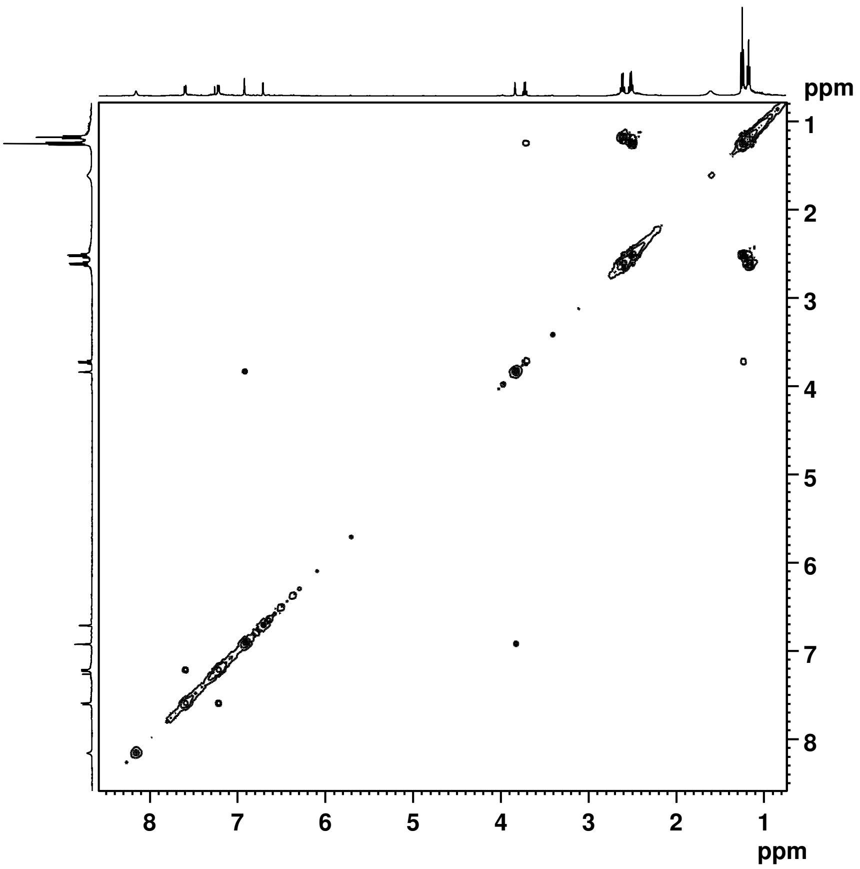

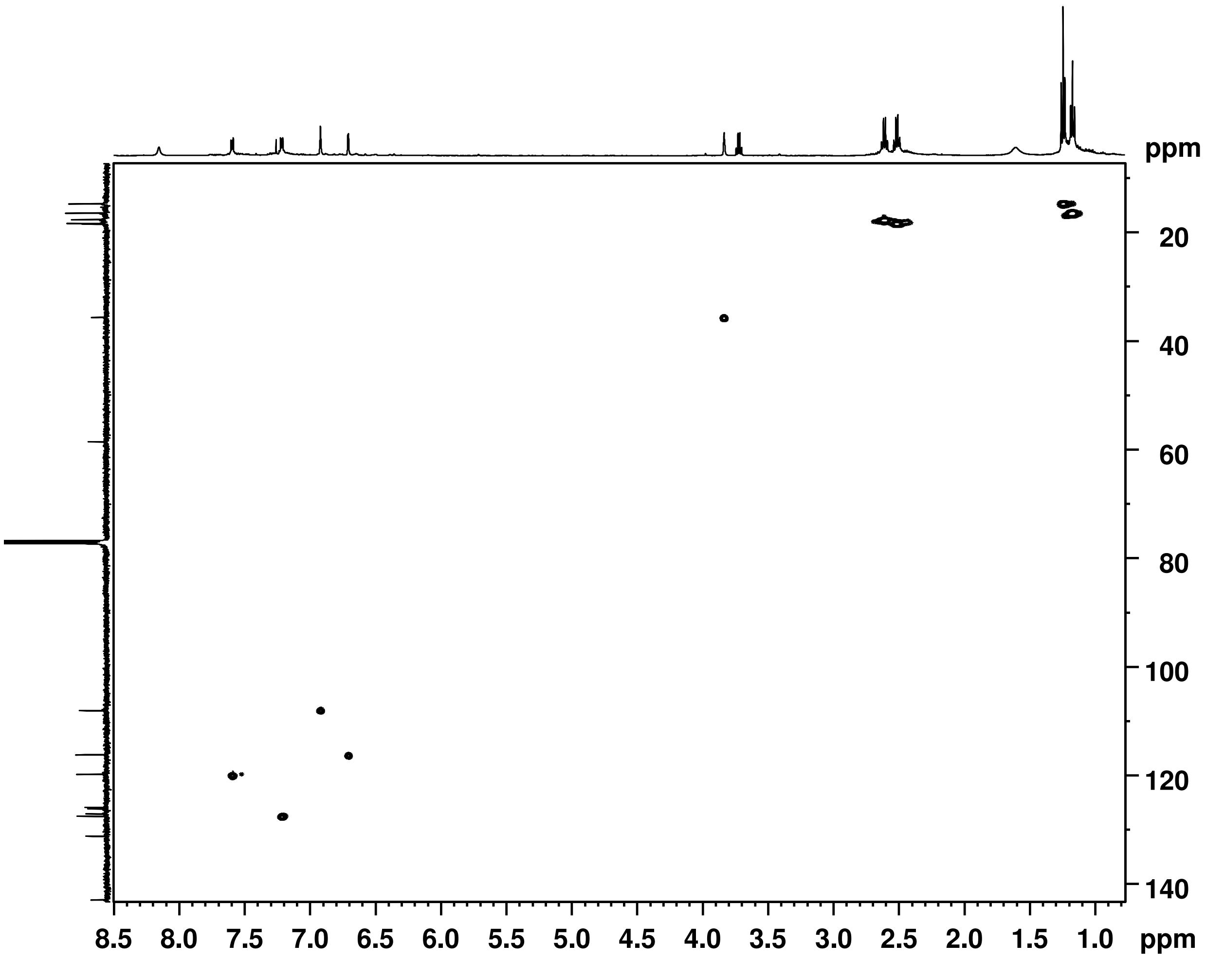

Figure S67. ^{1}H-^{1}H COSY (top) and HSQC (bottom) NMR spectra of carbatripyrrin **14b** in $CDCl_3$.

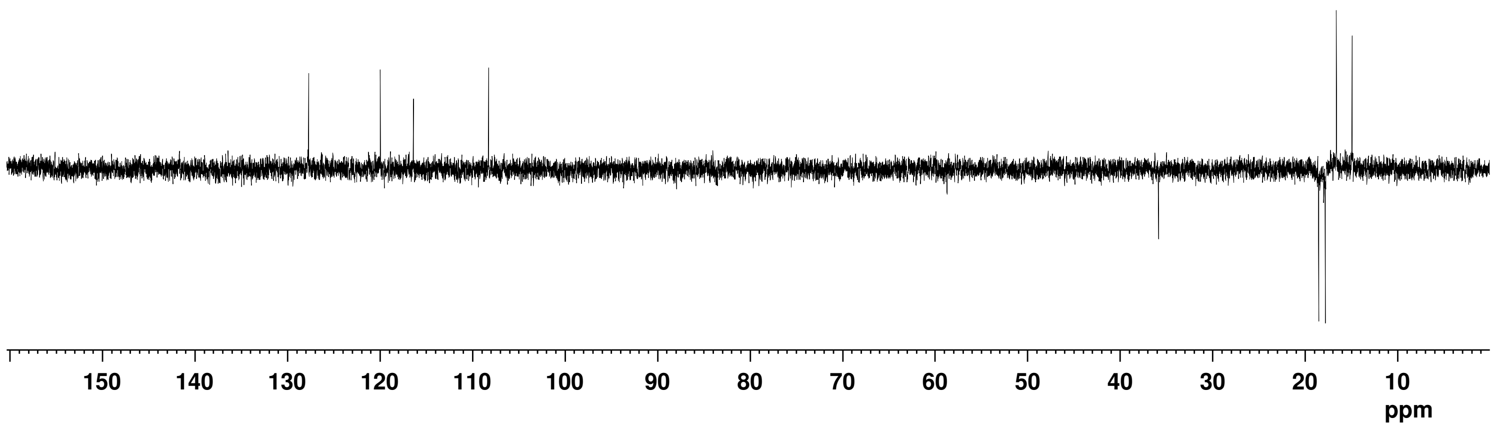

Figure S68. DEPT-135 NMR spectrum of carbatripyrrin **14b** in $CDCl_3$.

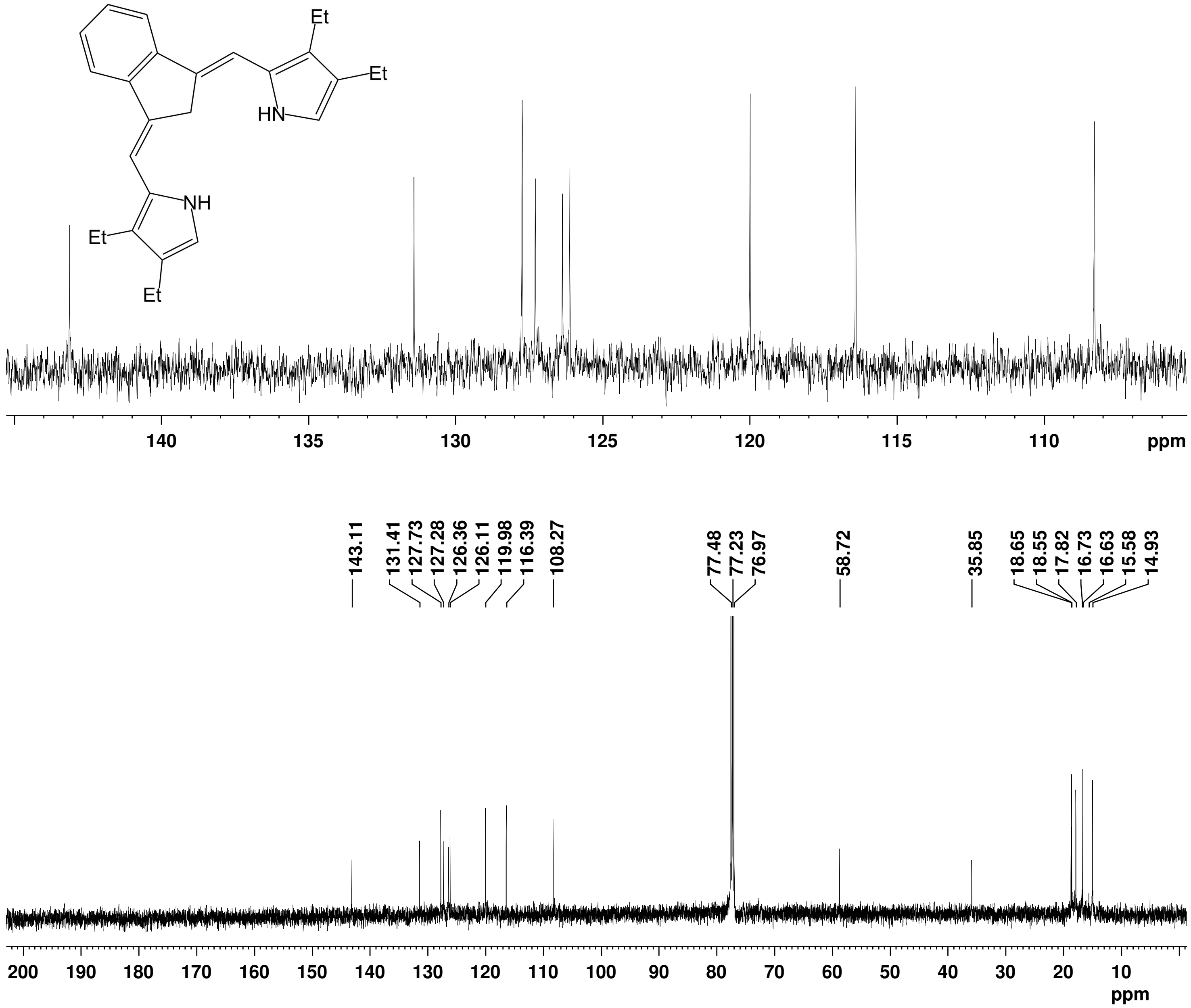

Figure S69. 125 MHz carbon-13 NMR spectrum of carbatripyrrin **14b** in $CDCl_3$.

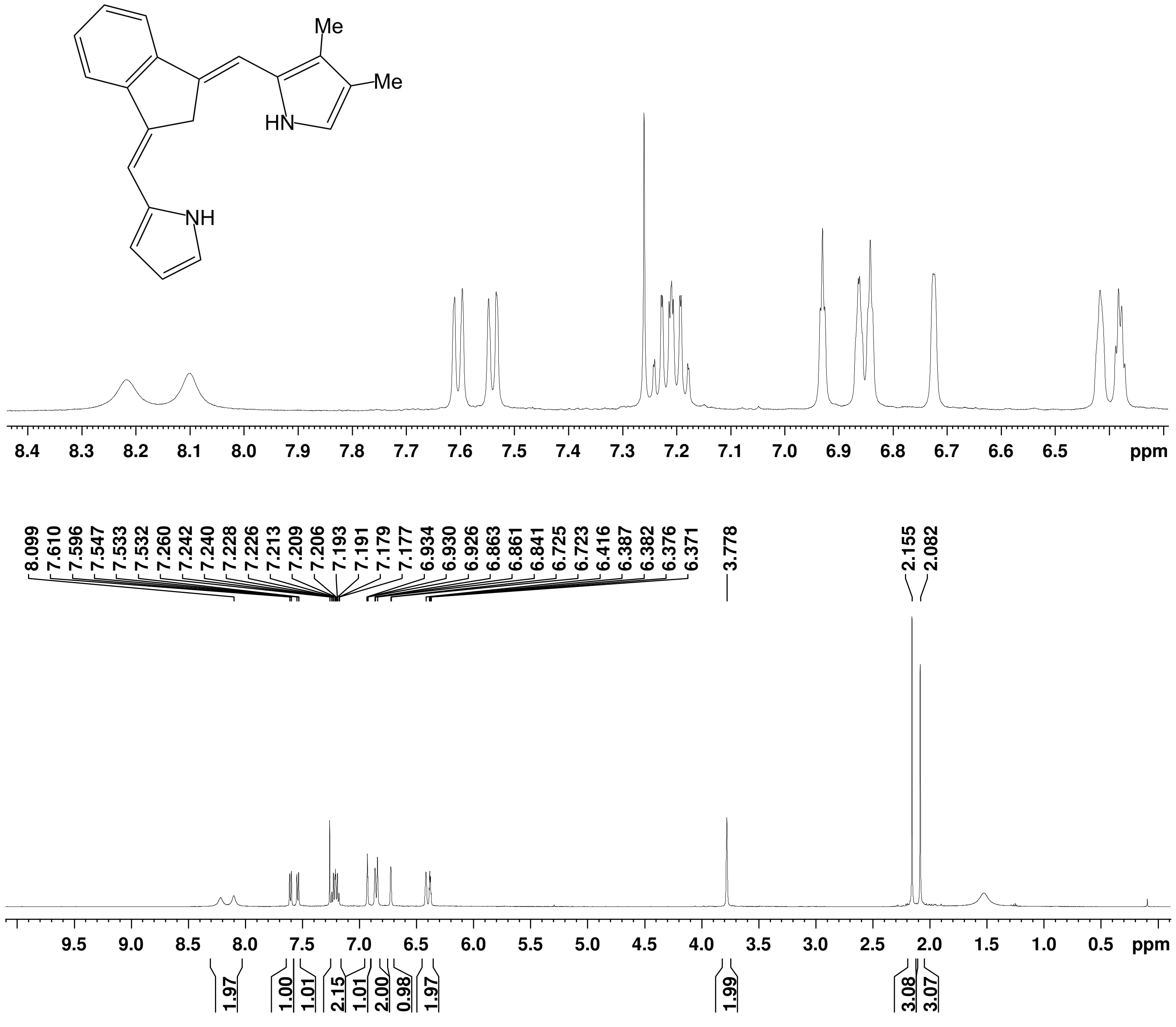

Figure S70. 500 MHz proton NMR spectrum of carbatripyrrin **14c** in $CDCl_3$.

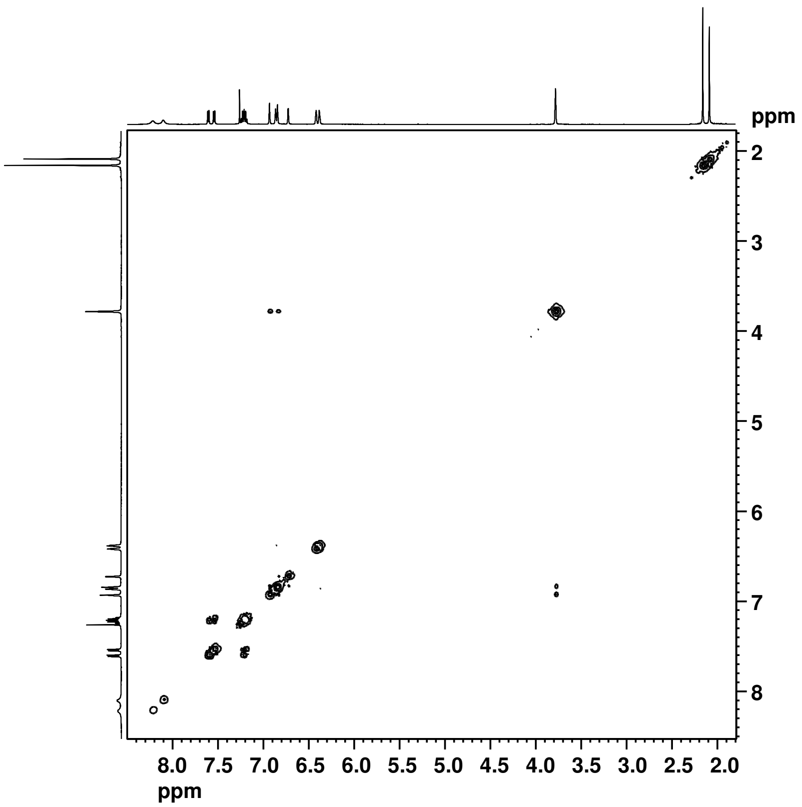

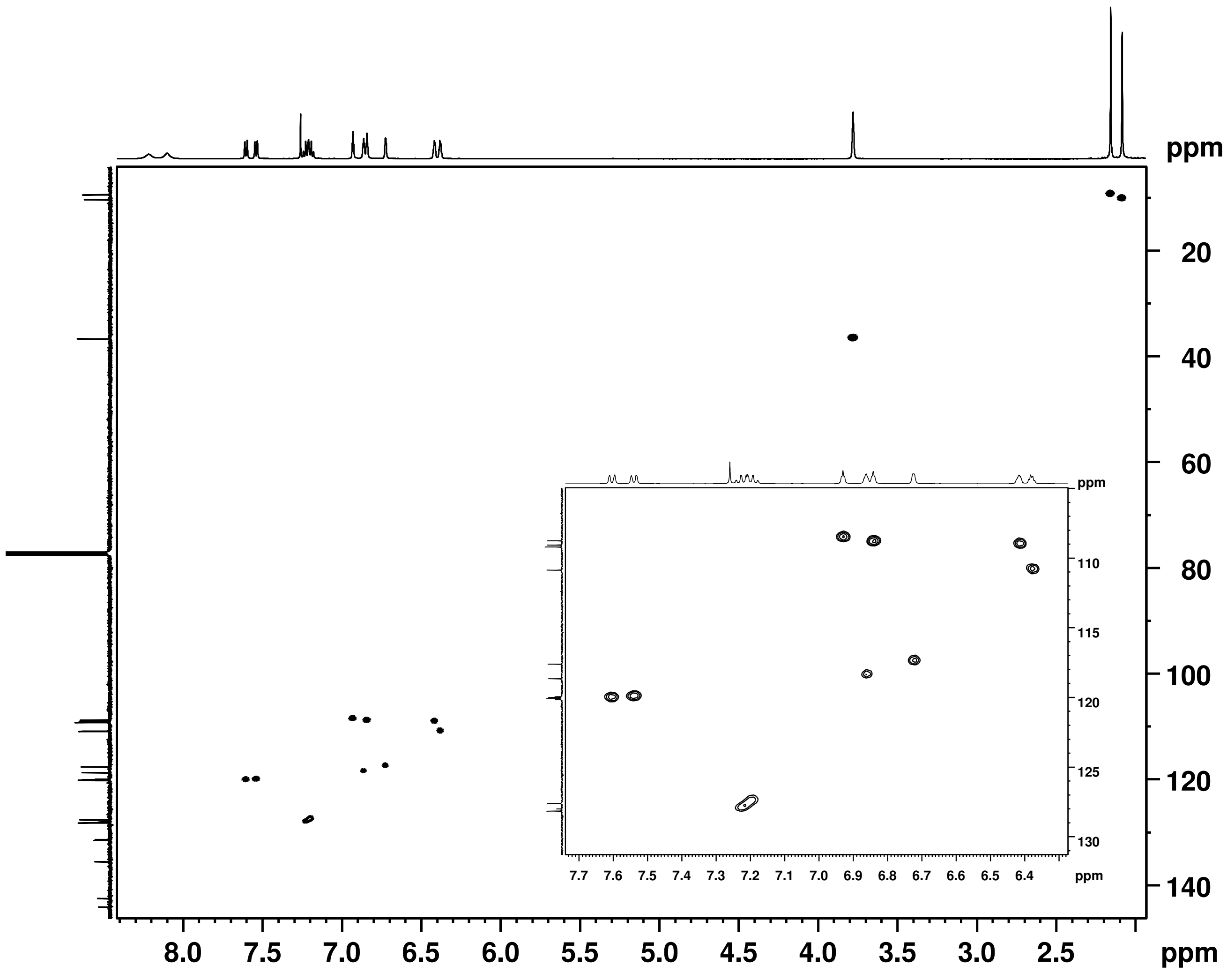

Figure S71. ^{1}H-^{1}H COSY (top) and HSQC (bottom) NMR spectra of carbatripyrrin **14c** in $CDCl_3$.

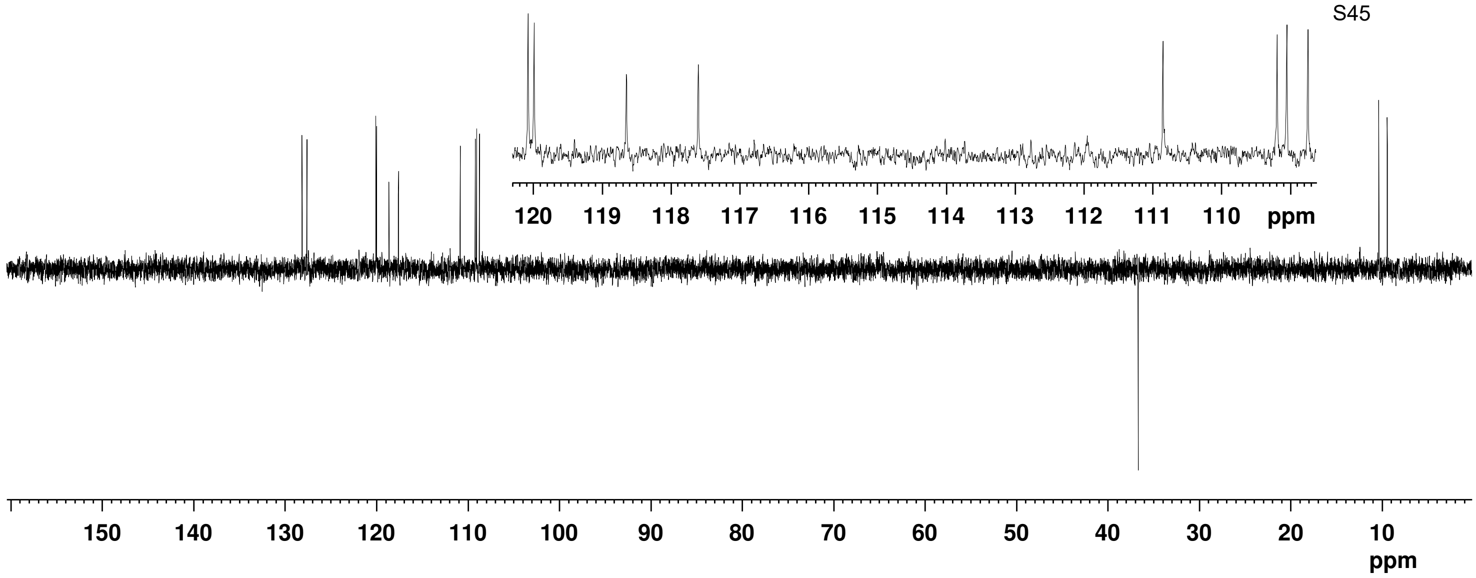

Figure S72. DEPT-135 NMR spectrum of carbatripyrrin **14c** in $CDCl_3$.

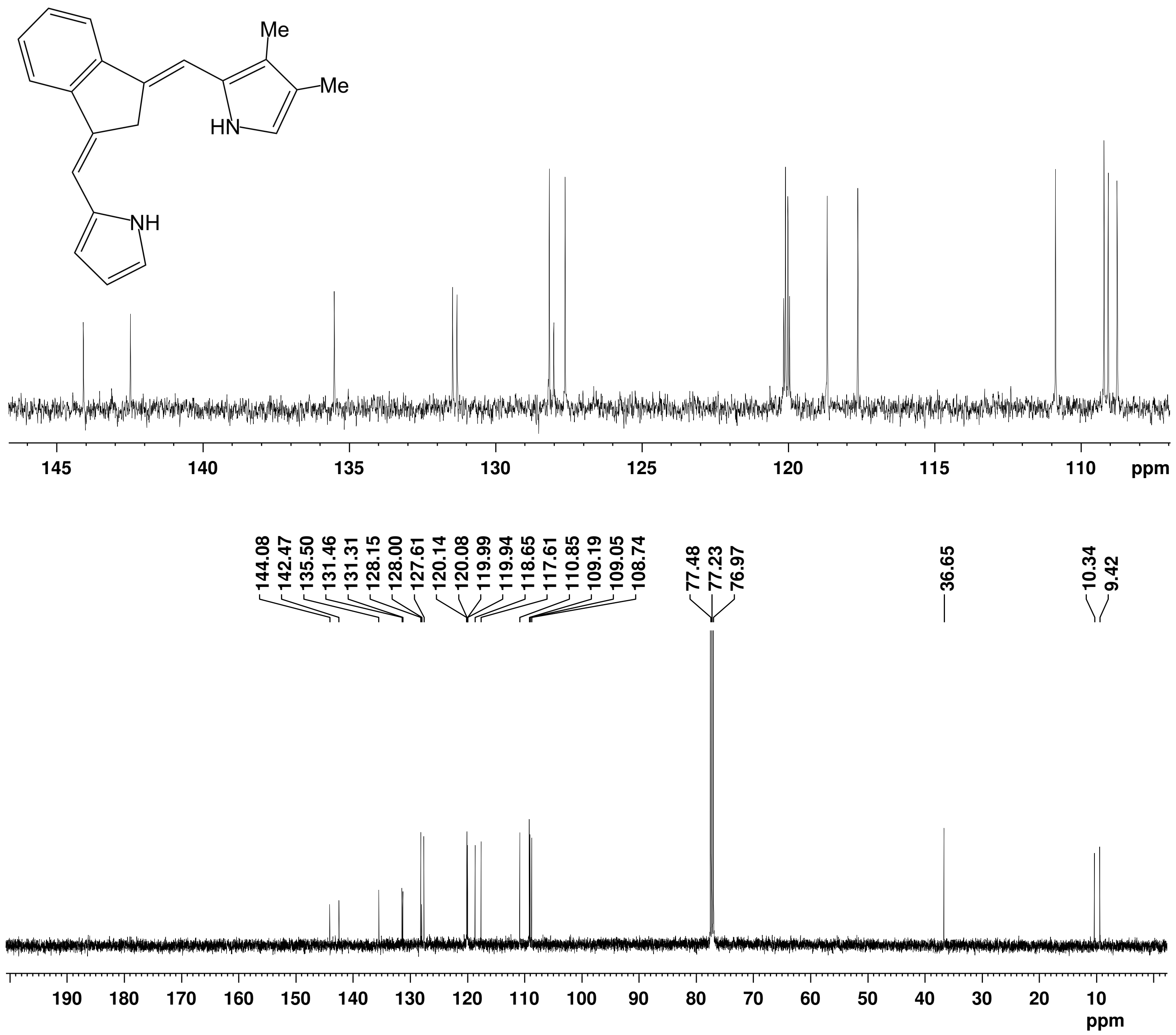

Figure S73. 125 MHz carbon-13 NMR spectrum of carbatripyrrin **14c** in $CDCl_3$.

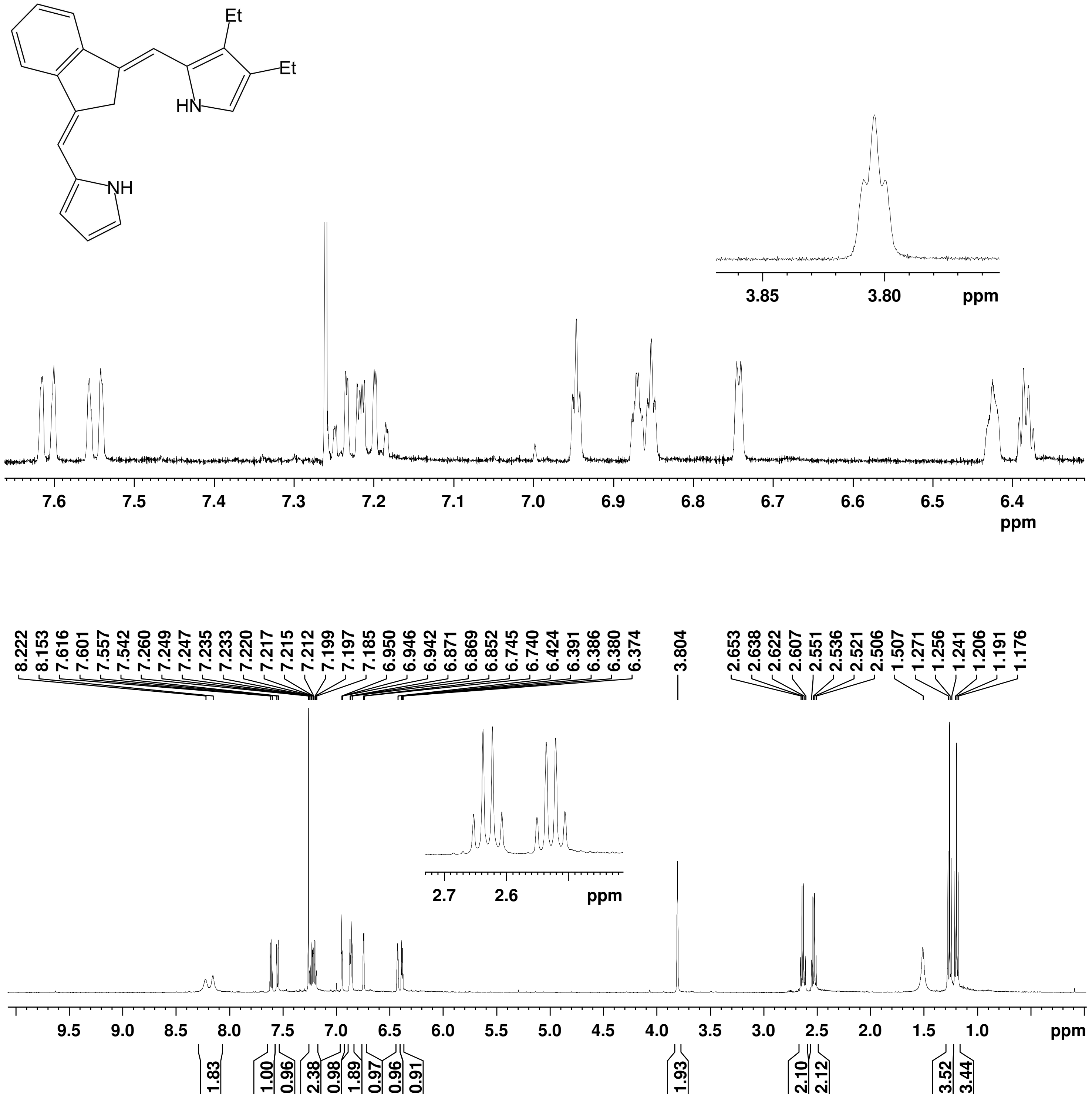

Figure S74. 500 MHz proton NMR spectrum of carbatripyrrin **14d** in $CDCl_3$.

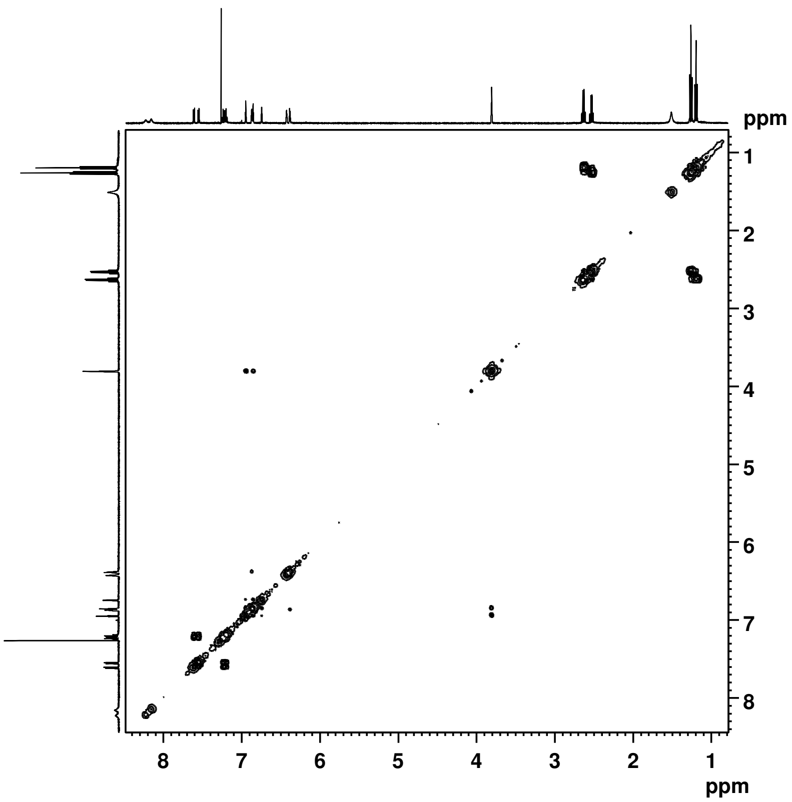

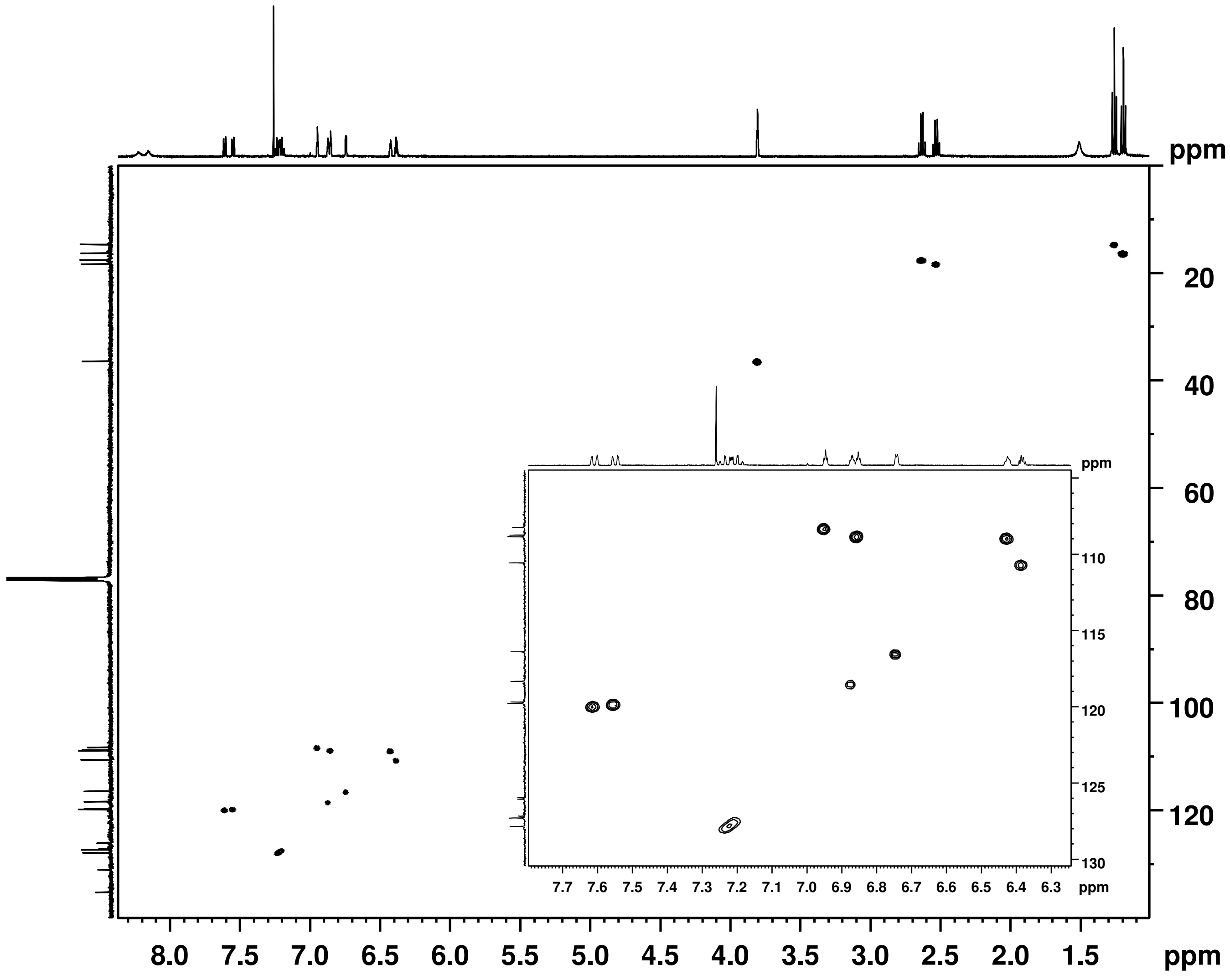

Figure S75. ^{1}H-^{1}H COSY (top) and HSQC (bottom) NMR spectra of carbatripyrrin **14d** in $CDCl_3$.

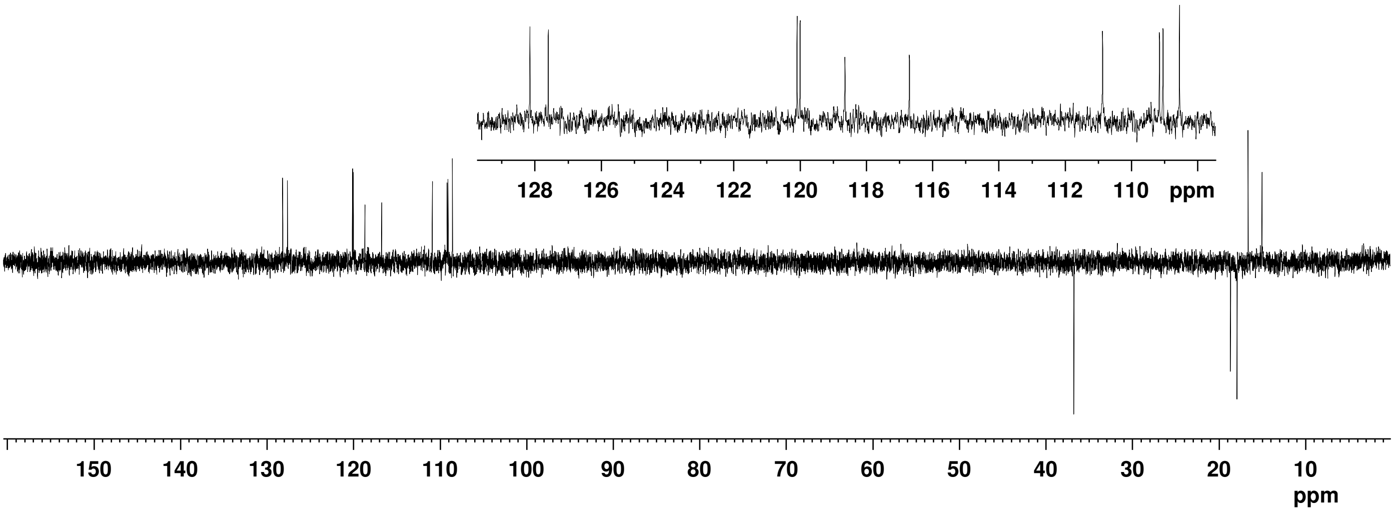

Figure S76. DEPT-135 NMR spectrum of carbatripyrrin **14d** in $CDCl_3$.

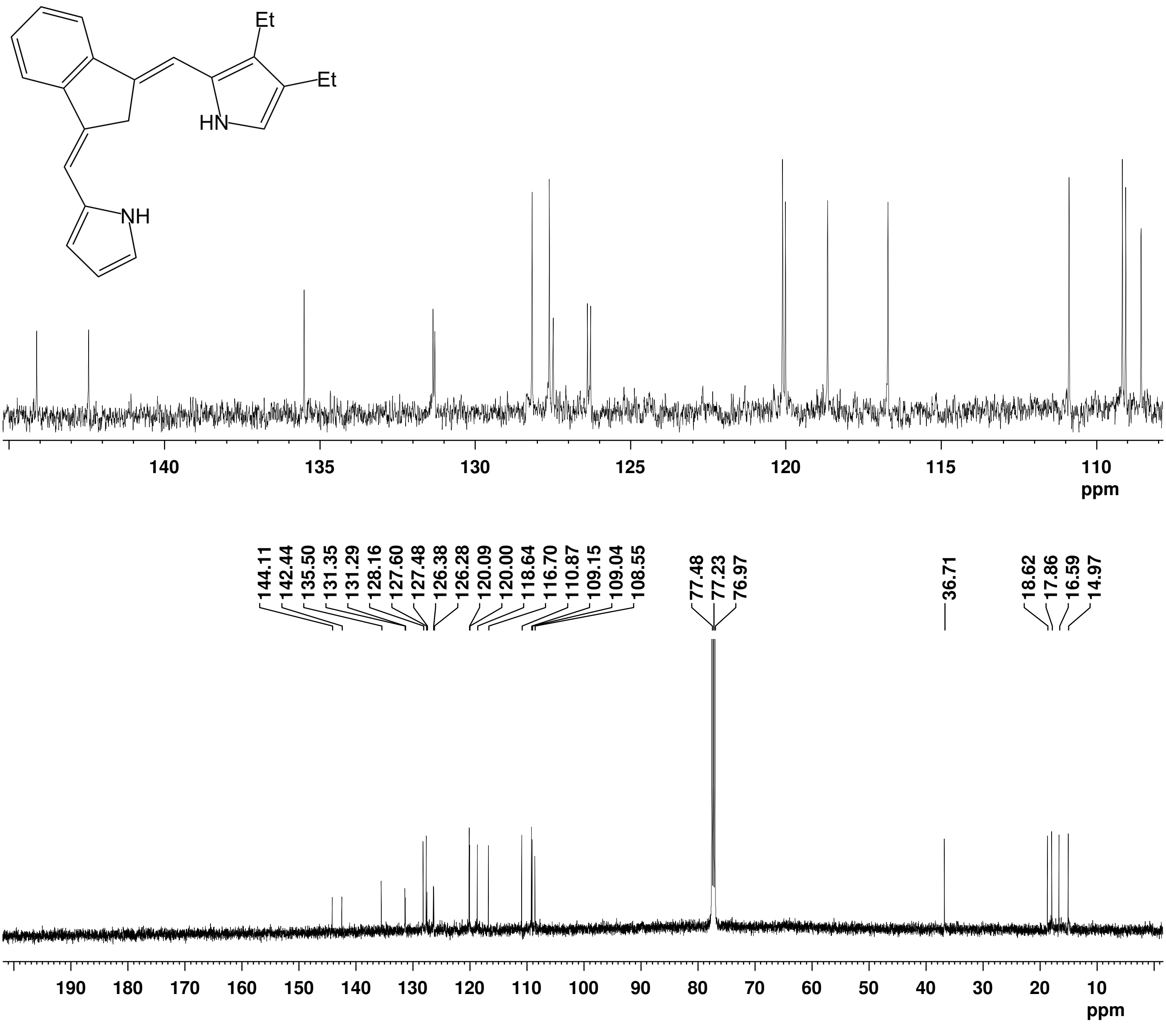

Figure S77. 125 MHz carbon-13 NMR spectrum of carbatripyrrin **14d** in $CDCl_3$.

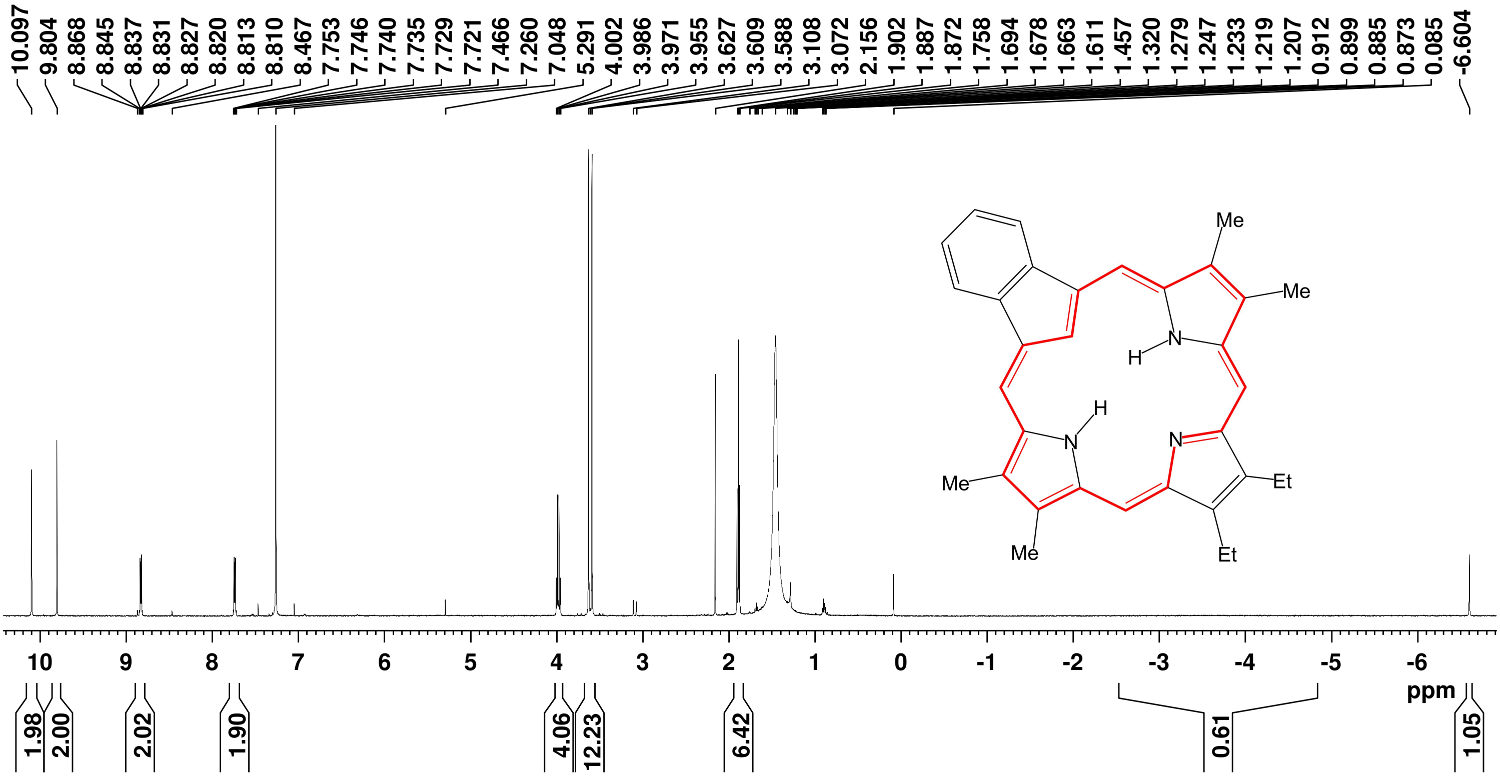

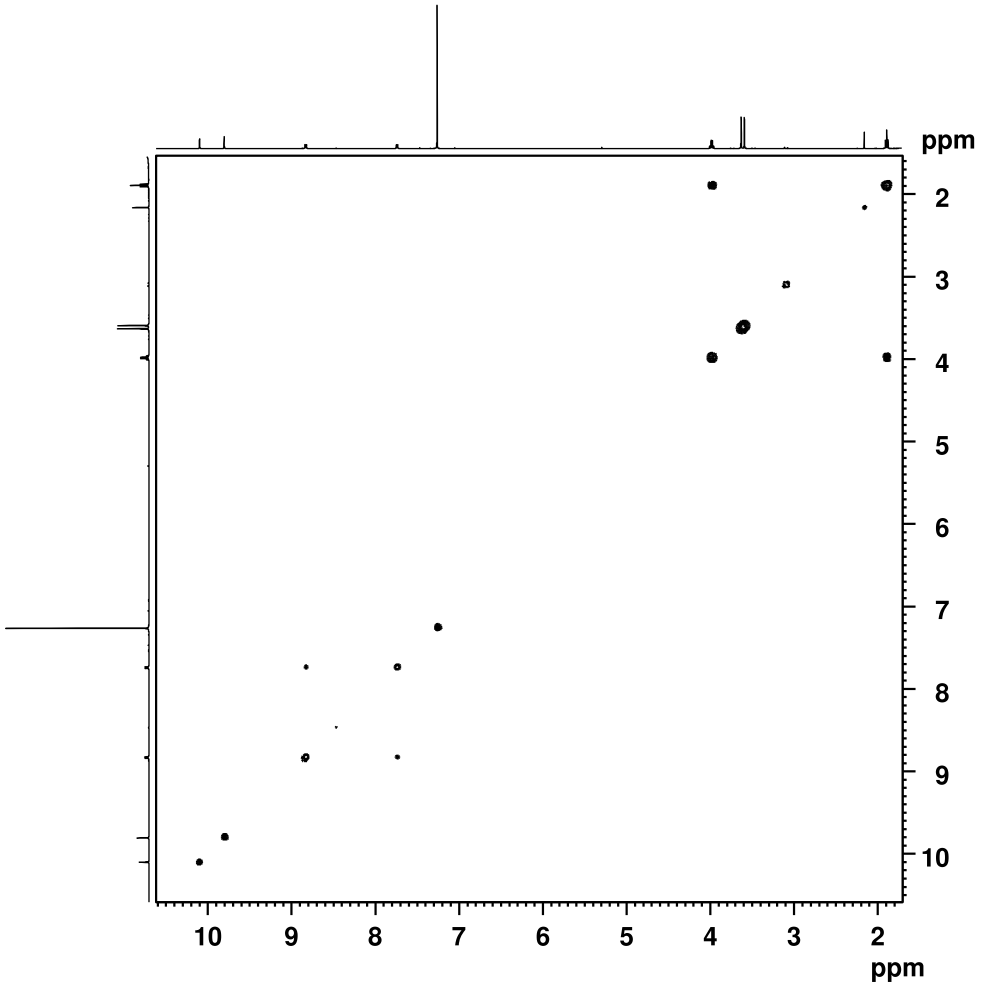

Figure S78. 500 MHz proton NMR (above) and 1H-1H COSY NMR spectra of carbaporphyrin **18a** in $CDCl_3$.

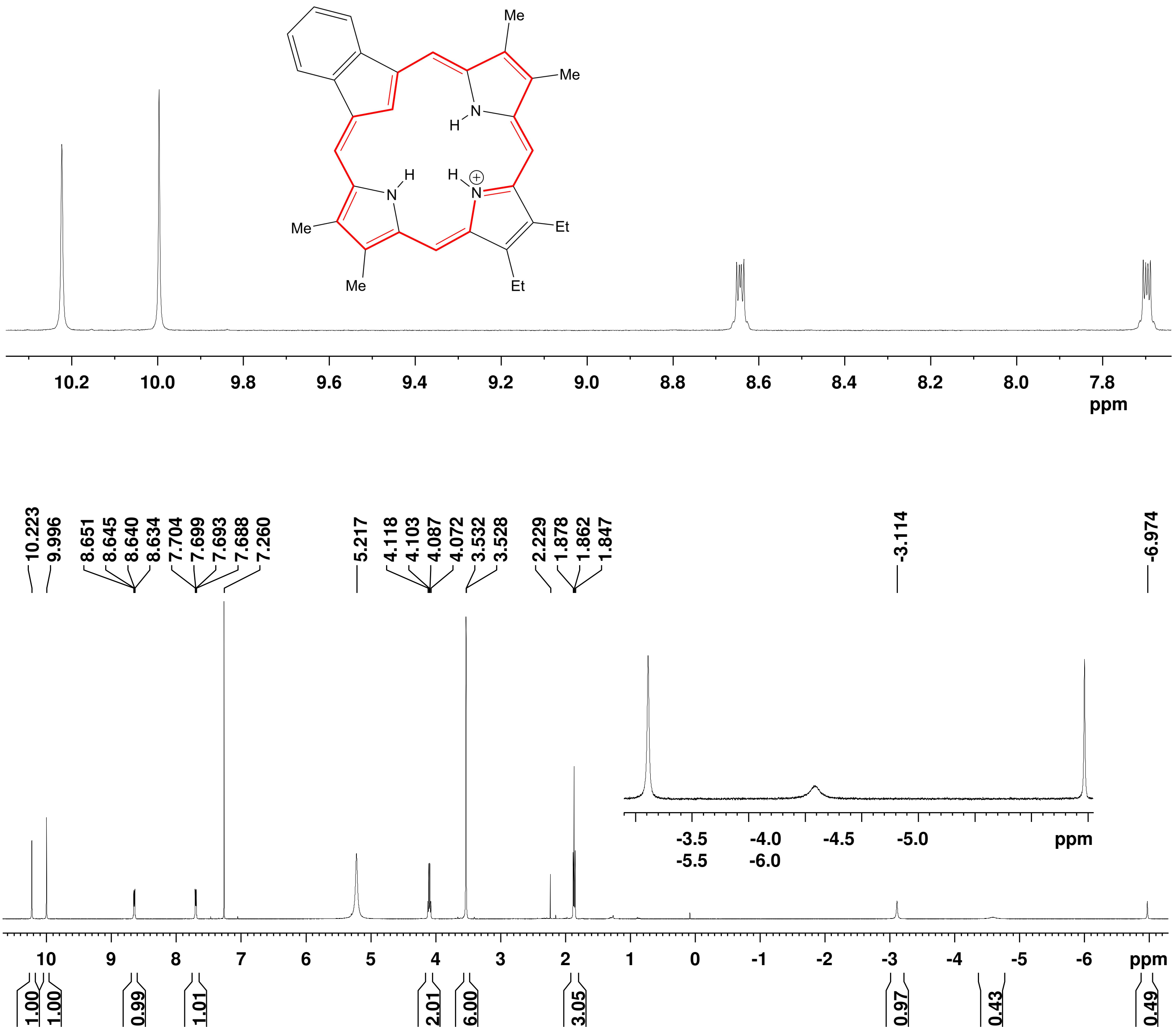

Figure S79. 500 MHz proton NMR spectrum of protonated carbaporphyrin **18a** in $CDCl_3$ containing 2 µL TFA.

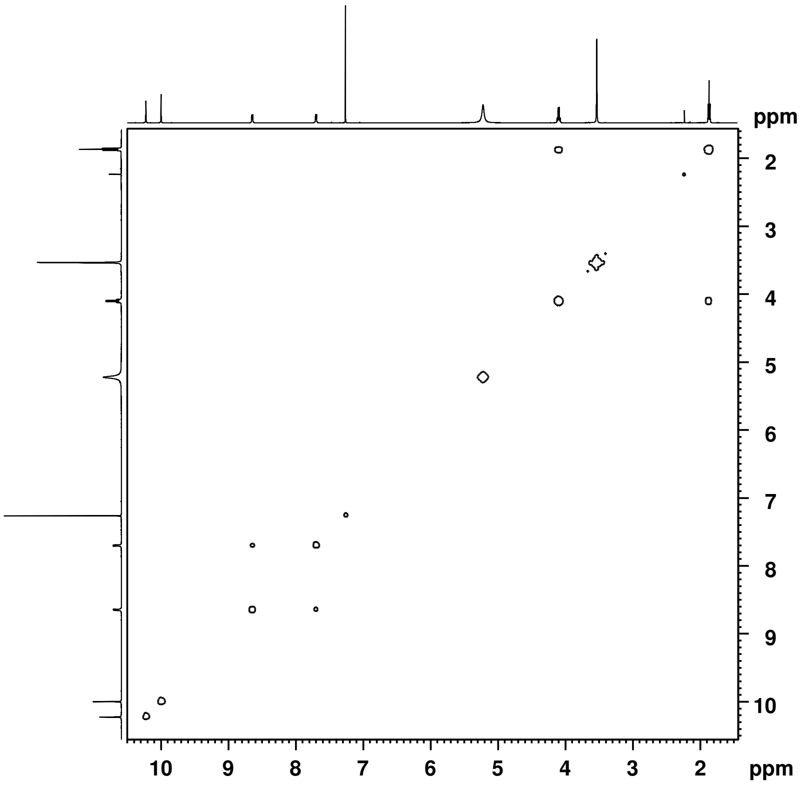

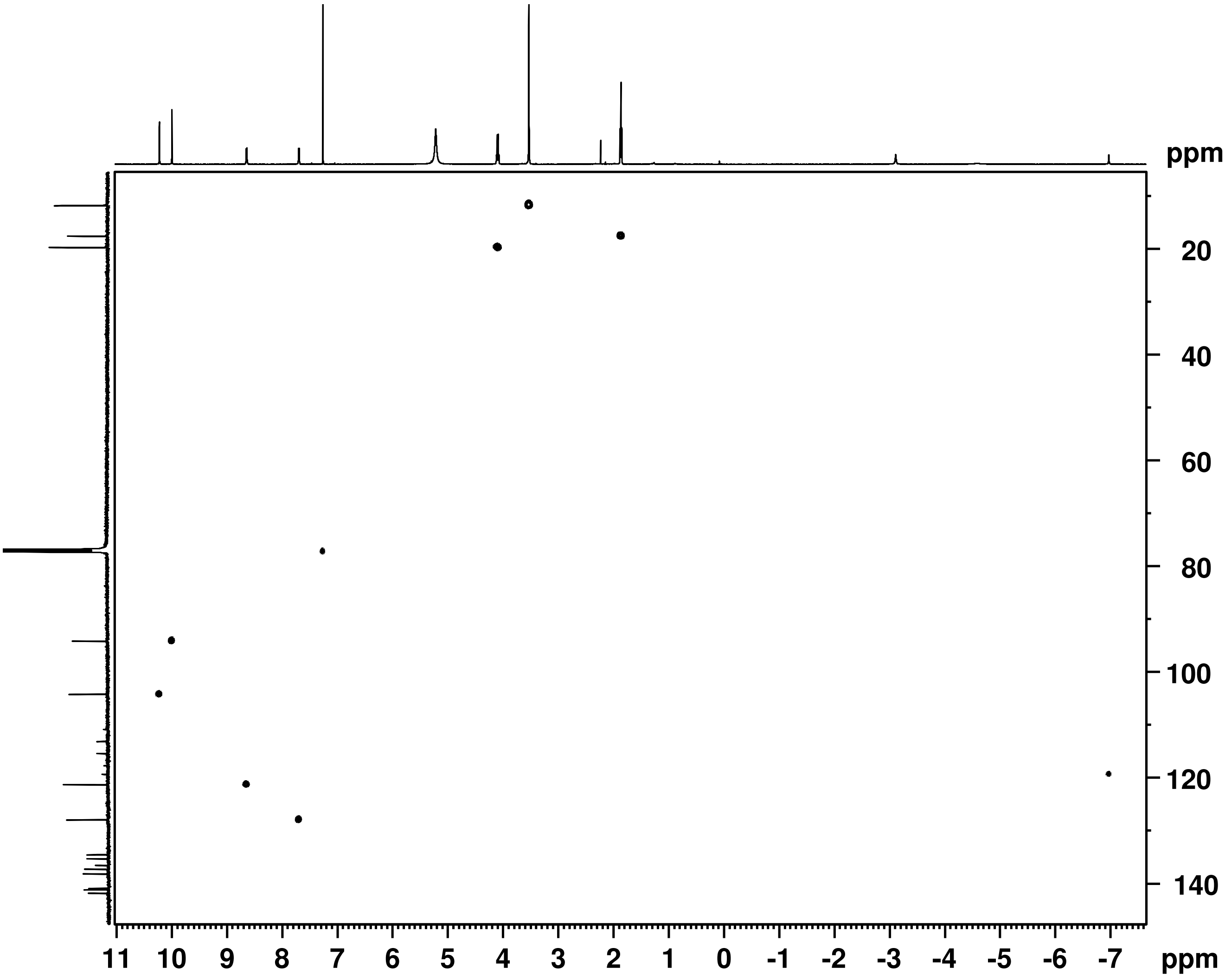

Figure S80. ^{1}H-^{1}H COSY (top) and HSQC (bottom) NMR spectra of **18a** in $CDCl_3$ with 2 μL TFA.

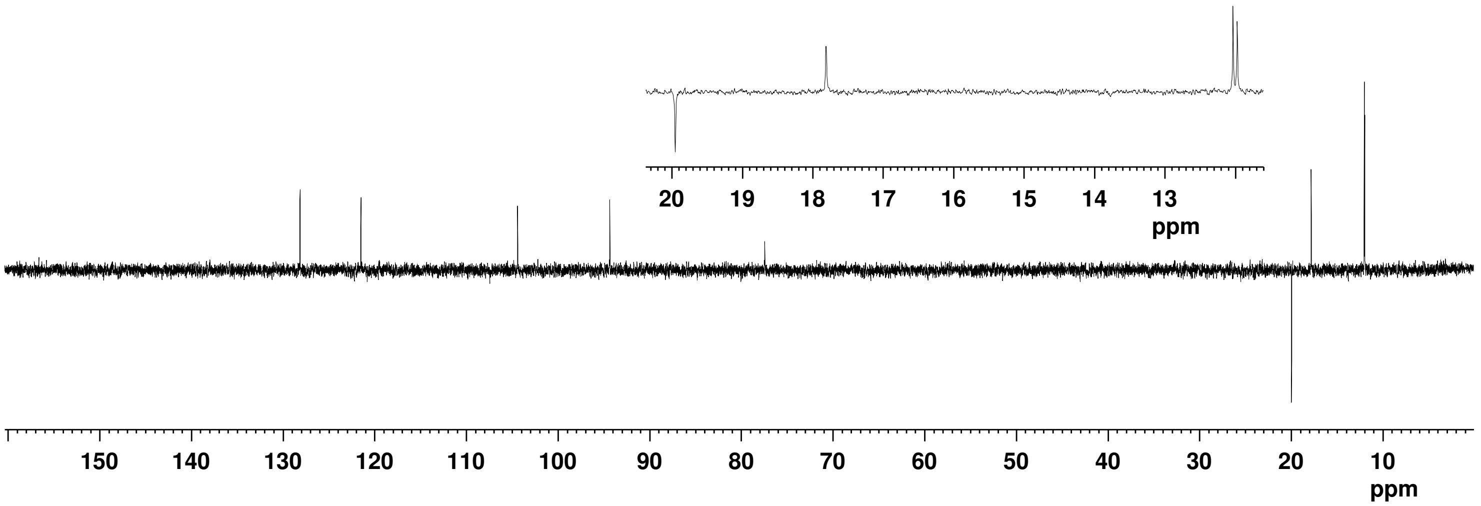

Figure S81. DEPT-135 NMR spectrum of **18a** in $CDCl_3$ with 2 µL TFA.

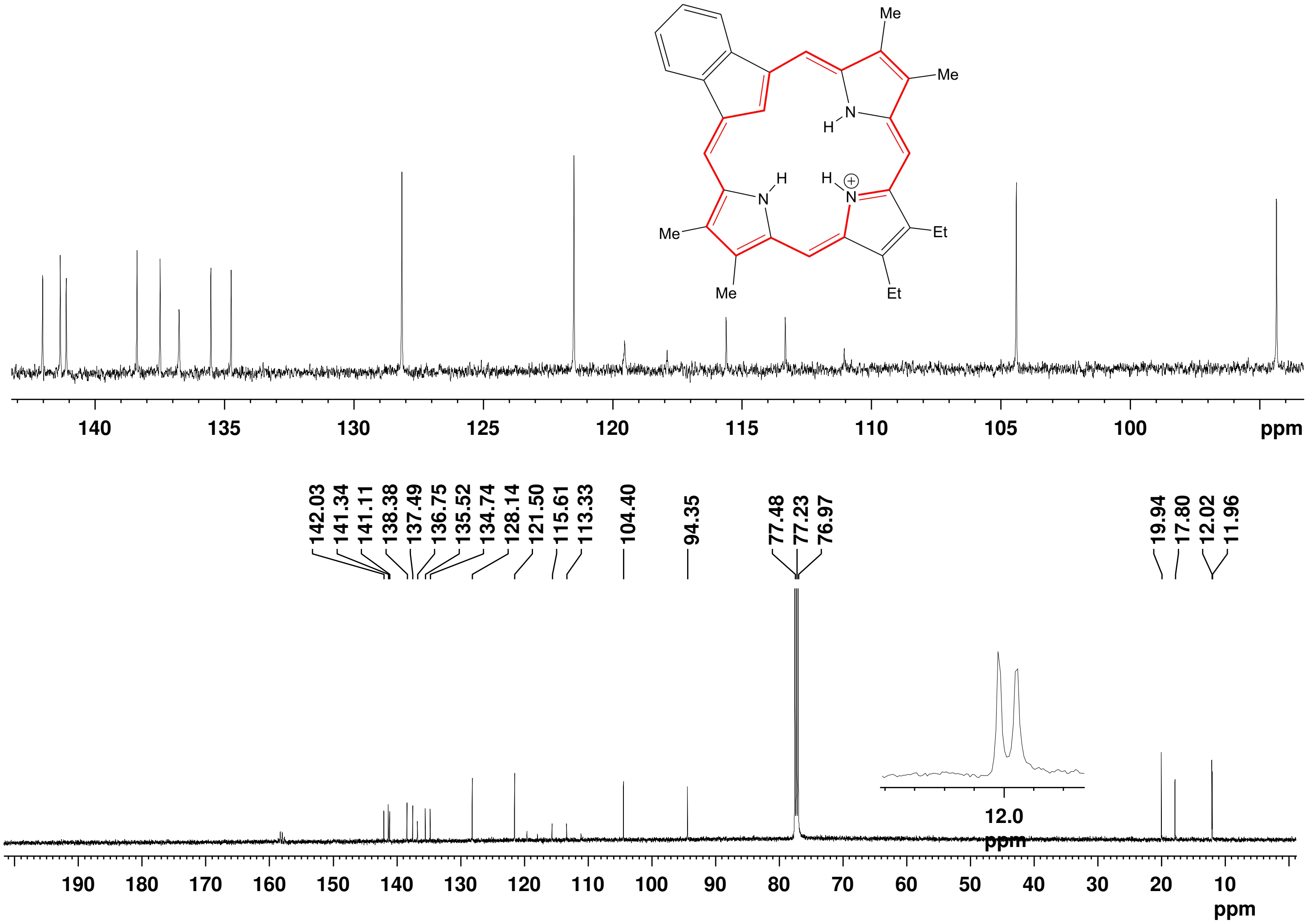

Figure S82. 125 MHz carbon-13 NMR spectrum of **18a** in $CDCl_3$ with 2 µL TFA.

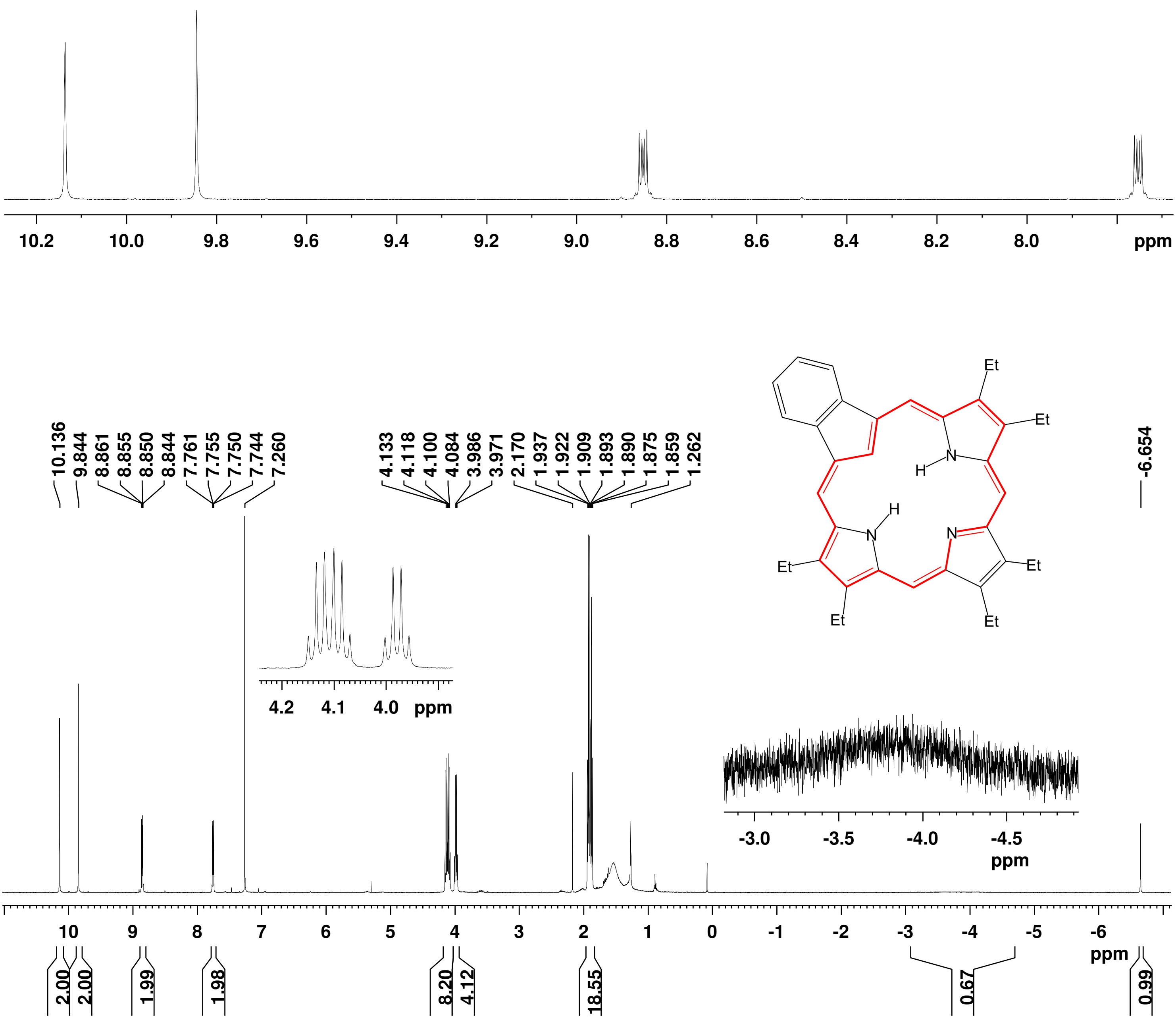

Figure S83. 500 MHz proton NMR spectrum of hexaethylcarbaporphyrin **18b** in $CDCl_3$.

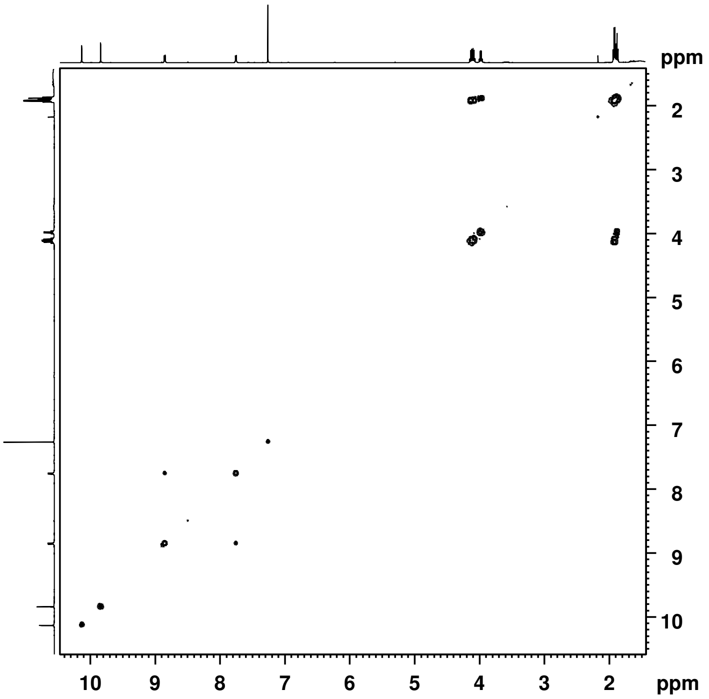

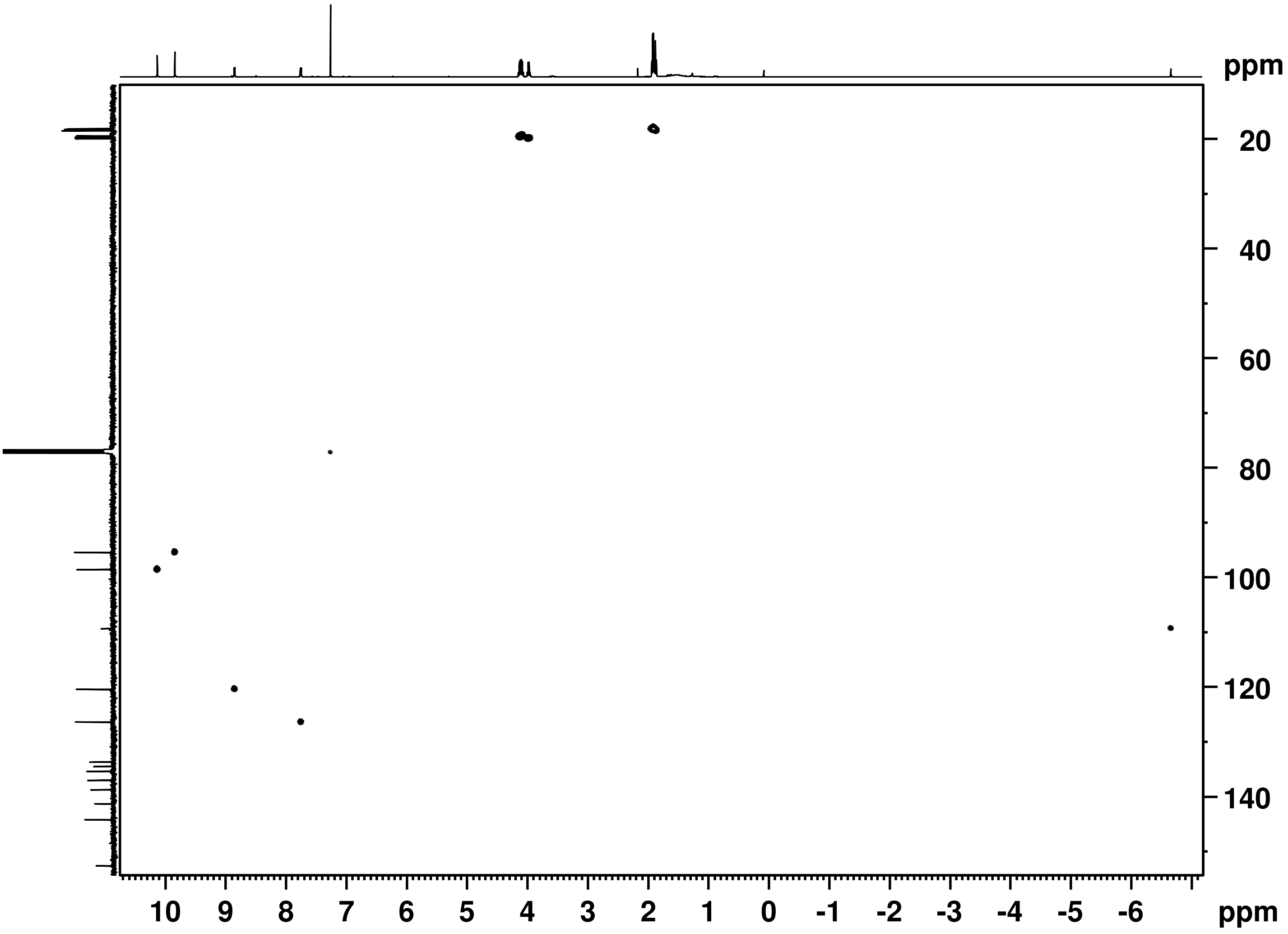

Figure S84. ^{1}H-^{1}H COSY (top) and HSQC (bottom) NMR spectra of **18b** in $CDCl_3$.

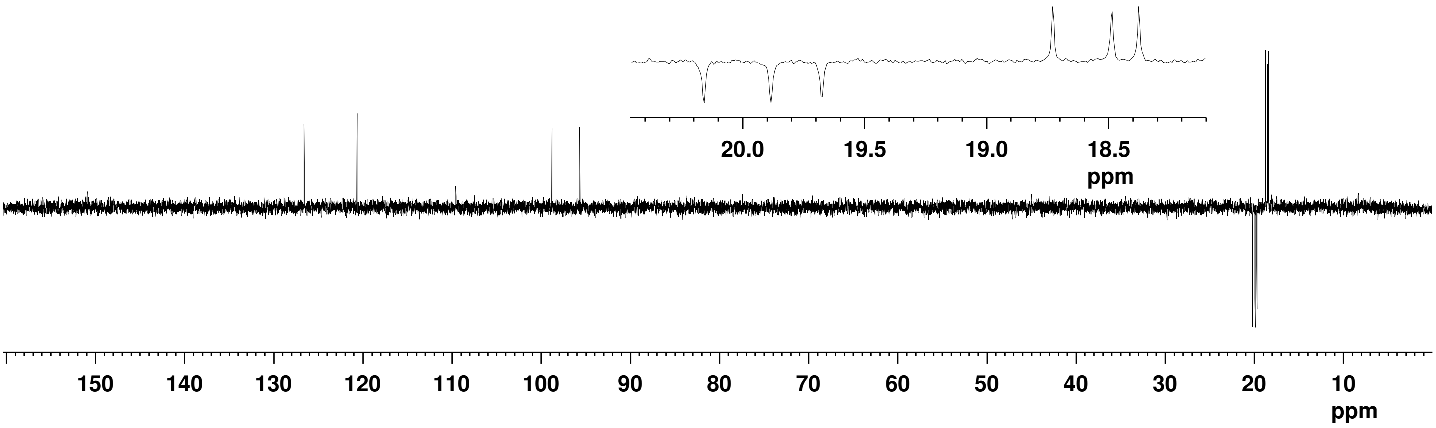

Figure S85. DEPT-135 NMR spectrum of hexaethylcarbaporphyrin **18b** in $CDCl_3$.

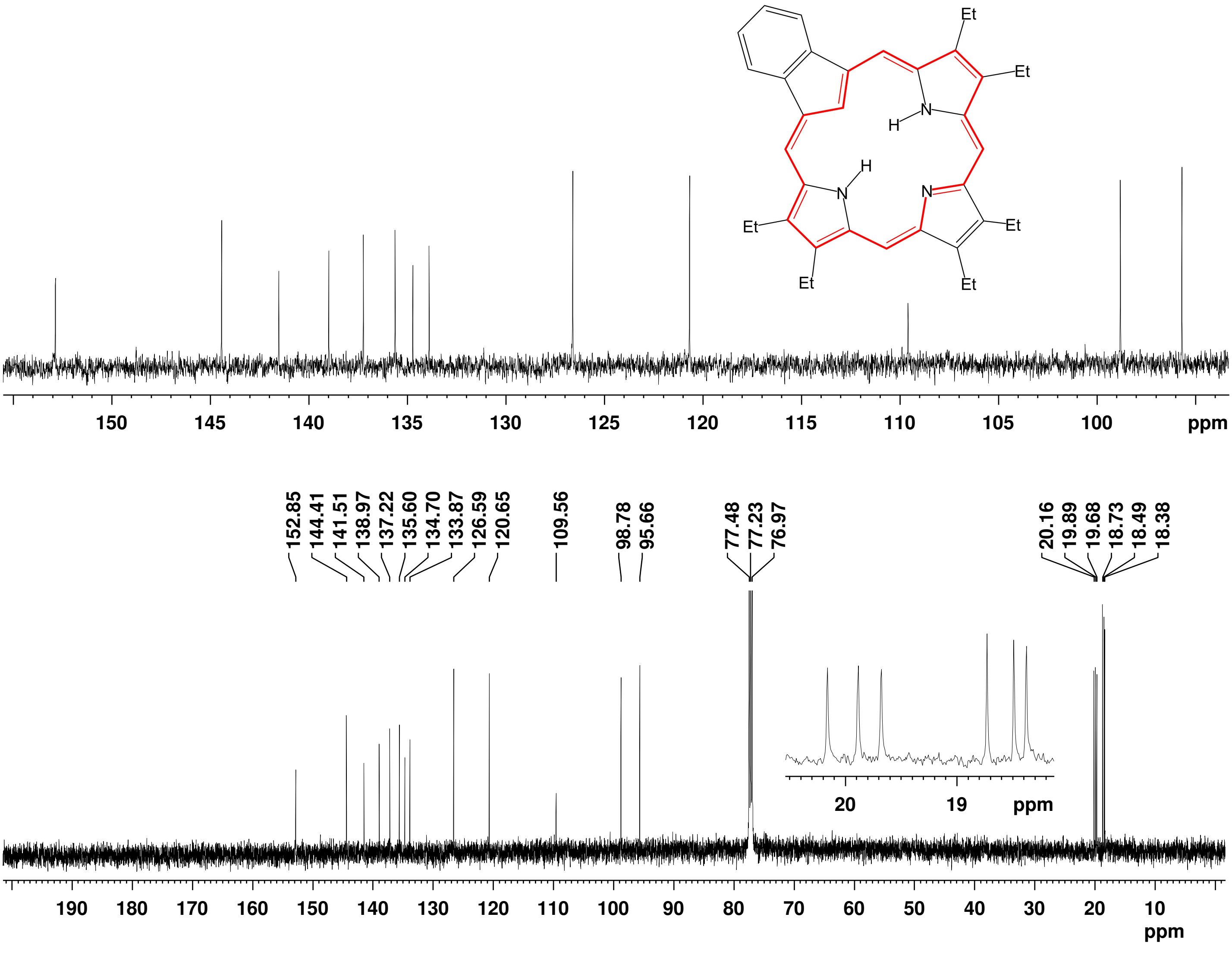

Figure S86. 125 MHz carbon-13 NMR spectrum of hexaethylcarbaporphyrin **18b** in $CDCl_3$.

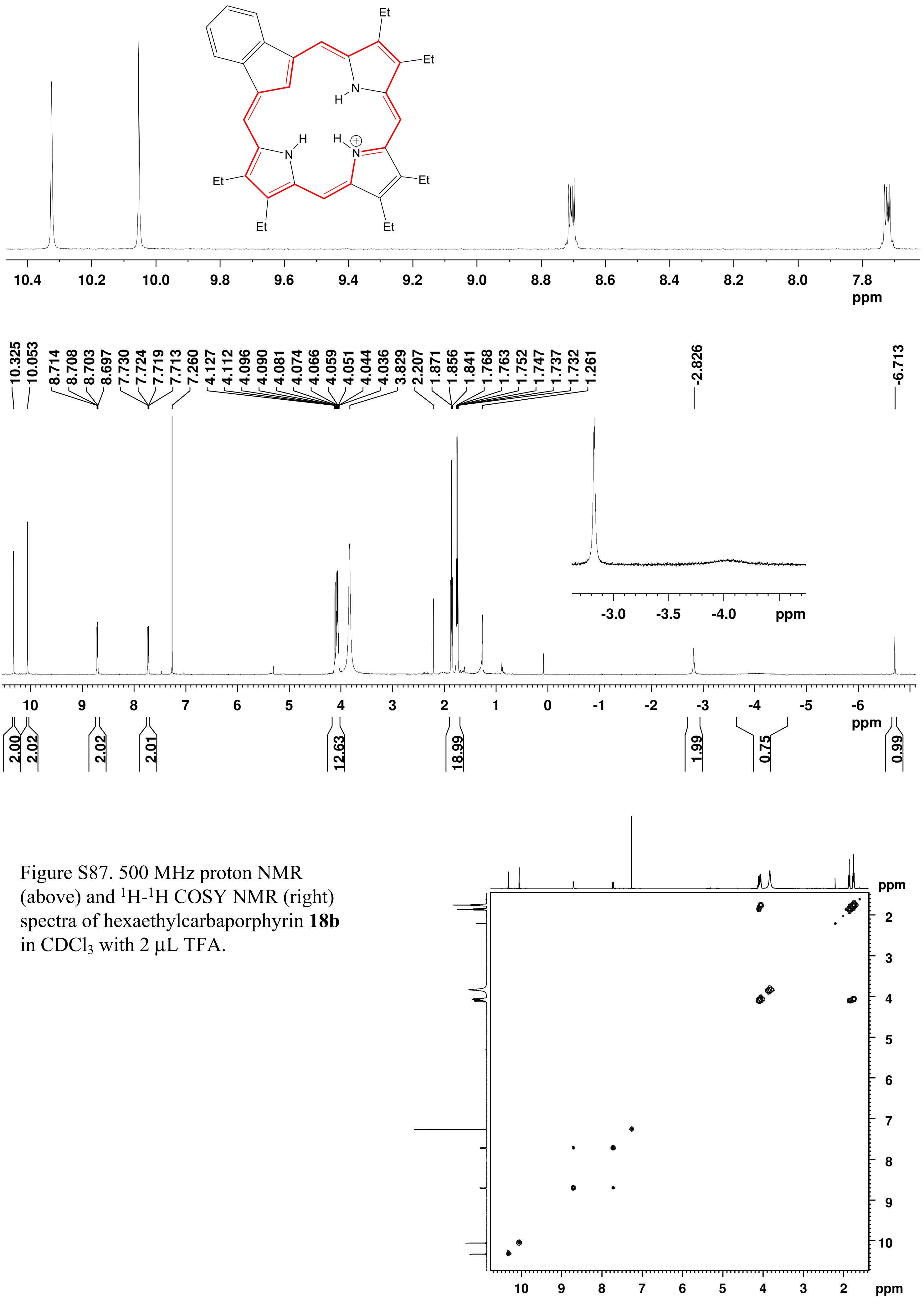

Figure S87. 500 MHz proton NMR (above) and ^{1}H-^{1}H COSY NMR (right) spectra of hexaethylcarbaporphyrin **18b** in $CDCl_3$ with 2 μL TFA.

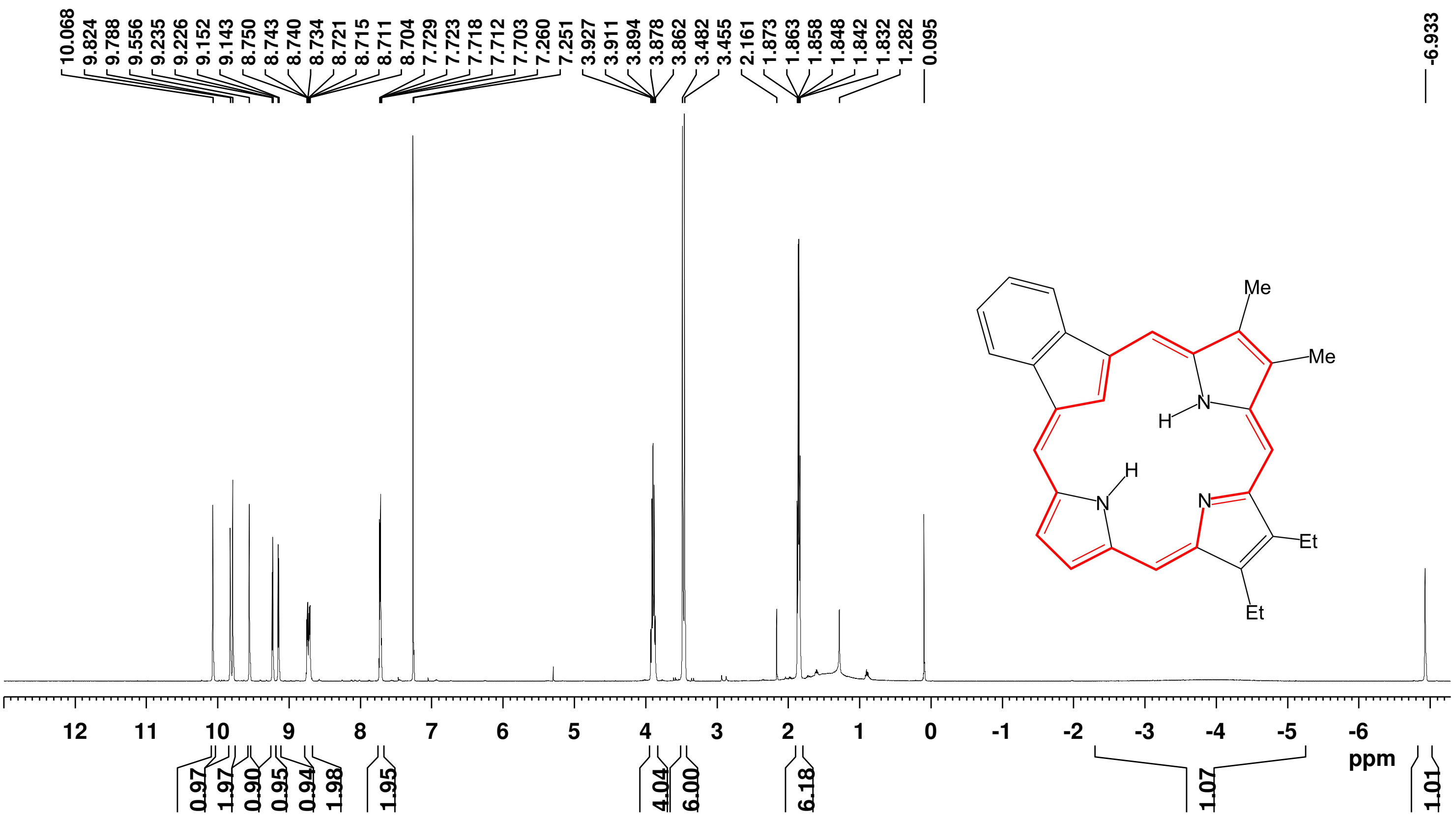

Figure S88. 500 MHz proton NMR spectrum of carbaporphyrin **18c** in $CDCl_3$.

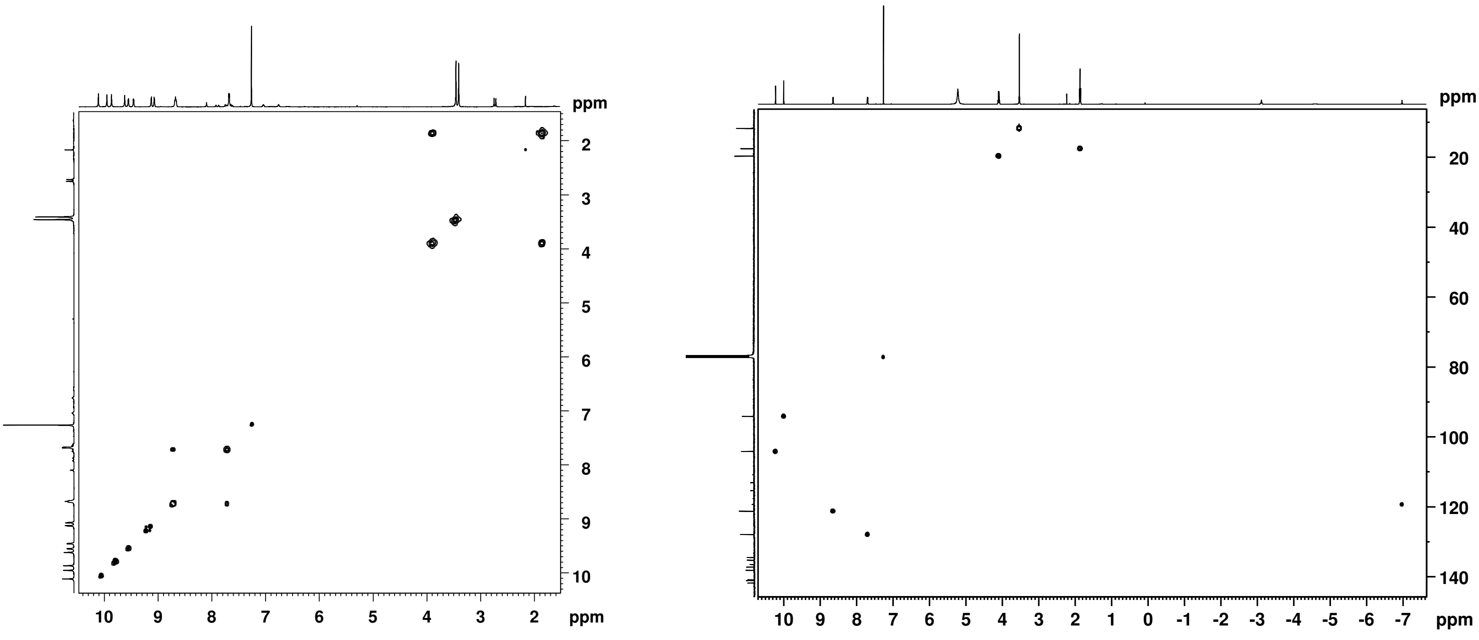

Figure S89. ^1H-^1H COSY (left) and HSQC NMR (right) spectra of **18c** in $CDCl_3$.

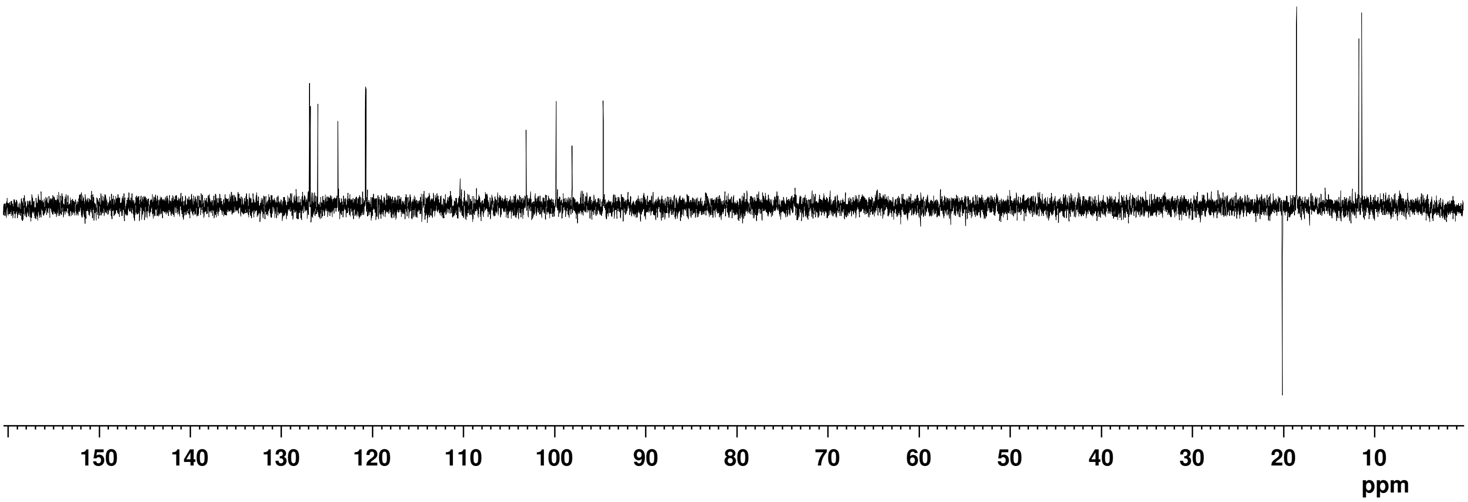

Figure S90. DEPT-135 NMR spectrum of **18c** in $CDCl_3$.

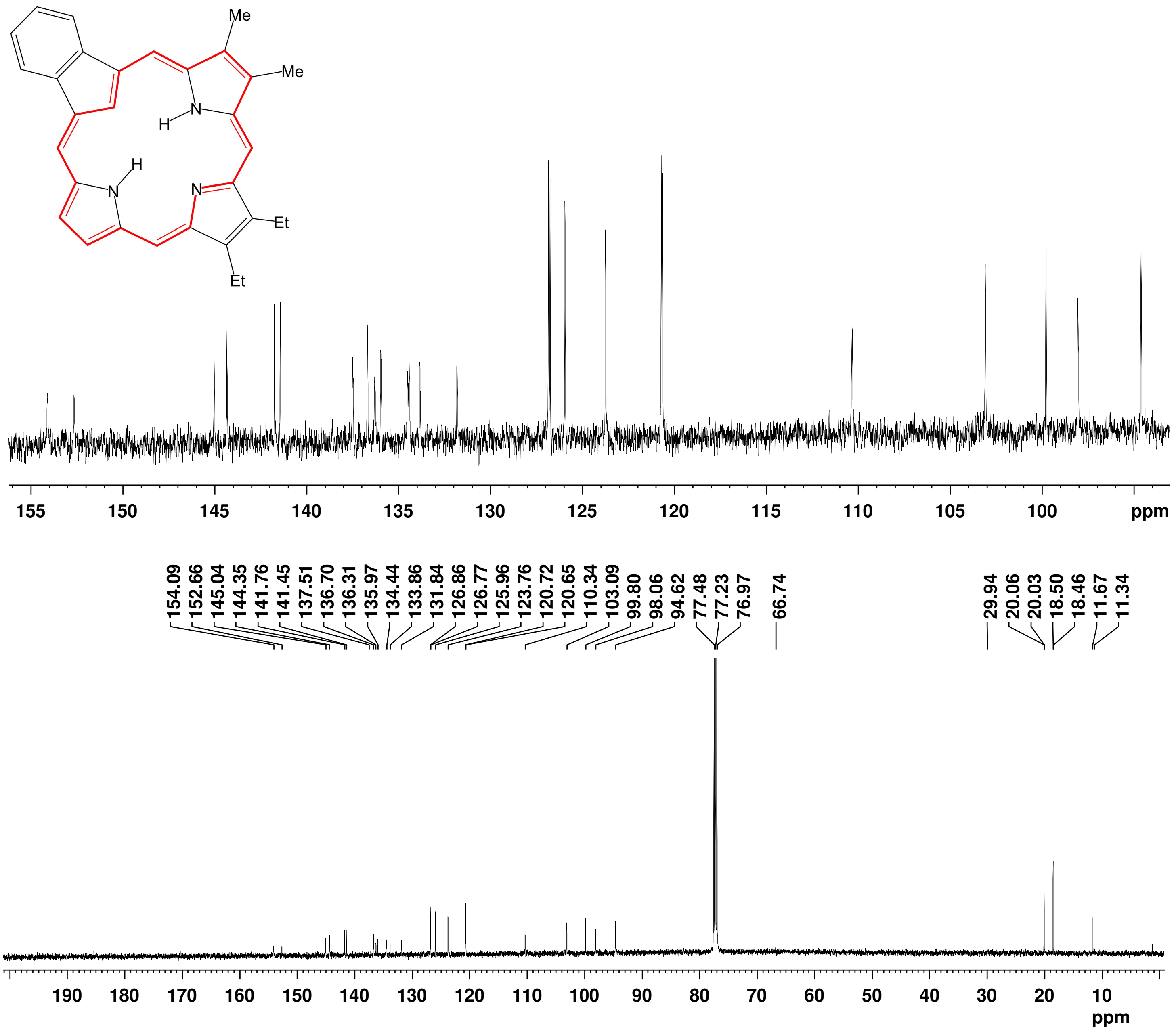

Figure S91. 125 MHz carbon-13 NMR spectrum of carbaporphyrin **18c** in $CDCl_3$.

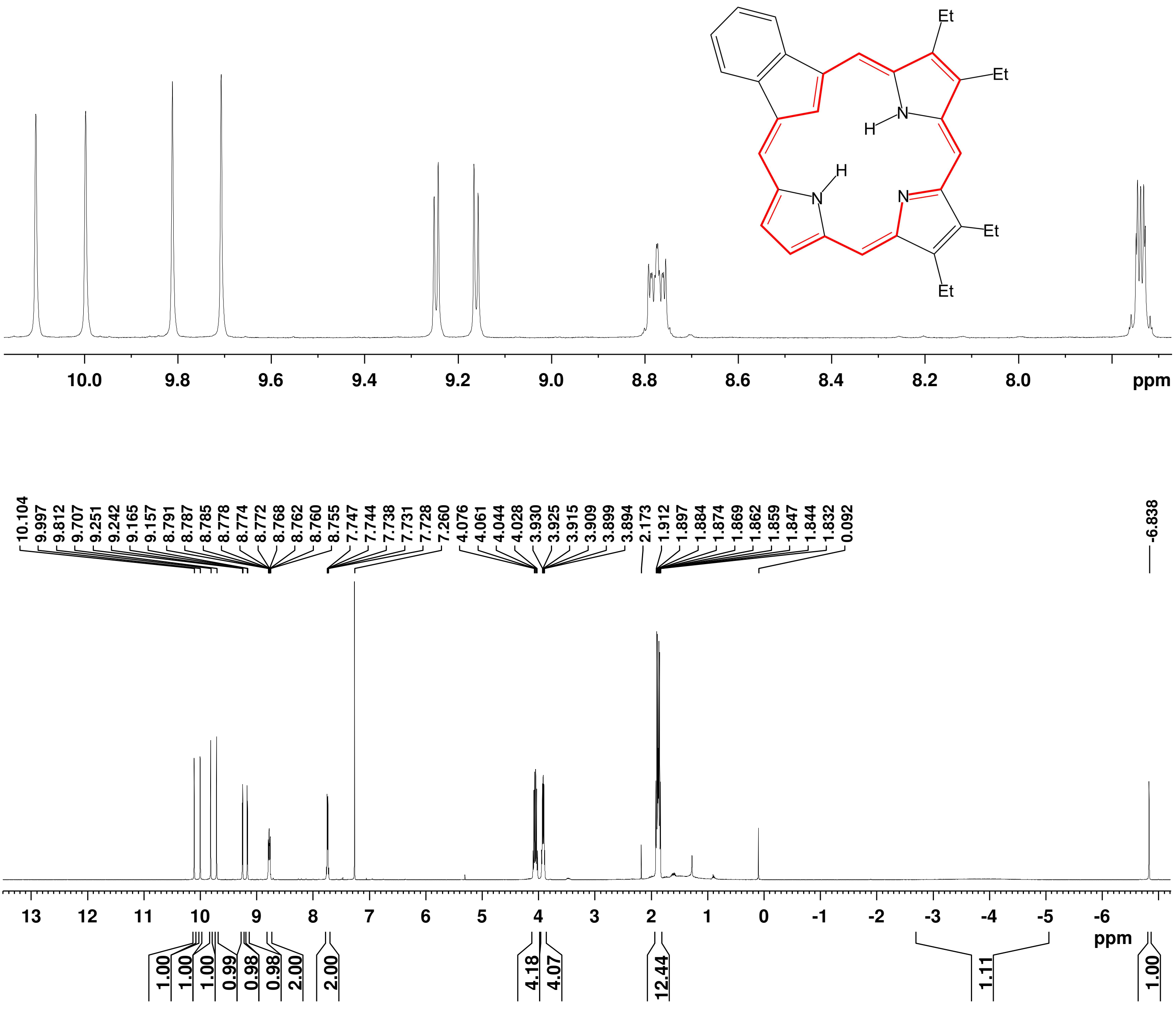

Figure S92. 500 MHz proton NMR spectrum of carbaporphyrin **18d** in $CDCl_3$.

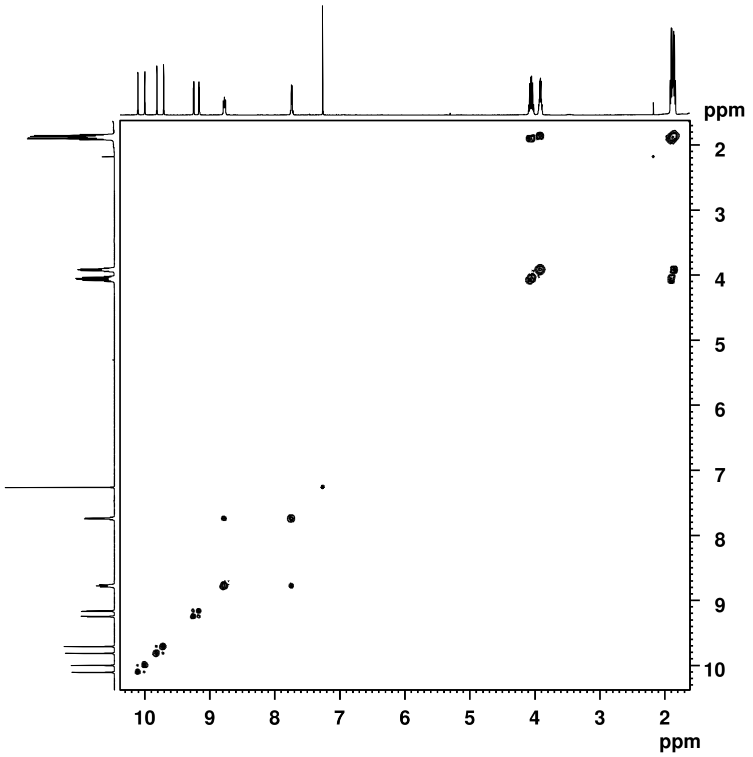

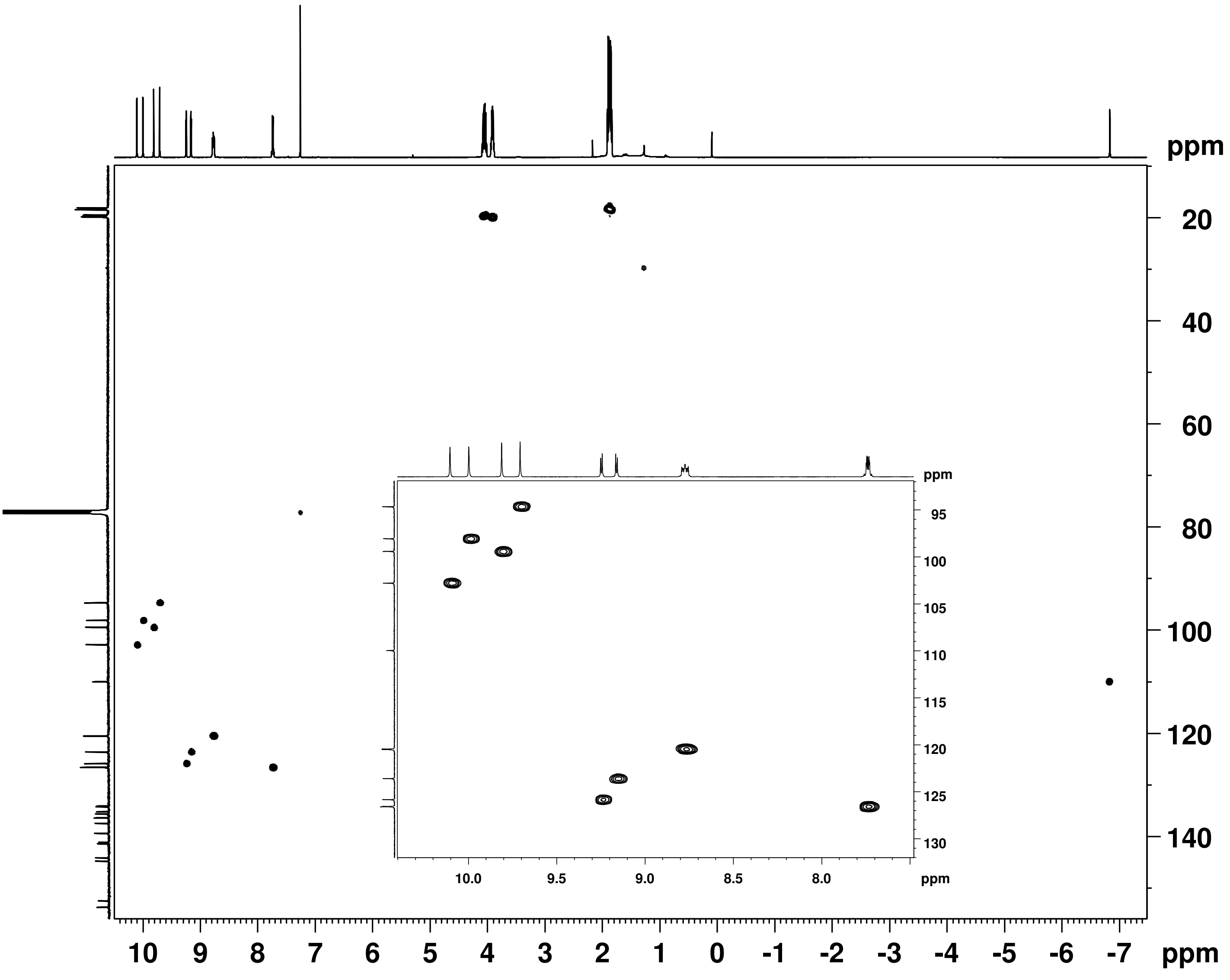

Figure S93. ^{1}H-^{1}H COSY (above) and HSQC (below) NMR spectra of carbaporphyrin **18d** in $CDCl_3$.

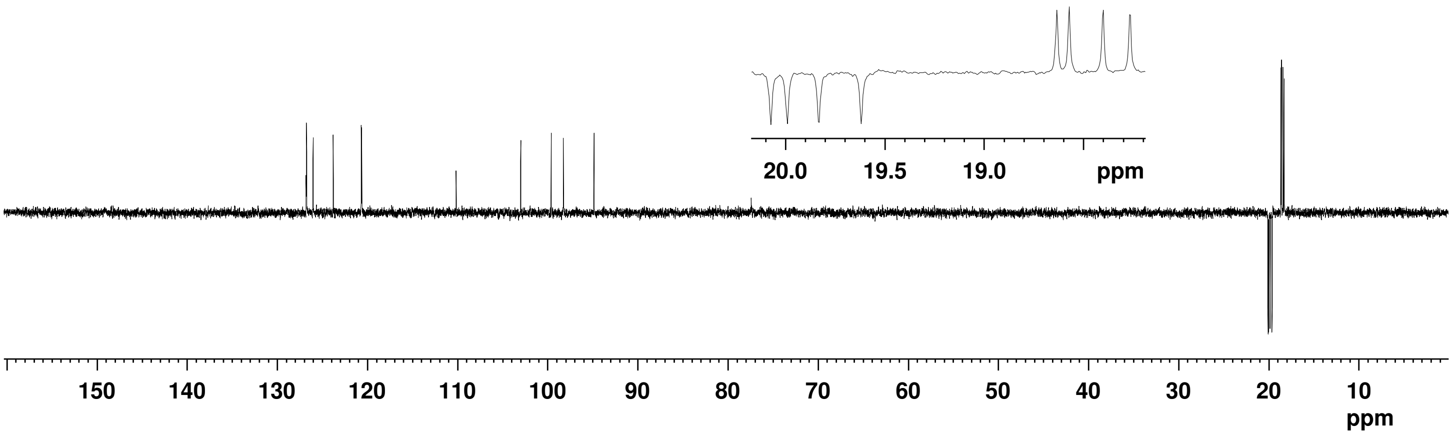

Figure S94. DEPT-135 NMR spectrum of carbaporphyrin **18d** in $CDCl_3$.

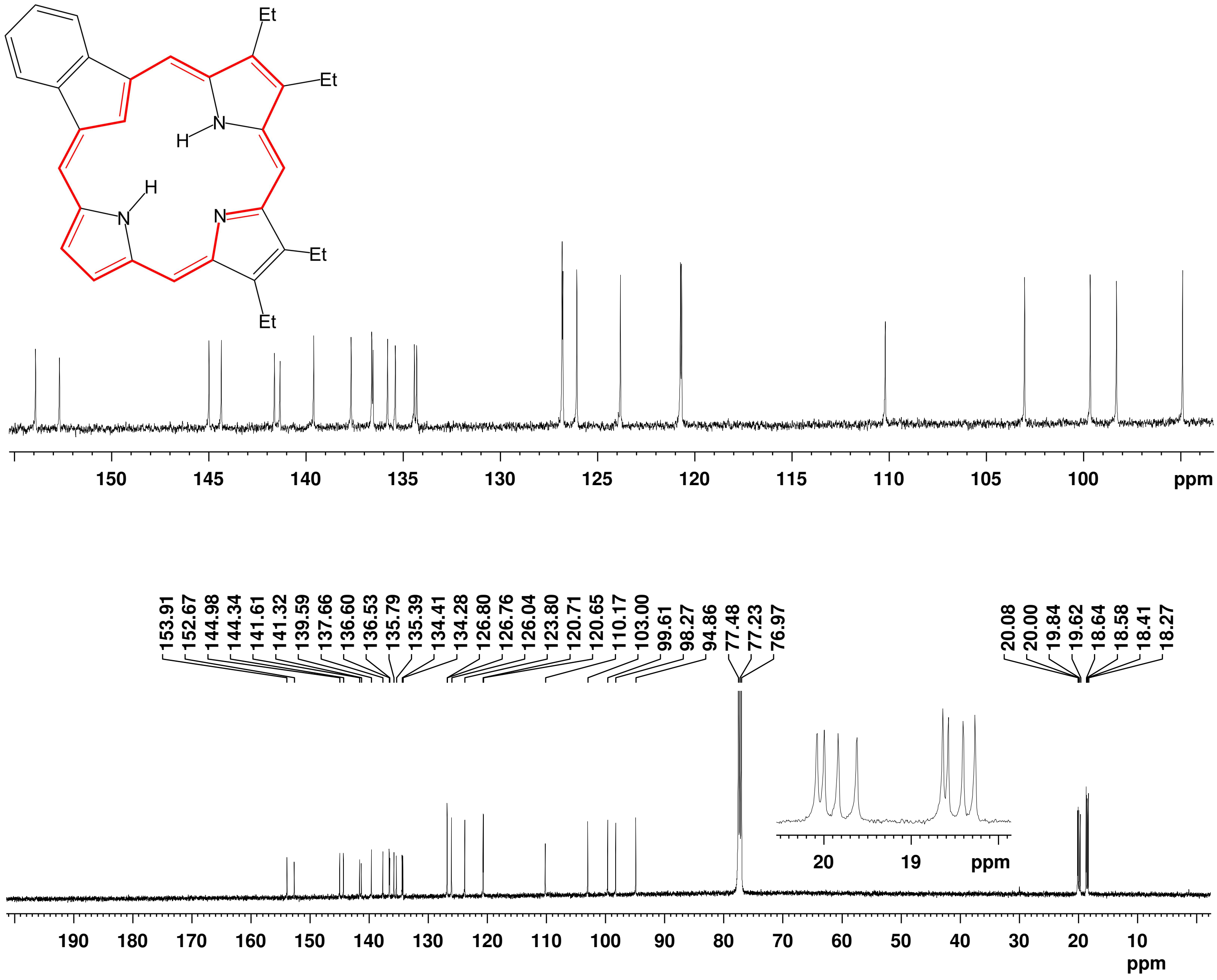

Figure S95. 125 MHz carbon-13 NMR spectrum of carbaporphyrin **18d** in $CDCl_3$.

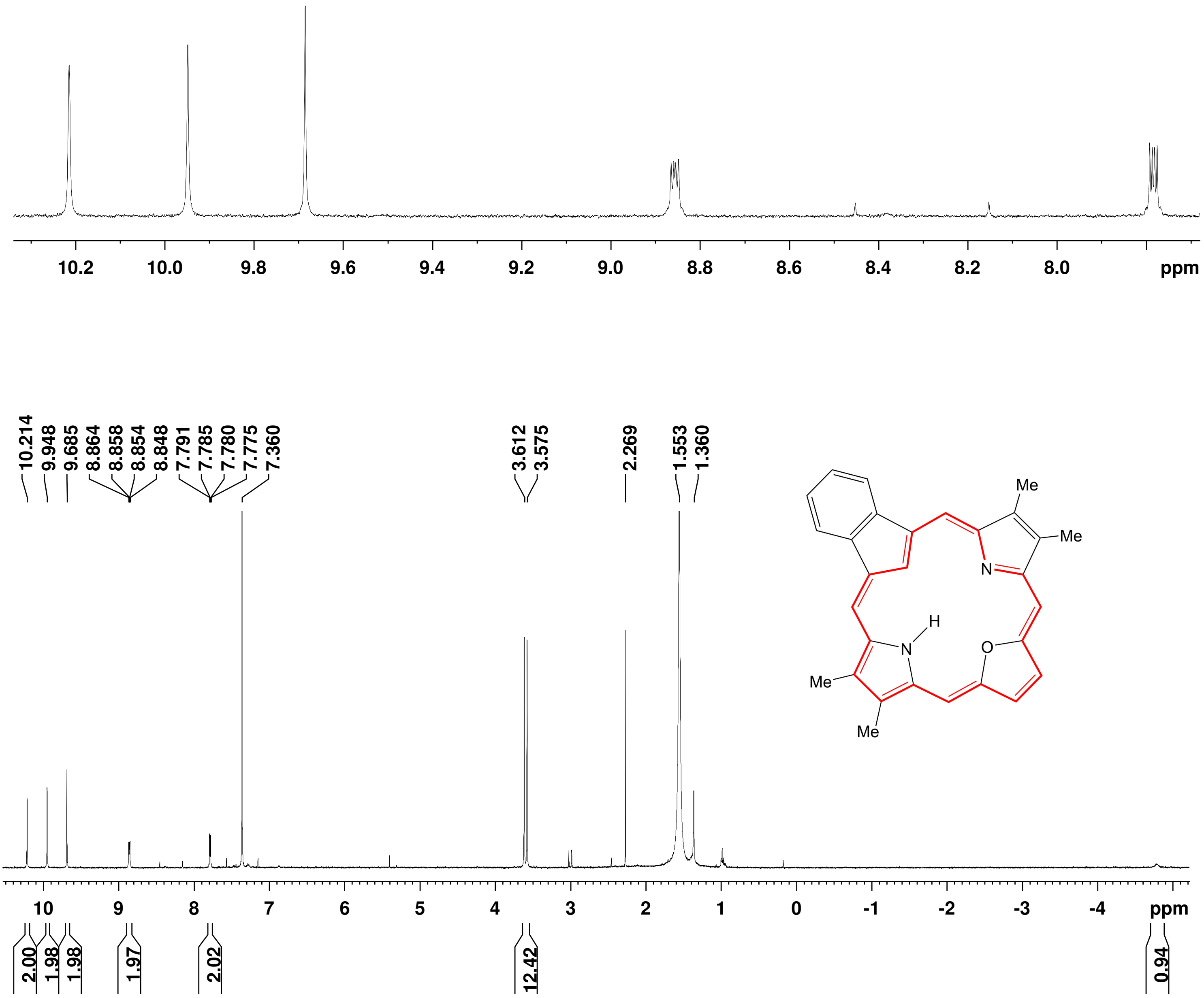

Figure S96. 500 MHz proton NMR spectrum of tetramethyloxaarbaporphyrin **19a** in $CDCl_3$.

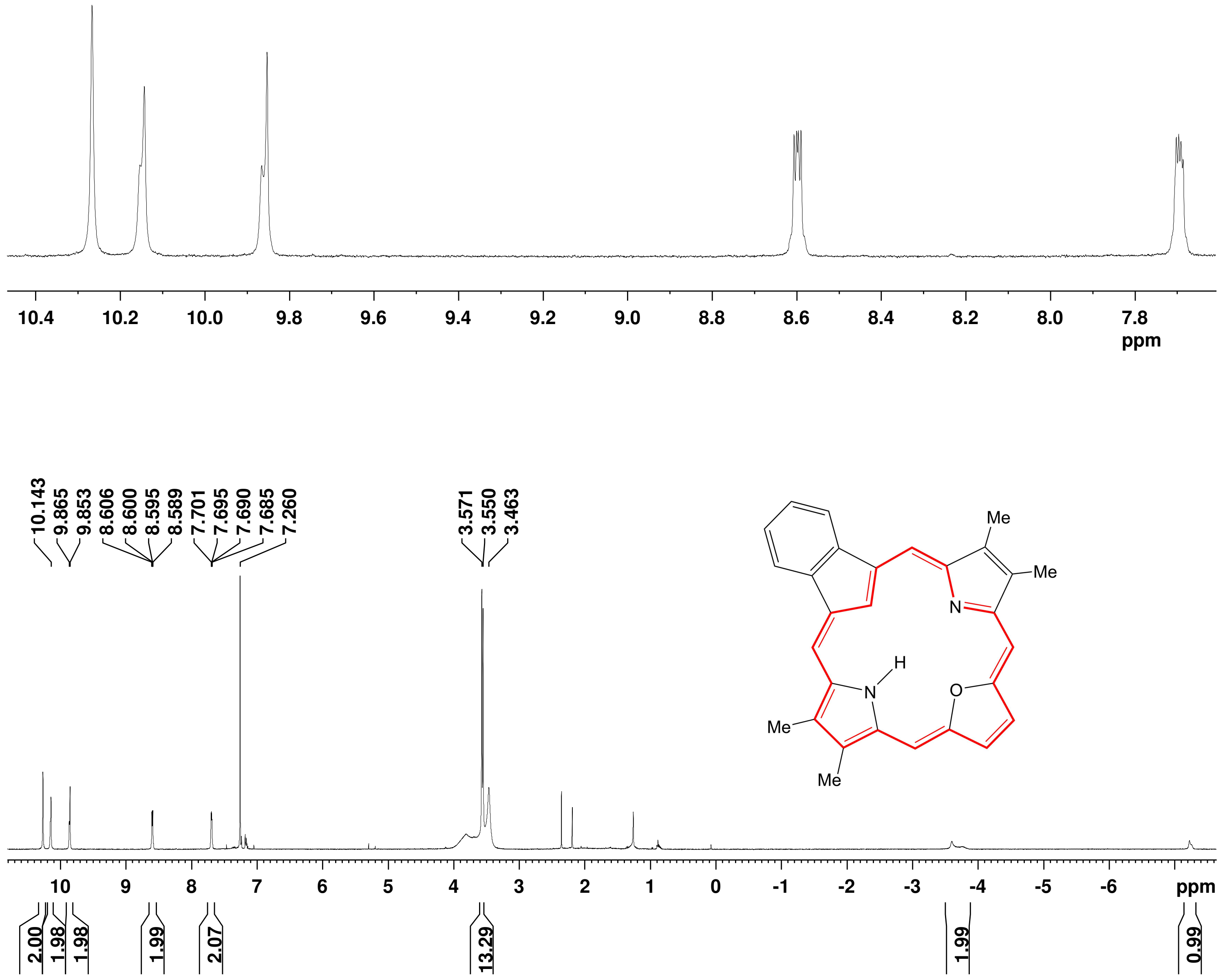

Figure S97. 500 MHz proton NMR spectrum of **19a** in $CDCl_3$ with 2 μL TFA.

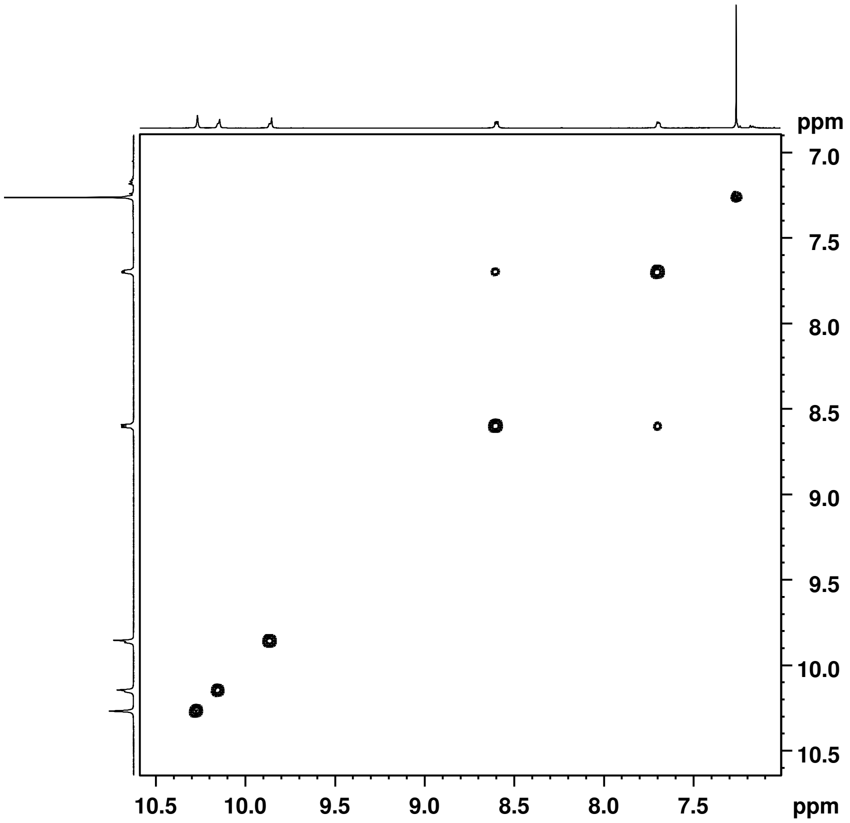

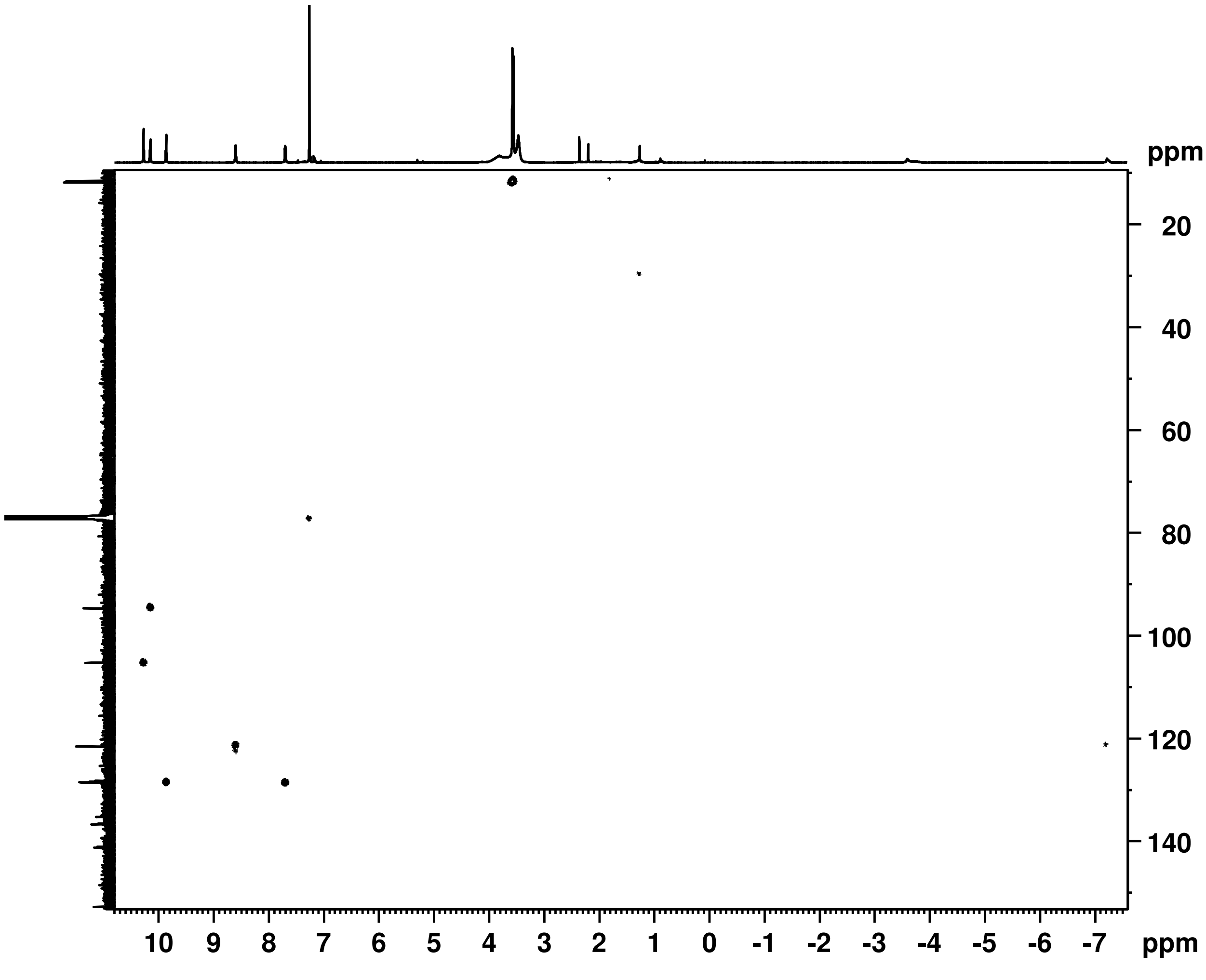

Figure S98. ^{1}H-^{1}H COSY (top) and HSQC (bottom) NMR spectra of **19a** in $CDCl_3$ with 2 μL TFA.

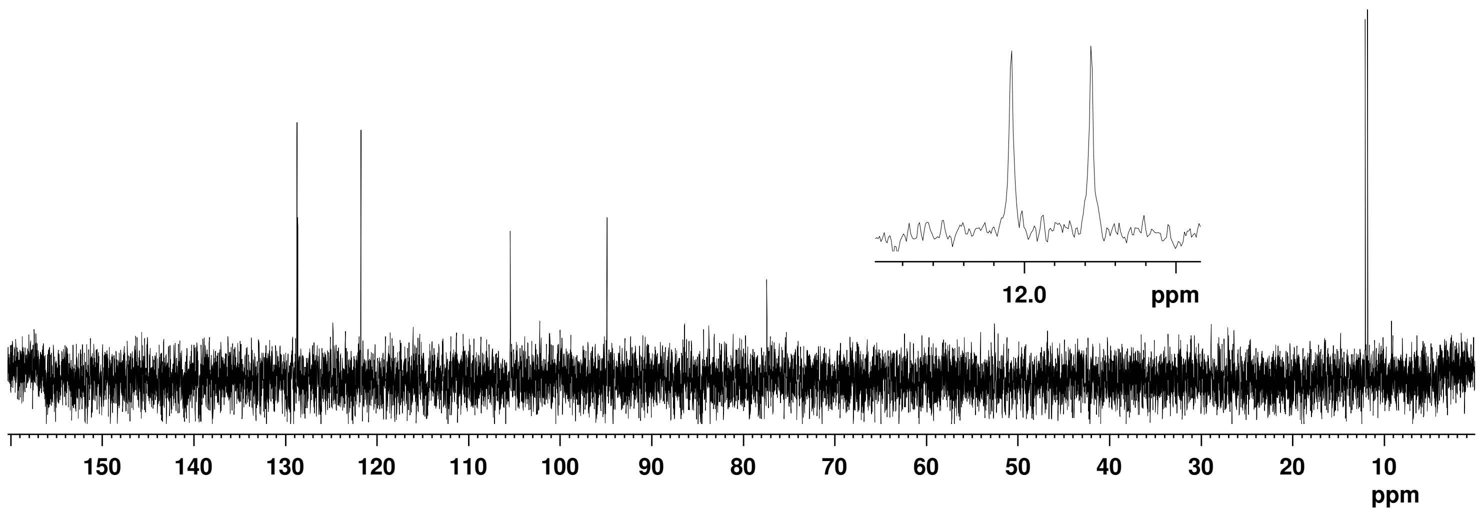

Figure S99. DEPT-135 NMR spectrum of **19a** in $CDCl_3$ with 2 μL TFA.

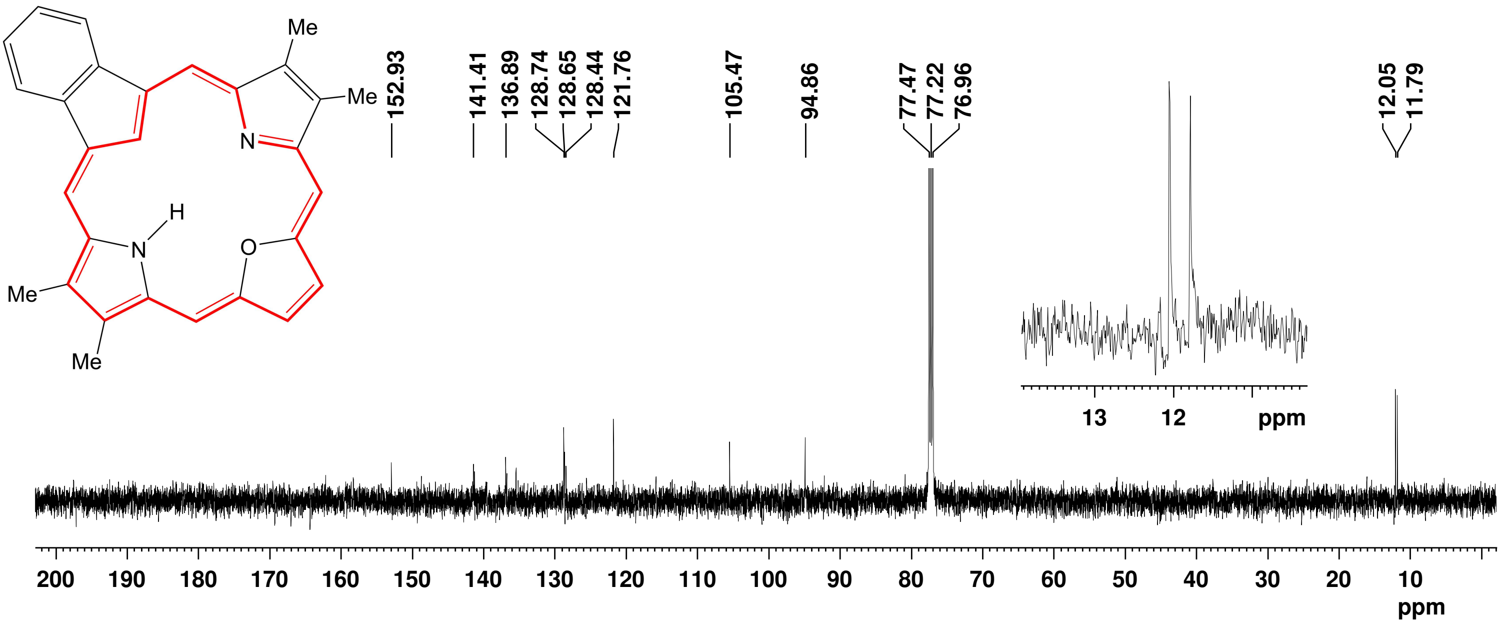

Figure S100. 125 MHz carbon-13 NMR spectrum of **19a** in $CDCl_3$ with 2 μL TFA.

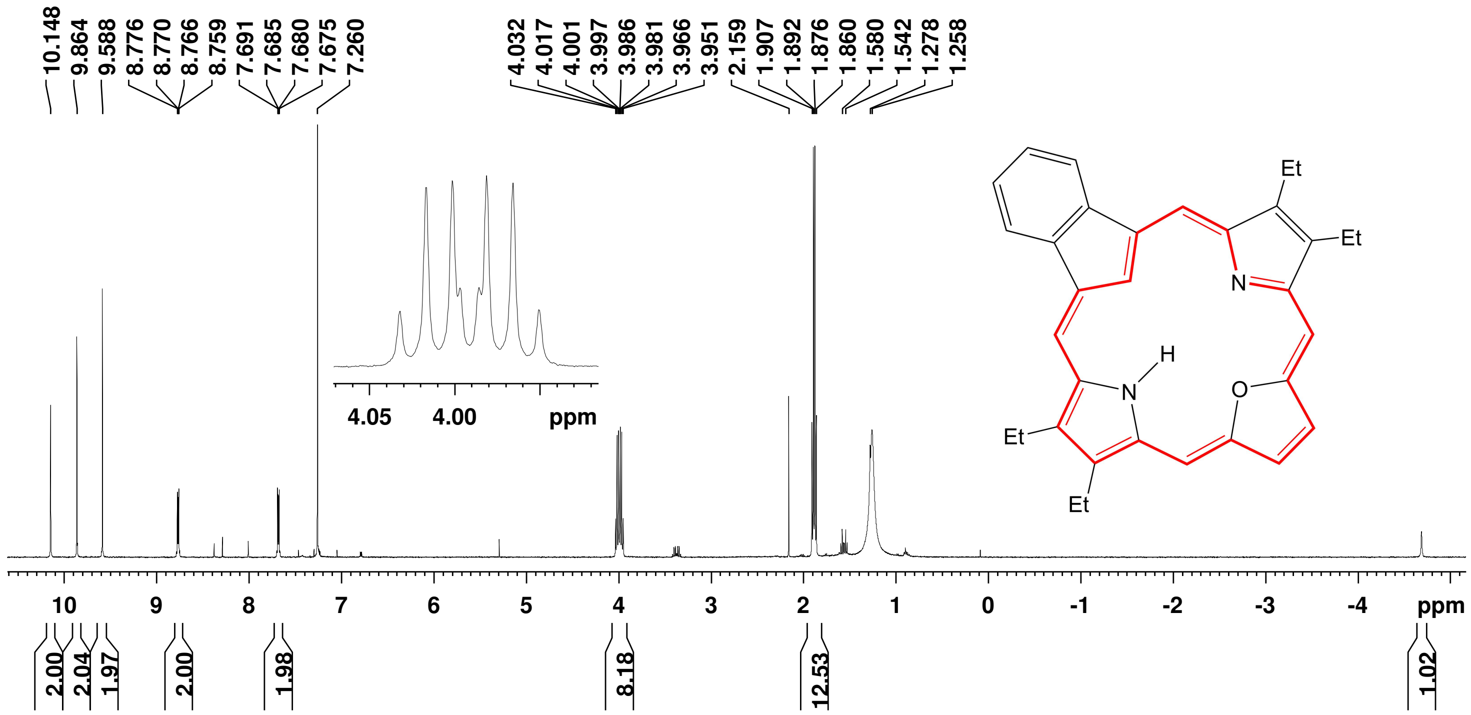

Figure S101. 500 MHz proton NMR spectrum of tetraethyloxacarbaporphyrin **19b** in $CDCl_3$.

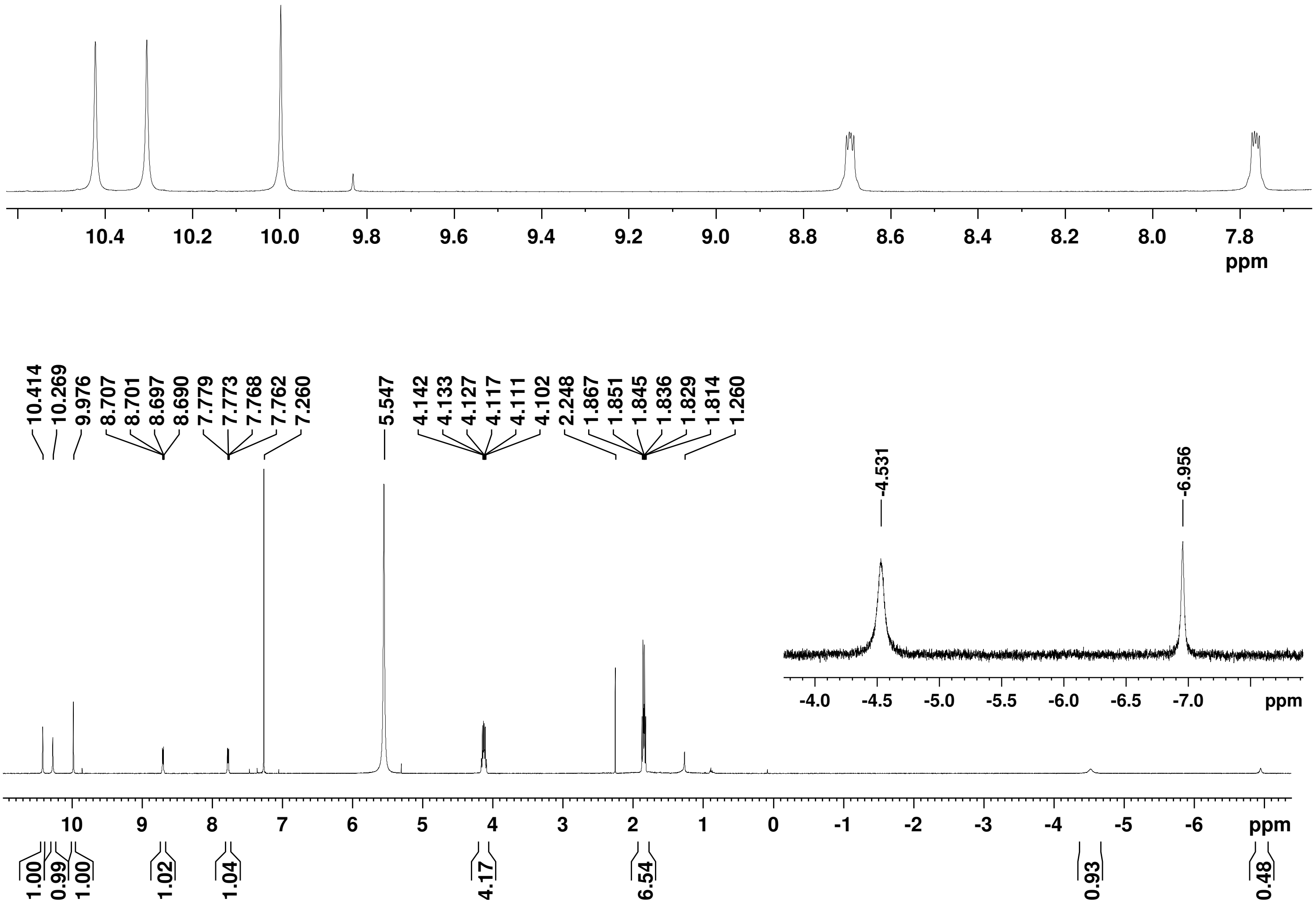

Figure S102. 500 MHz proton NMR spectrum of **19b** in $CDCl_3$ with 2 μL TFA.

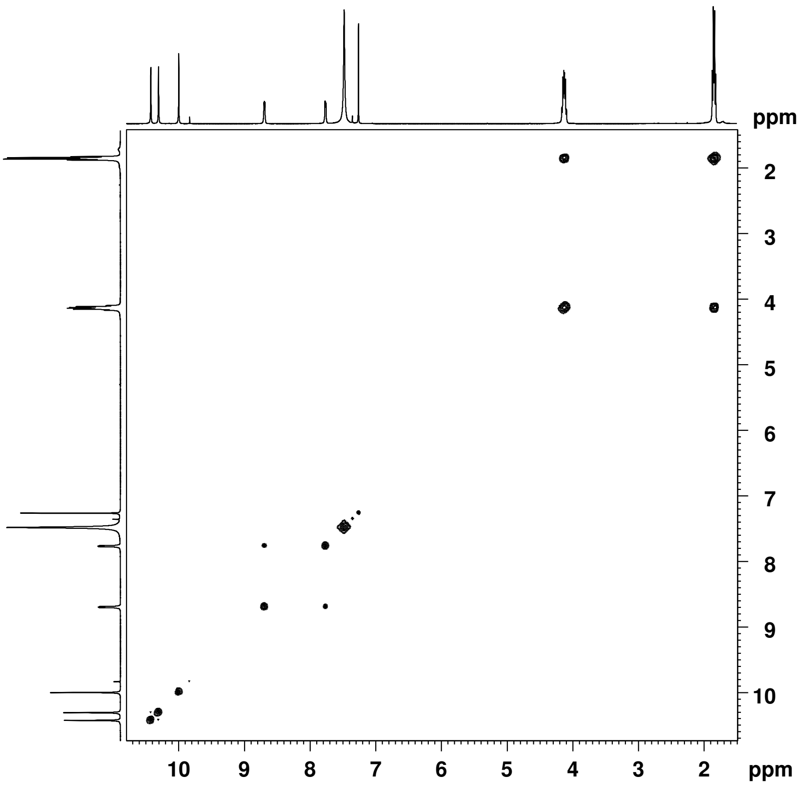

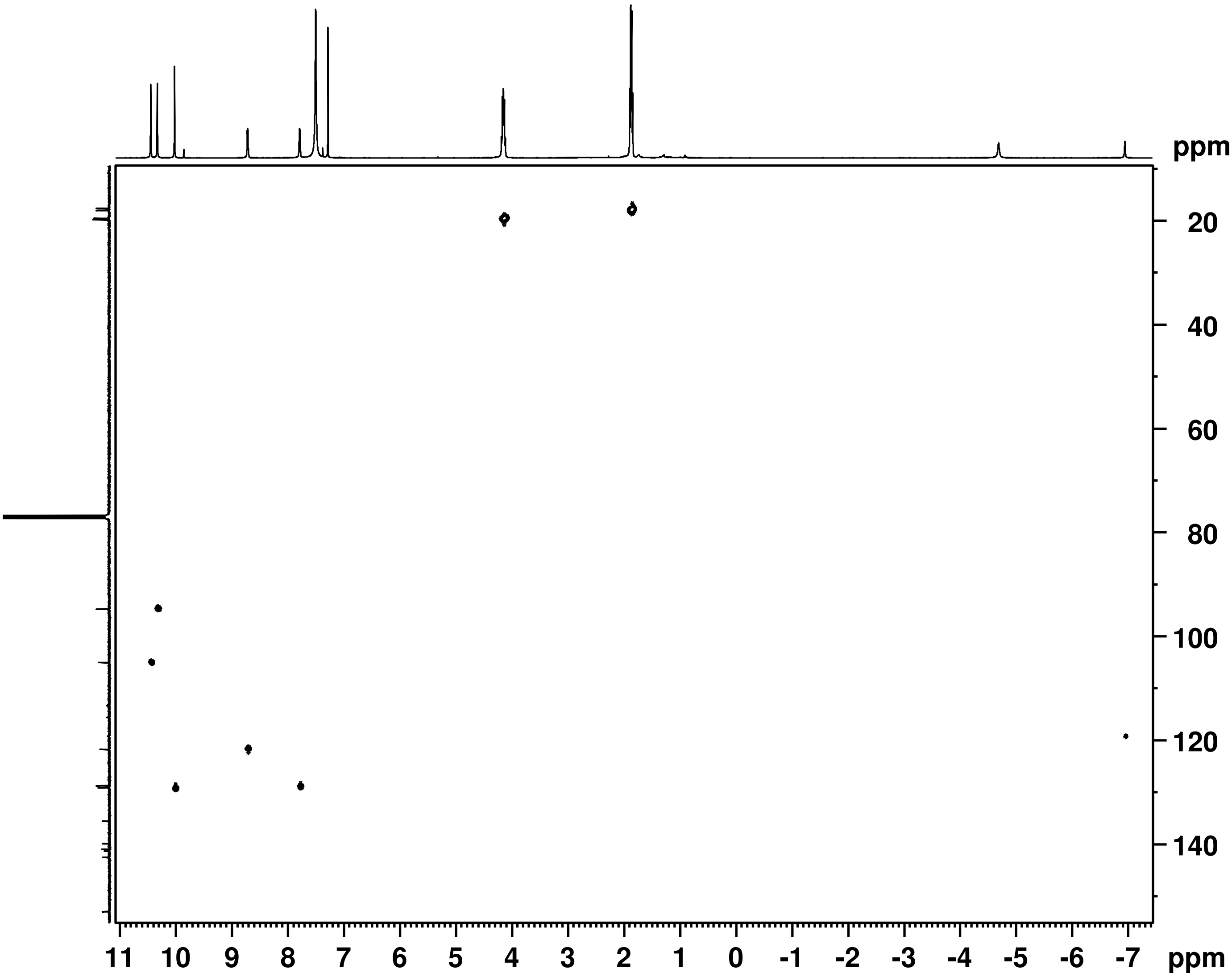

Figure S103. ^{1}H-^{1}H COSY (top) and HSQC (bottom) NMR spectra of **19b** in $CDCl_3$ with 2 μL TFA.

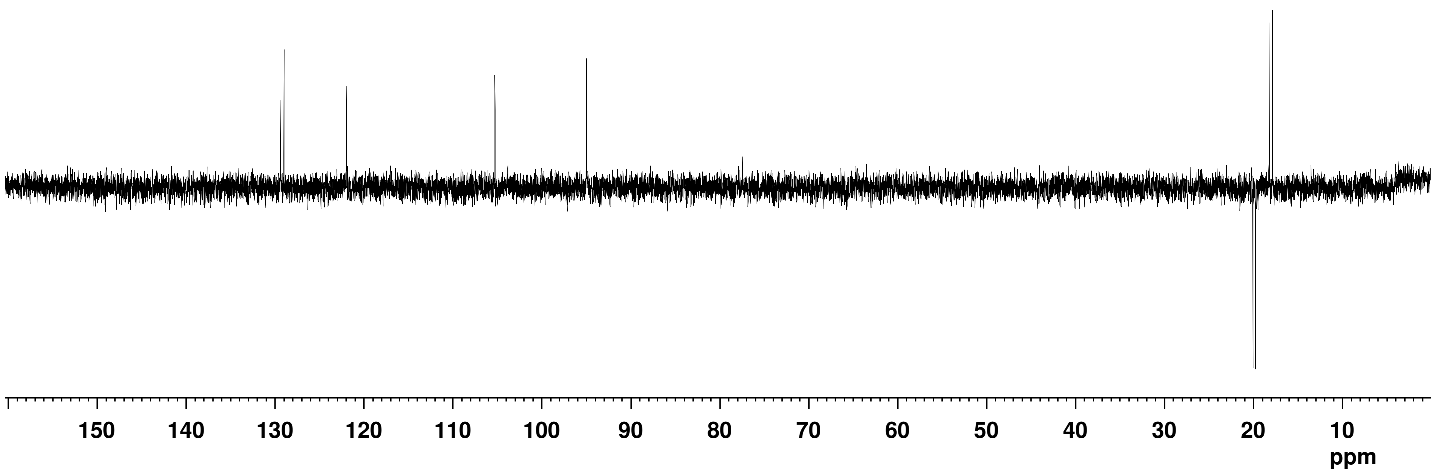

Figure S104. DEPT-135 NMR spectrum of tetraethyloxacarbaporphyrin **19b** in $CDCl_3$ with 2 μL TFA.

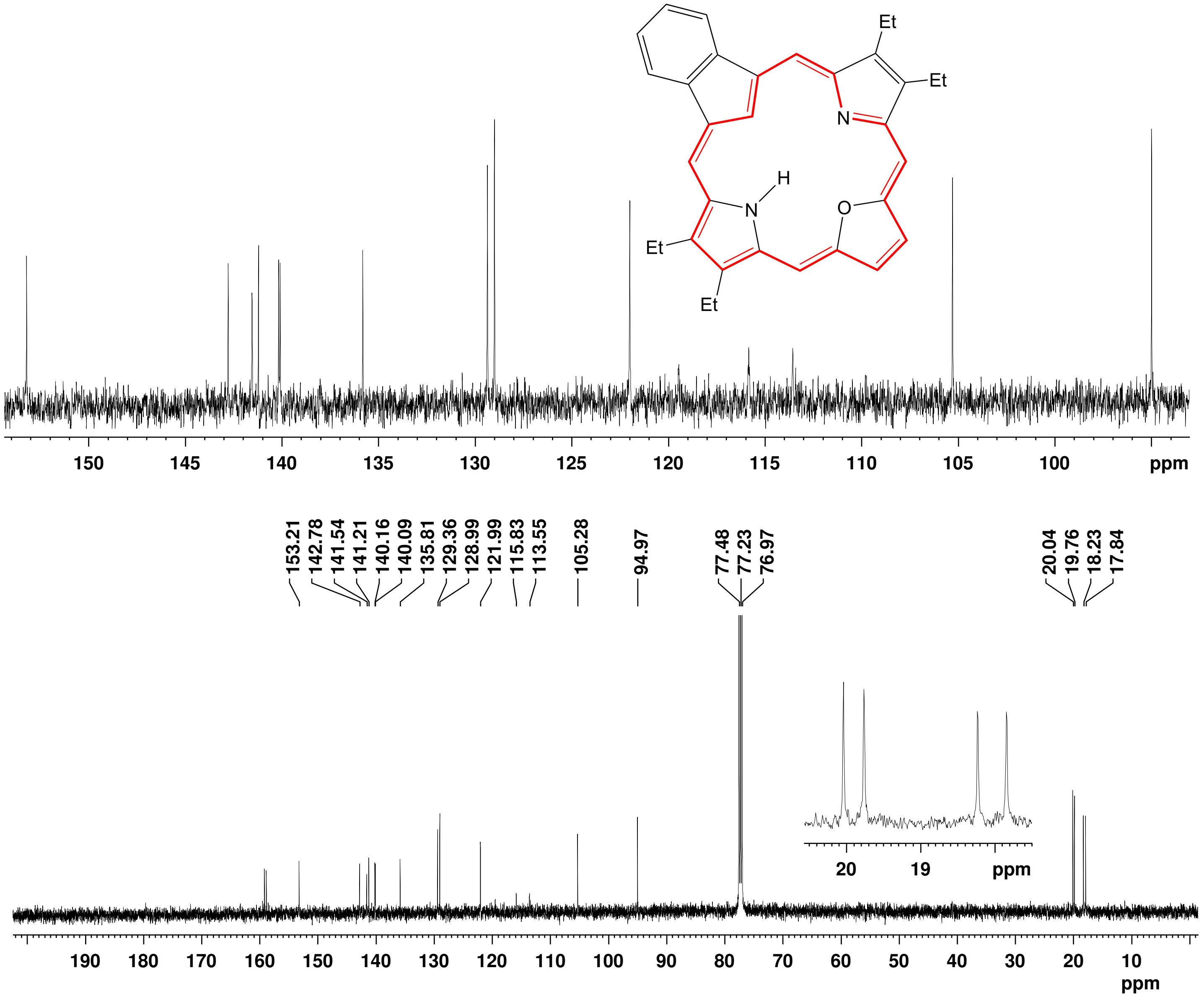

Figure S105. 125 MHz carbon-13 NMR spectrum of **19b** in $CDCl_3$ with 2 μL TFA.

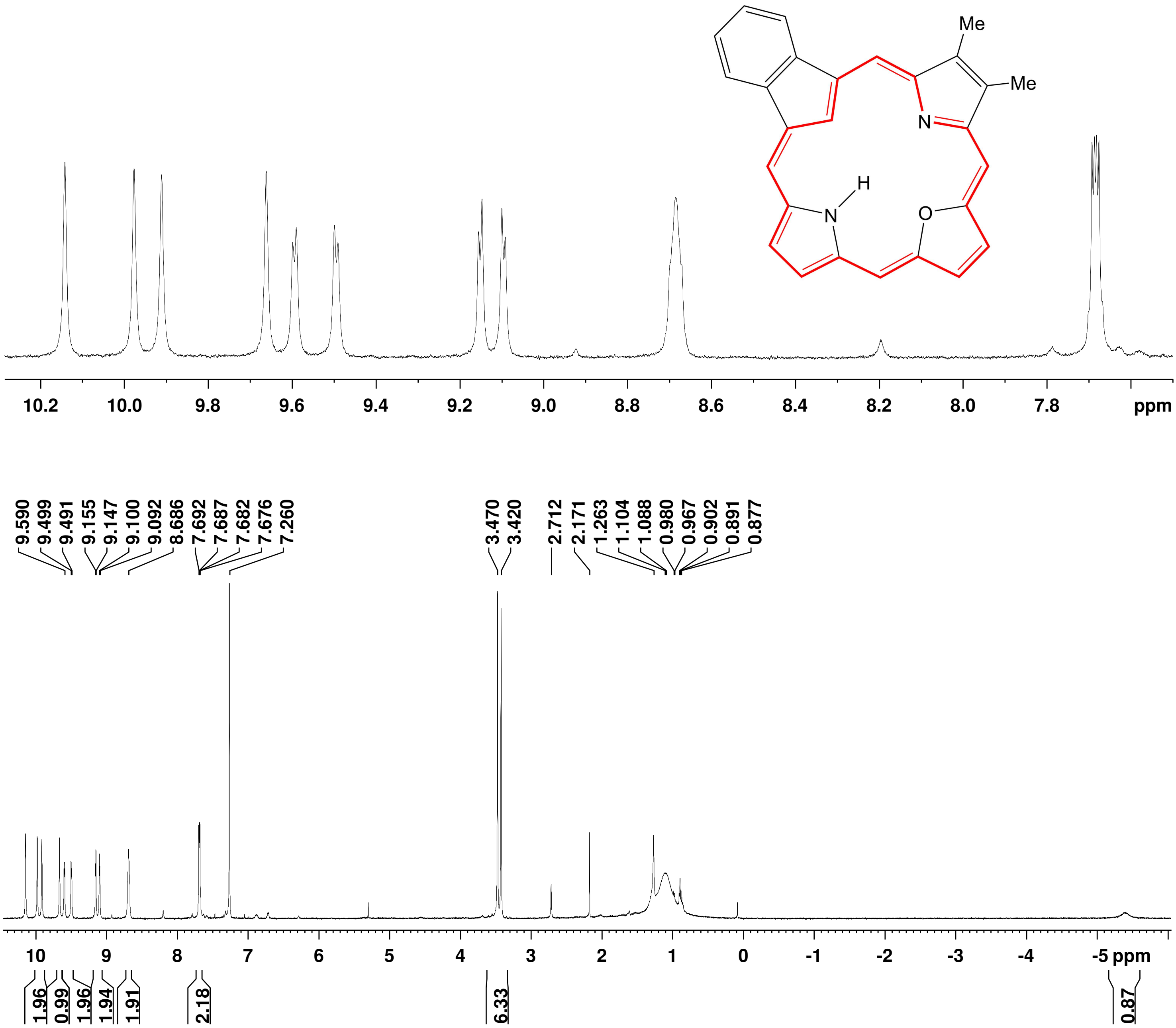

Figure S106. 500 MHz proton NMR spectrum of dimethyloxacarbaporphyrin **19c** in $CDCl_3$.

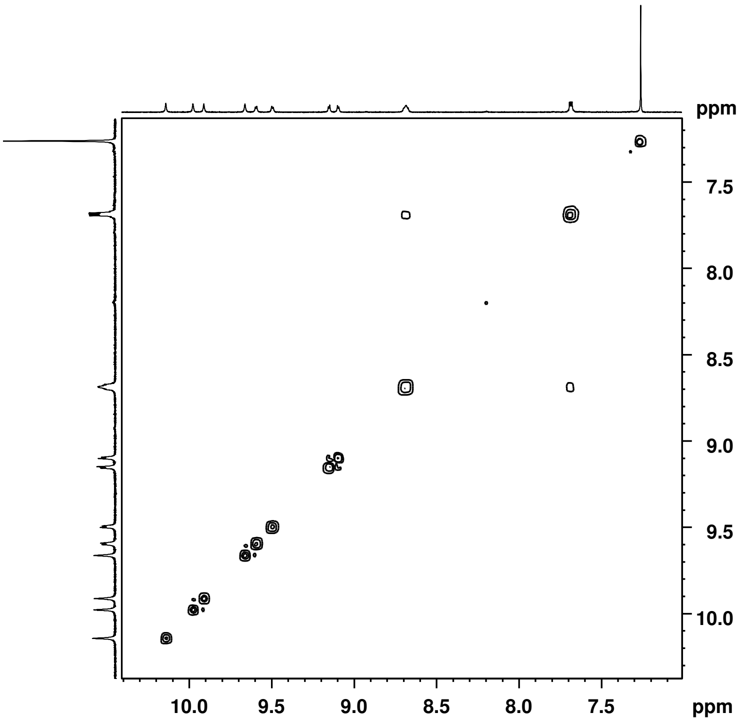

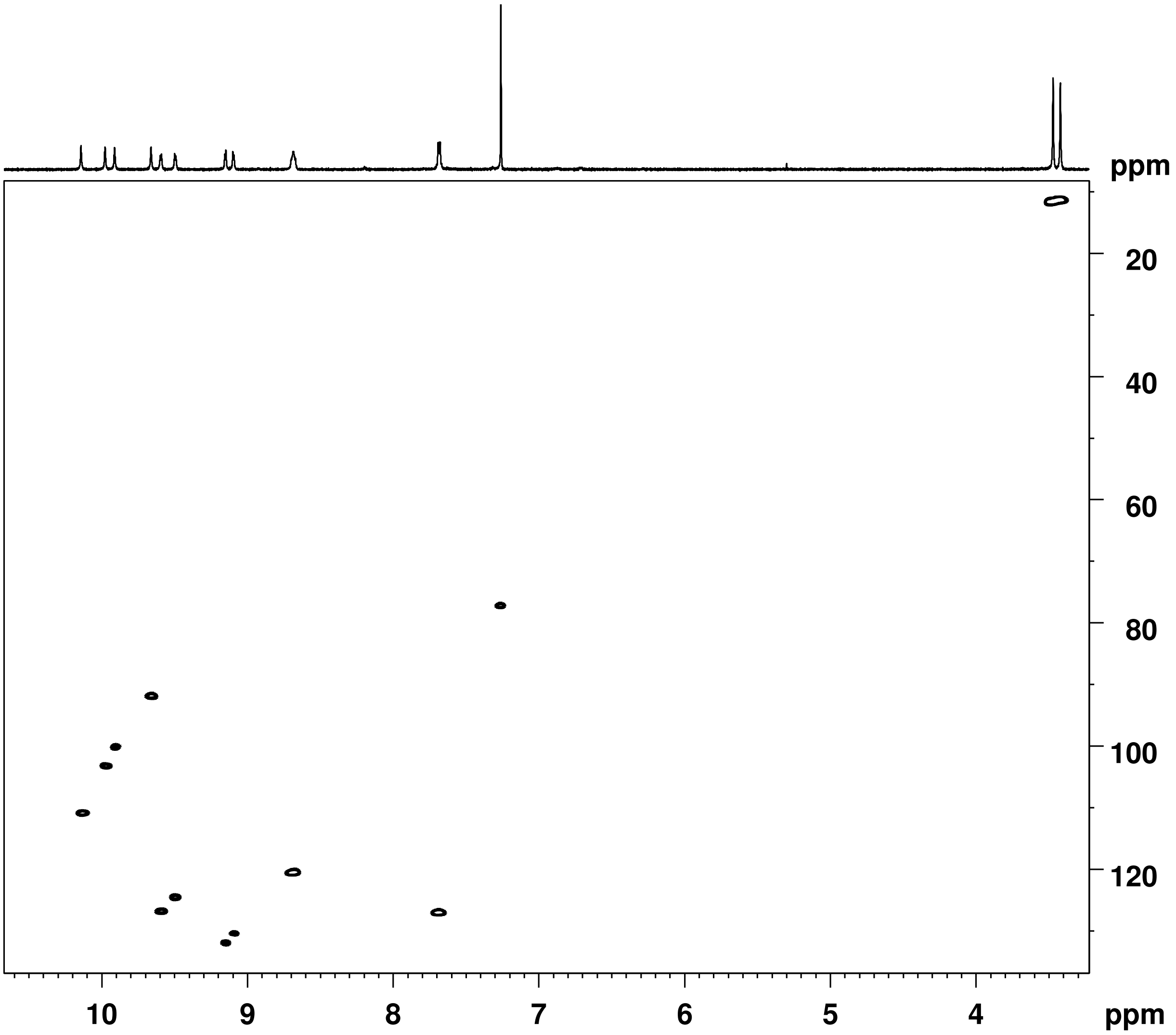

Figure S107. ^{1}H-^{1}H COSY (top) and HSQC (bottom) NMR spectra of **19c** in $CDCl_3$.

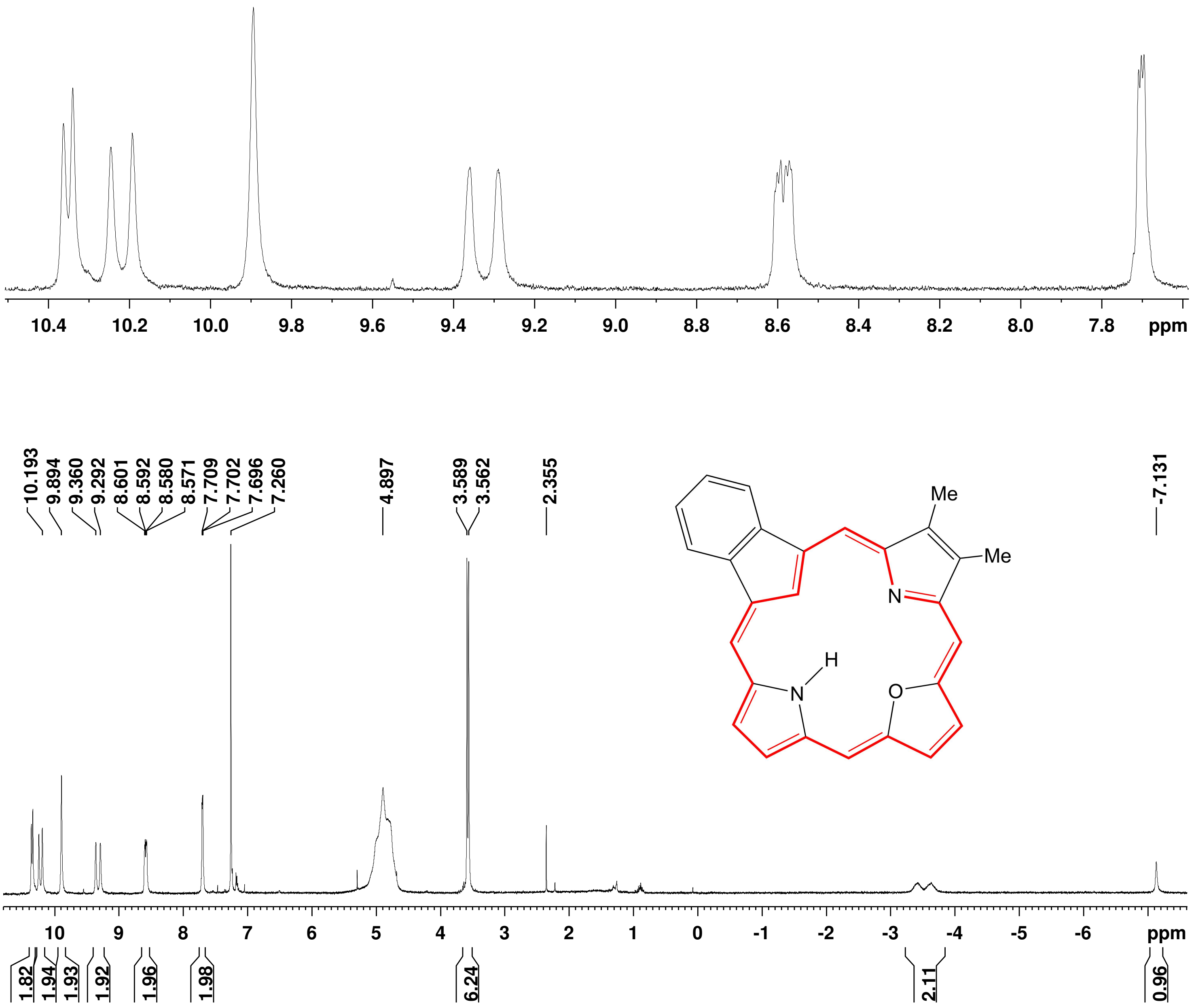

Figure S108. 500 MHz proton NMR spectrum of oxacarbaporphyrin **19c** in $CDCl_3$ with 2 μL TFA.

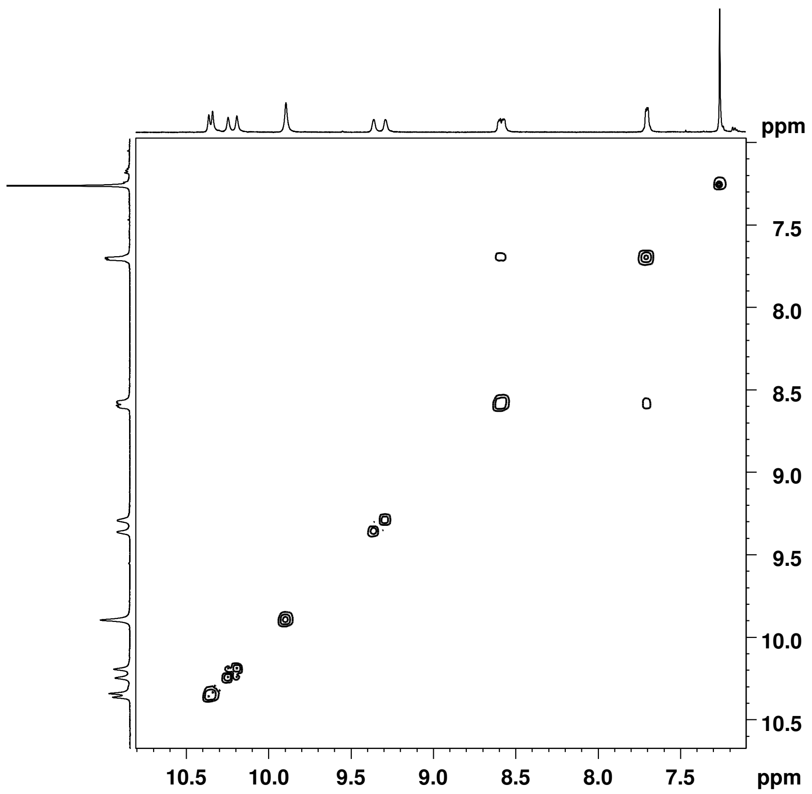

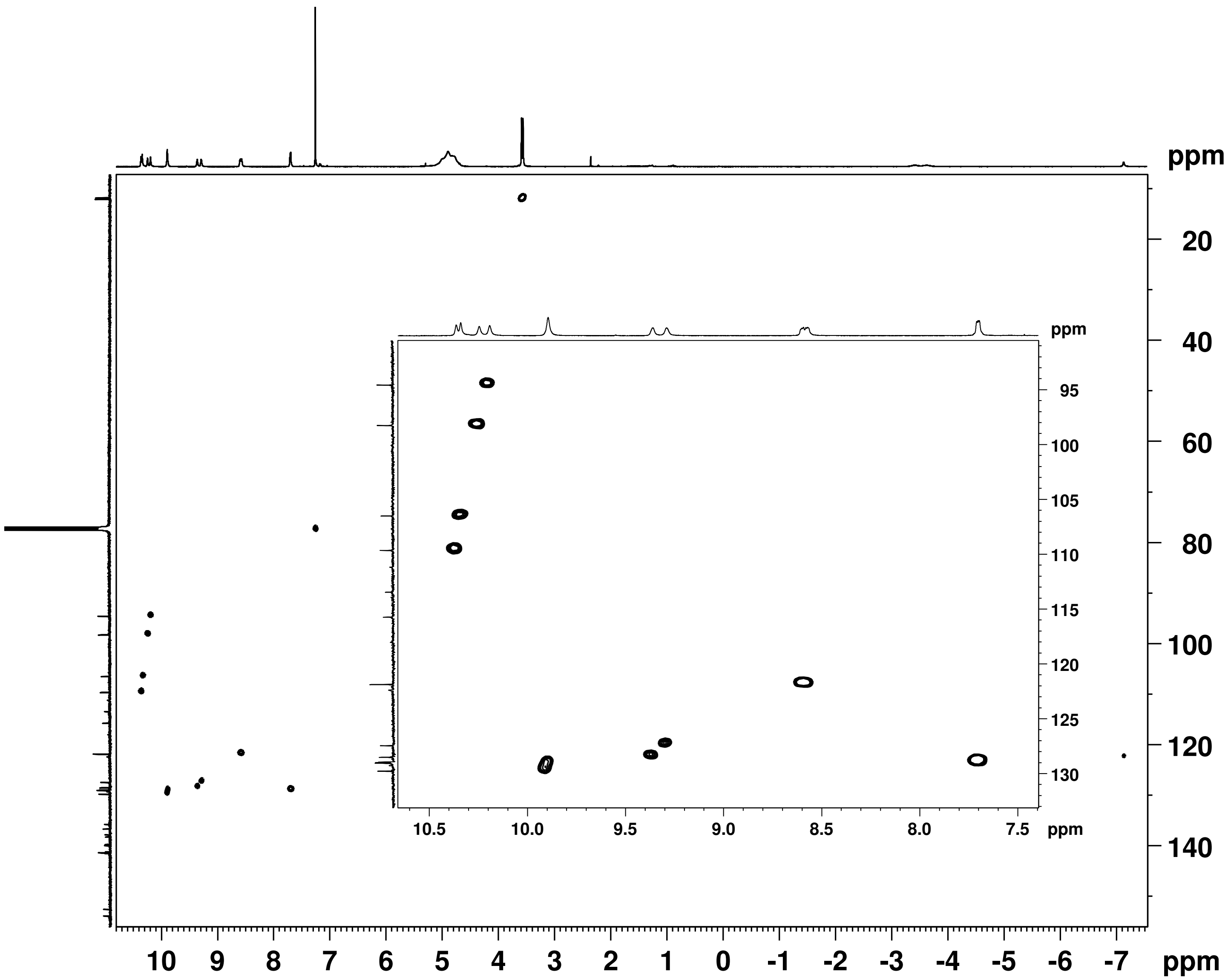

Figure S109. ^{1}H-^{1}H COSY (top) and HSQC (bottom) NMR spectra of **19c** in $CDCl_3$ with 2 μL TFA.

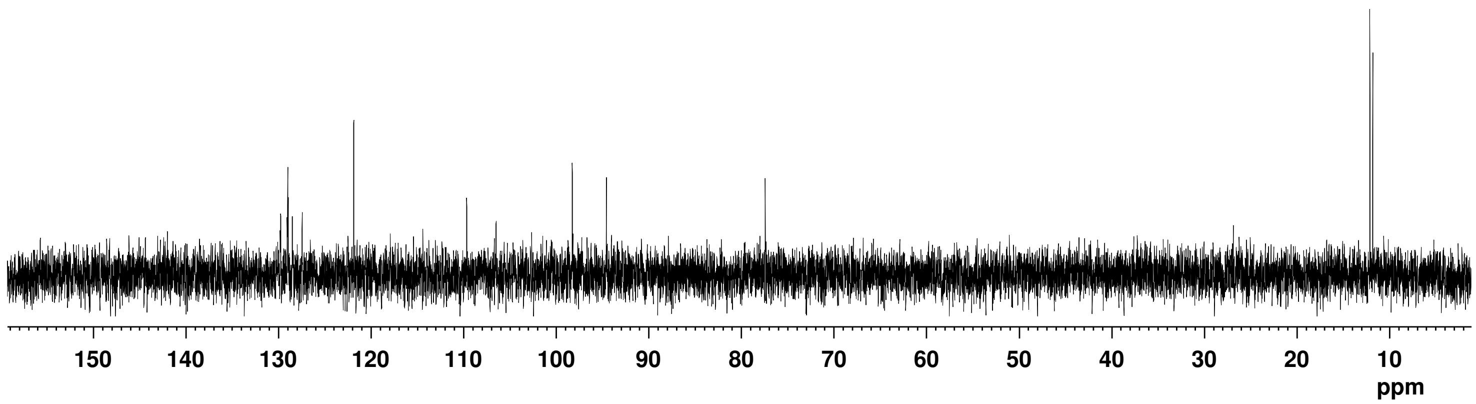

Figure S110. DEPT-135 NMR spectrum of oxacarbaporphyrin **19c** in CDCl3 with 2 μL TFA.

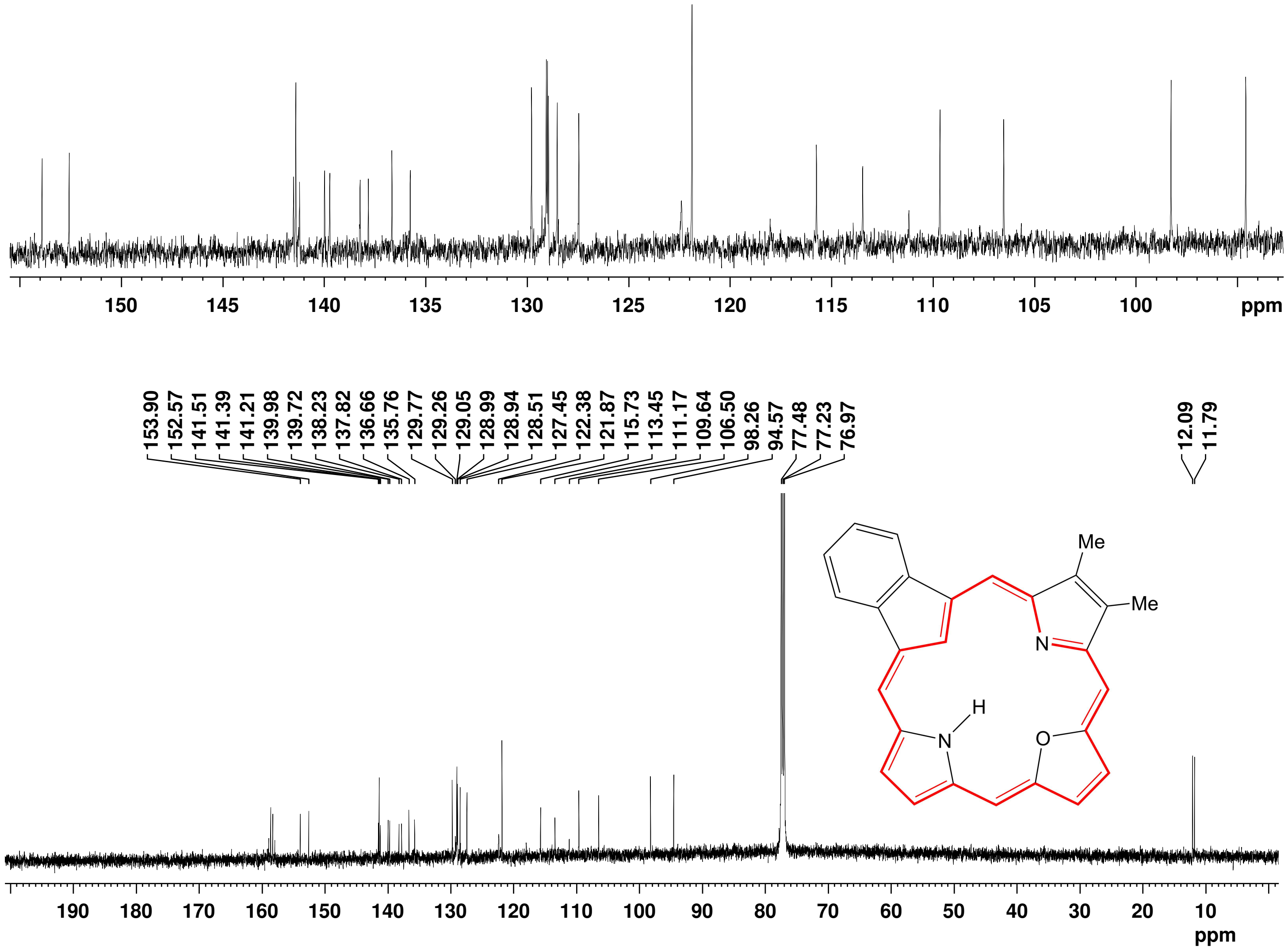

Figure S111. 125 MHz carbon-13 NMR spectrum of oxacarbaporphyrin **19c** in $CDCl_3$ with 2 μL TFA.

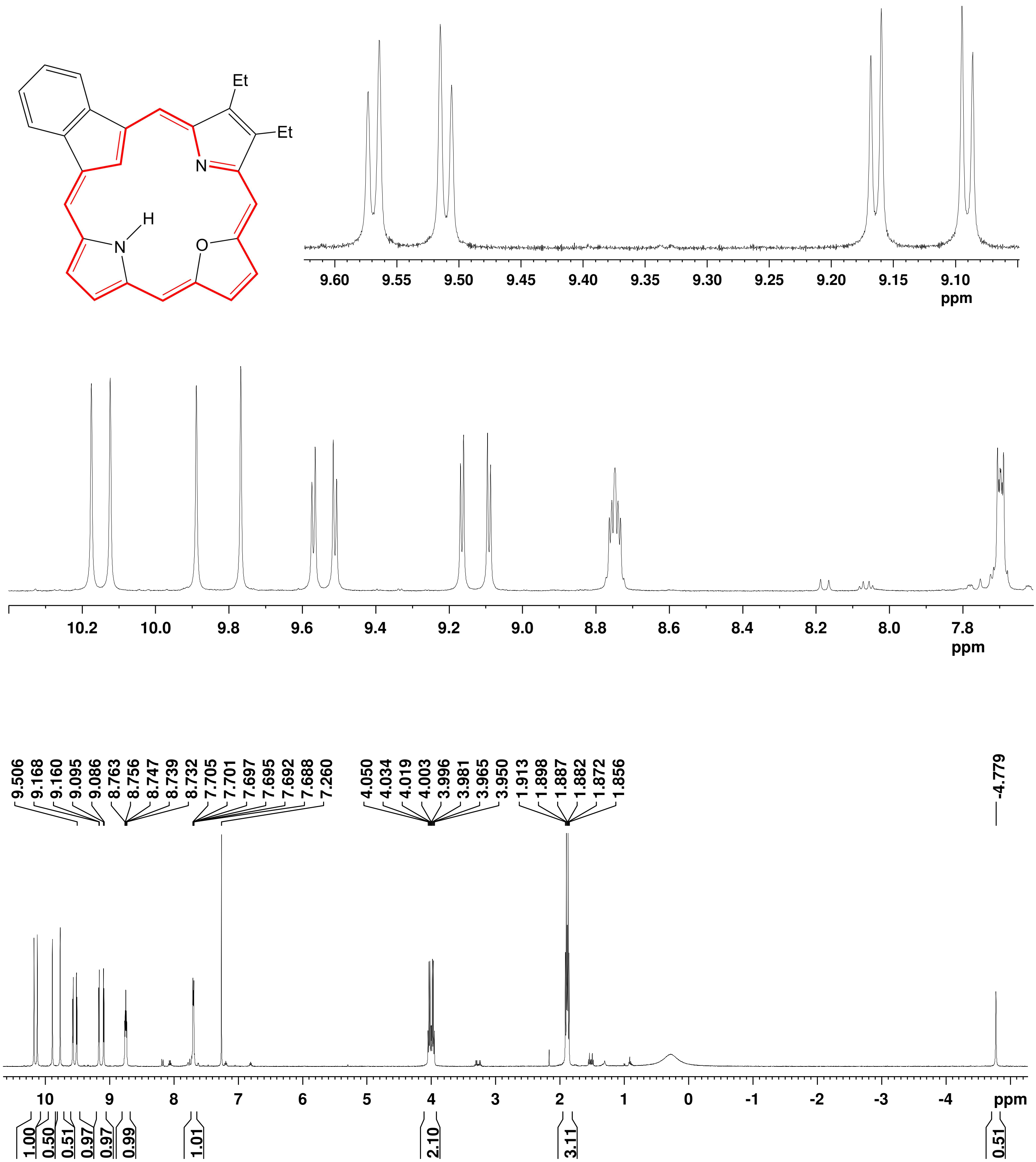

Figure S112. 500 MHz proton NMR spectrum of diethyloxacarbaporphyrin **19d** in $CDCl_3$.

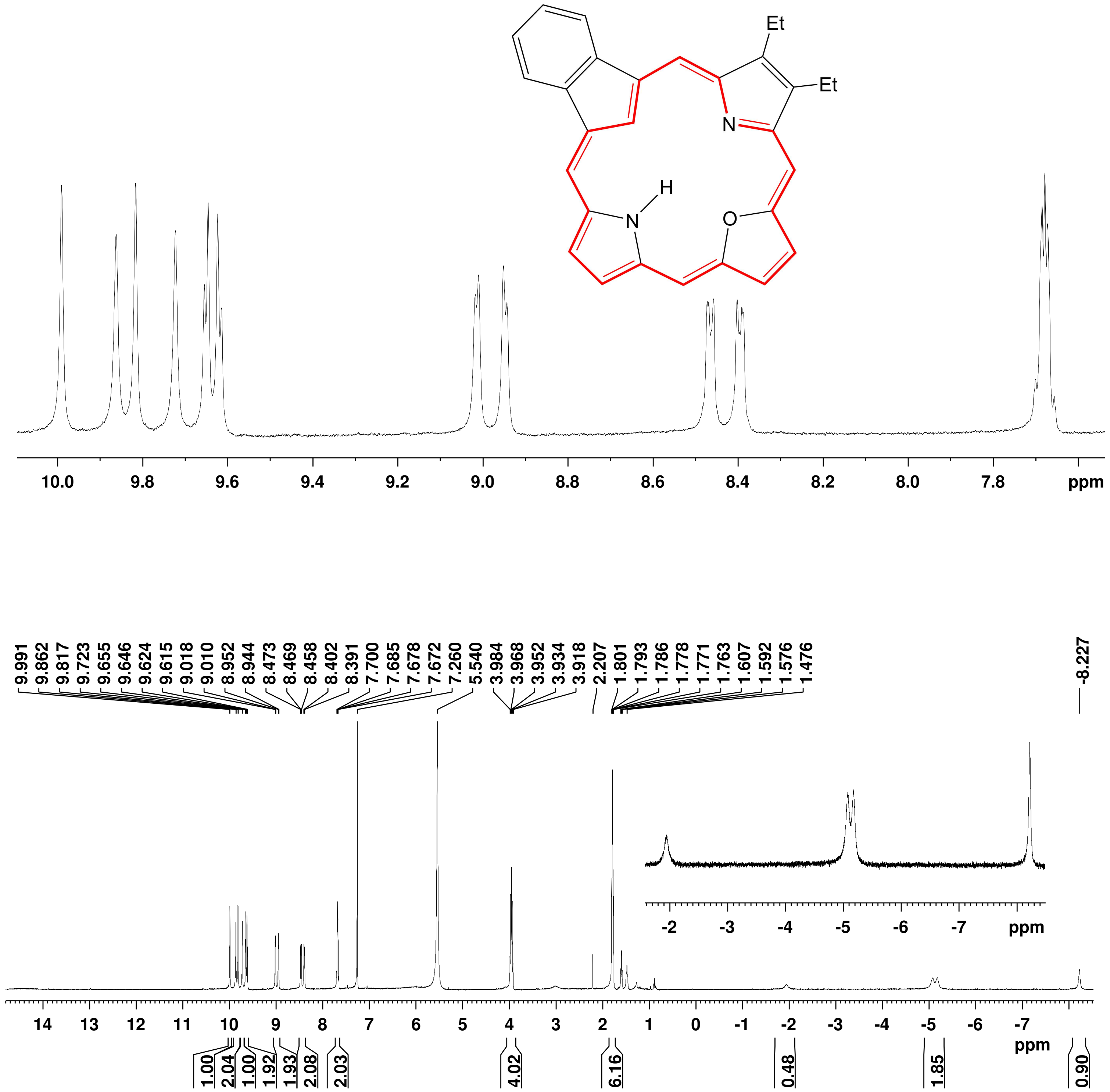

Figure S113. 500 MHz proton NMR spectrum of diethyloxacarbaporphyrin **19d** in $CDCl_3$ with 2 μL TFA.

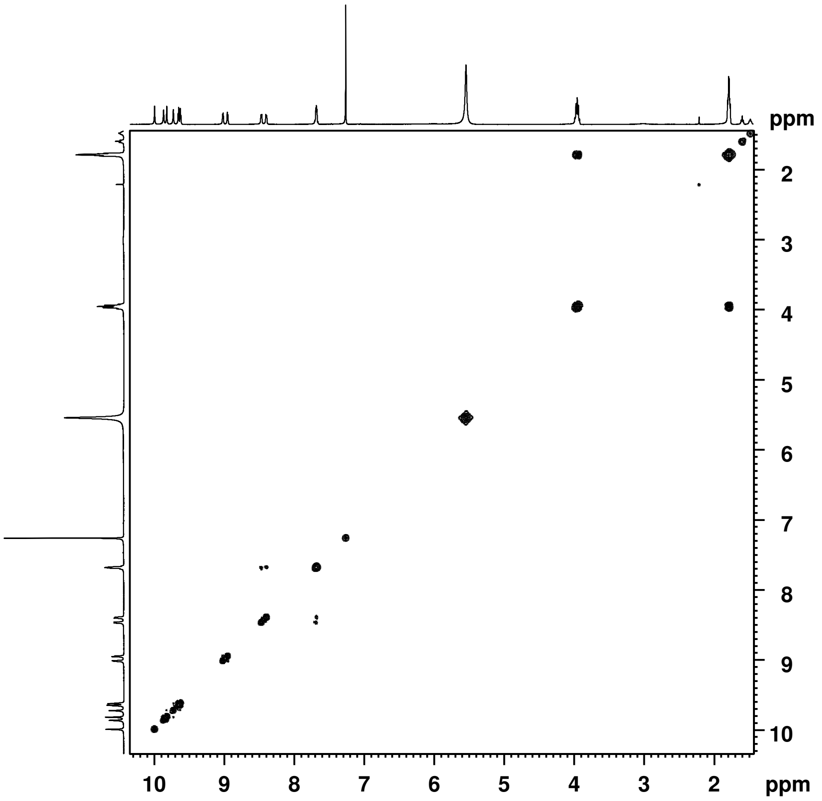

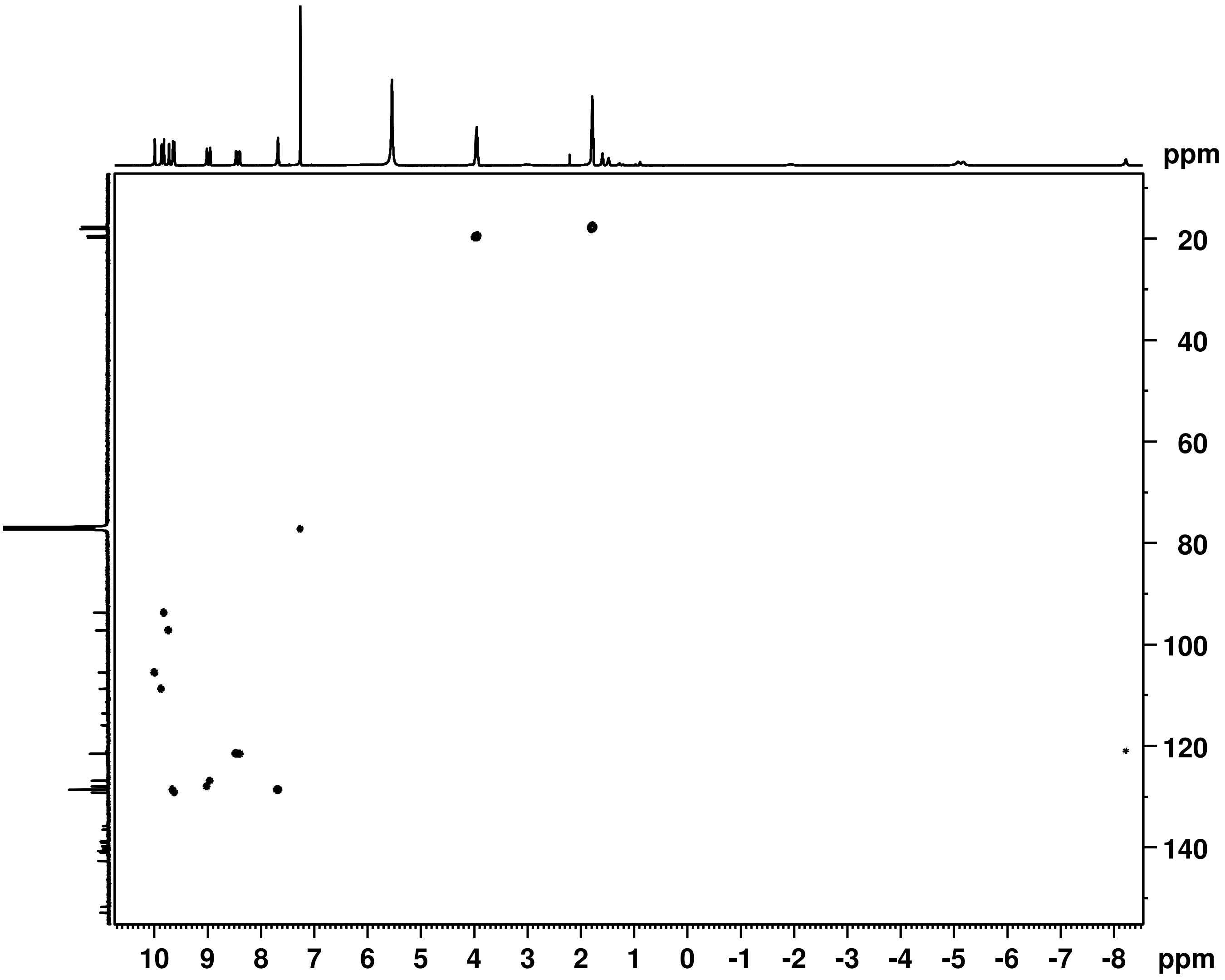

Figure S114. ^{1}H-^{1}H COSY (top) and HSQC (bottom) NMR spectra of **19d** in $CDCl_3$ with 2 μL TFA.

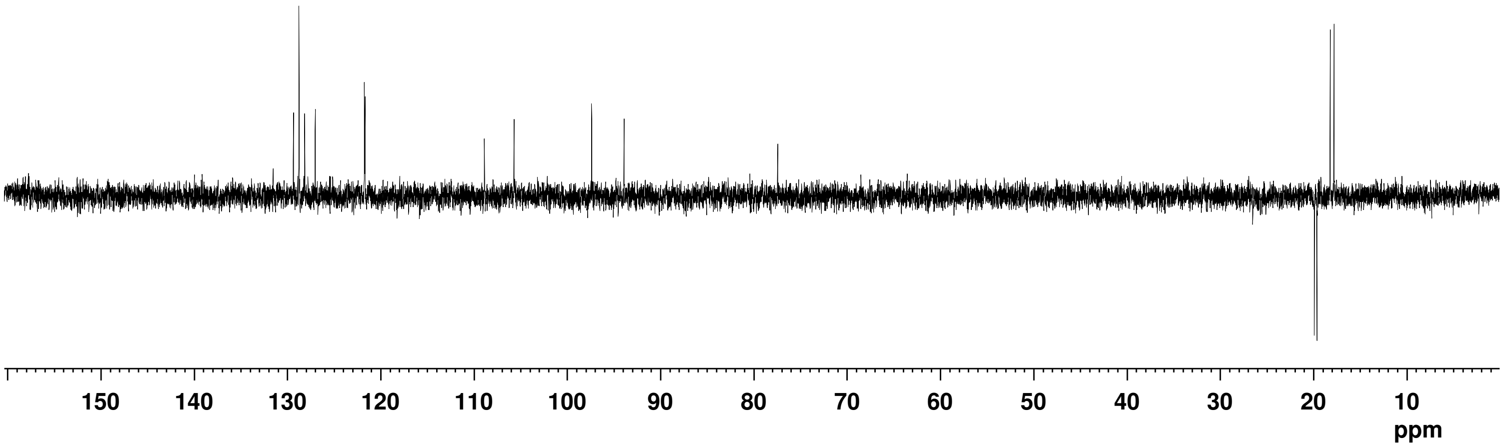

Figure S115. DEPT-135 NMR spectrum of diethyloxacarbaporphyrin **19d** in $CDCl_3$ with 2 μL TFA.

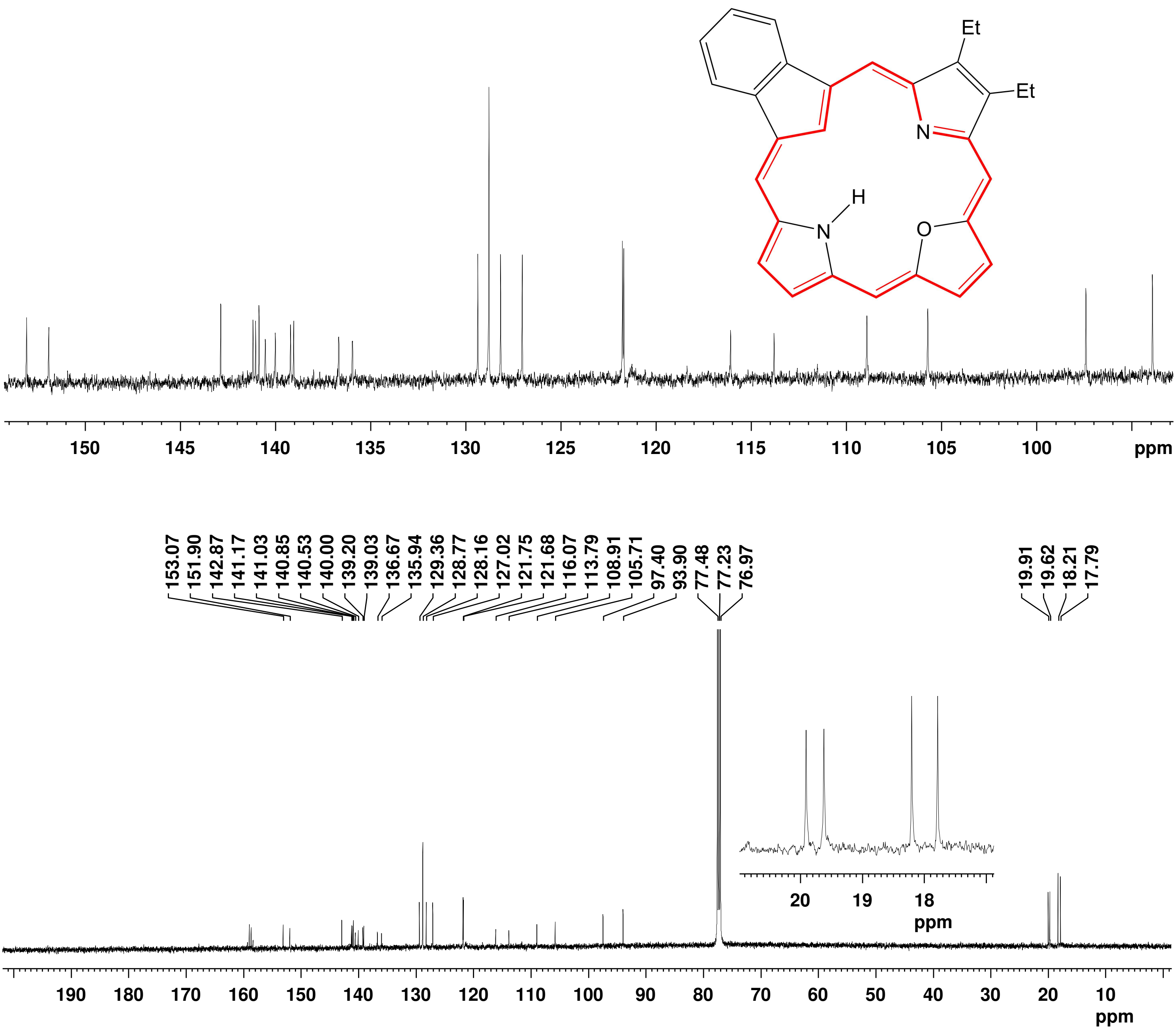

Figure S116. 125 MHz carbon-13 NMR spectrum of diethyloxacarbaporphyrin **19d** in $CDCl_3$ with 2 μL TFA.

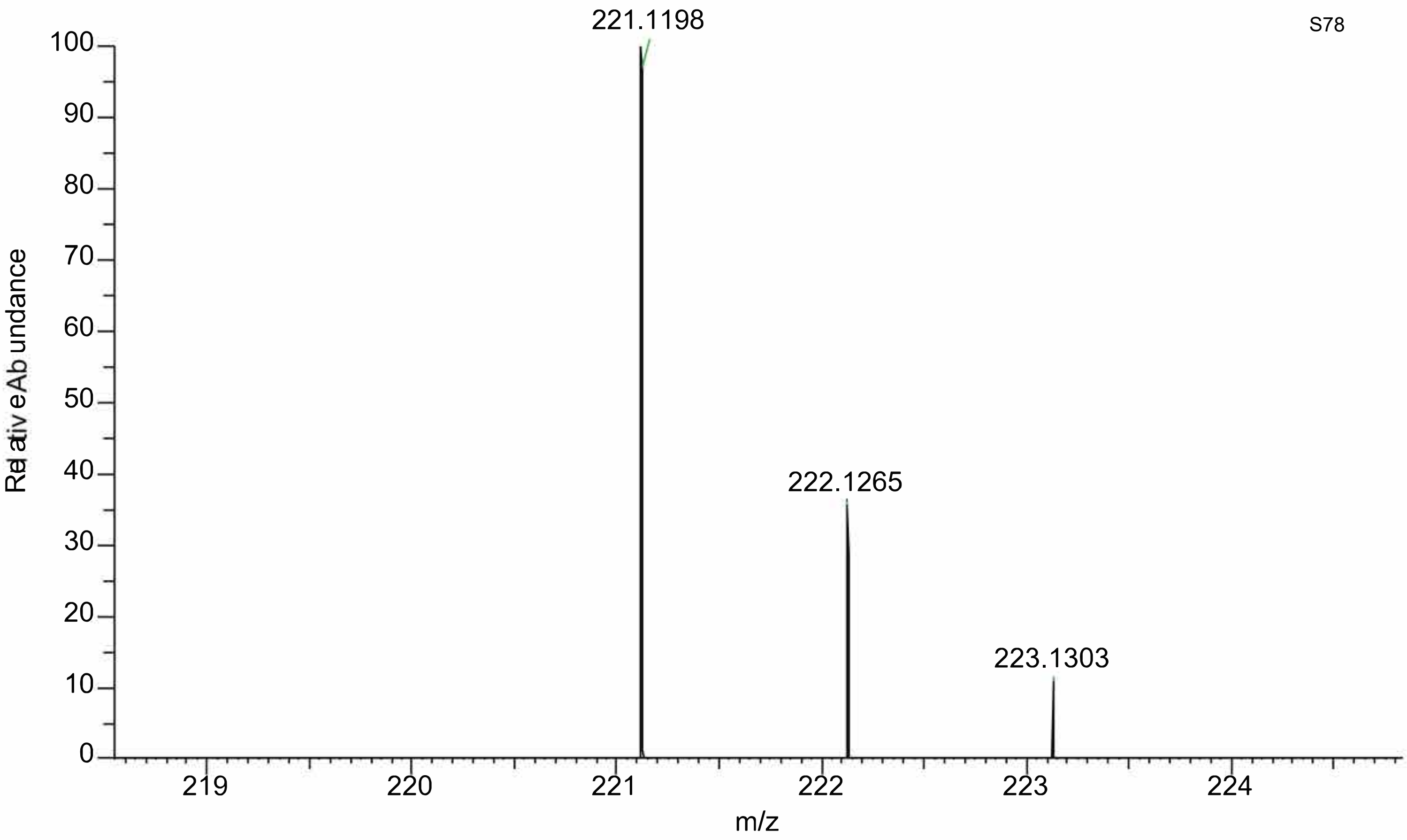

Figure S117. High resolution TOF-ESI mass spectrum of fulvene **16a**.

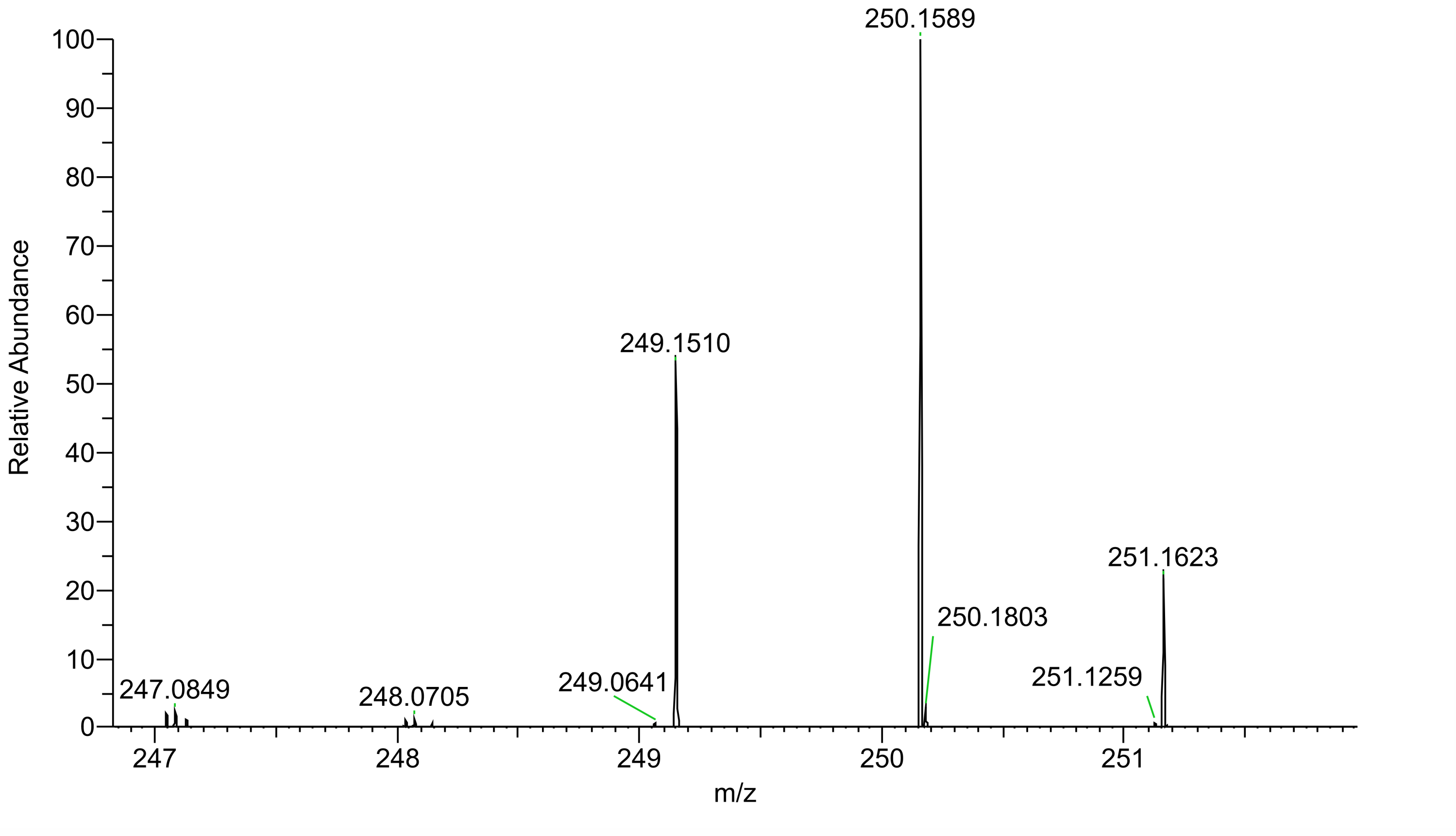

Figure S118. High resolution TOF-ESI mass spectrum of fulvene **16b**.

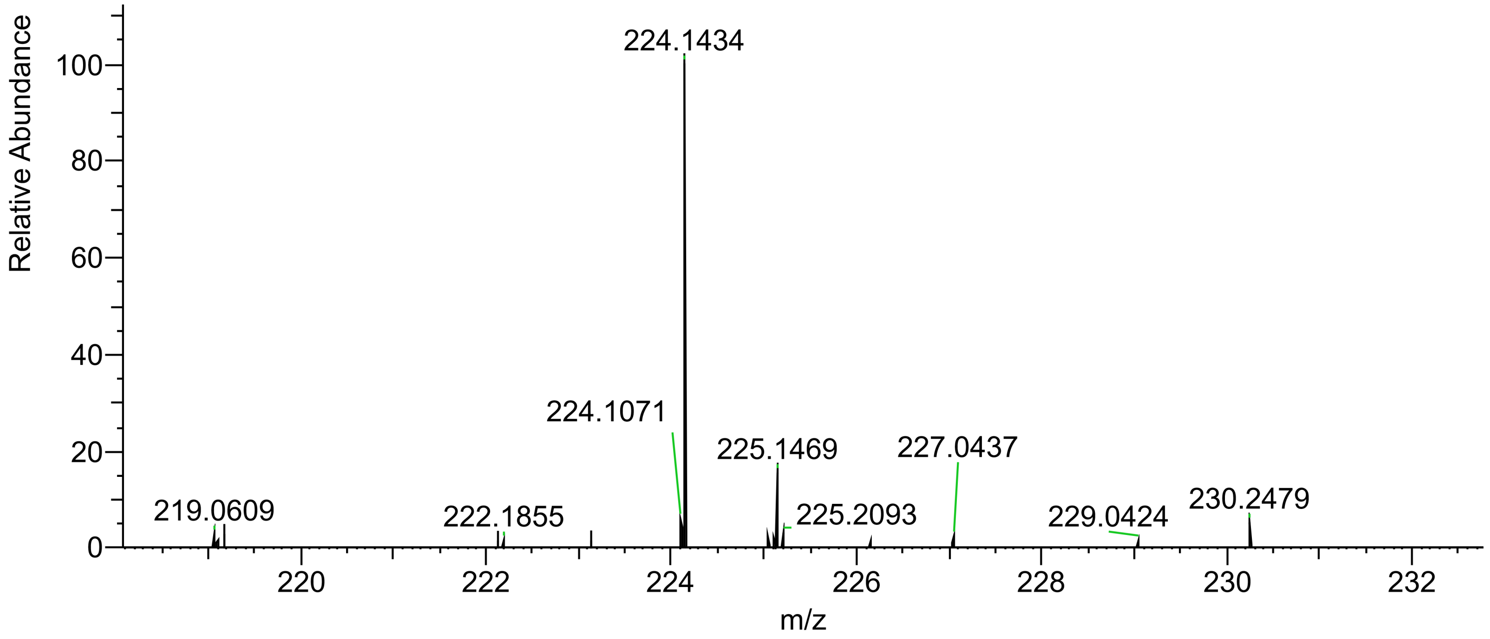

Figure S119. High resolution TOF-ESI mass spectrum of dihydrofulvene **17a**.

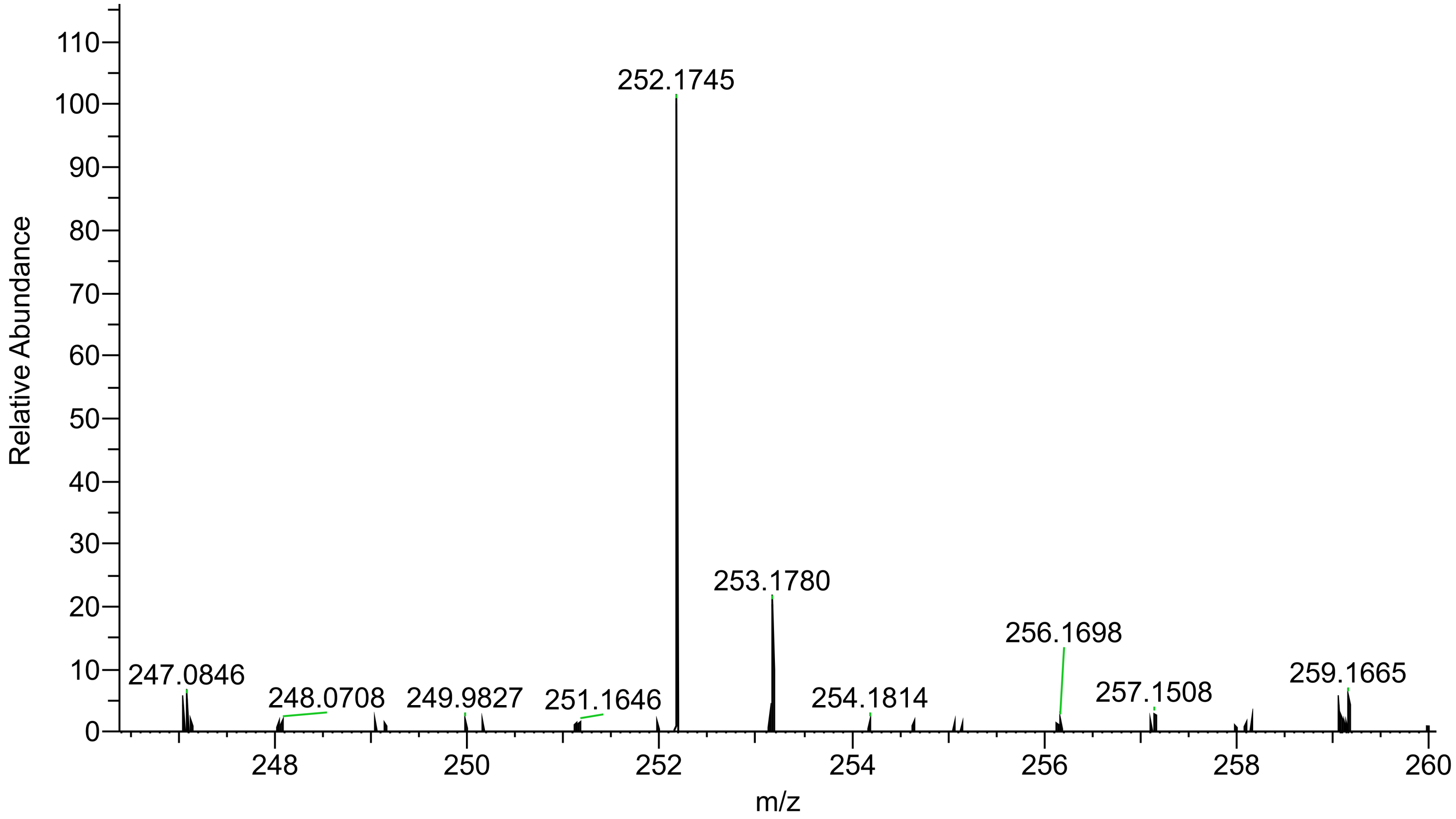

Figure S120. High resolution TOF-ESI mass spectrum of dihydrofulvene **17b**.

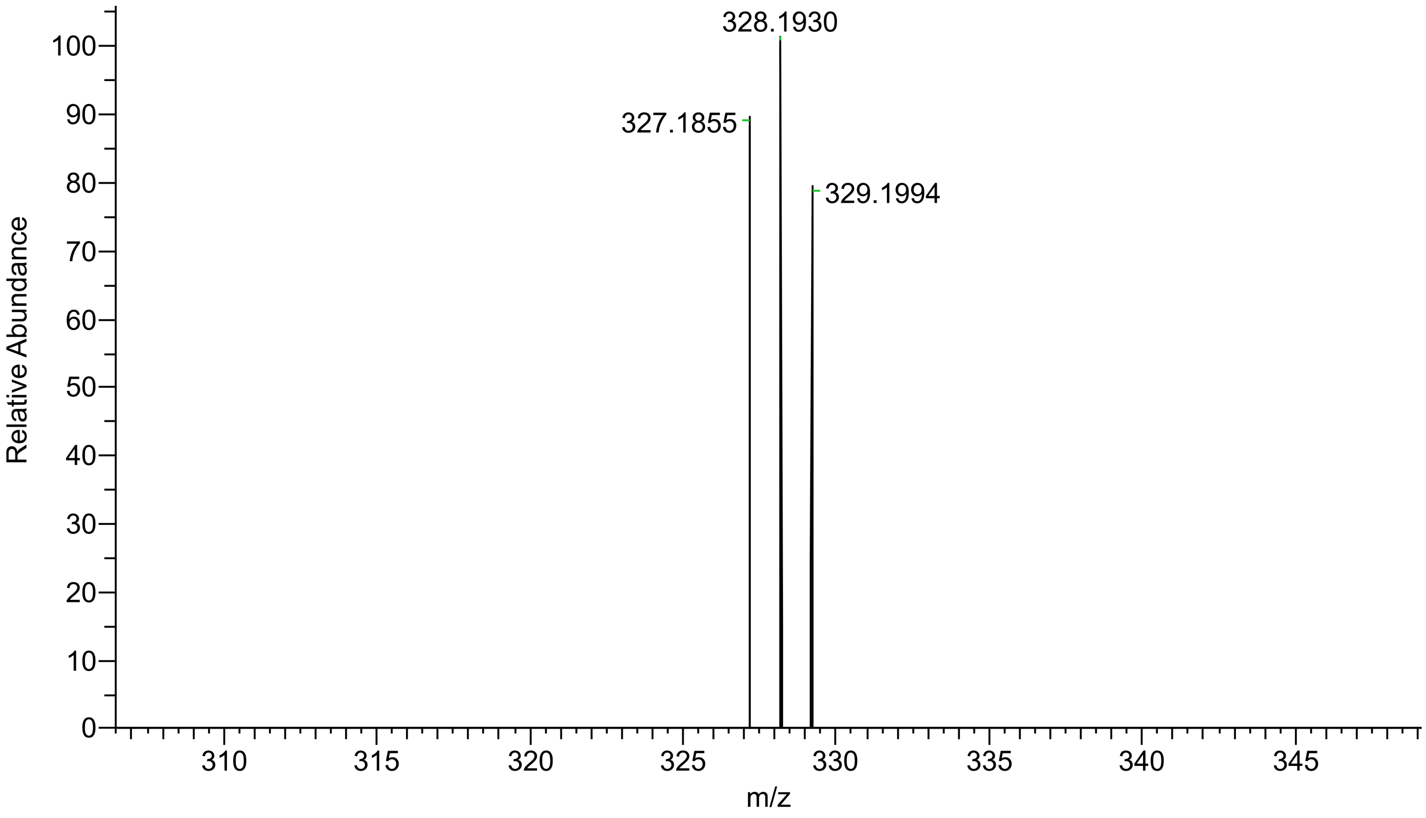

Figure S121. High resolution TOF-ESI mass spectrum of carbatripyrrin **14a**.

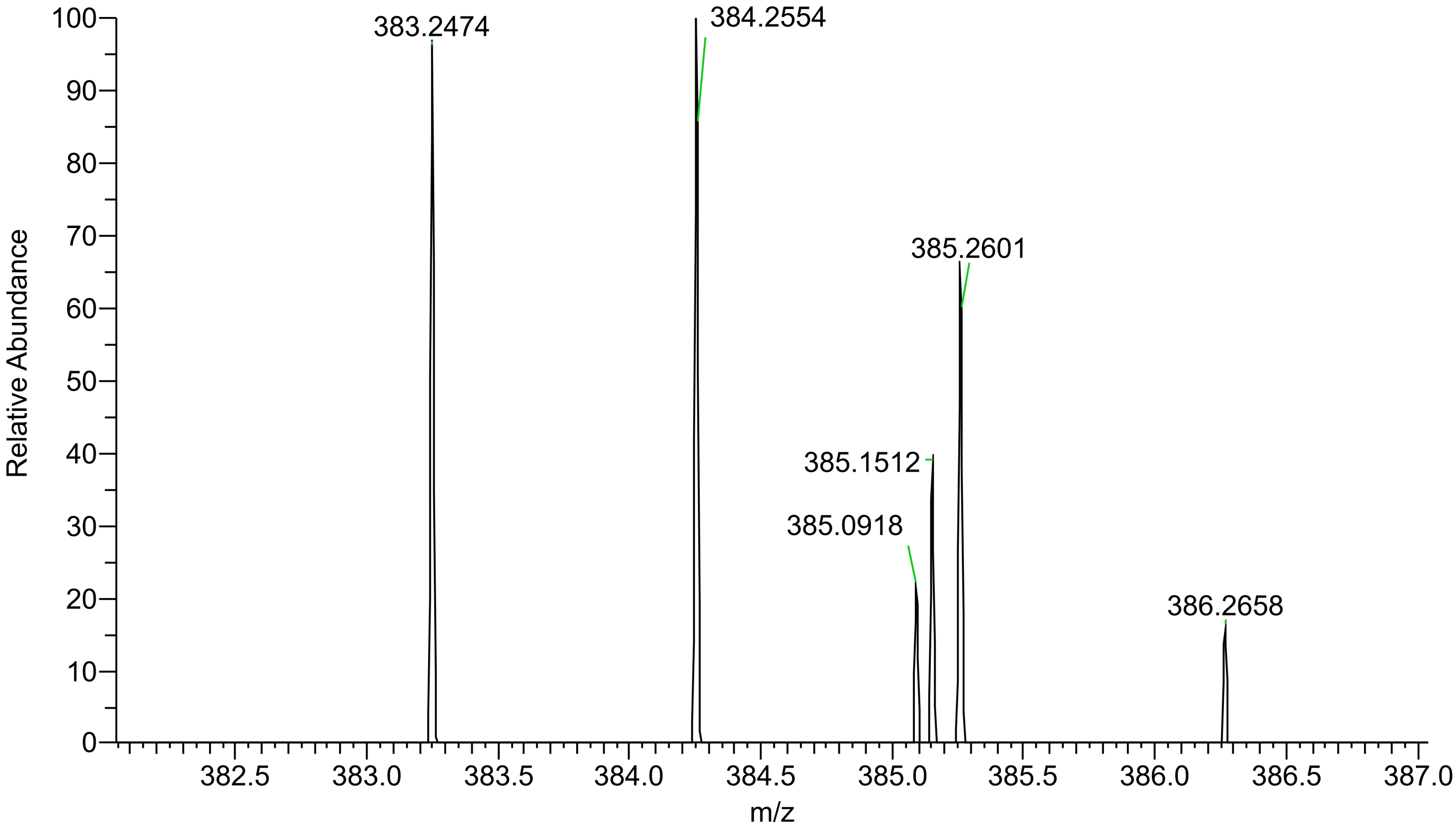

Figure S122. High resolution TOF-ESI mass spectrum of carbatripyrrin **14b.**

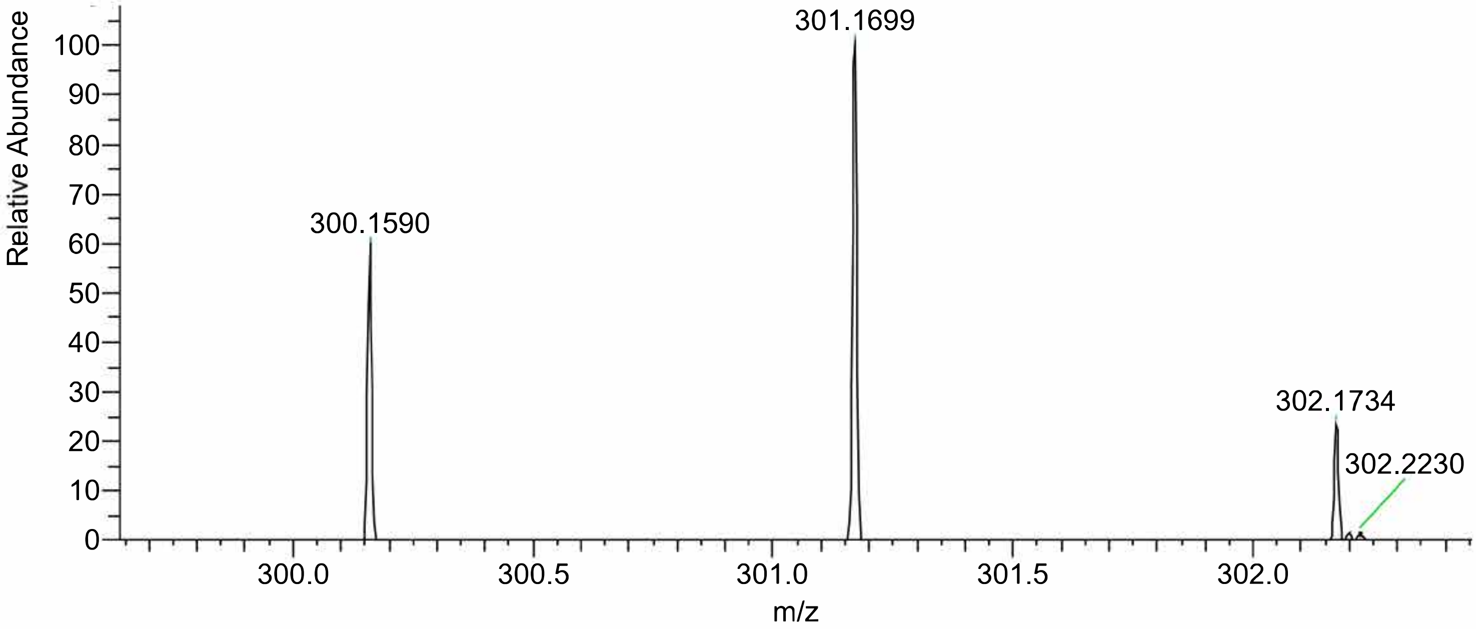

Figure S123. High resolution TOF-ESI mass spectrum of carbatripyrrin **14c**.

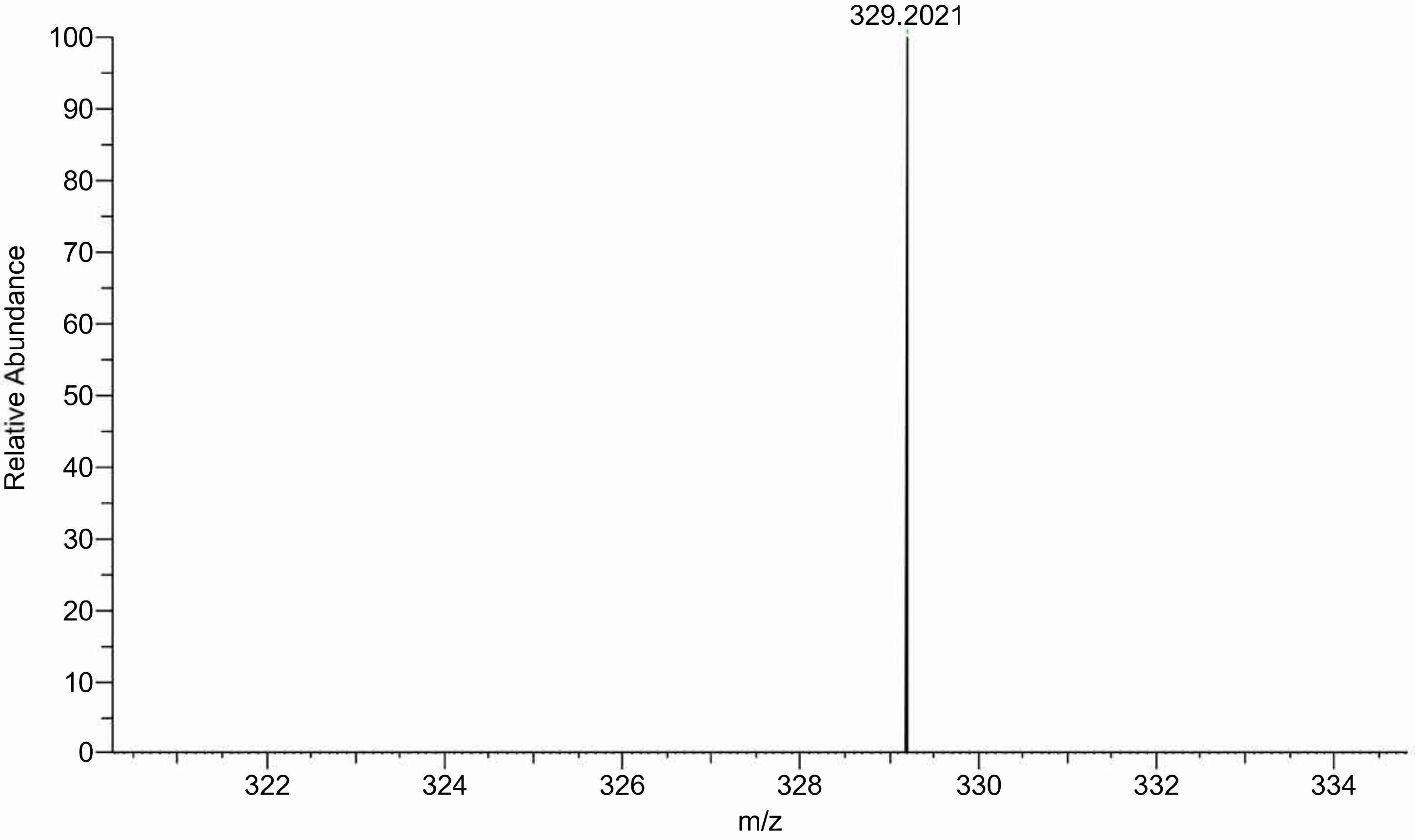

Figure S124. High resolution TOF-ESI mass spectrum of carbatripyrrin **14d**.

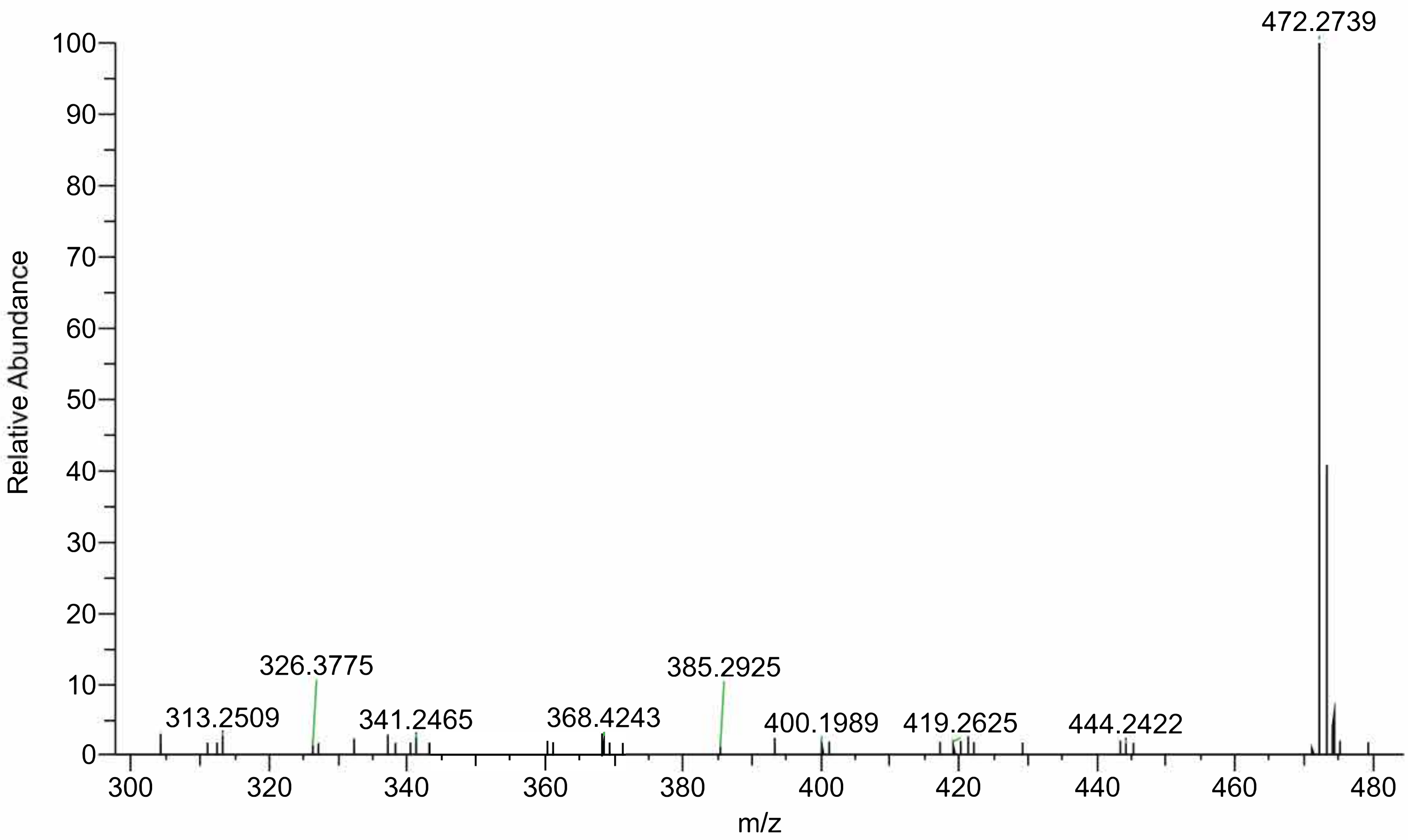

Figure S125. High resolution TOF-ESI mass spectrum of carbaporphyrin **18a**.

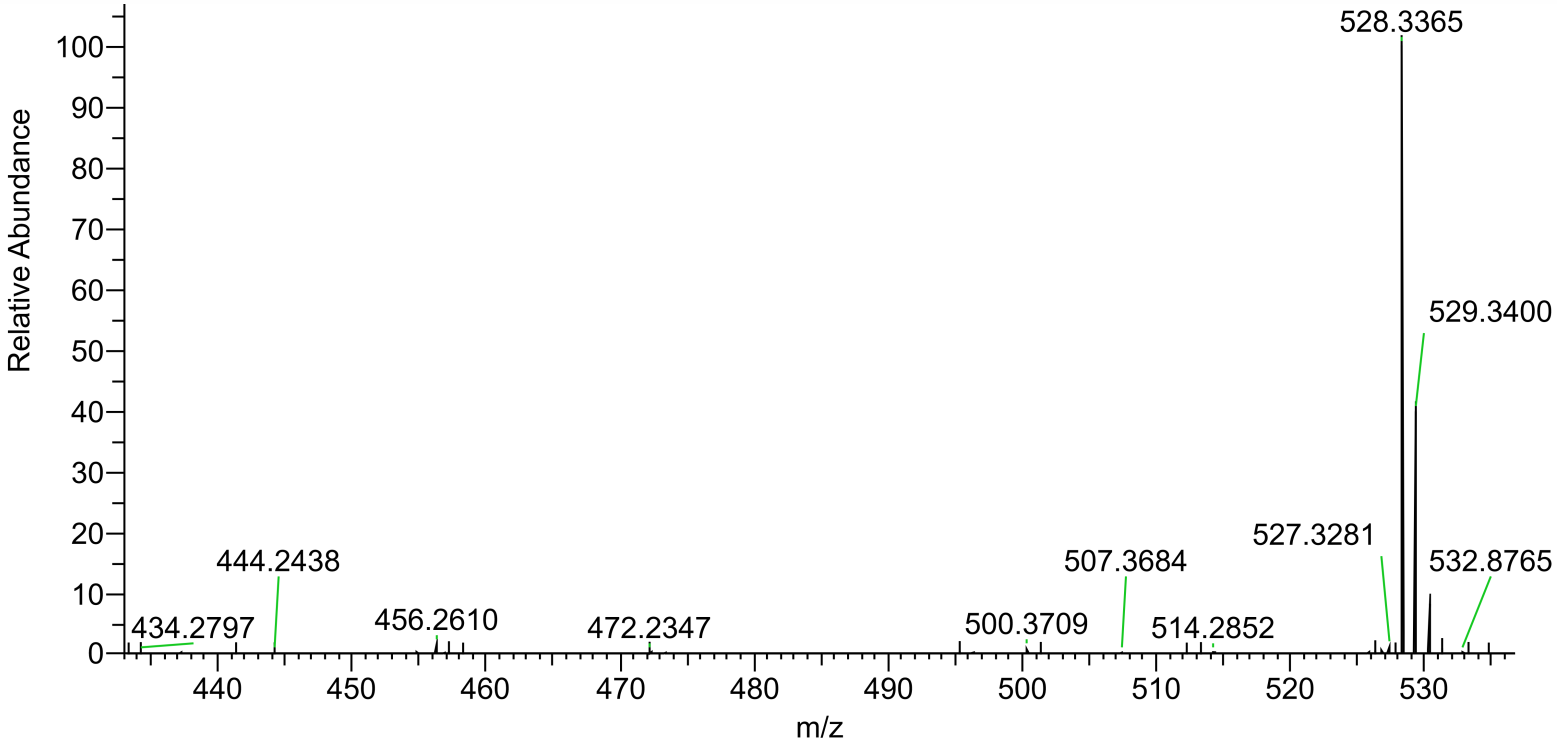

Figure S126. High resolution TOF-ESI mass spectrum of carbaporphyrin **18b**.

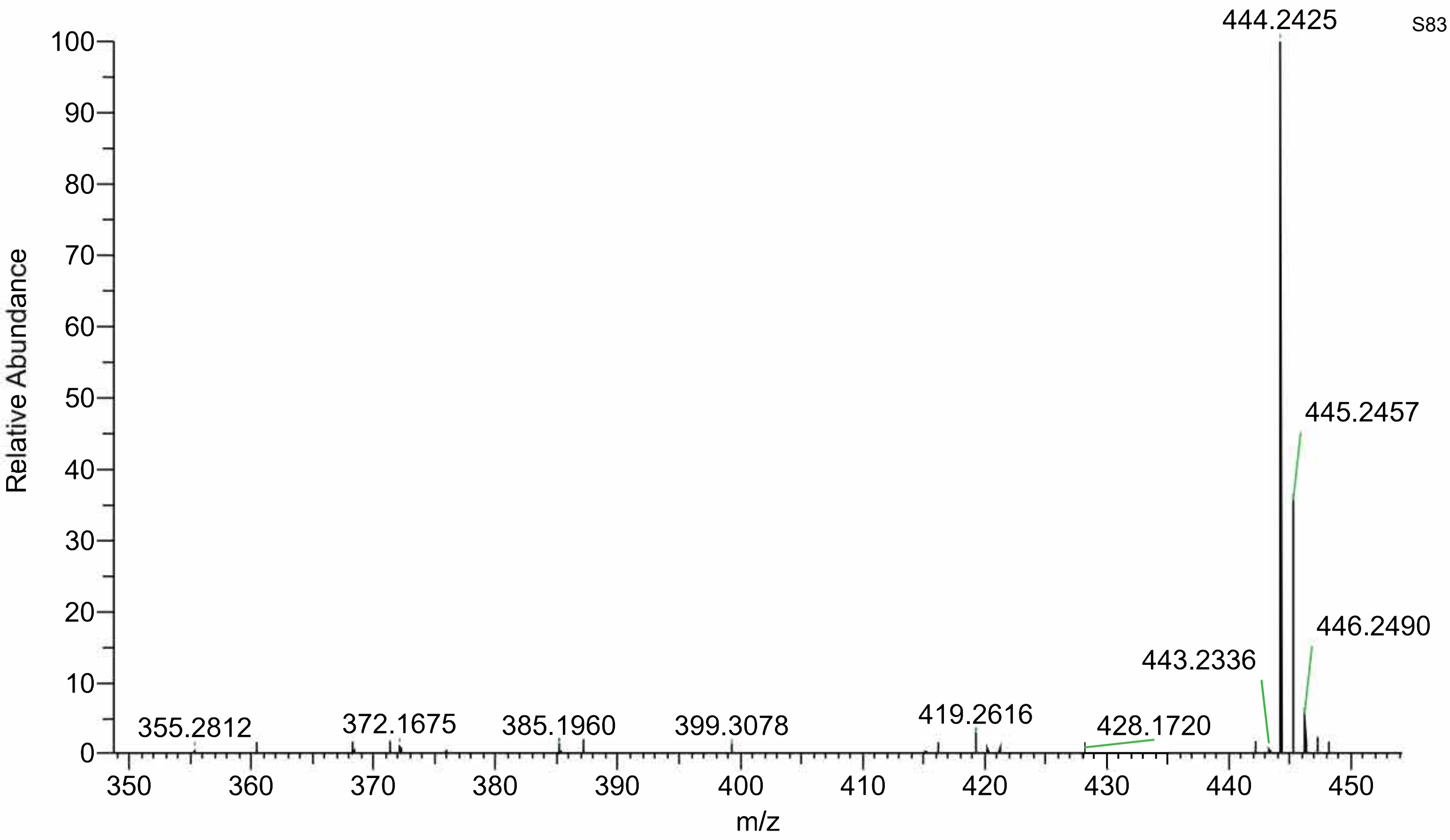

Figure S127. High resolution TOF-ESI mass spectrum of carbaporphyrin **18c**.

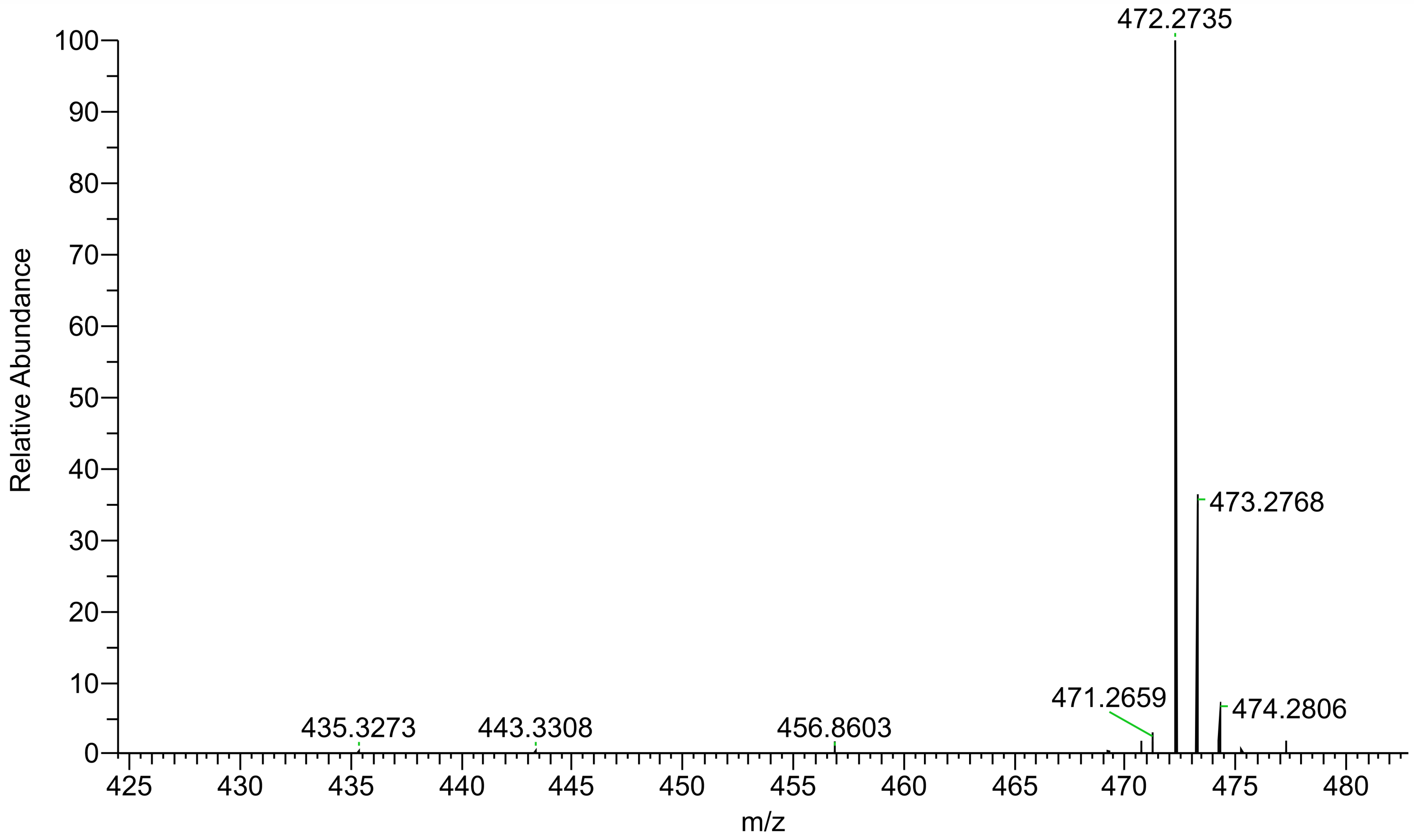

Figure S128. High resolution TOF-ESI mass spectrum of carbaporphyrin **18d**.

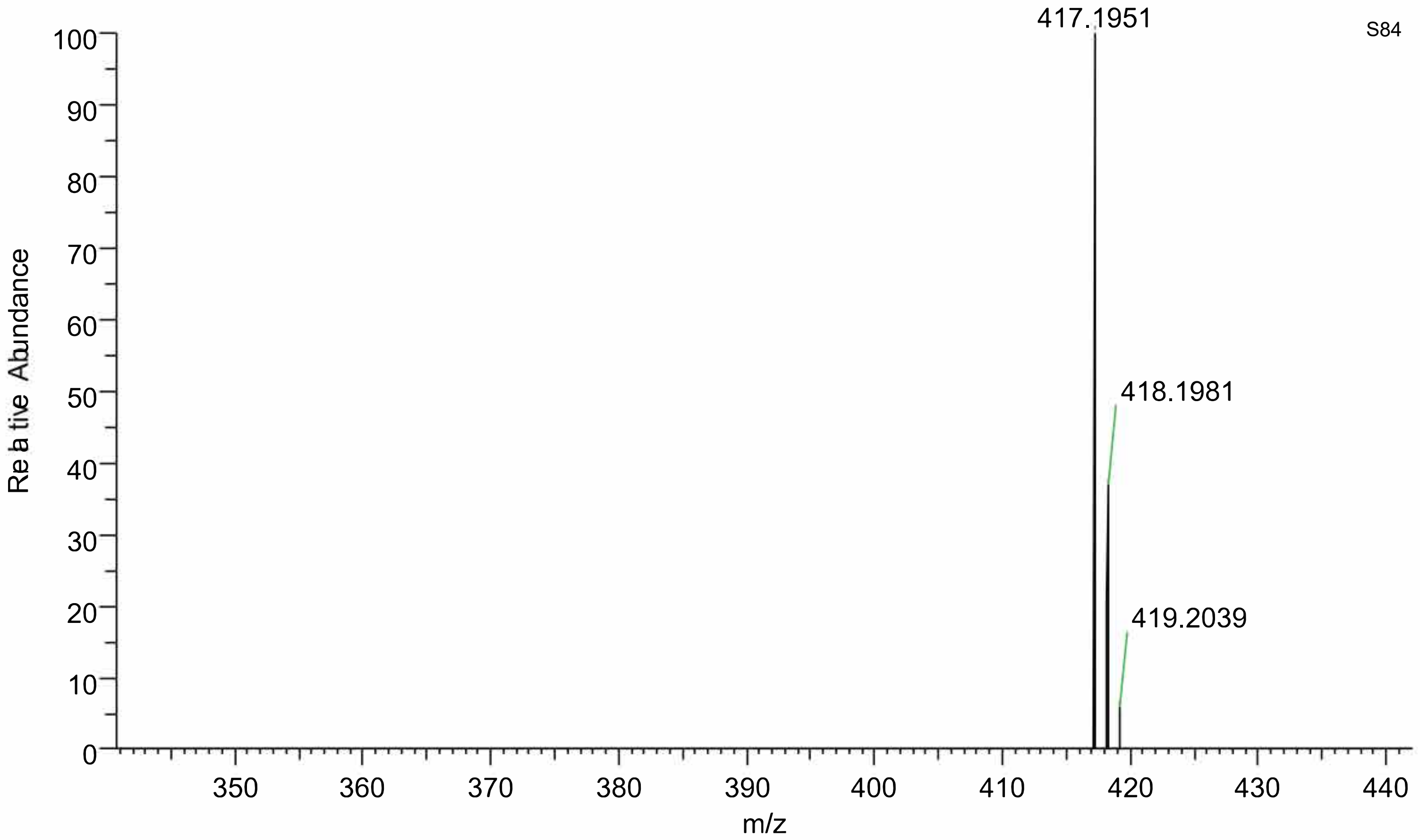

Figure S129. High resolution TOF-ESI mass spectrum of oxacarbaporphyrin **19a**.

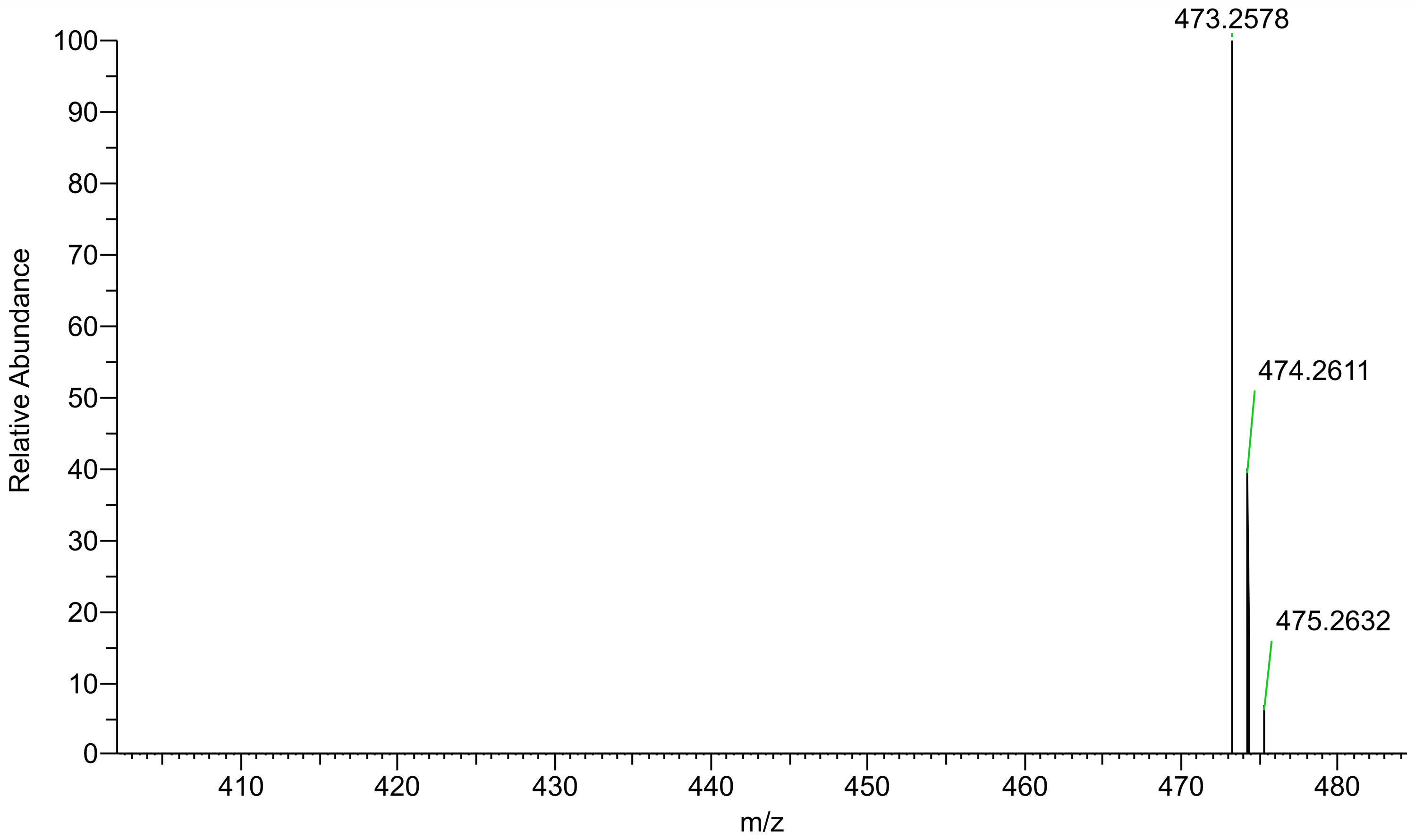

Figure S130. High resolution TOF-ESI mass spectrum of oxacarbaporphyrin **19b**.

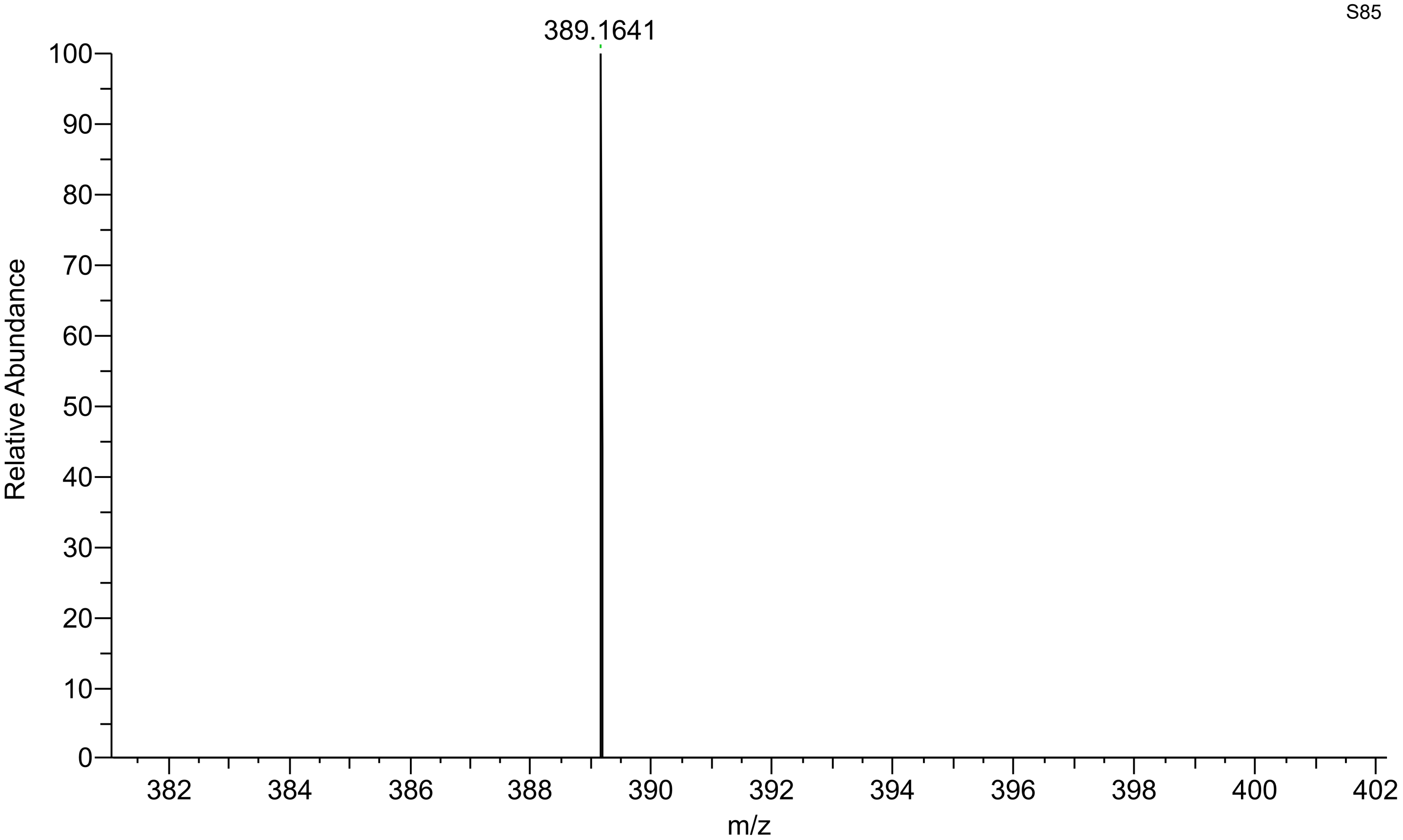

Figure S131. High resolution TOF-ESI mass spectrum of oxacarbaporphyrin **19c**.

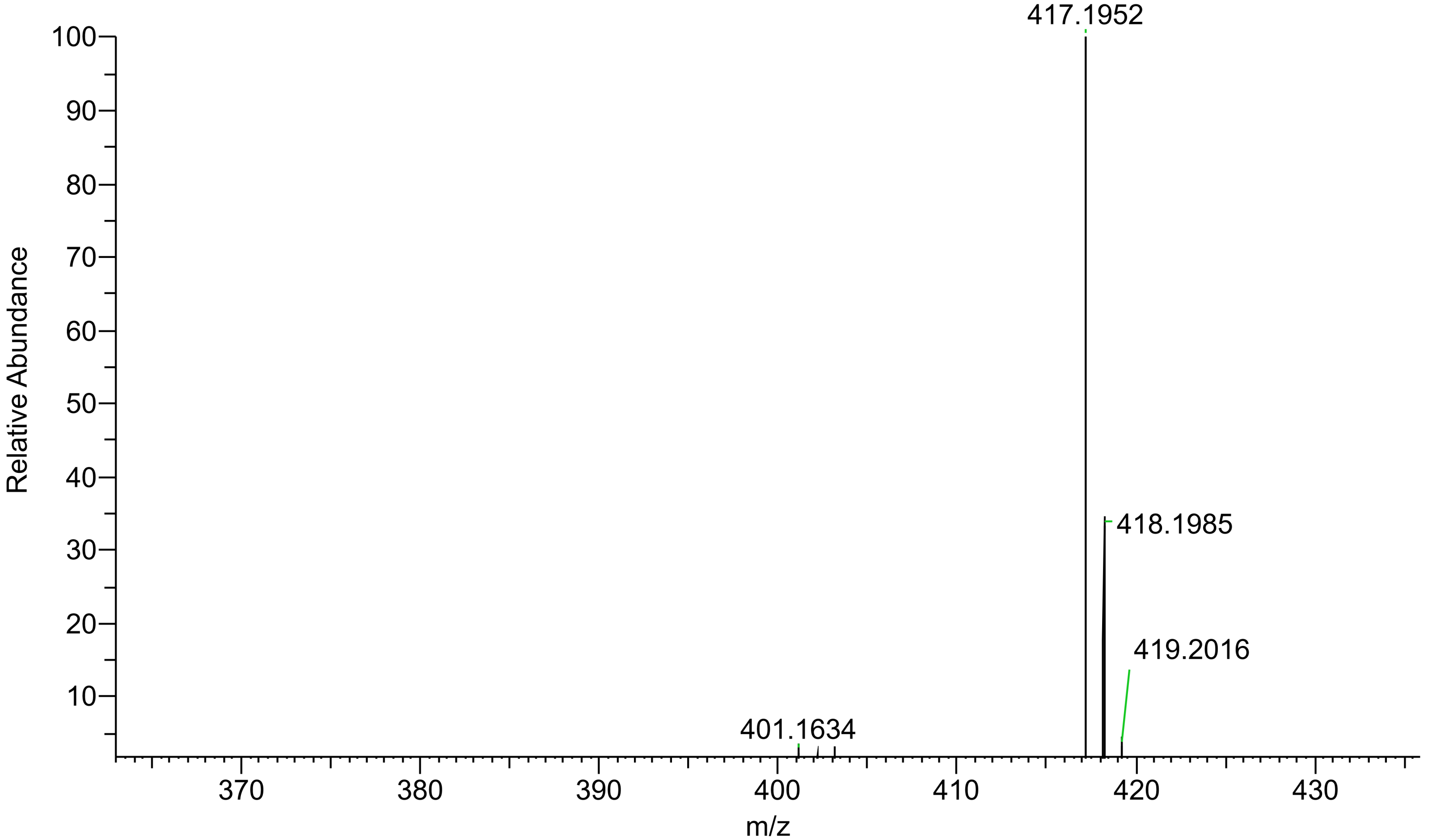

Figure S132. High resolution TOF-ESI mass spectrum of oxacarbaporphyrin **19d**.
